# Variation in movement patterns of mule deer: have we oversimplified migration?

**DOI:** 10.1186/s40462-021-00281-7

**Published:** 2021-08-26

**Authors:** Madelon van de Kerk, Randy T. Larsen, Daniel D. Olson, Kent R. Hersey, Brock R. McMillan

**Affiliations:** 1 Department of Plant and Wildlife Sciences, Brigham Young University, 4105 Life Sciences Building, Provo, UT 84602 USA; 2 Utah Division of Wildlife Resources, 1594 W North Temple, Suite 2110, Salt Lake City, UT 84114 USA; 3 Present Address: School of Environment and Sustainability, Western Colorado University, Kelley Hall 144, Gunnison, CO 81231 USA

**Keywords:** Migration, Movement patterns, Migratory continuum, Net squared displacement, Mule deer, *Odocoileus hemionus*

## Abstract

**Background:**

Conservation and management of migratory animals has gained attention in recent years, but the majority of research has focused on stereotypical ‘migrant’ and ‘resident’ behaviors, often failing to incorporate any atypical behaviors or characterize migratory behaviors beyond distance and timing of the migration. With migration threatened by anthropogenic development and climate change, it is crucial that we understand the full range of migratory behaviors. Our objective was to demonstrate and characterize the variation in migration strategies, including typical and atypical migratory behaviors for mule deer (*Odocoileus hemionus*) in Utah, USA.

**Methods:**

Because calculation of common metrics such as distance, timing, and use of stopovers during migration did not adequately describe the variation we observed in migratory behavior for this species—particularly when animals visited multiple (> 3) ranges for extended lengths of time—we developed additional methods and categories to describe observed variation in migratory behavior. We first categorized trajectories based on the number of discrete, separate ranges and range shifts between them. Then, we further characterized the variation in migration strategies by examining the timing, duration, and distance traveled within each of the categories. We also examined if and how frequently individual deer switched among categories from year to year.

**Results:**

We classified 1218 movement trajectories from 722 adult female mule deer, and found that 54.4% were dual-range migrants, who made one round-trip to one distinct range. Multi-range migrants (23.6%) made one round-trip during which they stayed at multiple discrete ranges. Commuters (1.0%) traveled to the same range multiple times, and poly migrants (1.5%) made multiple round-trips to different ranges. Gradual movers (2.5%) did not show a discrete range shift but moved gradually between ranges, whereas residents (12.6%) never left their home ranges, and dispersers (4.4%) left but never returned. Of the deer that we monitored for multiple years, 51.2% switched among categories.

**Conclusion:**

We conclude that the substantial number of atypical migratory strategies, as well as the number of deer that switched categories, underlines the importance of studying these less-stereotyped behaviors that may be exhibited by large proportions of populations. Acknowledging and investigating the full complexity and diversity in migratory strategies might uncover unknowns with respect to underlying factors and drivers of migration, and can help shape effective conservation strategies.

**Supplementary Information:**

The online version contains supplementary material available at 10.1186/s40462-021-00281-7.

## Background

Migration of large herbivores has gained attention in recent years, with management strategies aiming to reduce habitat fragmentation and movement barriers in order to maintain corridors between summer and winter ranges [1–3]. The loss of migration routes can be detrimental to population and community structures as well as plant–herbivore dynamics [4]. Additionally, we could lose one of the most iconic phenomena in the natural world, which may be impossible to restore [5, 6]. Despite an increasing amount of research, there are still many factors of migration that are not understood [7, 8]. For example, several authors have claimed that classic examples of typical migratory behavior (i.e. large-scale, seasonal movements between two discreet ranges) are probably the exception rather than the rule for many species, and that migration should be viewed as a continuum of behaviors ranging from resident to migrant [9, 10]. However, the majority of migration studies focus only on typical resident or typical migratory behaviors, failing to incorporate any atypical migratory movements [11, 12]. With migration threatened by anthropogenic development and climate change, it is crucial that we understand the full range of migratory behaviors [12].

Seasonal migration is generally considered to be a strategy to deal with spatiotemporal variation in resources and other selection pressures such as predators and parasites [7, 8, 13, 14]. For example, migratory individuals can have improved demographic rates compared to residents, although much remains to be learned [15, 16]. Similarly, many questions remain regarding the stimuli that trigger migration. Some evidence indicates that animals initiate migratory movements in response to changes in snowpack and forage quality and quantity [13, 17]. If these are the proximate reasons for migration, one would expect migratory behavior to vary across space and time in relation to these factors. Indeed, migratory behavior appears to vary along environmental gradients [18], and some animals are known to migrate in some years but not in others [19]. Trade-offs between the costs and benefits of migration can also result in partial migration, where one portion of a population remains resident and another portion migrates [20, 21]. Although recent studies have improved our understanding of migratory behavior dramatically, most of our knowledge still relies extensively on classic examples of stereotypical migration [9]. Studying the full range of migratory behaviors would allow for improved conservation of migrant and partially-migrant species [9, 11].

As most studies only consider typical examples of classic migration, little effort has been made to define exactly what type of movements can be considered migratory [11, 12]. Traditional views hold that migrations occur twice a year during fall and spring (at northern latitudes) to and from the winter range, and that migratory movements take place between separate, discrete ranges with perhaps some stopovers along the way. Animals that do not follow this typical pattern are often not considered in analyses. For example, Bischof et al. [8] and Mysterud et al. [22] excluded animals that exhibited ‘irregular’ or ‘ambiguous’ movement patterns, whereas both Monteith et al. [23] and Peters et al. [18] censored animals that migrated outside of the typical season. A recent review by Xu et al. [24] considered plasticity in migratory propensity as well as timing and destination, but failed to consider animals that might take multiple migratory trips and/or have multiple destinations. Cagnacci et al. [12] considered different methods to classify migrations and found that they only succeeded at identifying ‘neat and stereotypic’ migratory behaviors, but not atypical ones. They argued that, as these atypical behaviors appear to be the rule rather than the exception for ungulates, a conceptual framework of the full range of ungulate migratory behaviors should be adopted, rather than forcing methods to identify stereotyped movement patterns [12]. Although identifying and categorizing unique movement behaviors has been done for dispersal tactics, no comprehensive framework has been suggested for migration trajectories [25].

By excluding atypical migrations, we may be oversimplifying a complex behavioral phenomenon to the point that it negatively affects our inference [26]. Depending on the frequency of atypical migratory behavior as well as the biological question asked, assuming that all individuals that migrate follow the classic pattern could result in incorrect conclusions. This could potentially lead to ineffective conservation strategies, as well as inaccurate estimates of factors such as population connectivity and gene flow [27]. For example, the typical pattern of migration is described as a seasonal two-way movement in response to spatiotemporal variation in food resources. With increasing understanding that variation exists in populations, this idea that migration is in response to resource availability may be insufficient for many patterns of movement that are migratory. Additionally, if animals exhibit plasticity in their migratory behavior between years, it seems more likely that they switch to an intermediate behavior rather than from one extreme to the other [9, 10]. Therefore, studying animals that exhibit atypical, intermediate migratory behaviors is a great opportunity to gain a better understanding of what drives migratory behaviors and how animals can adapt their strategies [9]. To do this, we need to categorize and quantify migratory patterns in standardized, reproducible ways, so that they can be analyzed and compared [11, 12].

Here, we demonstrate an approach to characterizing migration patterns using GPS location data from mule deer (*Odocoileus hemionus*). Mule deer are a culturally and economically important species distributed throughout the intermountain western United States [28, 29]. They are also one of only a handful of remaining long distance migrating mammals in North America, the preservation of which provides major challenges in the area with the fastest rate of human population growth in the United States [29, 30]. Mule deer exhibit substantial variation in their migratory behavior, with both resident, migrant, and mixed migration strategies occurring across populations [31, 32]. The average distance of their migration is 66 km [33], but distances up to 772 km have recently been observed in Wyoming, marking the third longest ungulate migration globally after caribou and reindeer [34]. Atypical migratory behaviors by mule deer have been largely overlooked or excluded in scientific literature, even though they have been observed [35]. Moreover, although migratory plasticity appears to be low for mule deer, only switches between the two extremes of resident and typical migratory behaviors, and changes in timing or destination have been assessed [24, 36].

Our objective was to characterize and demonstrate the variation in migratory behaviors of mule deer, including typical migration as well as any atypical behaviors. We defined different categories of migration strategies based on the number of ranges used each year and the number of range shifts between them. We then used an automated process to categorize 1218 annual movement trajectories made by 722 unique deer, and estimated distance, timing and duration of migration for the trajectories within each category. We also investigated the trajectories of deer that we monitored for multiple years to assess how often they switched among strategies and between which strategies switching occurred. Thereby, we elucidated shortcomings of current practices and paved the way for much needed research addressing the full range of migratory behaviors, and how they might change with climate change and continued anthropogenic development.

## Methods

### Data collection

Adult female mule deer (> 1 year old in early winter) were captured in the early winters of 2014–2019, between 16 November and 23 December of each year. We captured deer throughout the state of Utah, USA, between 37° and 42° latitude and − 114° and − 109° longitude, at elevations ranging from 670 m in southwestern Utah to 3500 m in the northeast. Deer were net-gunned from a helicopter, after which they were hobbled, blindfolded and transported to a nearby landing zone in a sling under the helicopter. There, deer were taken out of the sling and fitted with a global positioning system (GPS)-collar (model type G2110E2H, G5-2DH, or W300; Advanced Telemetry Systems, Isanti, MN, USA). The GPS collars were programmed to acquire a fix at intervals ranging from 0.5 to 13 h, and several deer were recaptured in later years to replace their collars with a newer model. Otherwise, captured deer were monitored until their death, or until their collar failed. All captures were conducted by a private capture company contracted by the Utah Division of Wildlife Resources. Deer were captured and handled in compliance with the requirements of the Institutional Animal Care and Use Committee for Brigham Young University (protocol 150110) and the guidelines for the use of wild mammals in research of the American Society of Mammalogists [37].

### Data preparation

For each individual, we split up the data into trajectories lasting one ‘migratory year’, which started at July 1 and ended on June 30. We chose July 1 as start of the year because mule deer parturition in Utah has finished at that time [38]. For purposes of this study, we refer to the range an animal is at on July 1st as their ‘summer range’ for that year. For a typical migrant, one migratory trajectory should include some time spent at the summer range followed by fall migration to the winter range, followed by a stay at the winter range and finally spring migration back to the summer range. Some animals were monitored during multiple migratory years and therefore occurred in the dataset more than once. For each migratory year we only included animals that were monitored the entire 12 months and excluded animals that only had partial data. We reduced the GPS data to one average location per day to eliminate the effect of variable fix intervals and reduce the influence of exploratory movements [18, 39]. We used the average daily locations to calculate the daily net squared displacement (NSD) values of the annual movement trajectories. The NSD measures the straight line distance between a starting location and every subsequent location, and is a fundamental statistic in movement analyses as it synthesizes key properties of an animal movement trajectory [40, 41]. The NSD pattern of a typical migrant shows a double-sigmoidal curve or hat shape: starting off at low values as the animal remains in its summer range, then rapidly increasing as it migrates to its winter range in fall, relatively stable again during winter, followed by a rapid decrease in spring as the animal migrates back to its summer range [8, 41]. The NSD plot of a resident animal remains flat, as it resides in the same area year-round. However, upon initial examination of our NSD plots, we found that many of them were not residents, but also did not show the double-sigmoidal curve of a typical migrant.

### Identifying distinct ranges and range shifts

Although Xu et al. [24] examined plasticity in distance and timing of migration of mule deer, they did not categorized migration strategies beyond migratory or resident. However, simple metrics of distance and timing do not adequately capture the variation in migratory behaviors when animals take multiple trips away from their summer range or visit more than two ranges, which many mule deer in Utah did. Therefore, our first step was to identify the number of distinct ranges used by each deer, as well as the number and timing of range shifts (for pseudocode, see Additional File 1). We automated the detection of any range shifts in annual movement trajectories in Program R [42] (for pseudocode, see Additional File 1). We treated the NSD as a time series and used the R package *strucchange* [43] to identify ‘breakpoints’, indicating points in time where there was a change in the mean NSD, thereby splitting the time series up in ‘segments’. We considered all possible segmentations for any number of breakpoints and identified the optimal breakpoints for each trajectory as the set of breakpoints with the minimum BIC [44].

To avoid classifying occasional sallies or stopovers during migration as a separate range, we set the minimum segment length to a set number of consecutive days, representing the minimum time required to be spent at a location for it to be considered a separate range. There are not any clear definitions in the literature regarding length of stopovers for mule deer, but Sawyer and Kauffman [45] described stopover ecology for this species. In their study area, average duration of spring and fall migrations was only ~ 21 days ±  ~ 8 days (95% CI). Moreover, total migration distances ranged from 18–144 km with stopovers occurring every 5.2 to 6.7 km, which creates an expectation of multiple stopovers per individual of < 11 days in duration [45]. Therefore, we set the minimum segment length to 14 consecutive days, and included a 14 day buffer before and after the migratory year to avoid misclassifying movements that occurred at the end and beginning of the year.

**Fig. 1:**
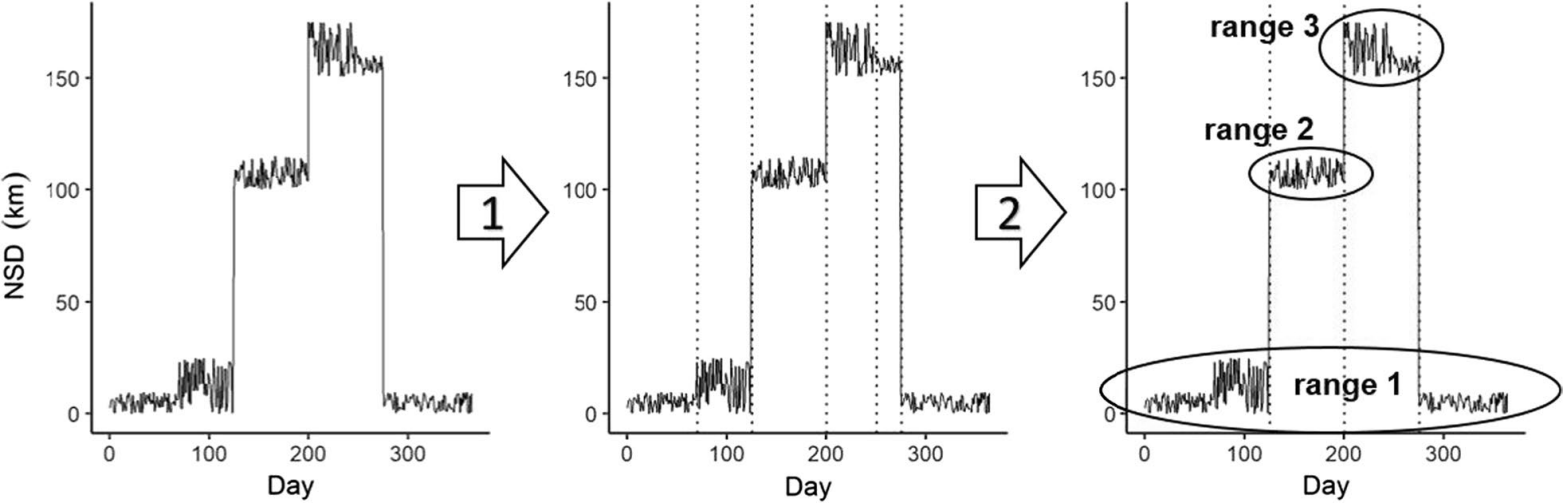
The process of analysis of an annual mule deer trajectory based on Net Squared Displacement (NSD). At step 1 the number of breakpoints (vertical dashed lines) is identified, at step 2 the segments that overlap are combined. This particular animal took one trip away from its summer range (range 1), during which it visited two other ranges (range 2 and 3) and was classified as a multi-range migrant

Not every breakpoint indicated a range shift, as a change in the mean NSD also occurs at a range expansion or shrinkage. Therefore, we used the R package *overlapping* [46] to calculate the overlap between the density distributions of NSD values among the segments. We considered segments to be the same range if the overlap was > 5% (Fig. 1) and disregarded the breakpoints between them. The remaining breakpoints were considered to indicate the timing of range shifts, with the segments in between shifts representing the amount of time spent at a particular range.

### Categorizing trajectories

**Fig. 2:**
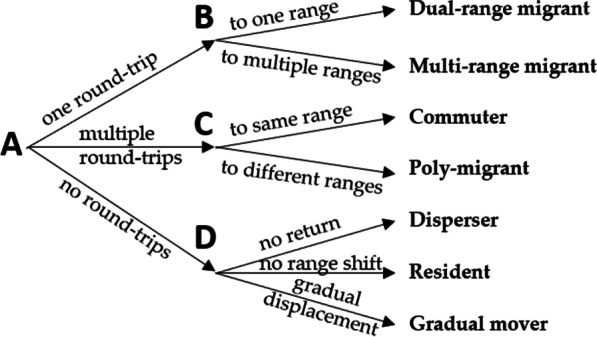
Decision tree indicating how we categorized movement trajectories into migration strategies for mule deer (*Odocoileus hemionus*) captured between 2014 and 2019 in Utah, USA

Our next step was to categorize the trajectories based on the ranges and range shifts we identified (for pseudocode, see Additional File 1). We grouped the trajectories into 7 distinct categories (Fig. 2). First, we split the trajectories up based on the number of round-trips taken (Fig. 2A). Each round-trip consisted of an “outbound movement”: a range shift where the animal leaves its summer range, as well as an “inbound movement”: a range shift where the animal returns back to its summer range. Trajectories encompassed either one, multiple, or no round-trips. Making one or more round-trips away from the summer range might allow animals to better exploit resources or avoid unfavorable conditions [47]. However, leaving a familiar range is inherently risky, and the risk increases with the number of round-trips taken [48].

The trajectories that encompassed one round-trip were then split up based on the number of ranges that were visited during that trip (Fig. 2B). *Dual-range migrants* visited one range during their trip in addition to their summer range, their annual trajectory comprising a total of two ranges. Deer that visited multiple distinct ranges during their trip were classified as *multi-range migrants*. Using multiple ranges during a trip requires additional range shifts and increases associated risks, but might also allow for better tracking of resources like plant phenology, also called green wave surfing or jumping [8].

The trajectories that encompassed multiple round-trips were split up based on the destinations of those trips (Fig. 2﻿C). Deer that visited the same range during each of their trips were classified as *commuters*, whereas *poly-migrants* traveled to different ranges. Commuting between two ranges might allow animals to optimally exploit resources in both ranges, whereas making multiple round-trips to different ranges allow the animal to use resources in even more ranges, but at the cost of traversing more unfamiliar and potentially riskier terrain [47].

Finally, we grouped the deer that did not make any round-trips into 3 separate categories (Fig. 2D). *Dispersers* made a distinct outbound movement, but did not make an inbound movement back to their original summer range. Although dispersal is usually limited to younger age classes, adult mule deer also occasionally disperse [49]. *Residents* remained on one range the whole year, whereas *gradual movers* did not stay within a typical home range, as determined by their NSDs surpassing 25 km for at least 14 consecutive days (average mule deer home range size 24.95 km^2^ [50]). We distinguished between residents and gradual movers because theoretically, residents would be at lower risk always being on familiar terrain, whereas gradual movers might spend more time on unfamiliar terrain but can also use resources of a larger area.

After classifying all the trajectories, we calculated the percentage of trajectories in each category. For trajectories that included at least one round-trip, we estimated single and total combined trip lengths as well as the mean and maximum distance between ranges. We also investigated whether animals migrated during typical fall and spring time periods. Consistent with previous examinations of migration, we reasoned that a migrant should typically be on its winter range at the minimum during January–March, the coldest winter months with the most snow cover in Northern latitudes [32, 51]. In addition, migrants should be back on their summer range, where available forage is more abundant and nutritious during most of the year that snow is not present, prior to parturition in June [38, 52] and stay as long as possible to maximize growth prior to winter. Therefore, for animals that took a single trip, we estimated whether outbound migration took place during September–December, and inbound migration during April–June. Finally, we also examined the frequency at which individuals we monitored for multiple years switched among strategies, and between which strategies they switched.

## Results

We categorized 1218 movement trajectories of 722 unique adult female mule deer from July 2015 through June 2020 (for all categorized trajectories, see Additional File 2). Deer were tracked for an average of 20 months; 293 deer were included in the analyses for multiple years.

**Fig. 3:**
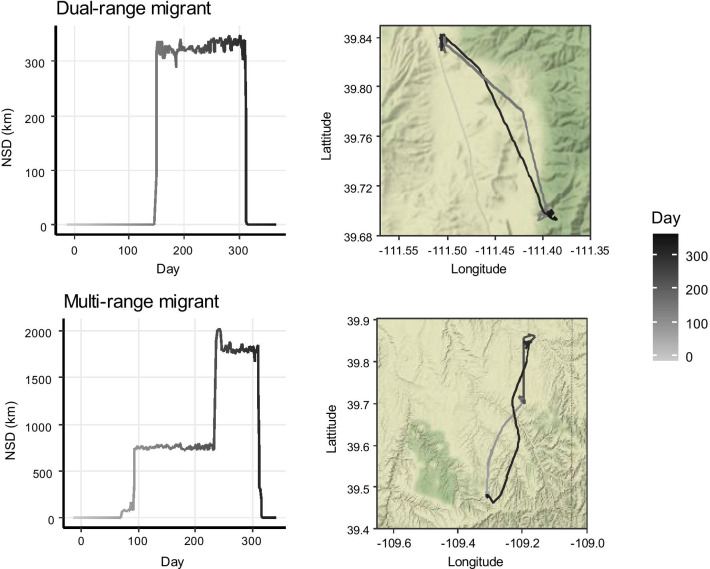
Examples of net-squared displacement (NSD) plots and movement trajectories for migration strategies comprising one distinct out and back trip observed in mule deer (*Odocoileus hemionus*) captured between 2014 and 2019 in Utah, USA. Categories include dual-range migrants (54.4% of 1218 trajectories) and multi-range migrants (23.6%)

**Fig. 4:**
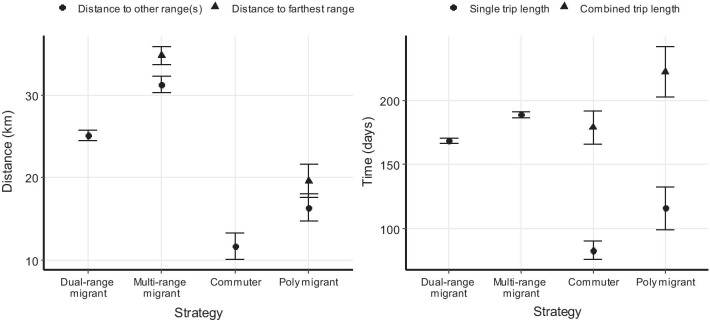
Average distance from summer range to any other range(s) that were visited as well as the distance to the farthest range for animals that visited multiple other ranges (left), and average trip length as well as total combined trip length for animals that took multiple trips away from the summer range (right), for mule deer (*Odocoileus hemionus*) captured between 2014 and 2019 in Utah, USA. Only trajectories that comprised at least one trip are included in this figure, and error bars indicate standard error of the mean

The majority of deer (n = 949, 77.9%, Fig. 3) made one round-trip away from its summer range. *Dual-range migrants* (n = 662, 54.4% of total) visited one range during this trip, whereas *multi-range migrants* (n = 287, 23.6% of total) visited multiple separate ranges. Departure dates for outbound movements, as indicated by the breakpoint indicating the first range shift and start of the trip, averaged November 9 ± 1.70 (SE) days and October 30 ± 2.20 days for *dual*- and *multi-range migrants*, respectively. Inbound movements, indicated by the second breakpoint at the end of the trip, started on 26 April ± 0.93 days and 6 May ± 3.48 days. Movements took place during typical fall and spring time periods for 80.8% of *dual-range migrants* (n = 535, 43.9% of total) and 14.3% of *multi-range migrants* (n = 41, 3.4% of total). For *dual-range migrants*, the distance between the two ranges was on average 25.15 ± 0.65 km, and the trip length was 167.82 ± 2.13 days (Fig. 4). Trips of *multi-range migrants* lasted 188.67 ± 2.46 days and comprised 3.20 ± 0.03 ranges that were on average 31.30 ± 1.05 km from the summer range, with the farthest range being 34.82 ± 1.07 km away (Fig. 4).

**Fig. 5:**
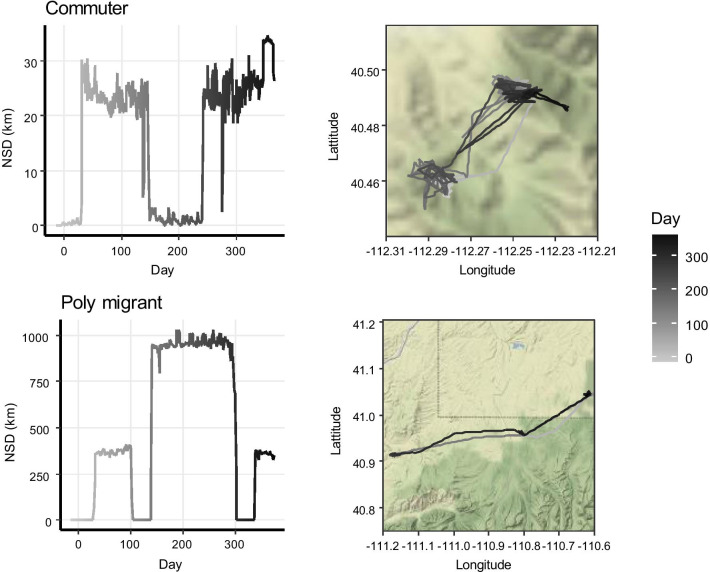
Examples of net-squared displacement (NSD) plots and movement trajectories for migration strategies comprising multiple distinct out and back trips observed in mule deer (*Odocoileus hemionus*) captured between 2014 and 2019 in Utah, USA. Categories include commuters (1.0% of 1218 trajectories) and poly migrants (1.5%)

Several trajectories (n = 32, 2.6%, Fig. 5) comprised not one but multiple round-trips away from the summer range. *Commuters* (n = 12, 1.0% of total) took multiple trips to the same range, whereas *poly-migrants* (n = 18, 1.5% of total) took trips to different ranges. *Commuters* took on average 2.21 ± 0.11 trips to the same range 11.67 ± 1.62 km away from their summer range, for a combined trip length of 178.71 ± 12.05 days. *Poly-migrants* took 2.05 ± 0.10 trips to 2.17 ± 0.12 different ranges that were on average 16.36 ± 1.67 km from their summer range, for a combined trip length of 221.94 ± 19.67 days (Fig. 4).

**Fig. 6:**
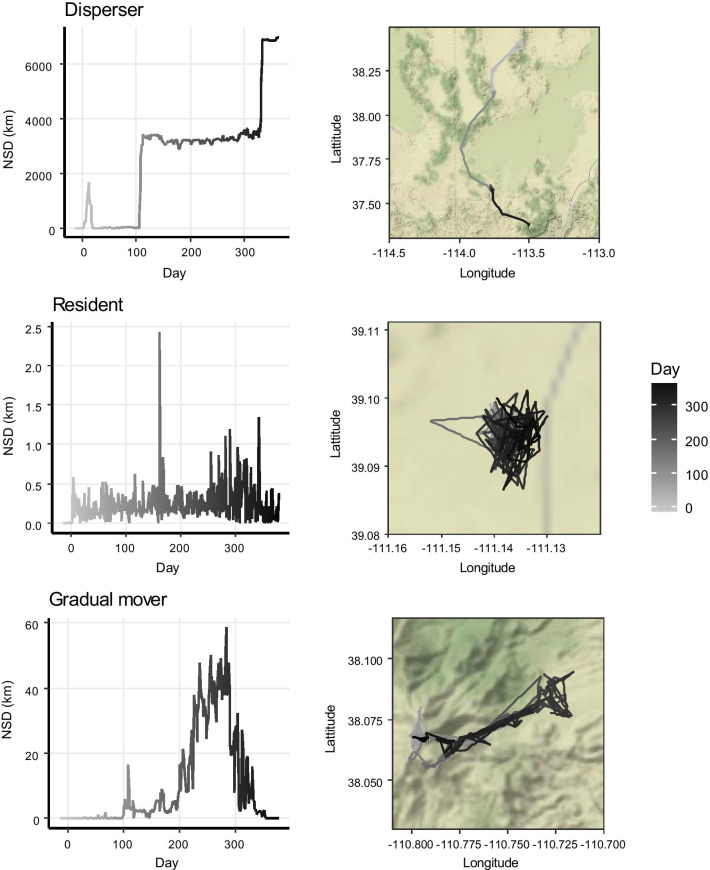
Examples of net-squared displacement (NSD) plots and movement trajectories for migration strategies not comprising any distinct out and back trips observed in mule deer (*Odocoileus hemionus*) captured between 2014 and 2019 in Utah, USA. Categories include dispersers (4.4% of 1218 trajectories), residents (12.6%) and gradual movers (2.5%)

A substantial number of movement trajectories (n = 237, 19.5%, Fig. 6) did not encompass any round-trips. Among this category, *dispersers* (n = 53, 4.4% of total) showed only an outbound movement but no inbound movement back to their original summer range. They left their summer range around 14 October (± 13 days) and used 2.21 ± 0.15 other ranges. For the remaining trajectories (n = 184), no significant range shifts were detected, including the *residents* (n = 153, 12.6% of total), who stayed at one range, and the *gradual movers* (n = 31, 2.5% of total), who exhibited gradual range shifts. *Residents* showed an average displacement of 1.55 ± 0.07 km, whereas *gradual movers* displaced and average of 8.03 ± 0.92 km throughout the year.

Out of 722 unique deer we monitored for at least a year, 293 deer were tracked for multiple years. The majority of these deer (51.2%) exhibited more than one unique strategy (Table 1). Between successive years, deer switched among categories 41.9% of the time (Table 2).

**Table 1:** Number of mule deer (*Odocoileus hemionus*) captured between 2014 and 2019 in Utah, USA that we monitored for 2, 3, or 4 consecutive years (n), and the percentage that used 1, 2, 3, or 4 different strategies among years

Years monitored	1 strategy	2 strategies	3 strategies	4 strategies	Total n
2 years	52.2	47.8	–	–	138
3 years	50.5	41.1	8.4	–	107
4 years	35.4	45.8	14.6	4.2	48

**Table 2:** Transition matrix indicating the percentage of mule deer (*Odocoileus hemionus*) captured between 2014 and 2019 in Utah, USA that exhibited a specific strategy in one year (column) and switched to another strategy in the next year (row) or exhibited the same strategy again (main diagonal, italic)

	Dual-range migrant	Multi-range migrant	Commuter	Poly-migrant	Disperser	Resident	Gradual mover
Dual-range migrant	*67.8*	45.5	25.0	20.0	45.5	21.6	66.7
Multi-range migrant	21.2	*45.5*	25.0	40.0	18.2	9.5	16.7
Commuter	0.7	0.9	*0.0*	0.0	0.0	0.0	0.0
Poly-migrant	0.7	1.8	0.0	*20.0*	4.5	0.0	0.0
Disperser	4.8	1.8	0.0	20.0	*22.7*	2.7	0.0
Resident	3.7	3.6	25.0	0.0	4.5	*62.2*	16.7
Gradual mover	1.1	0.9	25.0	0.0	4.5	4.1	*0.0*

## Discussion

It has been widely acknowledged that a complete understanding of the behaviors and drivers resulting in different kinds and degrees of migration should be a focus point of animal movement research [11, 53]. However, the majority of recent migration studies have focused on effects of environmental factors on the decision of whether to migrate or not, thereby largely ignoring the occurrence of different migratory strategies beyond stereotyped resident and migrant behaviors, and discarding data from animals whose movement patterns do not fit these categories [8, 12, 18, 22, 23]. Additionally, the lack of clear definitions and criteria defining migration patterns prevents consistently categorizing and comparing animals across studies and species [11]. We developed categories of migratory behavior based on unique criteria and found that, out of 1218 annual migration trajectories, 87.4% could be considered migratory, but only half of those fit the typical pattern of ‘classic’ migration. The substantial number of atypical migratory strategies underlines the importance of acknowledging and studying these less-stereotyped behaviors exhibited by a majority of migrants.

Only 43.9% of all the trajectories in our study could be considered typical migrants, who made one round-trip to a single winter range and migrated during fall and spring, respectively. Another 10.4% were *dual-range migrants* that traveled outside of the typical time periods. The largest group of atypical behaviors were the *multi-range migrants* (23.6%); individuals that did not migrate to a single winter range, but instead migrated to several different ranges. Although there are not any clear definitions in the literature regarding duration of stopovers, we chose a conservative 14 day limit based on data available for mule deer [45]. If a longer duration were chosen, then some of these *multi-range migrants* could be classified as typical migrants. The animals we labeled as *multi-range migrants*, however, used intermediate ranges for longer duration (often many months) than has been described for stopovers with this species [45]. Moreover, many of these animals spent more time on intermediate ranges than they did on either summer or winter ranges each year and it seems appropriate to label those behaviors as use of multiple ranges as opposed to simply stopover areas. *Poly migrants* (1.5%) also visited multiple ranges but did so during not one but multiple round-trips. *Commuters* (1.0%) only visited one range besides the summer range, but made multiple trips to that separate range. *Dispersers* (4.4%) left their summer range but never returned, even though this study only included adults (≥ 2 years old at start of summer) whereas mule deer are thought to disperse as yearlings [49]. Finally, *gradual movers* (2.5%) did not show any distinct range shifts but did not fit the *resident* (12.6%) category because their movements covered an area substantially bigger than a typical home range [50]. Although we did not explicitly include a category of ‘nomads’, which can be broadly defined as making irregular, non-migratory long-distance movements [54], both our *disperser* and our *gradual mover* category might fit this label.

Our results are mostly descriptive in nature and several patterns of movement illustrated herein lack a clear linkage to ecological significance. Based on previous examinations of migration in mule deer [2, 23, 32, 36] and hypothesized patterns of cultural transmission from adult to offspring [5], we were expecting animals to use mostly typical patterns of movement (e.g., resident, one-time migrant, or one-time migrant with stop overs) and be consistent among years. Our results illustrate that there is considerable variation among individuals and plasticity within individuals that is not explained by a single overarching idea or concept (e.g., access to high quality food or reducing the risk of predation [4]). The different migratory behaviors exist likely because there is, or was, an increase in fitness of an individual or group of individuals that adopted the behavior. Future research should focus on understanding the ecological significance of various patterns of migration to enhance conservation. For example, the use of multiple ranges is important to note because one cannot arbitrarily select one range to be managed as the winter range and disregard the others [10]. *Commuters* and *poly-migrants* might only need to escape winter conditions for short amounts of time, or migrate for reasons completely unrelated to weather or resource availability [11].

Utah is topographically, climatically and ecologically diverse, spanning a large variety ecoregions, many of which are representative of large parts of the mule deer’s range outside of Utah [55]. Thus, although no other studies reported proportions of atypical migratory behaviors of mule deer, we have no reason to believe mule deer in Utah behave differently than mule deer elsewhere. As the timing of mule deer migration is associated with factors such as winter severity and plant phenology [23], environmental diversity can result in heterogeneity in migratory behaviors [11, 22]. Indeed, we saw substantial variation among migratory trajectories. We also observed more plasticity than typically reported for this species, as the majority of deer switched among different strategies from one year to the next. This appears to contrast with a study by Sawyer et al. [36], who concluded that mule deer demonstrate little plasticity in terms of whether they migrate or not. However, they focused on switches between typical dual-range migrant and resident behavior, which were uncommon in our sample as well. Variation and plasticity in migration strategies may be a mechanism to avoid a mismatch with weather conditions in fall and plant phenology in spring [23]. Although the tracking of plant phenology in spring, also known as green wave surfing [2], has mostly been studied in the context of typical migration, atypical patterns may emerge when migrants are unable to track phenology closely due to landscape barriers (such as rivers and roads), discontinuities in resources, or risks such as vulnerability to predation. However, Bischof et al. [8] point out that any appropriately timed movement between ranges with different phenological development can provide a nutritional benefit, even without exploiting the full potential of the green wave. In addition to variation in timing, strategies that involve the use of multiple winter ranges could also be an adaptation to maximize resource use by balancing the benefits of migration with the risks and costs of moving. The costs of moving increase with distance, and multi-range migrants traveled farther than any other category. Climate variations may cause different migration strategies to be adaptive or maladaptive in different years [10]; one possible explanation for multiple strategies coexisting at the same time within a population.

Although simplifying complex behavior is often an acceptable and practical method to answer ecological questions, it could also lead us to disregard complexities that might hold the key to a full understanding of a complex phenomenon such as migration. Atypical migrants may represent an important part of the population, whose behavior might uncover unknowns on the underlying factors and drivers of migration [12]. Additionally, detailed investigations of migratory movement yield important information for conservation and management plans. For example, animals that move slower and more gradually between ranges might be less vulnerable to predation or starvation, but they may also have more opportunity to damage crops and cause conflict along their way [8]. Standardizing or simplifying migratory behavior can result in inadequate or even incorrect information resulting in ineffective management. For the migrants that used more than one winter range, for example, there was no ‘average’ range to be managed, nor can we easily decide which range is most important [10]. Therefore, more detailed description and parameterization of migratory behaviors is not only beneficial for migration research, but also of vital importance for maintaining and restoring this iconic phenomenon.

## Conclusion

By developing categories that encompass migratory strategies that fall in the typical migrant and resident categories as well as all atypical, mixed or intermediate strategies, we have helped elucidate the need for research addressing the entire range of migratory behaviors. One important question to answer would be how the proportion of different strategies relates to factors thought to affect migratory timing, such as winter severity and plant phenology. A related question is whether individual deer switch between strategies from year to year depending on certain intrinsic and external conditions to improve their fitness. Certain strategies could be restrained or prohibited by habitat alterations such as landscape barriers, resulting in a decrease of migratory diversity. Such a decrease may negatively affect populations because migratory diversity makes populations more resilient to environmental change and population declines [56], which is particularly important in the light of current climate change and anthropogenic pressures. Therefore, acknowledging and investigating the full complexity and diversity in migratory strategies can help shape effective conservation and management plans for populations at higher or lower levels of risk [36].

## Supplementary Information

**Additional file 1**. Pseudocode for trajectory classification.

**Additional file 2**. Net squared displacement plots and categories of all annual movement trajectories.

## Data Availability

R codes and daily net squared displacements for all animals included in this study will be made available at our website https://utahwildlife.shinyapps.io/analytics/.

## References

[1] Middleton AD, Kauffman MJ, McWhirter DE, Jimenez MD, Cook RC, Cook JG (2013). Linking anti-predator behaviour to prey demography reveals limited risk effects of an actively hunting large carnivore. Ecol Lett.

[2] Kauffman MJ, Copeland HE, Berg J, Bergen S, Cole E, Cuzzocreo M, et al. Ungulate migrations of the western United States, Volume 1. U.S. Geological Survey Scientific Investigations Report 2020–5101; 2020.

[3] Sawyer H, Lambert MS, Merkle JA (2020). Migratory disturbance thresholds with mule deer and energy development. J Wildl Manag.

[4] Fryxell JM, Sinclair ARE (1988). Causes and consequences of migration by large herbivores. Trends Ecol Evol.

[5] Jesmer BR, Merkle JA, Goheen JR, Aikens EO, Beck JL, Courtemanch AB (2018). Is ungulate migration culturally transmitted? Evidence of social learning from translocated animals. Scienc.

[6] Bartlam-Brooks HLA, Bonyongo MC, Harris S (2011). Will reconnecting ecosystems allow long-distance mammal migrations to resume? A case study of a zebra *Equus burchelli* migration in Botswana. Oryx.

[7] Nelson ME, Mech LD, Frame PF (2004). Tracking of white-tailed deer migration by global positioning system. J Mammal.

[8] Bischof R, Loe LE, Meisingset EL, Zimmermann B, van Moorter B, Mysterud A (2012). A migratory northern ungulate in the pursuit of spring: jumping or surfing the green wave?. Am Nat.

[9] Dingle H, Drake VA (2007). What is migration?. Bioscience.

[10] Ball JP, Nordengren C, Wallin K (2001). Partial migration by large ungulates: characteristics of seasonal moose *Alces alces* ranges in northern Sweden. Wildl Biol.

[11] Cagnacci F, Focardi S, Heurich M, Stache A, Hewison AJM, Morellet N (2011). Partial migration in roe deer: migratory and resident tactics are end points of a behavioural gradient determined by ecological factors. Oikos.

[12] Cagnacci F, Focardi S, Ghisla A, van Moorter B, Merrill EH, Gurarie E (2016). How many routes lead to migration? Comparison of methods to assess and characterize migratory movements. J Anim Ecol.

[13] Fryxell JM, Avgar T (2012). Animal migration: catching the wave. Nature.

[14] Mysterud A, Qviller L, Meisingset EL, Viljugrein H (2015). Parasite load and seasonal migration in red deer. Oecologia.

[15] Fryxell JM, Greever J, Sinclair ARE (1988). Why are migratory ungulates so abundant?. Am Nat.

[16] Rolandsen CM, Solberg EJ, Saether B-E, Van MB, Herfindal I, Bjørneraas K (2017). On fitness and partial migration in a large herbivore—migratory moose have higher reproductive performance than residents. Oikos.

[17] Sabine DL, Morrison SF, Whitlaw HA, Ballard WB, Forbes GJ, Bowman J (2002). Migration behavior of white-tailed deer under varying winter climate regimes in New Brunswick. J Wildl Manag.

[18] Peters W, Hebblewhite M, Mysterud A, Eacker D, Hewison AJM, Linnell JDC (2019). Large herbivore migration plasticity along environmental gradients in Europe: life-history traits modulate forage effects. Oikos.

[19] Fieberg J, Kuehn DW, DelGiudice GD (2008). Understanding variation in autumn migration of northern white-tailed deer by long-term study. J Mammal.

[20] Berg JE, Hebblewhite M, St. Clair CC, Merrill EH (2019). Prevalence and mechanisms of partial migration in ungulates. Front Ecol Evol.

[21] Eggeman SL, Hebblewhite M, Bohm H, Whittington J, Merrill EH (2016). Behavioural flexibility in migratory behaviour in a long-lived large herbivore. J Anim Ecol.

[22] Mysterud A, Loe LE, Zimmermann B, Bischof R, Veiberg V, Meisingset E (2011). Partial migration in expanding red deer populations at northern latitudes—a role for density dependence?. Oikos.

[23] Monteith KL, Bleich VC, Stephenson TR, Pierce BM, Conner MM, Klaver RW (2011). Timing of seasonal migration in mule deer: effects of climate, plant phenology, and life-history characteristics. Ecosphere.

[24] Xu W, Barker K, Shawler A, Van Scoyoc A, Smith JA, Mueller T (2021). The plasticity of ungulate migration in a changing world. Ecology.

[25] Ducros D, Morellet N, Patin R, Atmeh K, Debeffe L, Cargnelutti B (2020). Beyond dispersal versus philopatry? Alternative behavioural tactics of juvenile roe deer in a heterogeneous landscape. Oikos.

[26] Montgomery RA, Moll RJ, Say-Sallaz E, Valeix M, Prugh LR (2019). A tendency to simplify complex systems. Biol Conserv.

[27] Battey CJ. Seasonal migration, gene flow, and speciation in North American birds. PhD Dissertation, University of Washington, Seattle, USA; 2018.

[28] Rittenhouse CD, Mong TW, Hart T (2015). Weather conditions associated with autumn migration by mule deer in Wyoming. PeerJ.

[29] Robb B, Huang Q, Sexton JO, Stoner D, Leimgruber P (2019). Environmental differences between migratory and resident ungulates - predicting movement strategies in Rocky Mountain mule deer *(Odocoileus hemionus)* with remotely sensed plant phenology, snow, and land cover. Remote Sens.

[30] Rogers L, Nielsen L, Brown R (1988). Homing tendencies of large mammals: a review. Translocat wild Anim.

[31] Monteith KL, Bleich VC, Stephenson TR, Pierce BM, Conner MM, Kie JG (2014). Life-history characteristics of mule deer: Effects of nutrition in a variable environment. Wildl Monogr.

[32] Nicholson MC, Bowyer RT, Kie JG (1997). Habitat selection and survival of mule deer: tradeoffs associated with migration. J Mammal.

[33] Berger J (2004). The last mile: how to sustain long-distance migration in mammals. Conserv Biol.

[34] Joly K, Gurarie E, Sorum MS, Kaczensky P, Cameron MD, Jakes AF (2019). Longest terrestrial migrations and movements around the world. Sci Rep.

[35] Sawyer H, Middleton AD, Hayes MM, Kauffman MJ, Monteith KL (2016). The extra mile: Ungulate migration distance alters the use of seasonal range and exposure to anthropogenic risk. Ecosphere.

[36] Sawyer H, Merkle JA, Middleton AD, Dwinnell SPH, Monteith KL (2018). Migratory plasticity is not ubiquitous among large herbivores. J Anim Ecol.

[37] Sikes RS, Gannon WL (2011). Guidelines of the American Society of Mammalogists for the use of wild mammals in research. J Mammal.

[38] Freeman ED, Larsen RT, Peterson ME, Anderson CR, Hersey KR, Mcmillan BR (2014). Effects of male-biased harvest on mule deer: Implications for rates of pregnancy, synchrony, and timing of parturition. Wildl Soc Bull.

[39] Gurarie E, Cagnacci F, Peters W, Fleming CH, Calabrese JM, Mueller T (2017). A framework for modelling range shifts and migrations: asking when, whither, whether and will it return. J Anim Ecol.

[40] Turchin P (1998). Quantitative analysis of movement: measuring and modeling population redistribution in animals and plants.

[41] Bunnefeld N, Börger L, van Moorter B, Rolandsen CM, Dettki H, Solberg EJ (2011). A model-driven approach to quantify migration patterns: individual, regional and yearly differences. J Anim Ecol.

[42] R Development Core Team (2020). R: A language and environment for statistical computing. Version 3.6.3.

[43] Zeileis A, Leisch F, Hornik K, Kleiber C (2002). strucchange: an R package for testing for structural change in linear regression models. J Stat Softw.

[44] Zeileis A, Kleiber C, Krämer W, Hornik K (2003). Testing and dating of structural changes in practice. Comput Stat Data Anal.

[45] Sawyer H, Kauffman MJ (2011). Stopover ecology of a migratory ungulate. J Anim Ecol.

[46] Pastore M (2018). Overlapping: a R package for estimating overlapping in empirical distributions. J Open Source Softw.

[47] Denryter K, Stephenson TR, Monteith KL (2021). Broadening the migratory portfolio of altitudinal migrants. Ecology.

[48] Singh NJ, Borger L, Dettki H, Bunnefeld N, Ericsson G (2012). From migration to nomadism: movement variability in a northern ungulate across its latitudinal range. Ecol Appl.

[49] Robinette WL (1966). Mule deer home range and dispersal in Utah. J Wildl Manag.

[50] Webb SL, Dzialak MR, Houchen D, Kosciuch KL, Winstead JB (2013). Spatial ecology of female mule deer in an area proposed for wind energy development. West N Am Nat.

[51] Kunkel K., Stevens LE, Stevens SE, Sun L, Janssen E, Wuebbles D, et al. Regional climate trends and scenarios for the U.S. national Climate Assessment. Part 4. Climate of the U.S. Great Plains. NOAA Technical Report NESDIS 142–4. 2013.

[52] Lendrum PE, Anderson CR, Monteith KL, Jenks JA, Bowyer RT (2014). Relating the movement of a rapidly migrating ungulate to spatiotemporal patterns of forage quality. Mamm Biol.

[53] Bolger DT, Newmark WD, Morrison TA, Doak DF (2008). The need for integrative approaches to understand and conserve migratory ungulates. Ecol Lett.

[54] Teitelbaum CS, Mueller T (2019). Beyond migration: causes and consequences of nomadic animal movements. Trends Ecol Evol.

[55] Woods AJ, Lammers DA, Bryce SA, Omernik JM, Denton RL, Domeier M, et al. Ecoregions of Utah (color poster with map, descriptive text, summary tables, and photographs). Reston, Virginia, U.S. Geological Survey (map scale 1:1,175,000).; 2001.

[56] Gilroy JJ, Gill JA, Butchart SHM, Jones VR, Franco AMA (2016). Migratory diversity predicts population declines in birds. Ecol Lett.

