# Supplementary material for: Variation in movement patterns of mule deer: have we oversimplified migration?
Source: Mov Ecol. 2021 Aug 26;9:44. doi: 10.1186/s40462-021-00281-7 (PMC8394567; doi:10.1186/s40462-021-00281-7)
Supplement: Supplementary file 1 — Additional file 1. Pseudocode for trajectory classification. [file 40462_2021_281_MOESM1_ESM.docx]

**Additional File 1: pseudocode**

**Part I: identify ranges and range shifts**

FOR each trajectory

find all possible breakpoint combinations

WITH minimum segment length between breakpoints = 14

select breakpoint combination with highest AIC

calculate NSD density distribution of each segment between breakpoints

FOR each segment

calculate overlap with NSD densities of all other segments

IF overlap > 5% THEN

combine segments

ENDIF

ENDFOR

ENDFOR

**Part II: categorize each trajectory**

**Part IIA (corresponding to Figure 1A):**

FOR each trajectory

SET first segment: “summer range”

IF summer range occurs twice THEN

categorize as “one round-trip”

ELSE IF summer range occurs more than twice THEN

categorize as “multiple round-trips”

ELSE

categorize as “no round-trips”

ENDIF

ENDFOR

**Part IIB (corresponding to Figure 1B):**

FOR each ‘one round-trip’ trajectory

IF total number of ranges = 2 THEN

categorize as “dual-range migrant”

ELSE

categorize as “multi-range migrant”

ENDIF

ENDFOR

**Part IIC (corresponding to Figure 1C):**

FOR each ‘multiple round-trip’ trajectory

IF total number of ranges = 2 THEN

categorize as “commuter”

ELSE

categorize as “poly migrant”

ENDIF

ENDFOR

**Part IID (corresponding to Figure 1D):**

FOR each ‘no round-trips’ trajectory

IF total number of ranges > 1 THEN

categorize as “disperser”

ELSE

IF NSD > 25 for ≥14 successive days THEN

categorize as “gradual mover”

ELSE

categorize as “resident”

ENDIF

ENDIF

ENDFOR
