# Supplementary material for: Variation in movement patterns of mule deer: have we oversimplified migration?
Source: Mov Ecol. 2021 Aug 26;9:44. doi: 10.1186/s40462-021-00281-7 (PMC8394567; doi:10.1186/s40462-021-00281-7)
Supplement: Supplementary file 2 — Additional file 2. Net squared displacement plots and categories of all annual movement trajectories. [file 40462_2021_281_MOESM2_ESM.pdf]

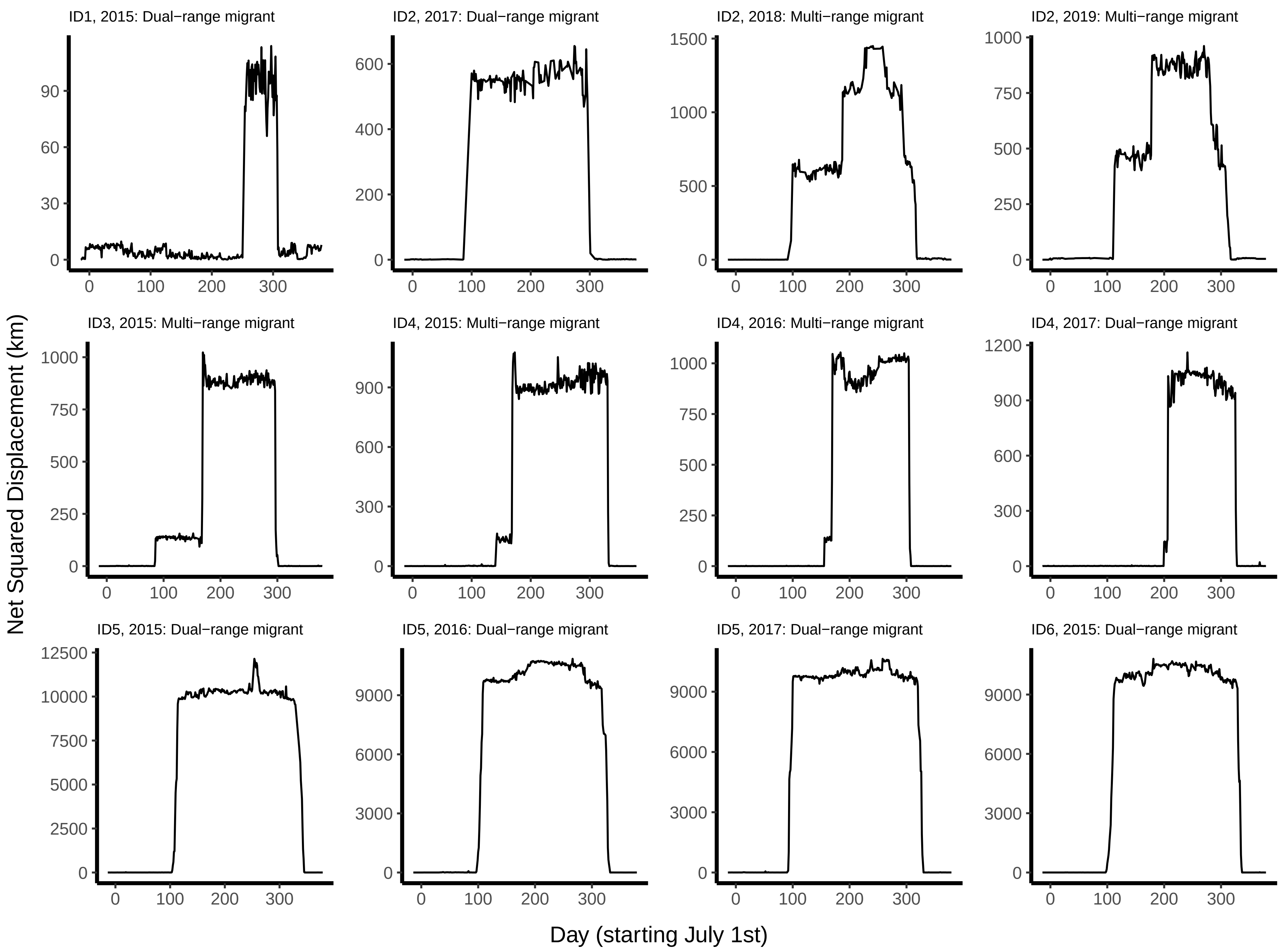
ID1, 2015: Dual−range migrant
ID2, 2017: Dual−range migrant
ID2, 2018: Multi−range migrant
ID2, 2019: Multi−range migrant
ID3, 2015: Multi−range migrant
ID4, 2015: Multi−range migrant
ID4, 2016: Multi−range migrant
ID4, 2017: Dual−range migrant
ID5, 2015: Dual−range migrant
ID5, 2016: Dual−range migrant
ID5, 2017: Dual−range migrant
ID6, 2015: Dual−range migrant
Net Squared Displacement (km)
Day (starting July 1st)

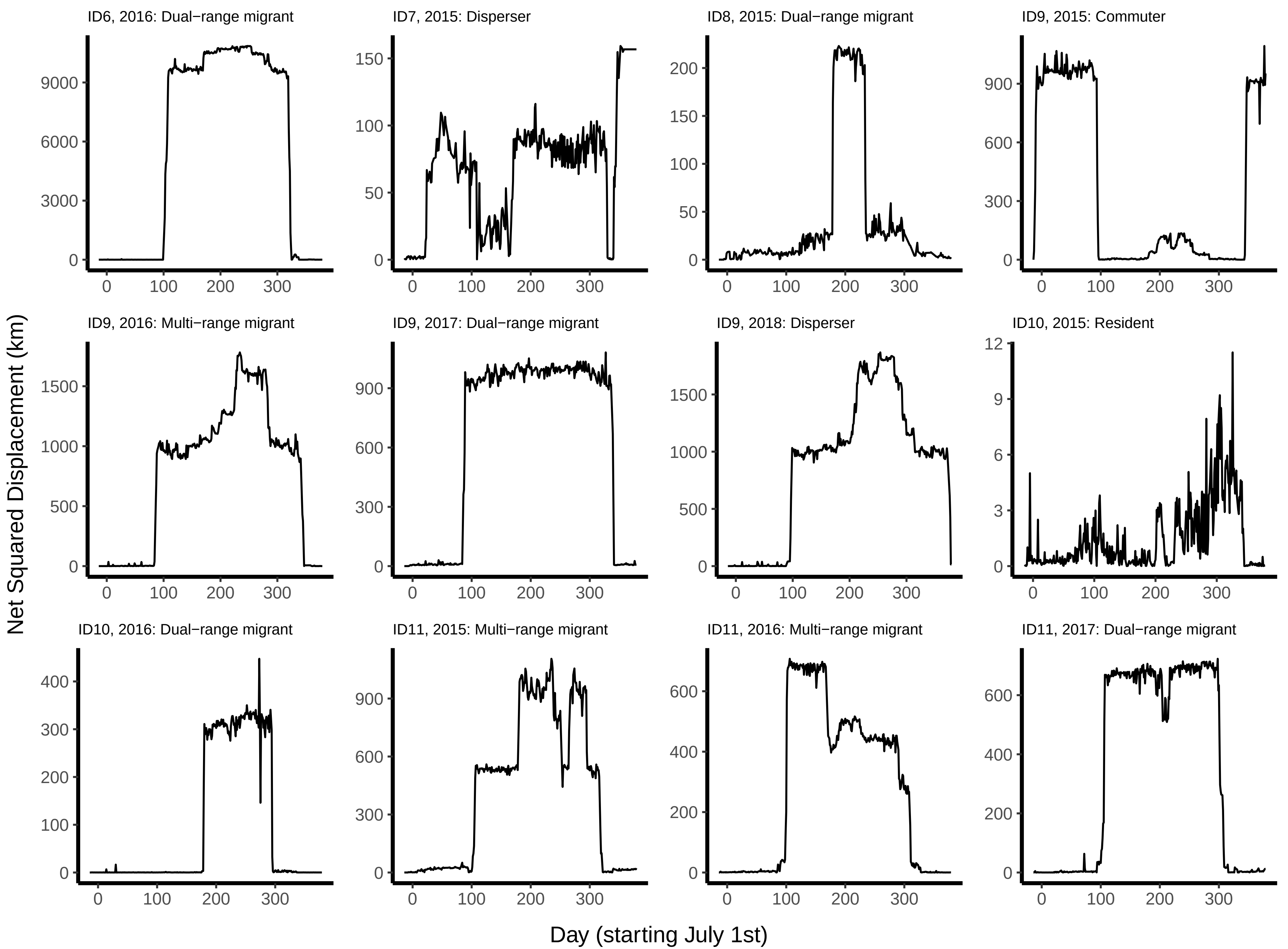

ID6, 2016: Dual−range migrant
ID7, 2015: Disperser
ID8, 2015: Dual−range migrant
ID9, 2015: Commuter
ID9, 2016: Multi−range migrant
ID9, 2017: Dual−range migrant
ID9, 2018: Disperser
ID10, 2015: Resident
ID10, 2016: Dual−range migrant
ID11, 2015: Multi−range migrant
ID11, 2016: Multi−range migrant
ID11, 2017: Dual−range migrant
Net Squared Displacement (km)
Day (starting July 1st)

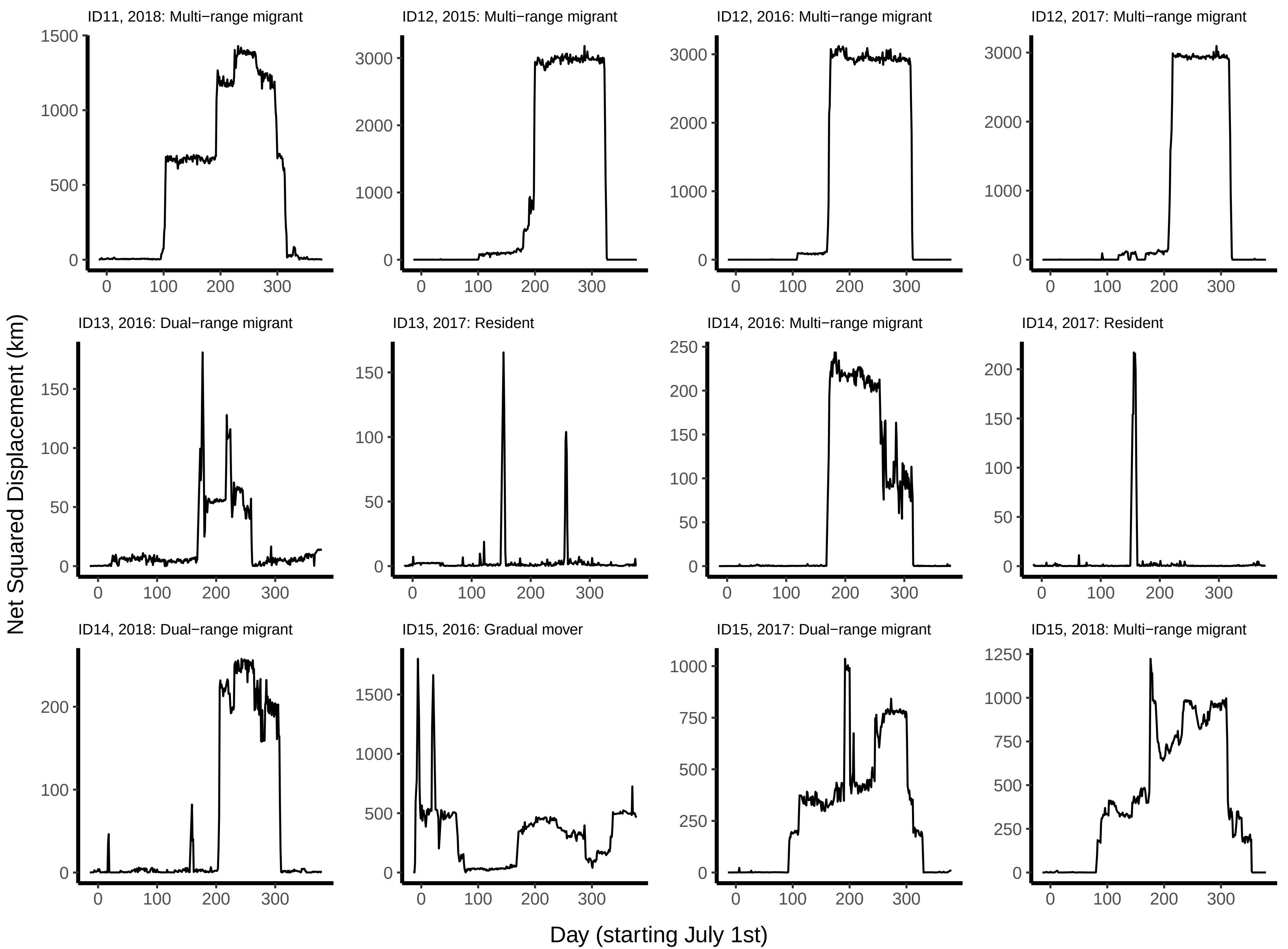

ID11, 2018: Multi−range migrant
ID12, 2015: Multi−range migrant
ID12, 2016: Multi−range migrant
ID12, 2017: Multi−range migrant
ID13, 2016: Dual−range migrant
ID13, 2017: Resident
ID14, 2016: Multi−range migrant
ID14, 2017: Resident
ID14, 2018: Dual−range migrant
ID15, 2016: Gradual mover
ID15, 2017: Dual−range migrant
ID15, 2018: Multi−range migrant
Net Squared Displacement (km)
Day (starting July 1st)

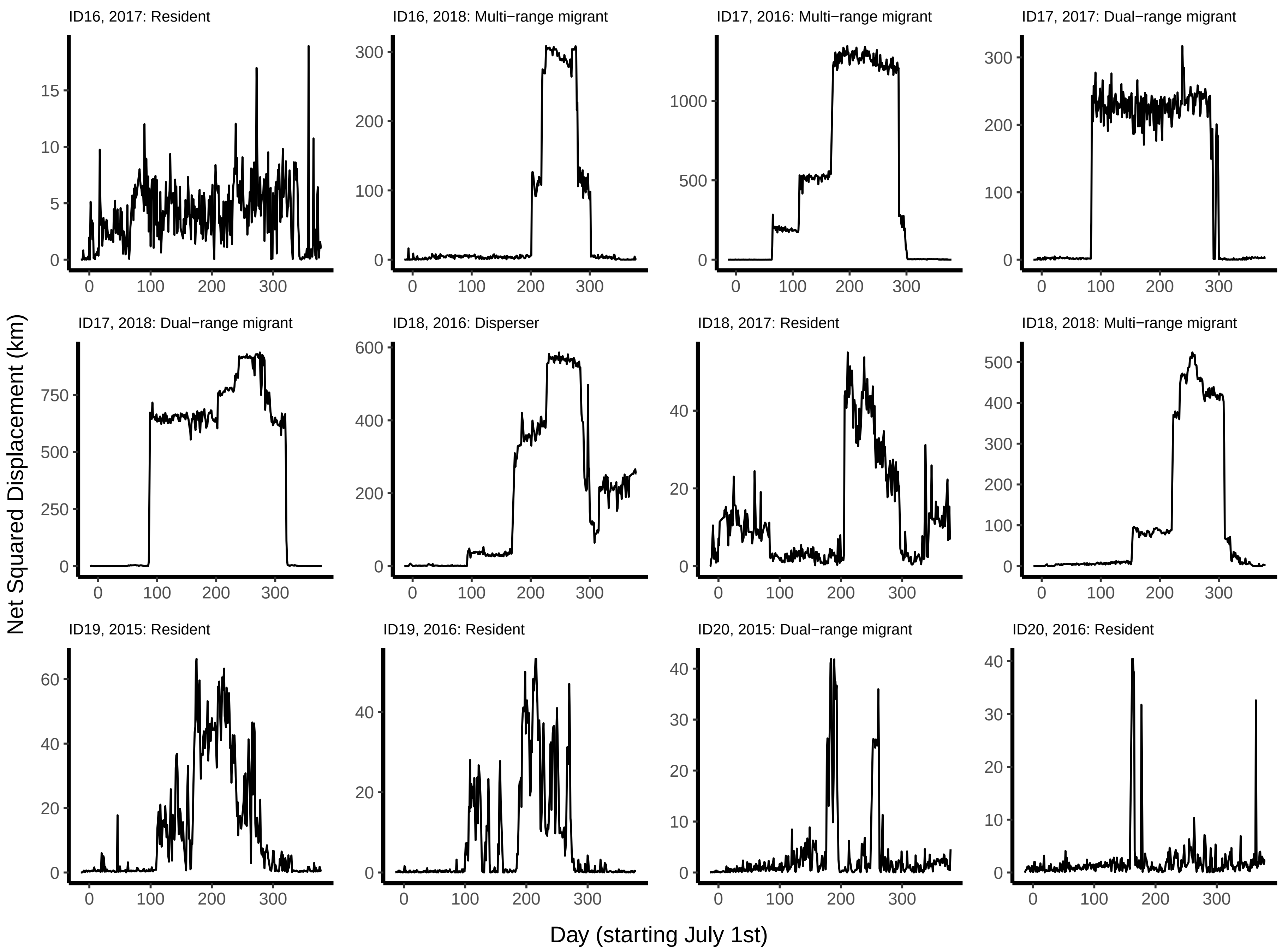

ID16, 2017: Resident
ID16, 2018: Multi−range migrant
ID17, 2016: Multi−range migrant
ID17, 2017: Dual−range migrant
ID17, 2018: Dual−range migrant
ID18, 2016: Disperser
ID18, 2017: Resident
ID18, 2018: Multi−range migrant
ID19, 2015: Resident
ID19, 2016: Resident
ID20, 2015: Dual−range migrant
ID20, 2016: Resident
Net Squared Displacement (km)
Day (starting July 1st)

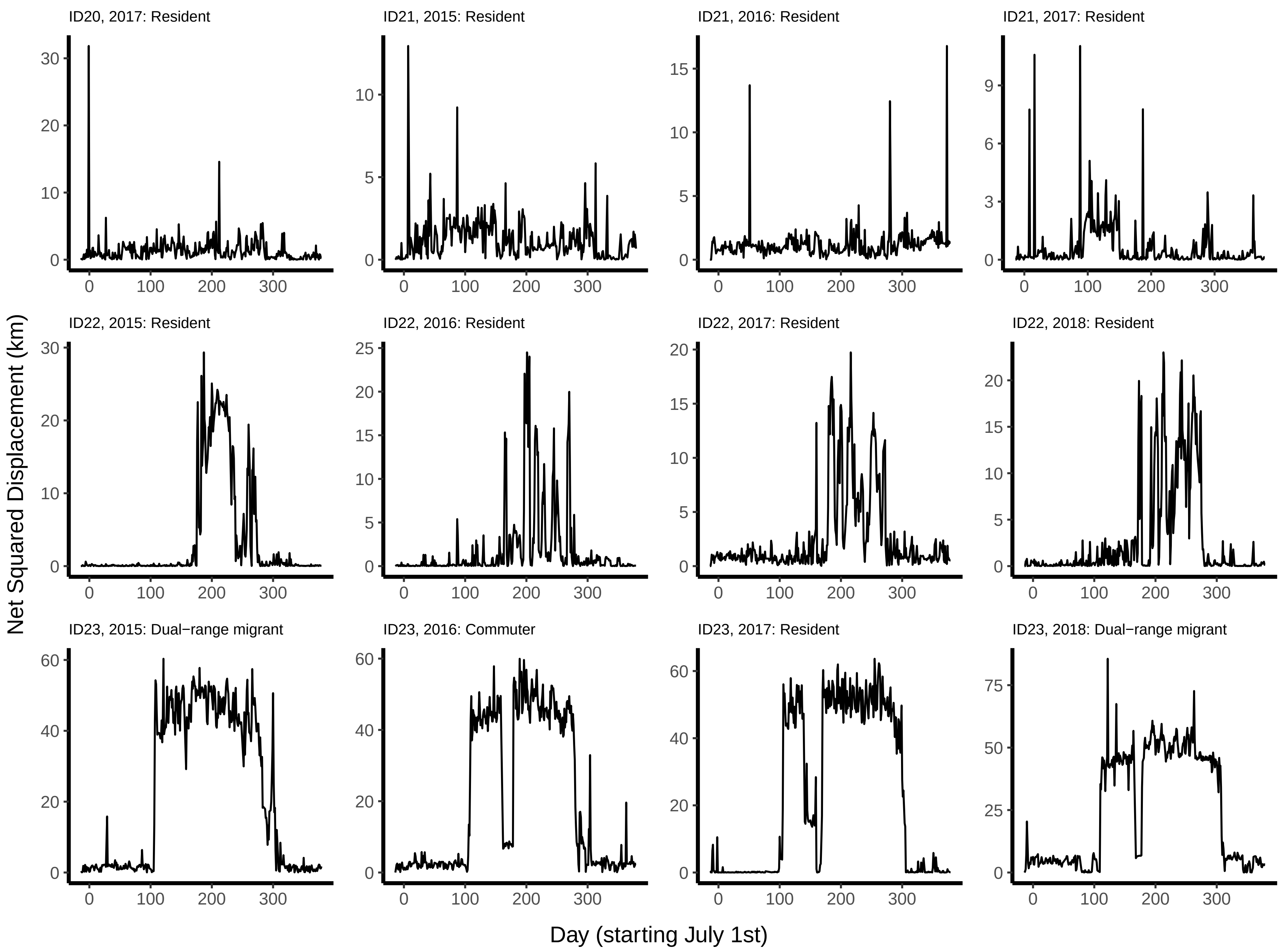

ID20, 2017: Resident
ID21, 2015: Resident
ID21, 2016: Resident
ID21, 2017: Resident
ID22, 2015: Resident
ID22, 2016: Resident
ID22, 2017: Resident
ID22, 2018: Resident
ID23, 2015: Dual−range migrant
ID23, 2016: Commuter
ID23, 2017: Resident
ID23, 2018: Dual−range migrant
Net Squared Displacement (km)
Day (starting July 1st)

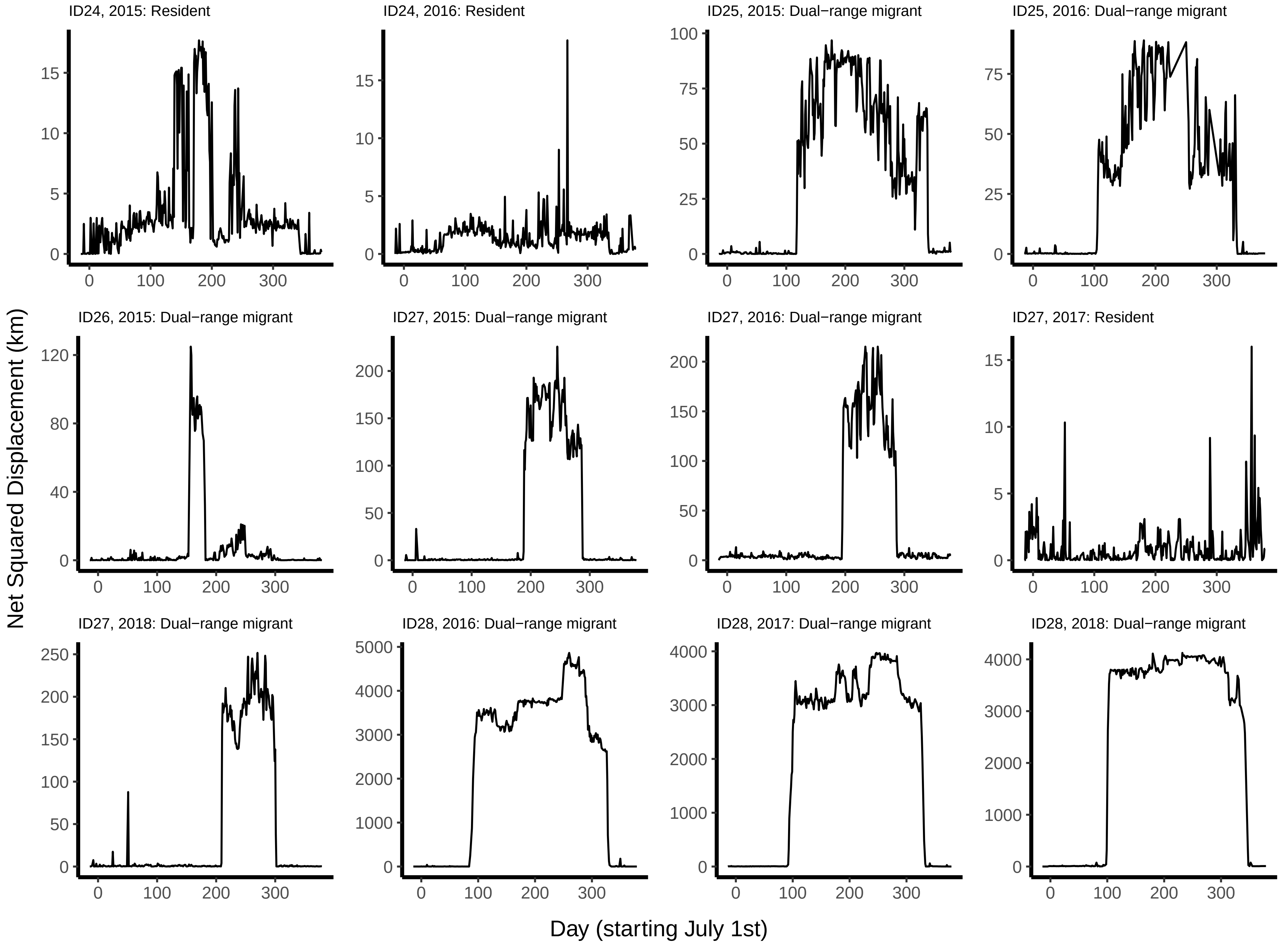
ID24, 2015: Resident
ID24, 2016: Resident
ID25, 2015: Dual−range migrant
ID25, 2016: Dual−range migrant
ID26, 2015: Dual−range migrant
ID27, 2015: Dual−range migrant
ID27, 2016: Dual−range migrant
ID27, 2017: Resident
ID27, 2018: Dual−range migrant
ID28, 2016: Dual−range migrant
ID28, 2017: Dual−range migrant
ID28, 2018: Dual−range migrant
Net Squared Displacement (km)
Day (starting July 1st)

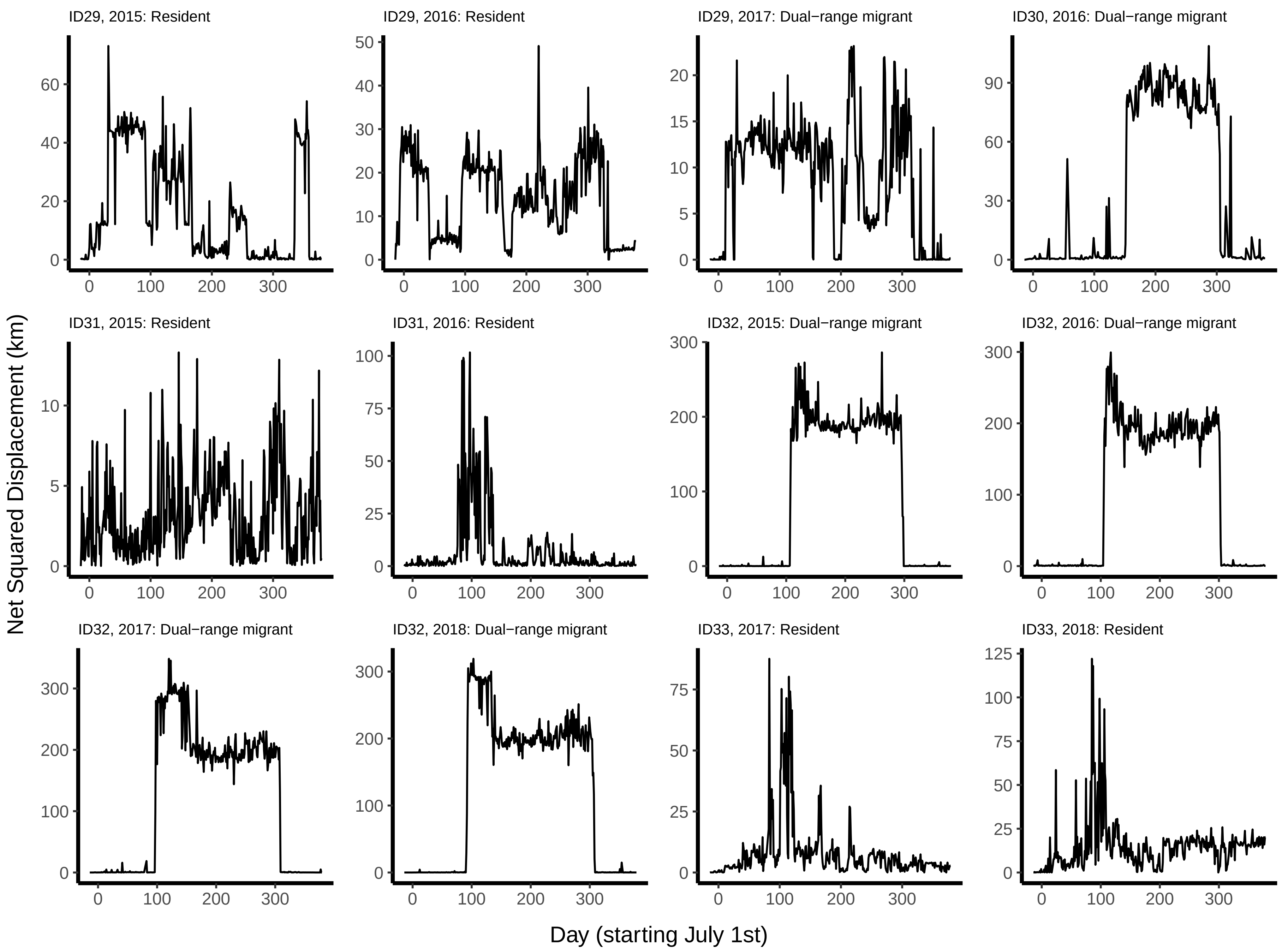
ID29, 2015: Resident
ID29, 2016: Resident
ID29, 2017: Dual−range migrant
ID30, 2016: Dual−range migrant
ID31, 2015: Resident
ID31, 2016: Resident
ID32, 2015: Dual−range migrant
ID32, 2016: Dual−range migrant
ID32, 2017: Dual−range migrant
ID32, 2018: Dual−range migrant
ID33, 2017: Resident
ID33, 2018: Resident
Net Squared Displacement (km)
Day (starting July 1st)

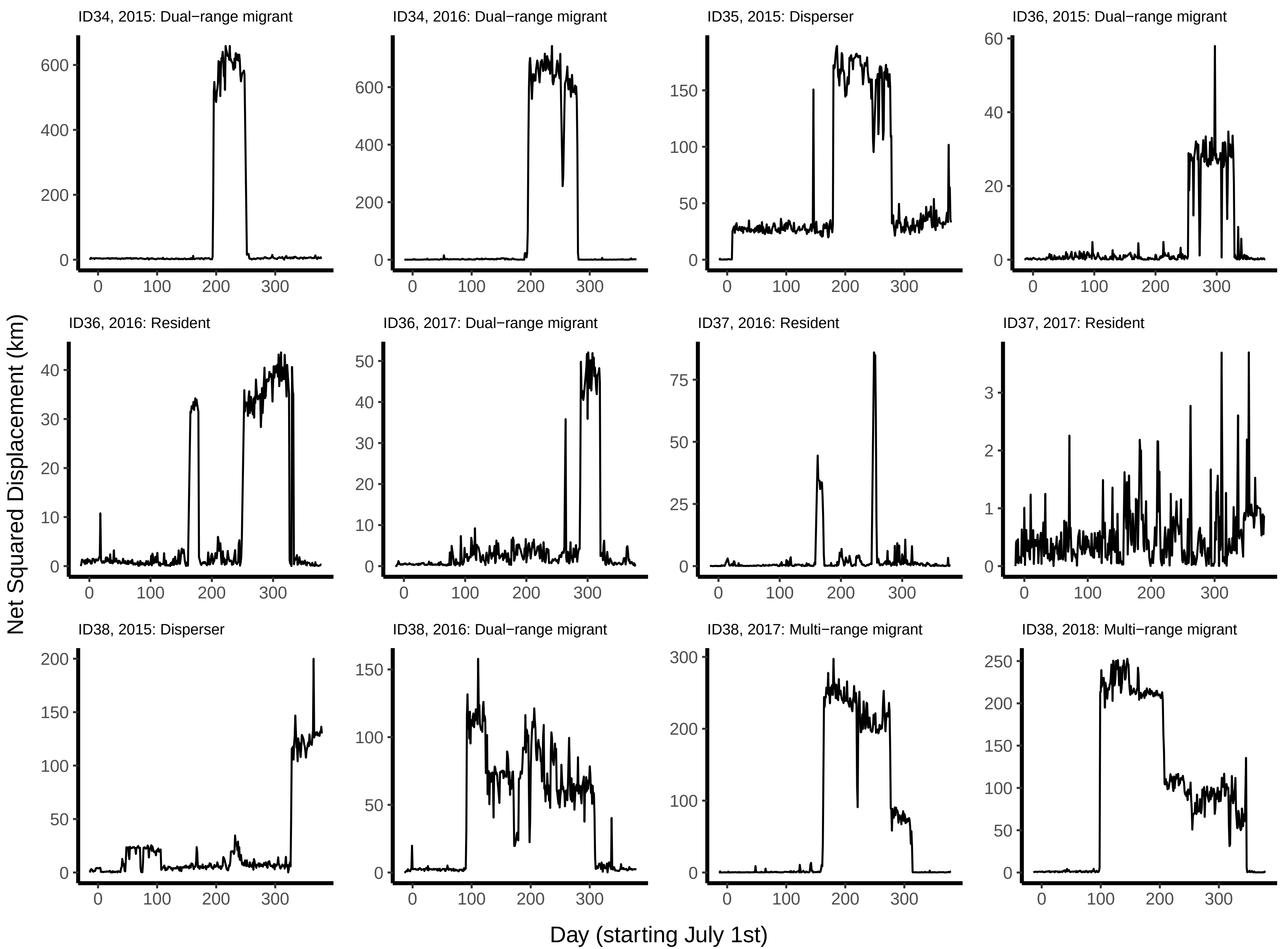

ID34, 2015: Dual−range migrant
ID34, 2016: Dual−range migrant
ID35, 2015: Disperser
ID36, 2015: Dual−range migrant
ID36, 2016: Resident
ID36, 2017: Dual−range migrant
ID37, 2016: Resident
ID37, 2017: Resident
ID38, 2015: Disperser
ID38, 2016: Dual−range migrant
ID38, 2017: Multi−range migrant
ID38, 2018: Multi−range migrant
Net Squared Displacement (km)
Day (starting July 1st)

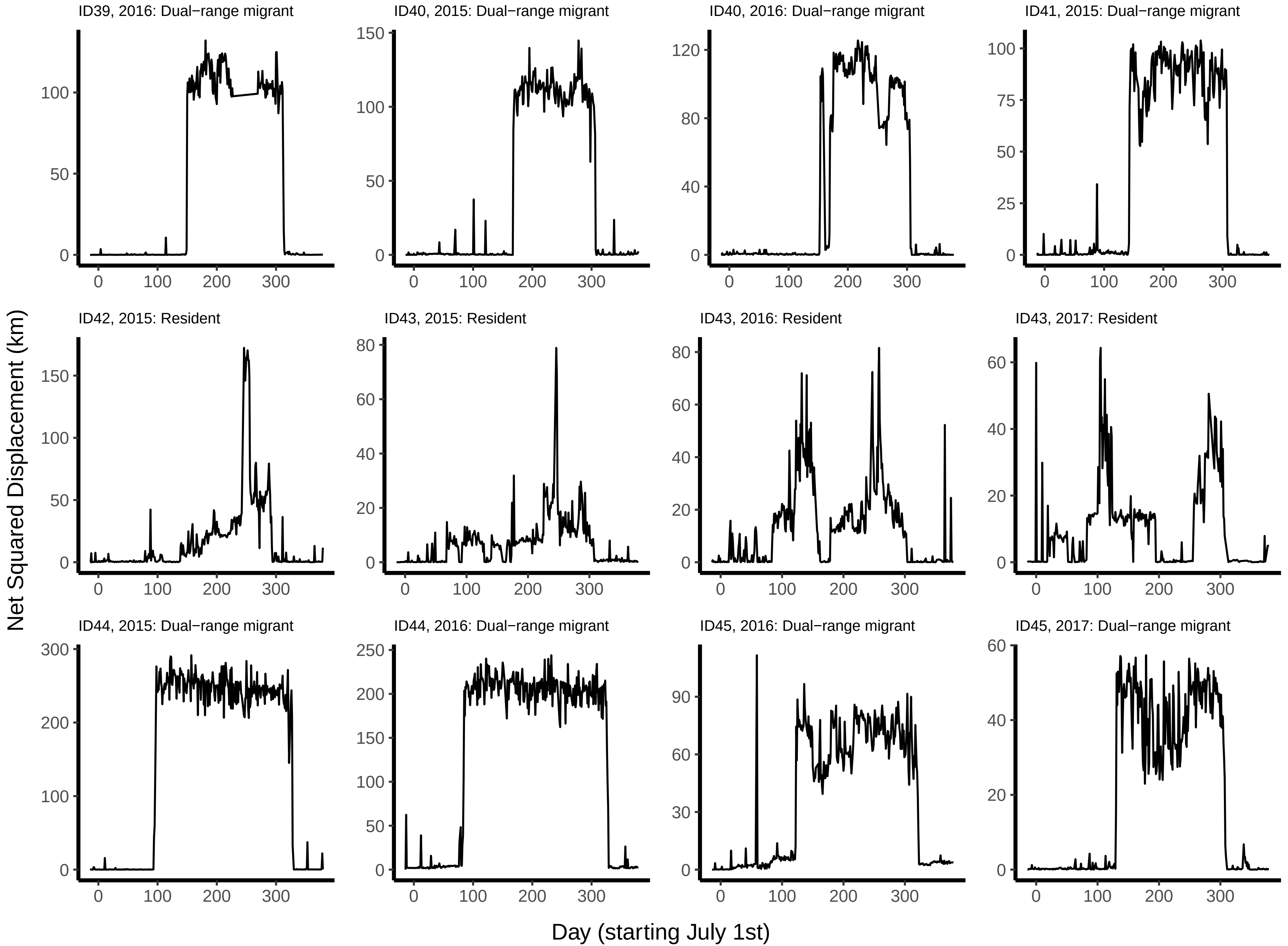

ID39, 2016: Dual−range migrant
ID40, 2015: Dual−range migrant
ID40, 2016: Dual−range migrant
ID41, 2015: Dual−range migrant
ID42, 2015: Resident
ID43, 2015: Resident
ID43, 2016: Resident
ID43, 2017: Resident
ID44, 2015: Dual−range migrant
ID44, 2016: Dual−range migrant
ID45, 2016: Dual−range migrant
ID45, 2017: Dual−range migrant
Net Squared Displacement (km)
Day (starting July 1st)

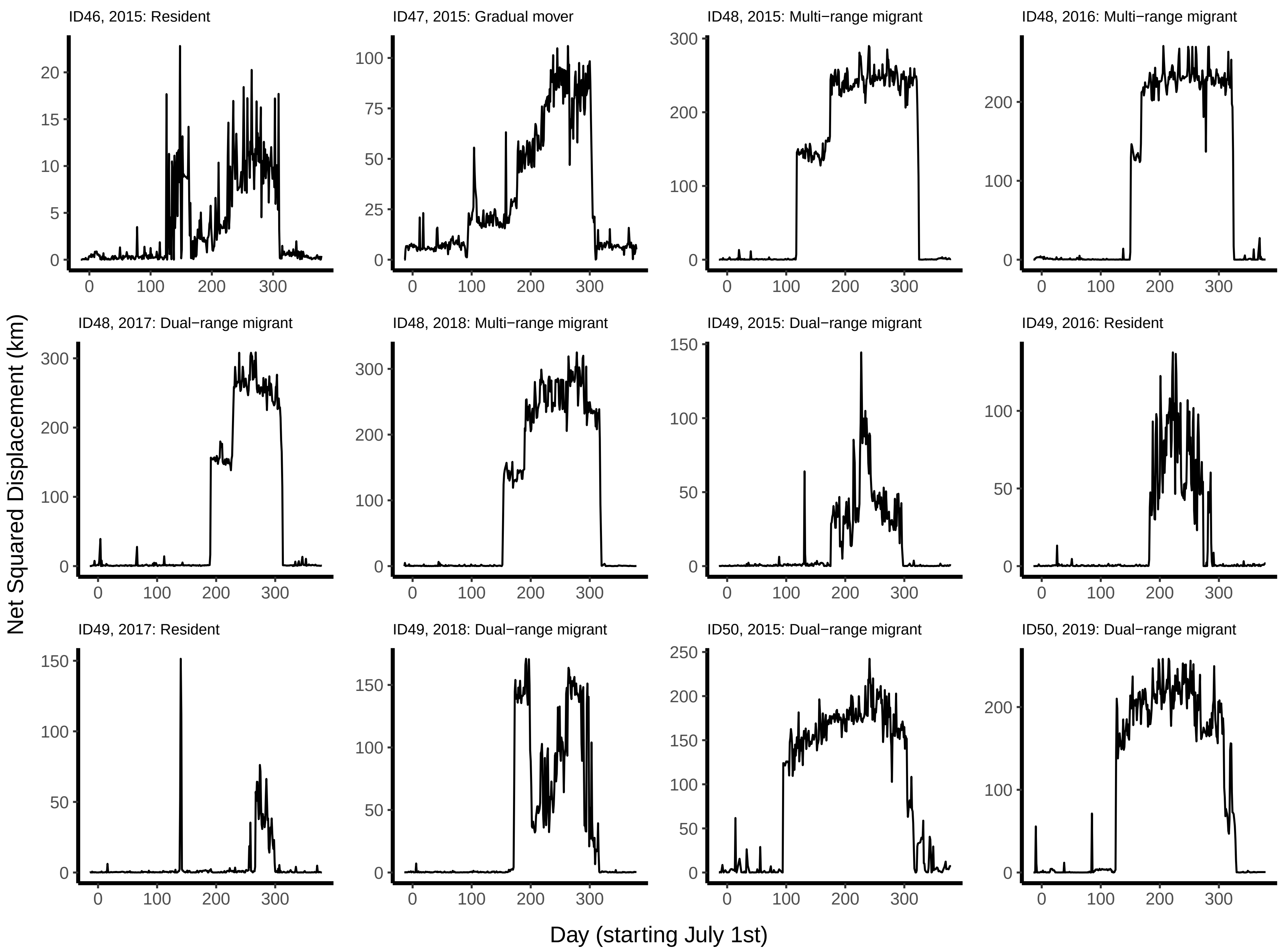
ID46, 2015: Resident
ID47, 2015: Gradual mover
ID48, 2015: Multi−range migrant
ID48, 2016: Multi−range migrant
ID48, 2017: Dual−range migrant
ID48, 2018: Multi−range migrant
ID49, 2015: Dual−range migrant
ID49, 2016: Resident
ID49, 2017: Resident
ID49, 2018: Dual−range migrant
ID50, 2015: Dual−range migrant
ID50, 2019: Dual−range migrant
Net Squared Displacement (km)
Day (starting July 1st)

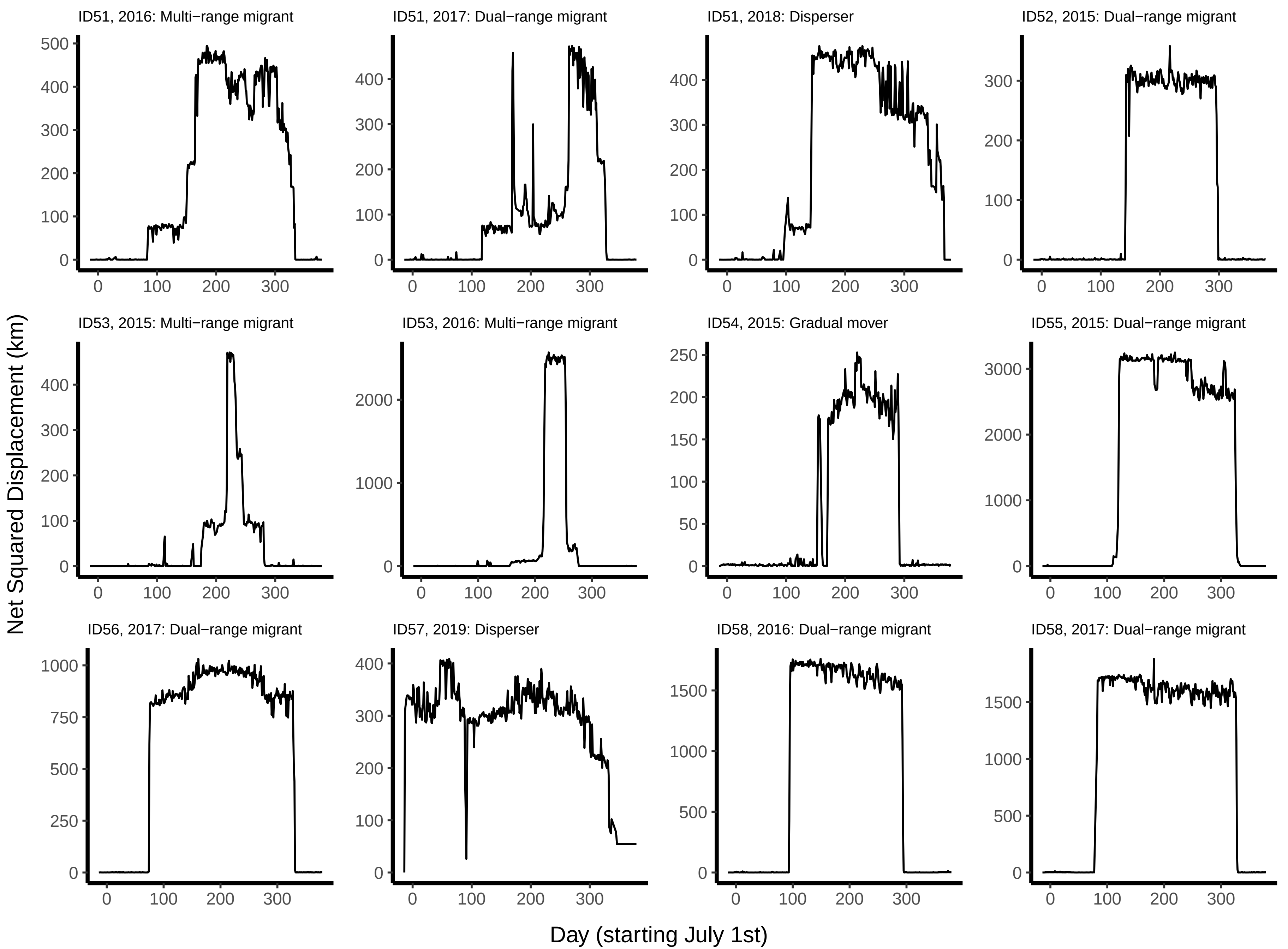

ID51, 2016: Multi−range migrant
ID51, 2017: Dual−range migrant
ID51, 2018: Disperser
ID52, 2015: Dual−range migrant
ID53, 2015: Multi−range migrant
ID53, 2016: Multi−range migrant
ID54, 2015: Gradual mover
ID55, 2015: Dual−range migrant
ID56, 2017: Dual−range migrant
ID57, 2019: Disperser
ID58, 2016: Dual−range migrant
ID58, 2017: Dual−range migrant
Net Squared Displacement (km)
Day (starting July 1st)

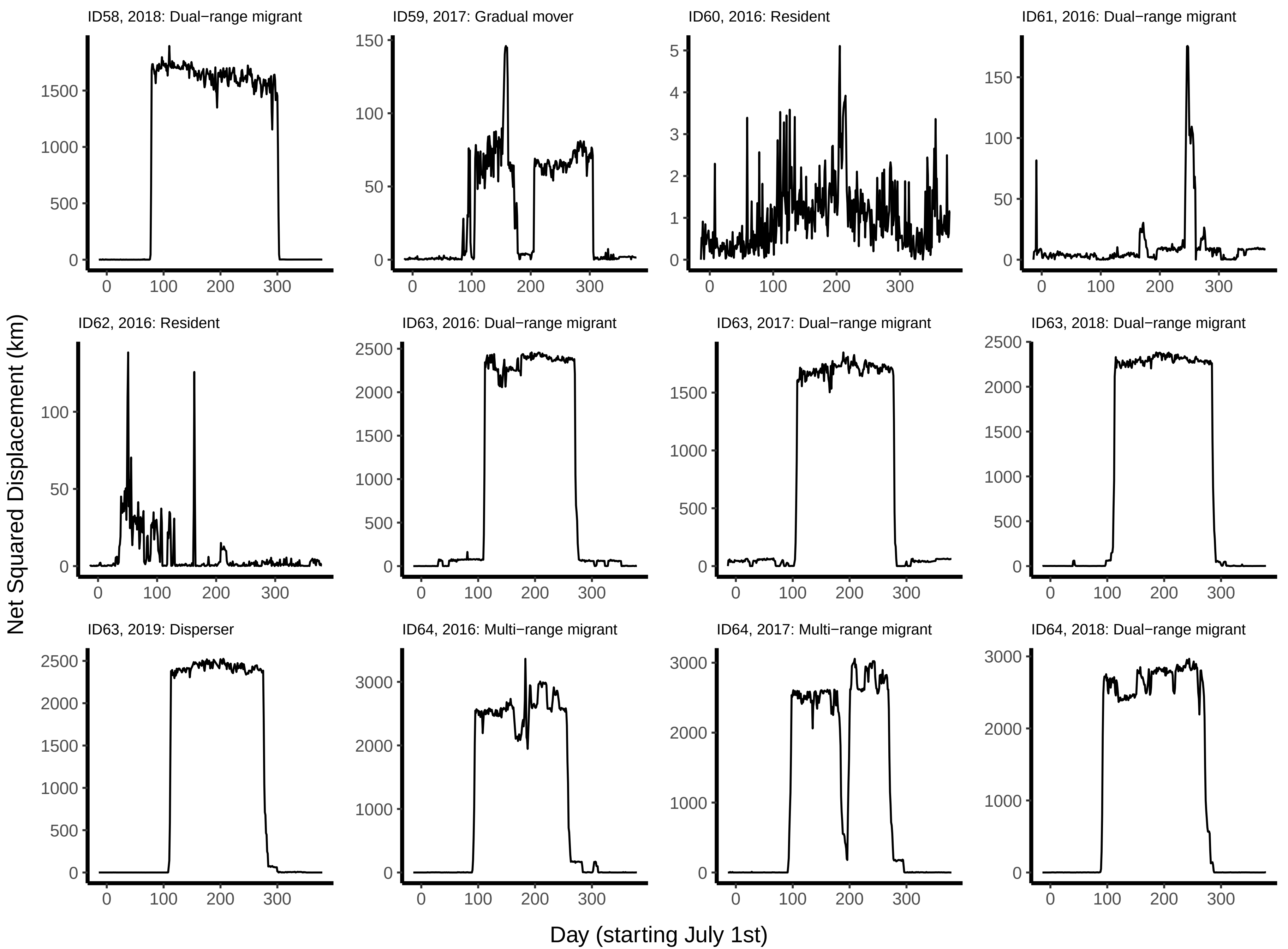

ID58, 2018: Dual−range migrant
ID59, 2017: Gradual mover
ID60, 2016: Resident
ID61, 2016: Dual−range migrant
ID62, 2016: Resident
ID63, 2016: Dual−range migrant
ID63, 2017: Dual−range migrant
ID63, 2018: Dual−range migrant
ID63, 2019: Disperser
ID64, 2016: Multi−range migrant
ID64, 2017: Multi−range migrant
ID64, 2018: Dual−range migrant
Net Squared Displacement (km)
Day (starting July 1st)

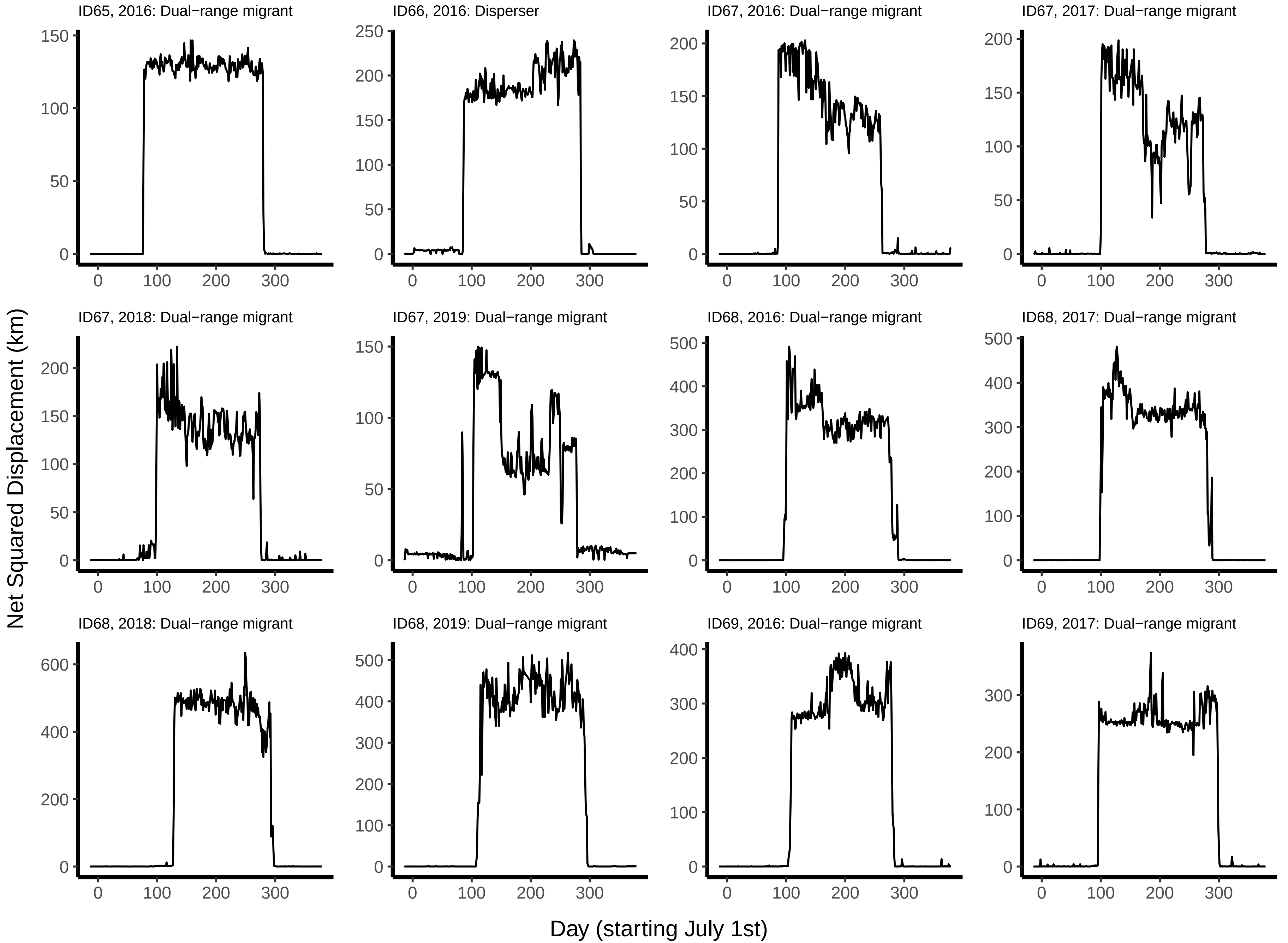
ID65, 2016: Dual−range migrant
ID66, 2016: Disperser
ID67, 2016: Dual−range migrant
ID67, 2017: Dual−range migrant
ID67, 2018: Dual−range migrant
ID67, 2019: Dual−range migrant
ID68, 2016: Dual−range migrant
ID68, 2017: Dual−range migrant
ID68, 2018: Dual−range migrant
ID68, 2019: Dual−range migrant
ID69, 2016: Dual−range migrant
ID69, 2017: Dual−range migrant
Net Squared Displacement (km)
Day (starting July 1st)

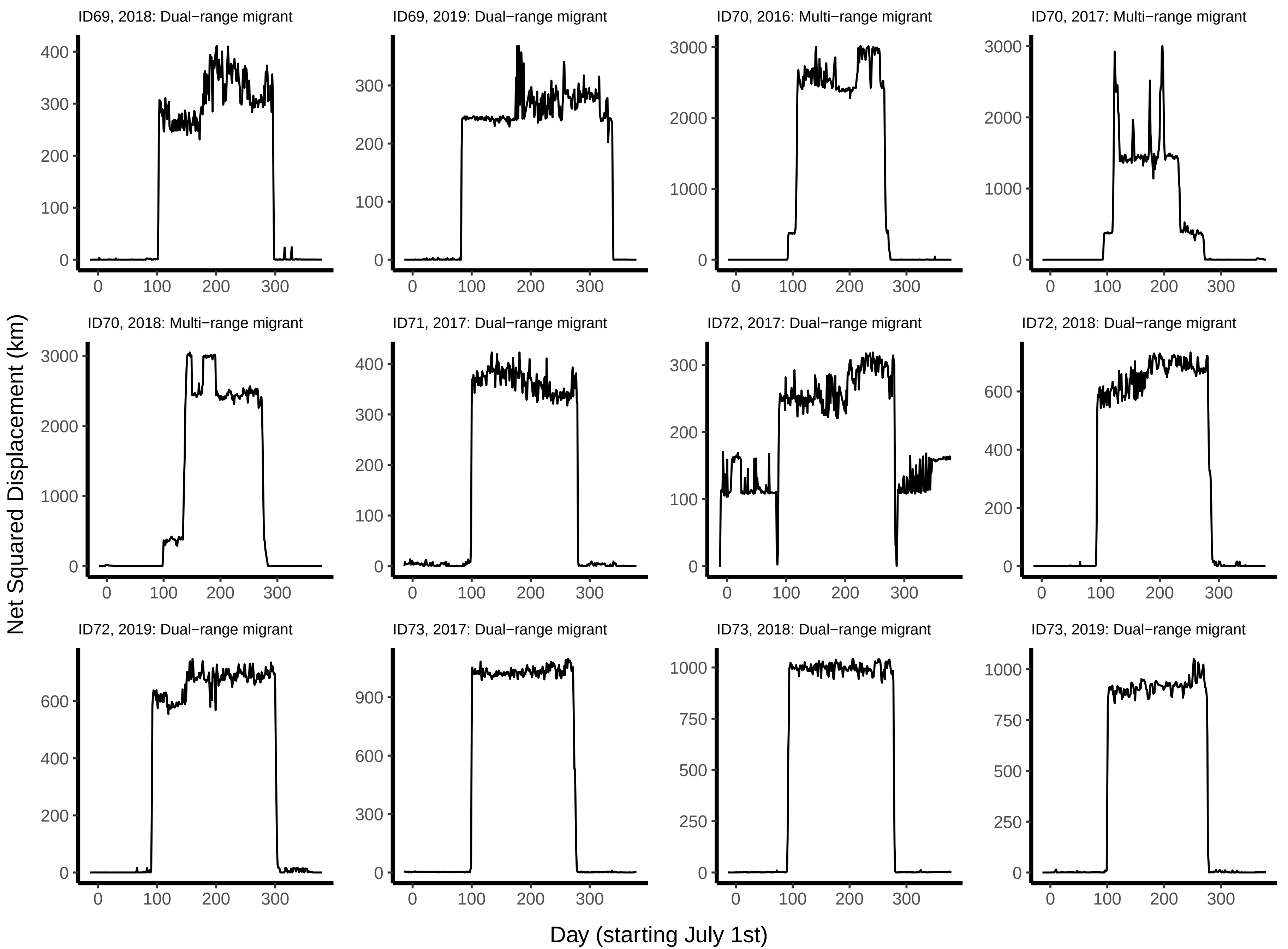
ID69, 2018: Dual−range migrant
ID69, 2019: Dual−range migrant
ID70, 2016: Multi−range migrant
ID70, 2017: Multi−range migrant
ID70, 2018: Multi−range migrant
ID71, 2017: Dual−range migrant
ID72, 2017: Dual−range migrant
ID72, 2018: Dual−range migrant
ID72, 2019: Dual−range migrant
ID73, 2017: Dual−range migrant
ID73, 2018: Dual−range migrant
ID73, 2019: Dual−range migrant
Net Squared Displacement (km)
Day (starting July 1st)

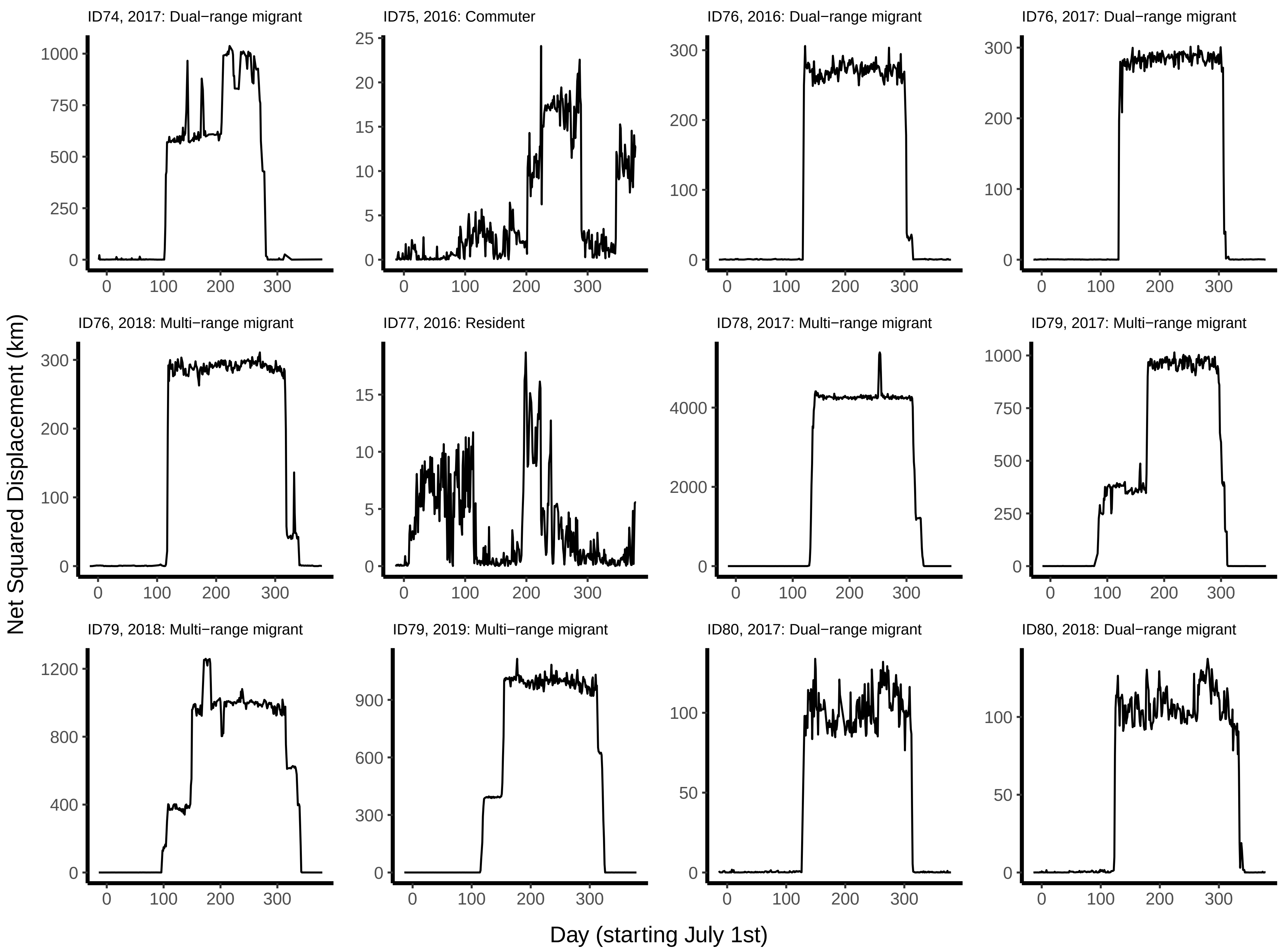

ID74, 2017: Dual−range migrant
ID75, 2016: Commuter
ID76, 2016: Dual−range migrant
ID76, 2017: Dual−range migrant
ID76, 2018: Multi−range migrant
ID77, 2016: Resident
ID78, 2017: Multi−range migrant
ID79, 2017: Multi−range migrant
ID79, 2018: Multi−range migrant
ID79, 2019: Multi−range migrant
ID80, 2017: Dual−range migrant
ID80, 2018: Dual−range migrant
Net Squared Displacement (km)
Day (starting July 1st)

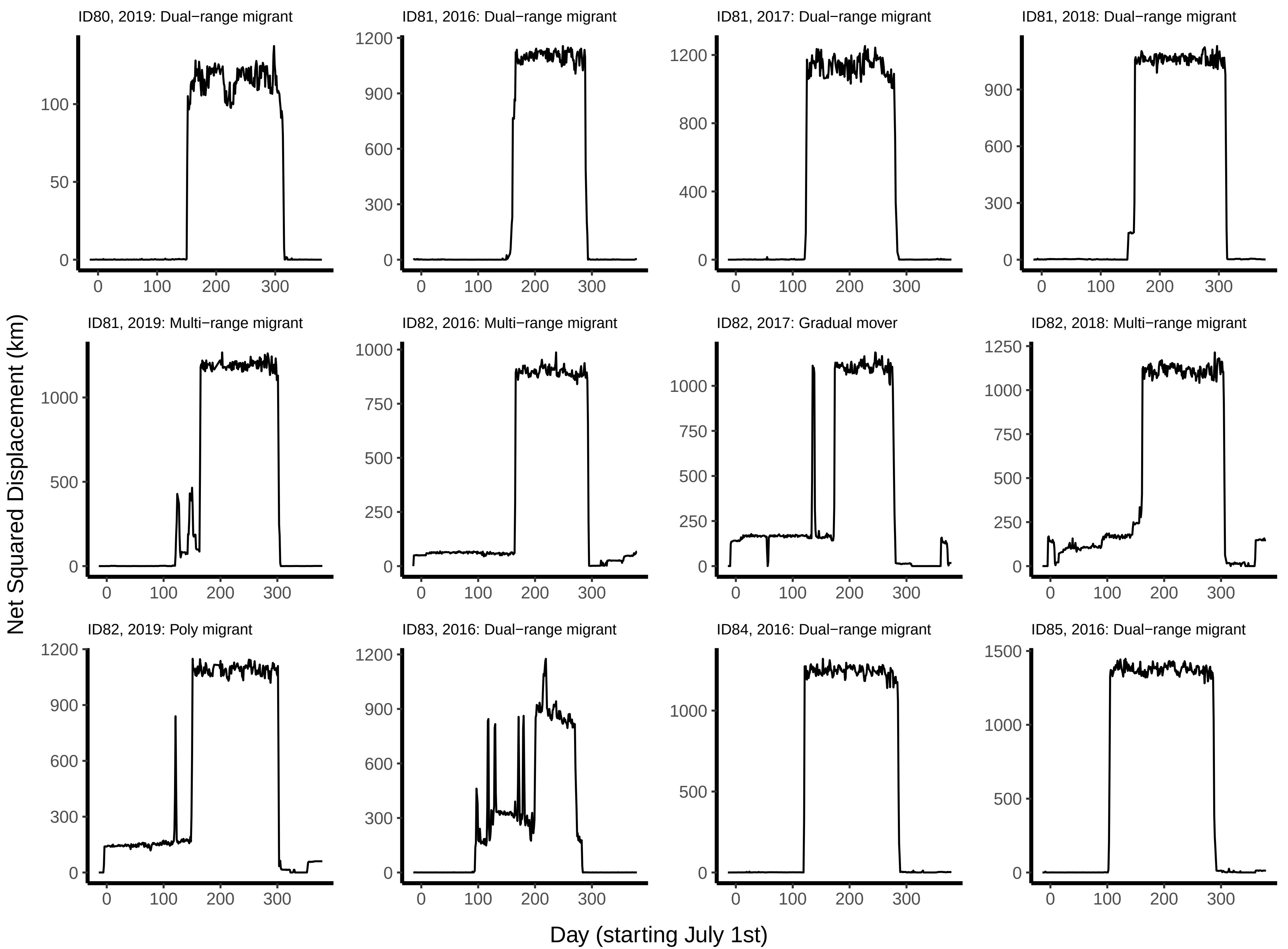

ID80, 2019: Dual−range migrant
ID81, 2016: Dual−range migrant
ID81, 2017: Dual−range migrant
ID81, 2018: Dual−range migrant
ID81, 2019: Multi−range migrant
ID82, 2016: Multi−range migrant
ID82, 2017: Gradual mover
ID82, 2018: Multi−range migrant
ID82, 2019: Poly migrant
ID83, 2016: Dual−range migrant
ID84, 2016: Dual−range migrant
ID85, 2016: Dual−range migrant
Net Squared Displacement (km)
Day (starting July 1st)

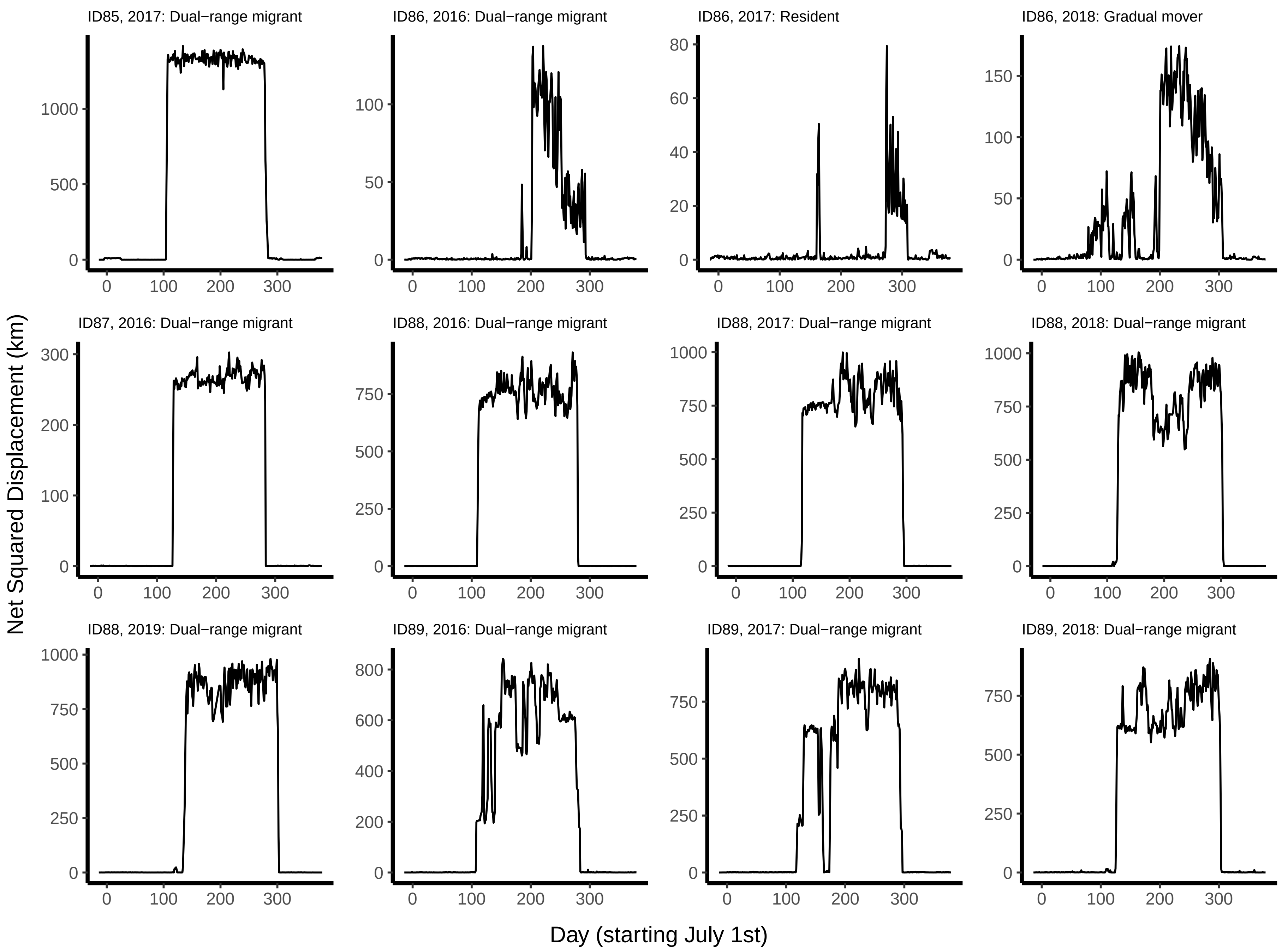
ID85, 2017: Dual−range migrant
ID86, 2016: Dual−range migrant
ID86, 2017: Resident
ID86, 2018: Gradual mover
ID87, 2016: Dual−range migrant
ID88, 2016: Dual−range migrant
ID88, 2017: Dual−range migrant
ID88, 2018: Dual−range migrant
ID88, 2019: Dual−range migrant
ID89, 2016: Dual−range migrant
ID89, 2017: Dual−range migrant
ID89, 2018: Dual−range migrant
Net Squared Displacement (km)
Day (starting July 1st)

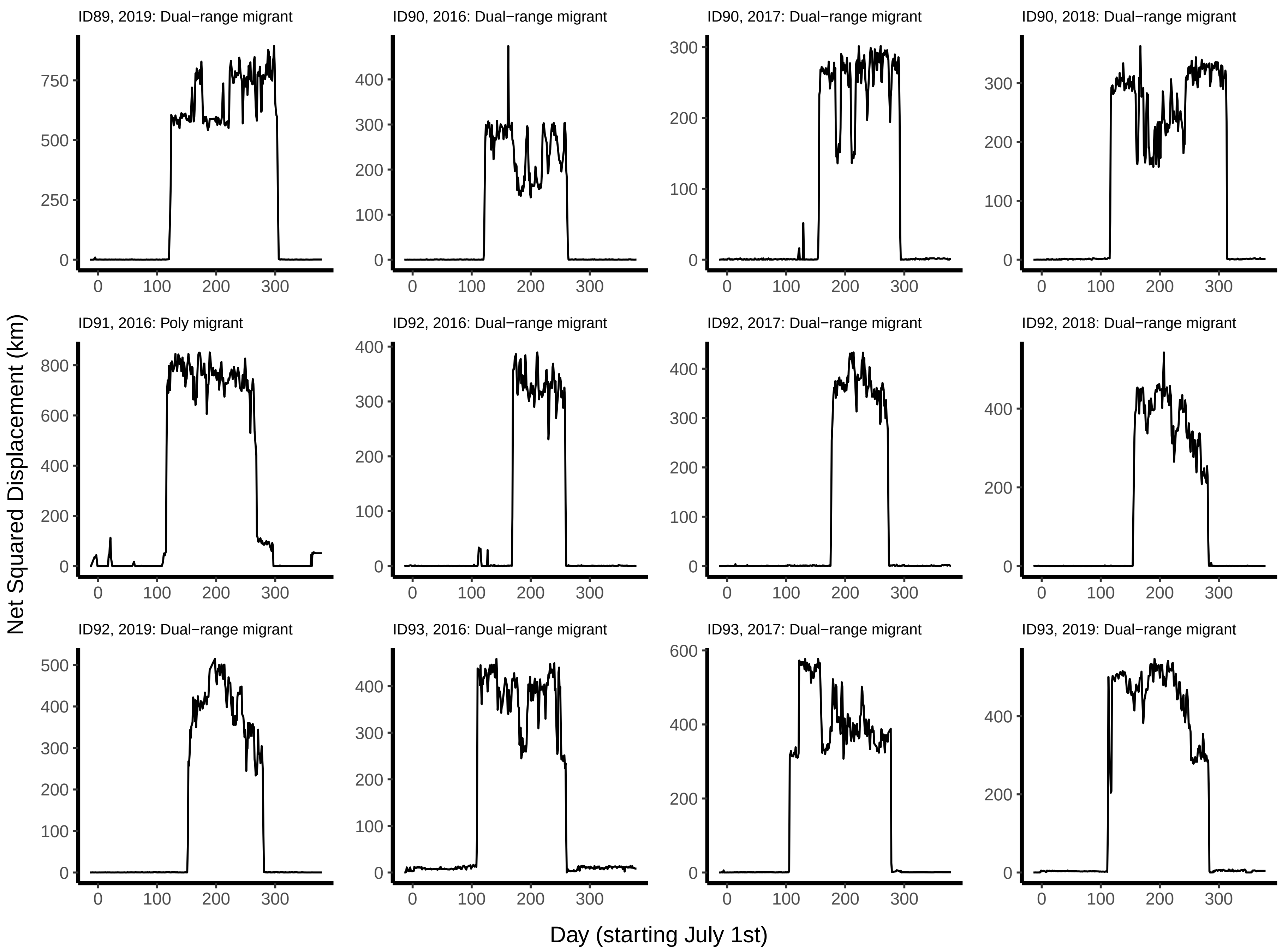
ID89, 2019: Dual−range migrant
ID90, 2016: Dual−range migrant
ID90, 2017: Dual−range migrant
ID90, 2018: Dual−range migrant
ID91, 2016: Poly migrant
ID92, 2016: Dual−range migrant
ID92, 2017: Dual−range migrant
ID92, 2018: Dual−range migrant
ID92, 2019: Dual−range migrant
ID93, 2016: Dual−range migrant
ID93, 2017: Dual−range migrant
ID93, 2019: Dual−range migrant
Net Squared Displacement (km)
Day (starting July 1st)

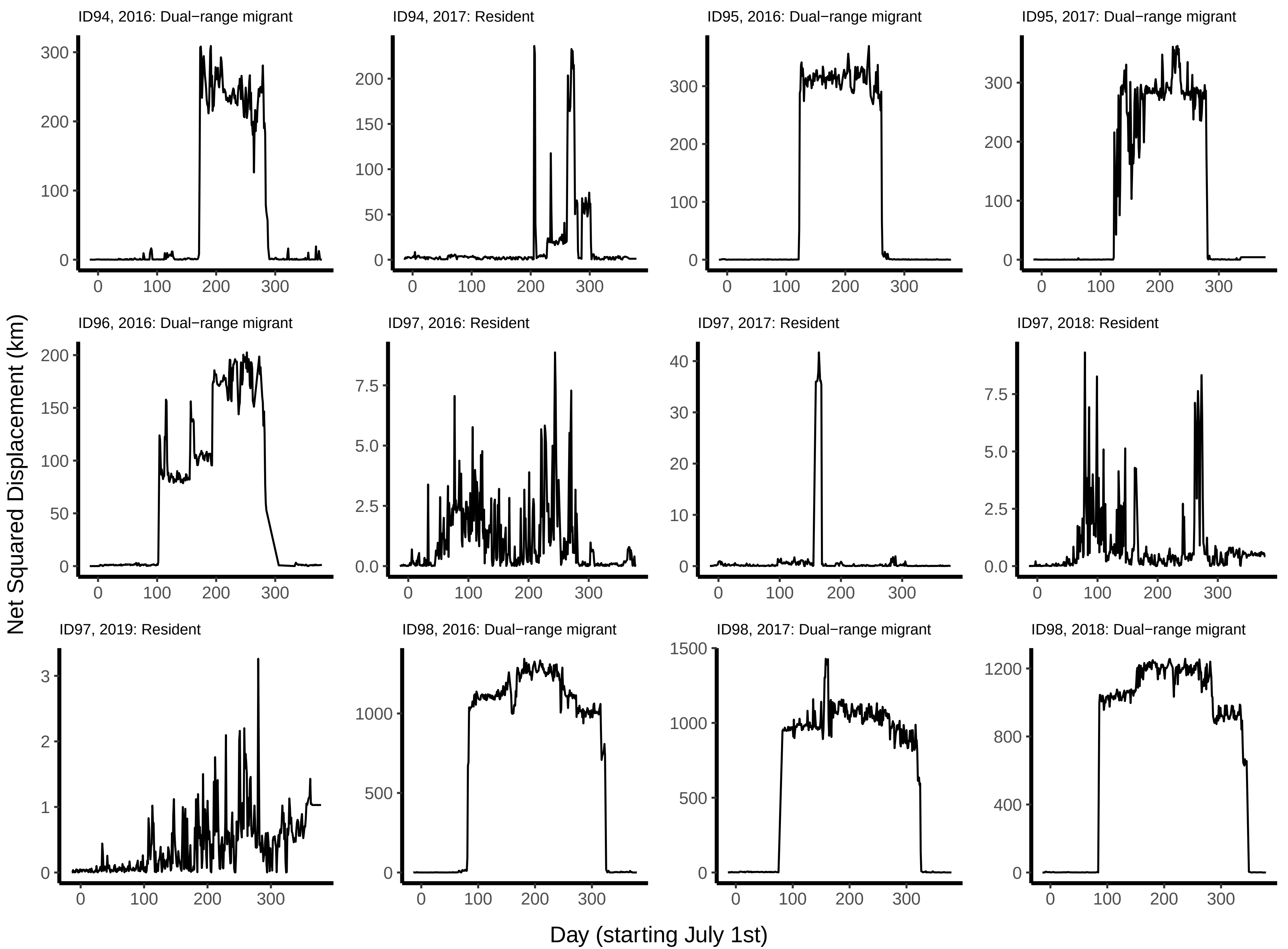

ID94, 2016: Dual−range migrant
ID94, 2017: Resident
ID95, 2016: Dual−range migrant
ID95, 2017: Dual−range migrant
ID96, 2016: Dual−range migrant
ID97, 2016: Resident
ID97, 2017: Resident
ID97, 2018: Resident
ID97, 2019: Resident
ID98, 2016: Dual−range migrant
ID98, 2017: Dual−range migrant
ID98, 2018: Dual−range migrant
Net Squared Displacement (km)
Day (starting July 1st)

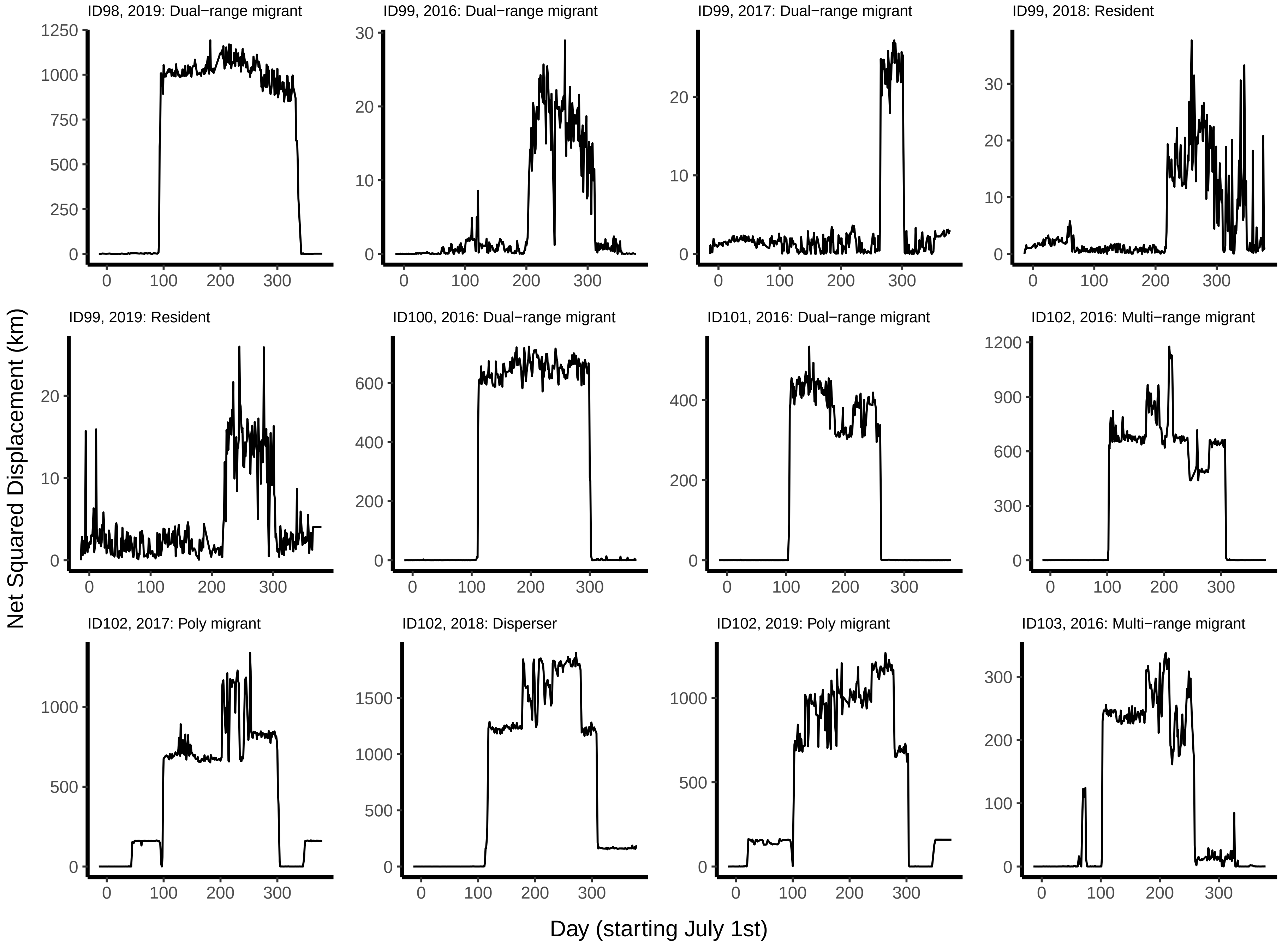
ID98, 2019: Dual−range migrant
ID99, 2016: Dual−range migrant
ID99, 2017: Dual−range migrant
ID99, 2018: Resident
ID99, 2019: Resident
ID100, 2016: Dual−range migrant
ID101, 2016: Dual−range migrant
ID102, 2016: Multi−range migrant
ID102, 2017: Poly migrant
ID102, 2018: Disperser
ID102, 2019: Poly migrant
ID103, 2016: Multi−range migrant
Net Squared Displacement (km)
Day (starting July 1st)

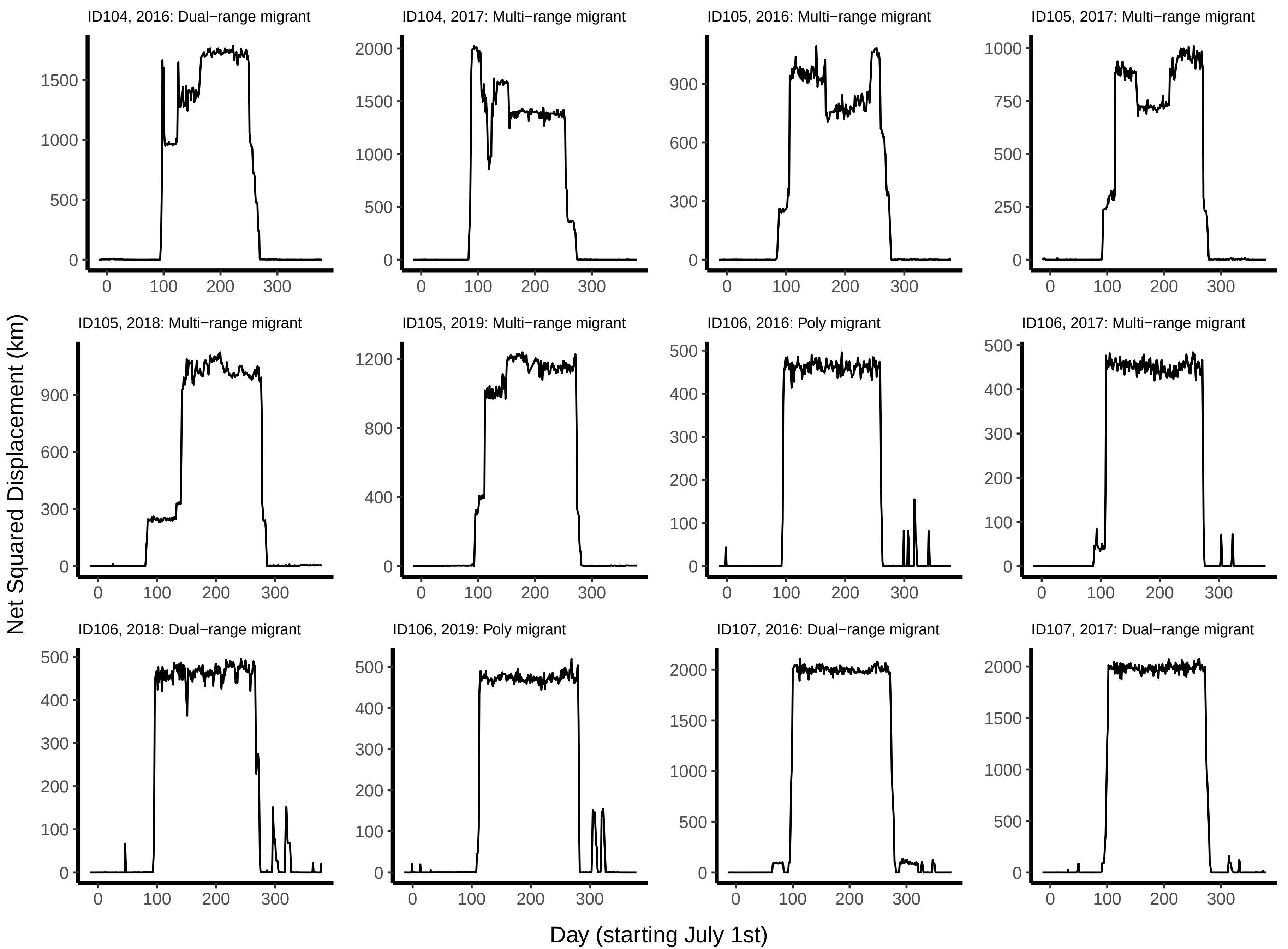

ID104, 2016: Dual−range migrant
ID104, 2017: Multi−range migrant
ID105, 2016: Multi−range migrant
ID105, 2017: Multi−range migrant
ID105, 2018: Multi−range migrant
ID105, 2019: Multi−range migrant
ID106, 2016: Poly migrant
ID106, 2017: Multi−range migrant
ID106, 2018: Dual−range migrant
ID106, 2019: Poly migrant
ID107, 2016: Dual−range migrant
ID107, 2017: Dual−range migrant
Net Squared Displacement (km)
Day (starting July 1st)

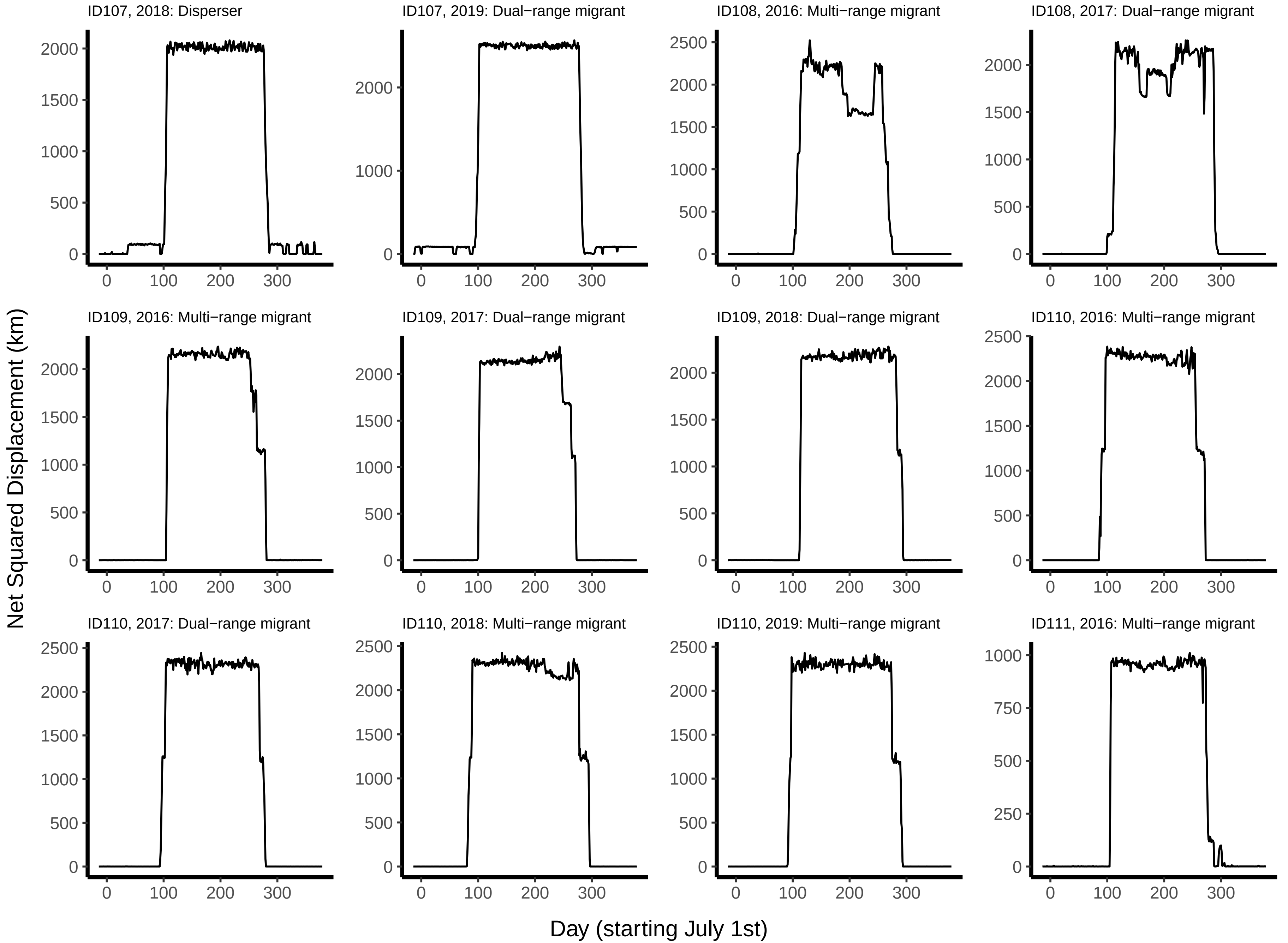

ID107, 2018: Disperser
ID107, 2019: Dual−range migrant
ID108, 2016: Multi−range migrant
ID108, 2017: Dual−range migrant
ID109, 2016: Multi−range migrant
ID109, 2017: Dual−range migrant
ID109, 2018: Dual−range migrant
ID110, 2016: Multi−range migrant
ID110, 2017: Dual−range migrant
ID110, 2018: Multi−range migrant
ID110, 2019: Multi−range migrant
ID111, 2016: Multi−range migrant
Net Squared Displacement (km)
Day (starting July 1st)

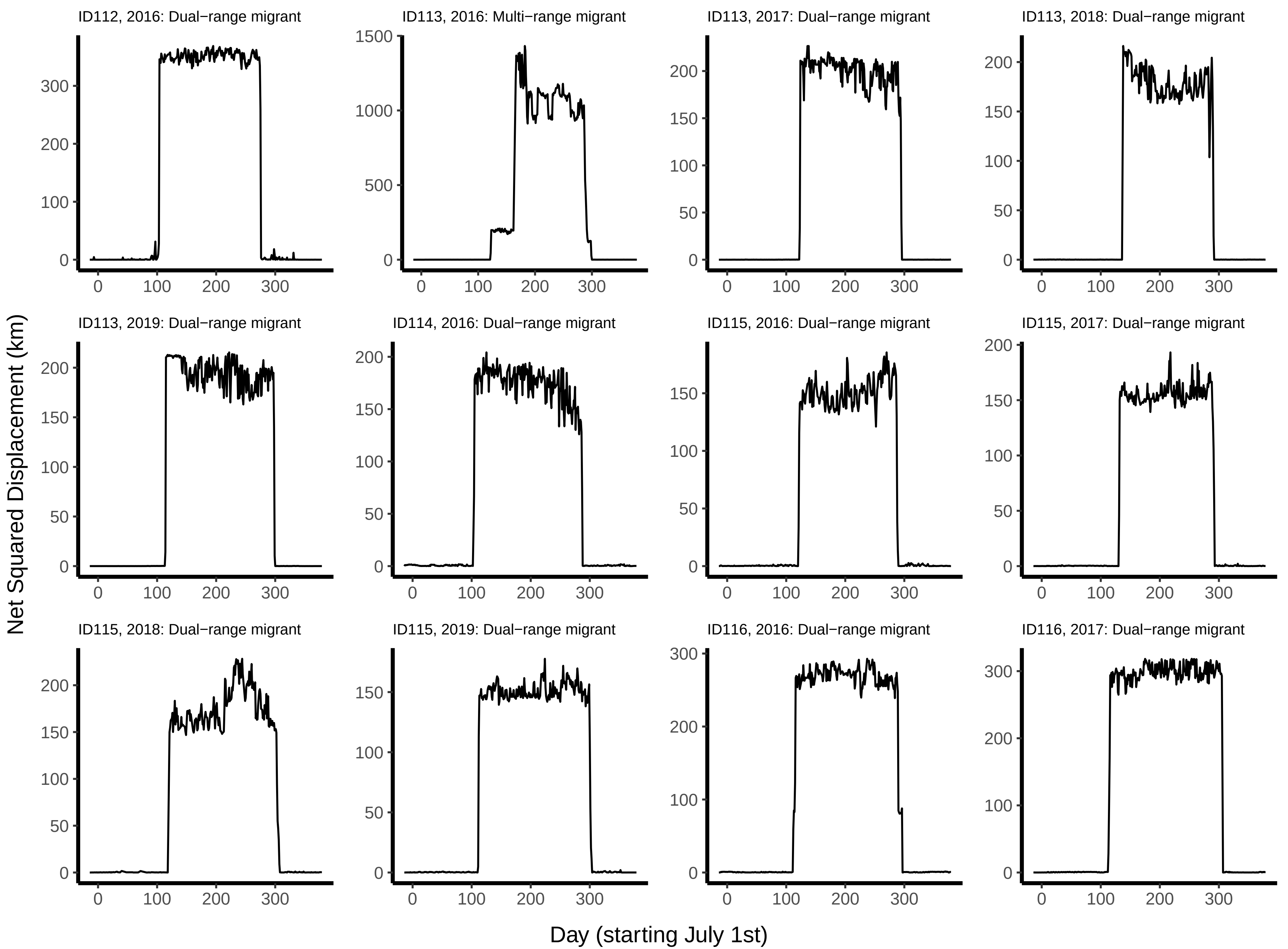

ID112, 2016: Dual−range migrant
ID113, 2016: Multi−range migrant
ID113, 2017: Dual−range migrant
ID113, 2018: Dual−range migrant
ID113, 2019: Dual−range migrant
ID114, 2016: Dual−range migrant
ID115, 2016: Dual−range migrant
ID115, 2017: Dual−range migrant
ID115, 2018: Dual−range migrant
ID115, 2019: Dual−range migrant
ID116, 2016: Dual−range migrant
ID116, 2017: Dual−range migrant
Net Squared Displacement (km)
Day (starting July 1st)

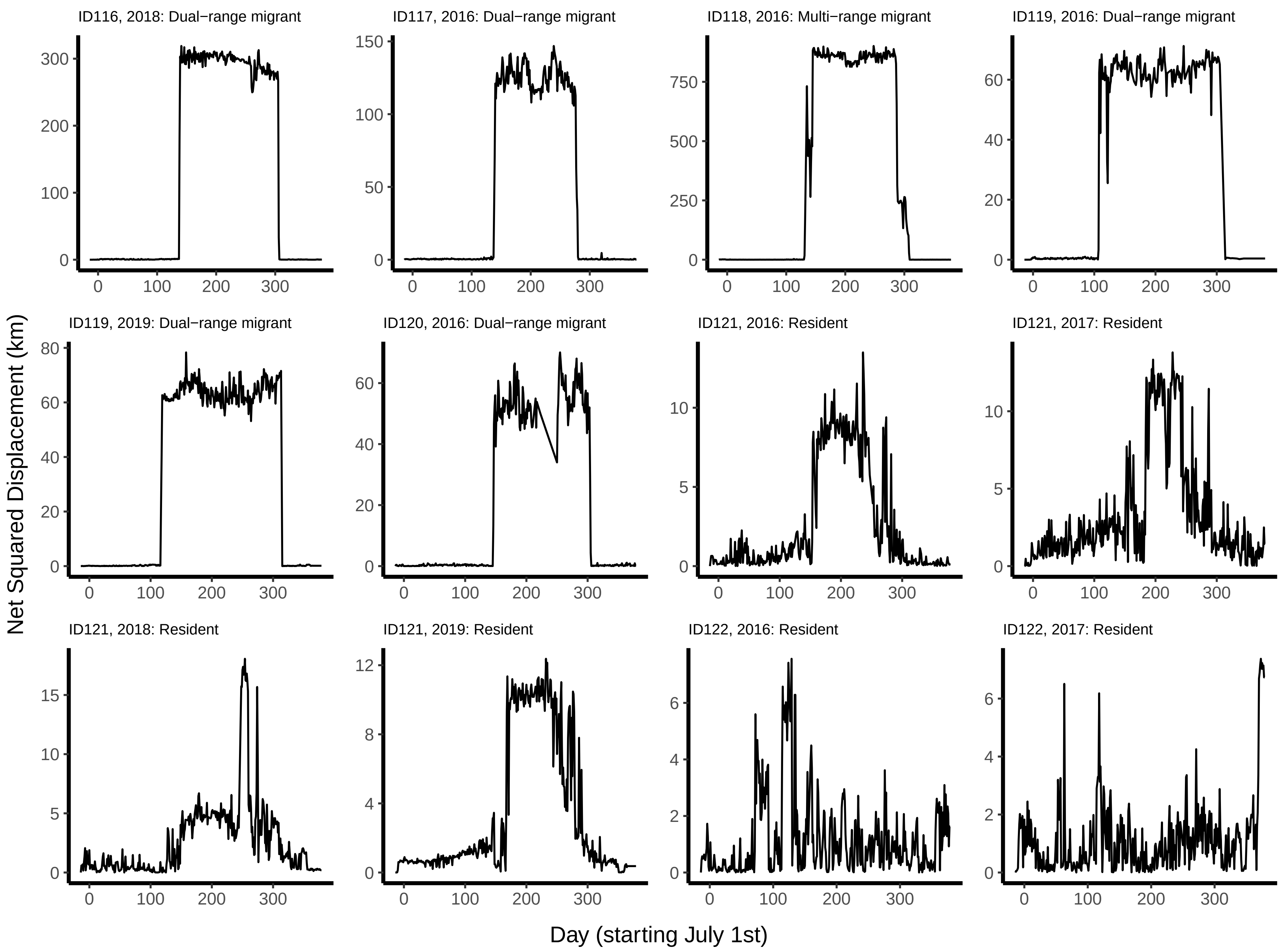

ID116, 2018: Dual−range migrant
ID117, 2016: Dual−range migrant
ID118, 2016: Multi−range migrant
ID119, 2016: Dual−range migrant
ID119, 2019: Dual−range migrant
ID120, 2016: Dual−range migrant
ID121, 2016: Resident
ID121, 2017: Resident
ID121, 2018: Resident
ID121, 2019: Resident
ID122, 2016: Resident
ID122, 2017: Resident
Net Squared Displacement (km)
Day (starting July 1st)
0
100
200
300

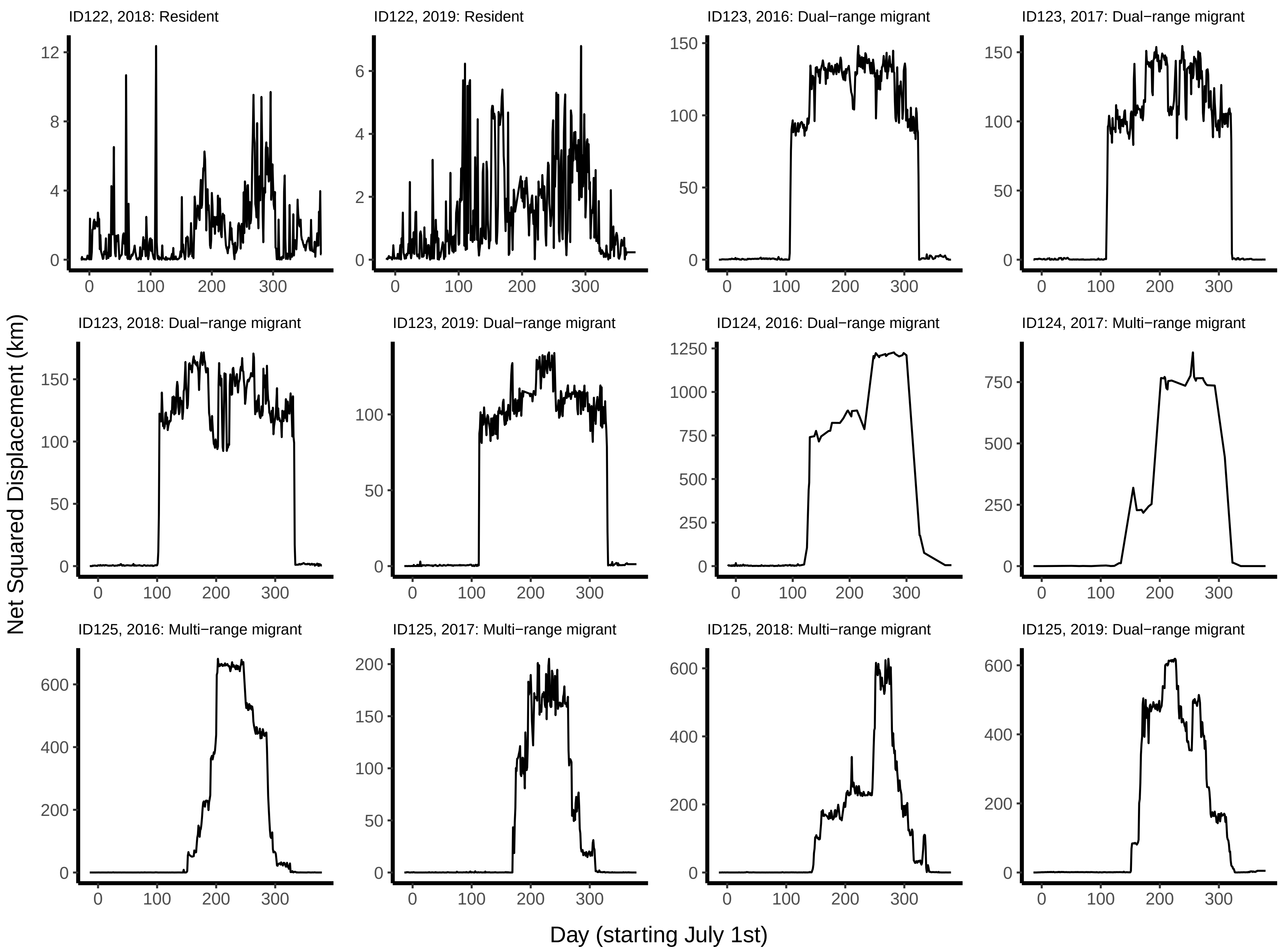
ID122, 2018: Resident
ID122, 2019: Resident
ID123, 2016: Dual−range migrant
ID123, 2017: Dual−range migrant
ID123, 2018: Dual−range migrant
ID123, 2019: Dual−range migrant
ID124, 2016: Dual−range migrant
ID124, 2017: Multi−range migrant
ID125, 2016: Multi−range migrant
ID125, 2017: Multi−range migrant
ID125, 2018: Multi−range migrant
ID125, 2019: Dual−range migrant
Net Squared Displacement (km)
Day (starting July 1st)

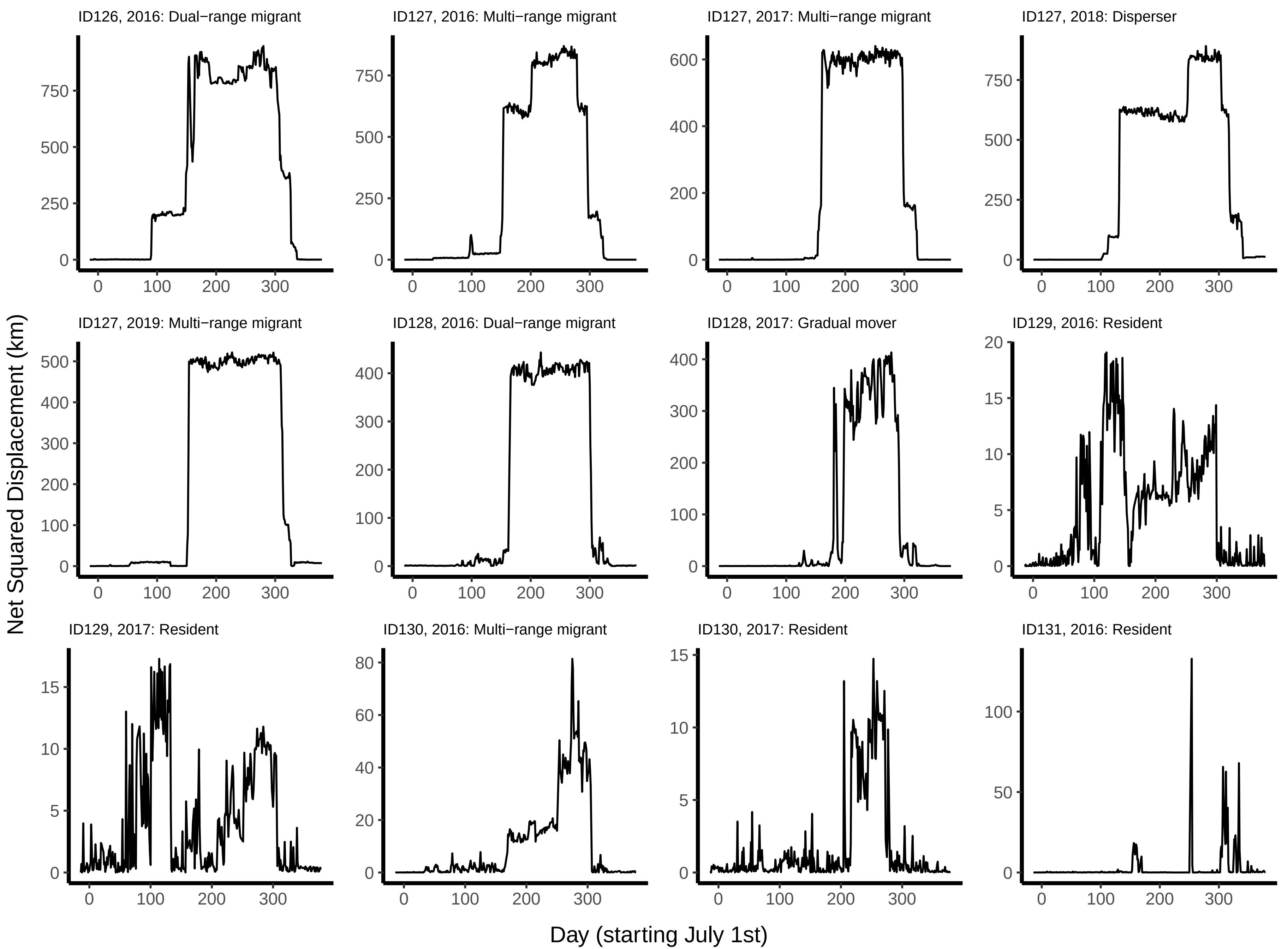

ID126, 2016: Dual−range migrant
ID127, 2016: Multi−range migrant
ID127, 2017: Multi−range migrant
ID127, 2018: Disperser
ID127, 2019: Multi−range migrant
ID128, 2016: Dual−range migrant
ID128, 2017: Gradual mover
ID129, 2016: Resident
ID129, 2017: Resident
ID130, 2016: Multi−range migrant
ID130, 2017: Resident
ID131, 2016: Resident
Net Squared Displacement (km)
Day (starting July 1st)

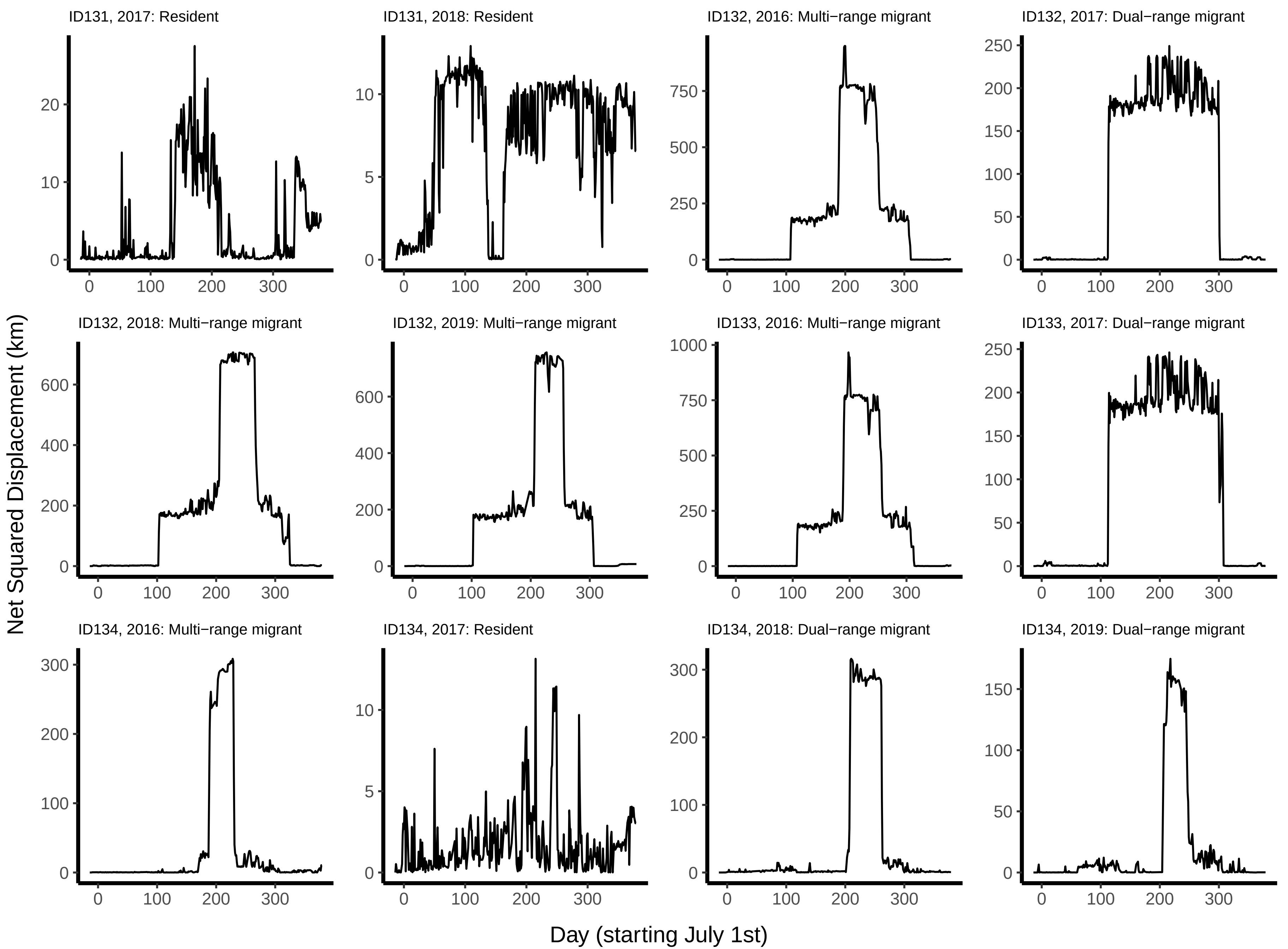

ID131, 2017: Resident
ID131, 2018: Resident
ID132, 2016: Multi−range migrant
ID132, 2017: Dual−range migrant
ID132, 2018: Multi−range migrant
ID132, 2019: Multi−range migrant
ID133, 2016: Multi−range migrant
ID133, 2017: Dual−range migrant
ID134, 2016: Multi−range migrant
ID134, 2017: Resident
ID134, 2018: Dual−range migrant
ID134, 2019: Dual−range migrant
Net Squared Displacement (km)
Day (starting July 1st)

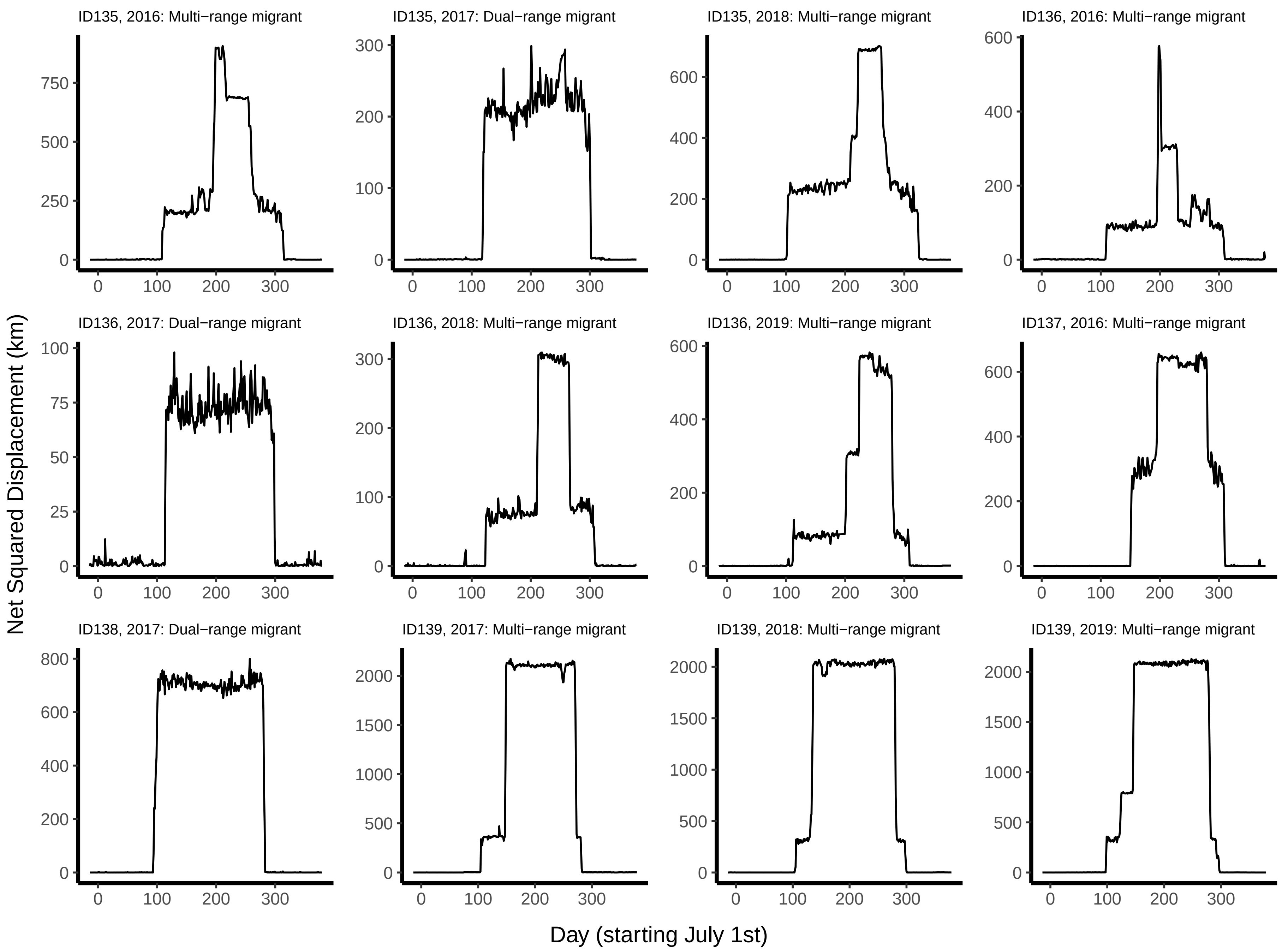
ID135, 2016: Multi−range migrant
ID135, 2017: Dual−range migrant
ID135, 2018: Multi−range migrant
ID136, 2016: Multi−range migrant
ID136, 2017: Dual−range migrant
ID136, 2018: Multi−range migrant
ID136, 2019: Multi−range migrant
ID137, 2016: Multi−range migrant
ID138, 2017: Dual−range migrant
ID139, 2017: Multi−range migrant
ID139, 2018: Multi−range migrant
ID139, 2019: Multi−range migrant
Net Squared Displacement (km)
Day (starting July 1st)

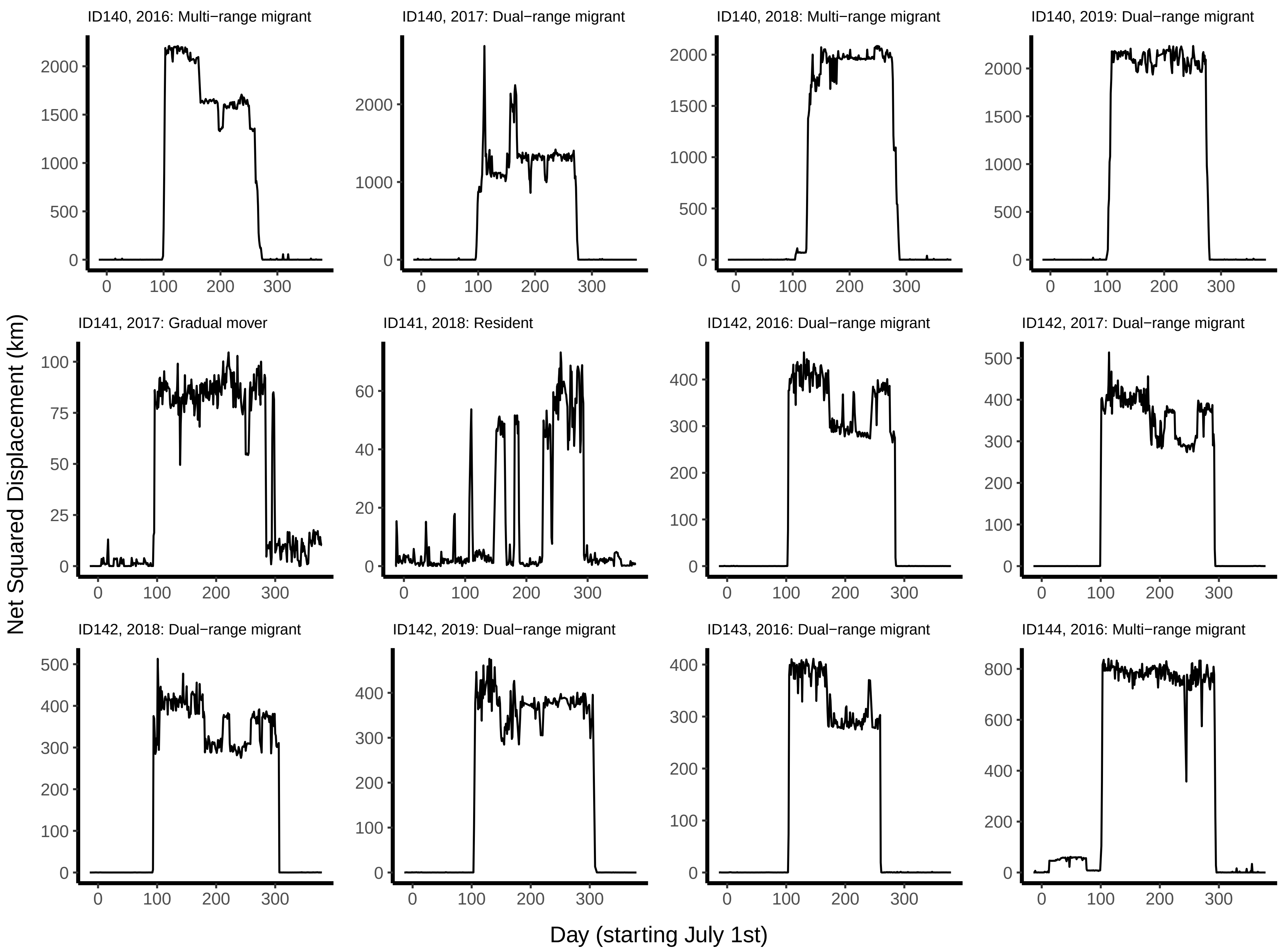

ID140, 2016: Multi−range migrant
ID140, 2017: Dual−range migrant
ID140, 2018: Multi−range migrant
ID140, 2019: Dual−range migrant
ID141, 2017: Gradual mover
ID141, 2018: Resident
ID142, 2016: Dual−range migrant
ID142, 2017: Dual−range migrant
ID142, 2018: Dual−range migrant
ID142, 2019: Dual−range migrant
ID143, 2016: Dual−range migrant
ID144, 2016: Multi−range migrant
Net Squared Displacement (km)
Day (starting July 1st)

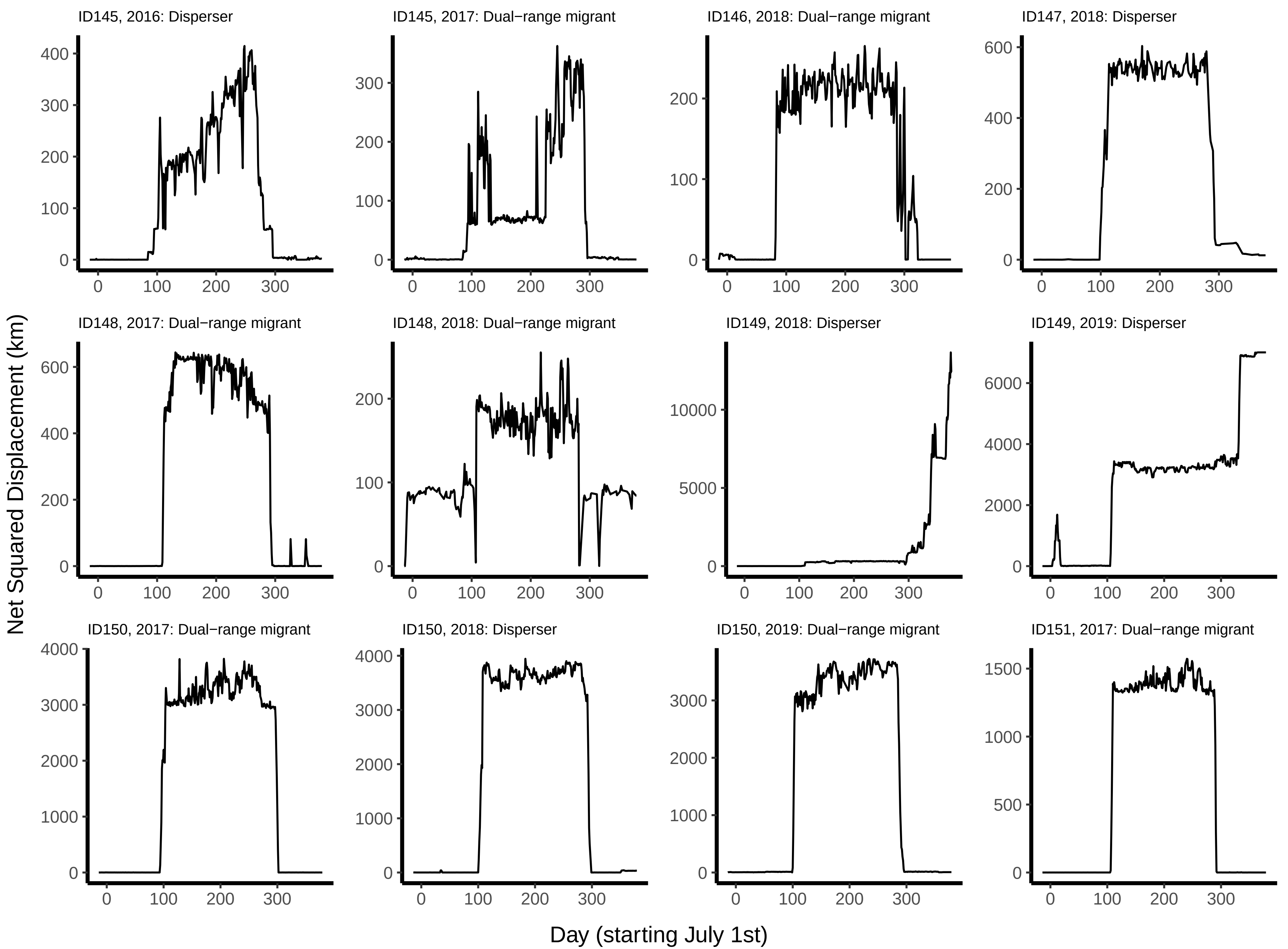

ID145, 2016: Disperser
ID145, 2017: Dual−range migrant
ID146, 2018: Dual−range migrant
ID147, 2018: Disperser
ID148, 2017: Dual−range migrant
ID148, 2018: Dual−range migrant
ID149, 2018: Disperser
ID149, 2019: Disperser
ID150, 2017: Dual−range migrant
ID150, 2018: Disperser
ID150, 2019: Dual−range migrant
ID151, 2017: Dual−range migrant
Net Squared Displacement (km)
Day (starting July 1st)

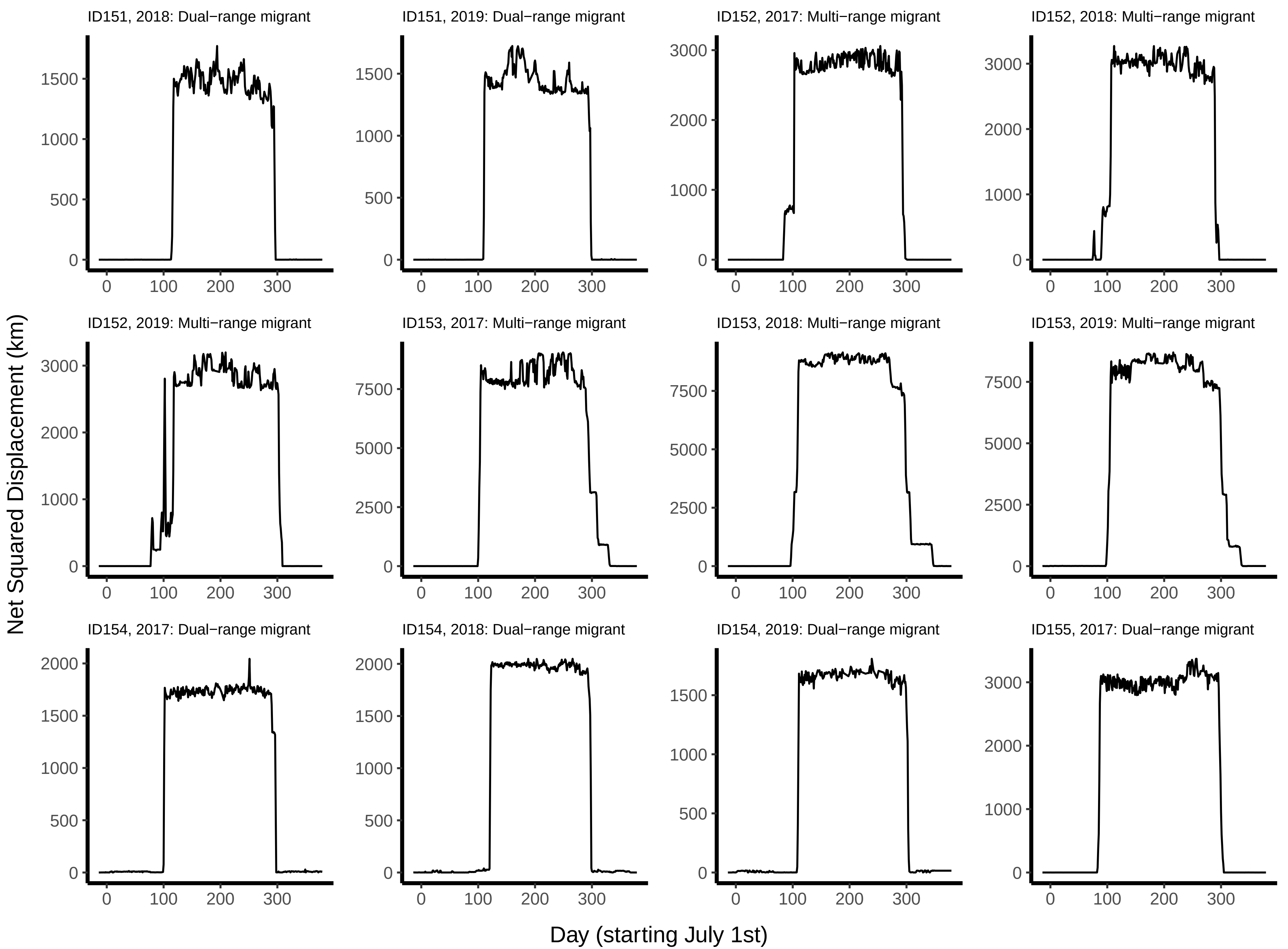
ID151, 2018: Dual−range migrant
ID151, 2019: Dual−range migrant
ID152, 2017: Multi−range migrant
ID152, 2018: Multi−range migrant
ID152, 2019: Multi−range migrant
ID153, 2017: Multi−range migrant
ID153, 2018: Multi−range migrant
ID153, 2019: Multi−range migrant
ID154, 2017: Dual−range migrant
ID154, 2018: Dual−range migrant
ID154, 2019: Dual−range migrant
ID155, 2017: Dual−range migrant
Net Squared Displacement (km)
Day (starting July 1st)

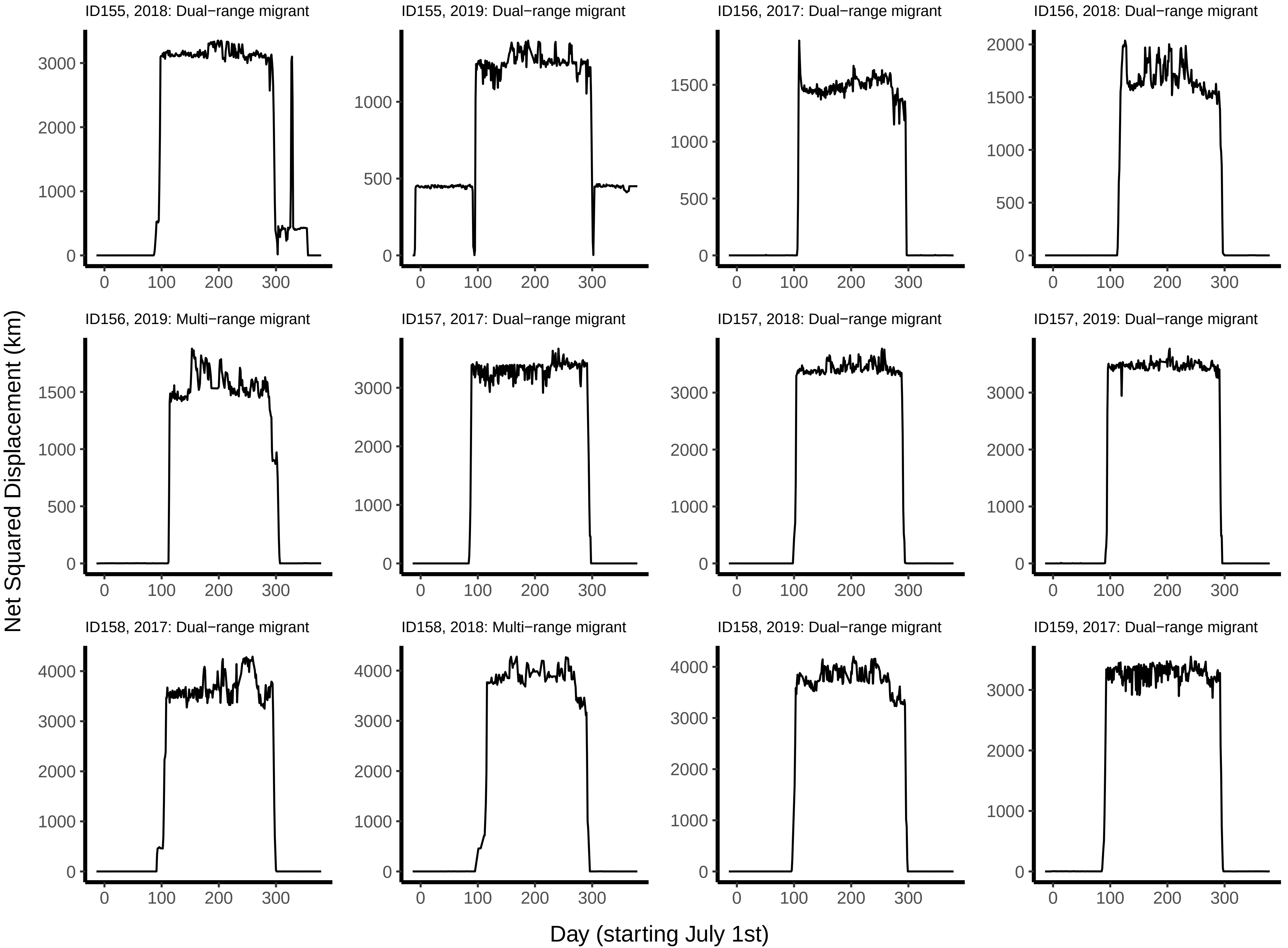
ID155, 2018: Dual−range migrant
ID155, 2019: Dual−range migrant
ID156, 2017: Dual−range migrant
ID156, 2018: Dual−range migrant
ID156, 2019: Multi−range migrant
ID157, 2017: Dual−range migrant
ID157, 2018: Dual−range migrant
ID157, 2019: Dual−range migrant
ID158, 2017: Dual−range migrant
ID158, 2018: Multi−range migrant
ID158, 2019: Dual−range migrant
ID159, 2017: Dual−range migrant
Net Squared Displacement (km)
Day (starting July 1st)
0
100
200
300

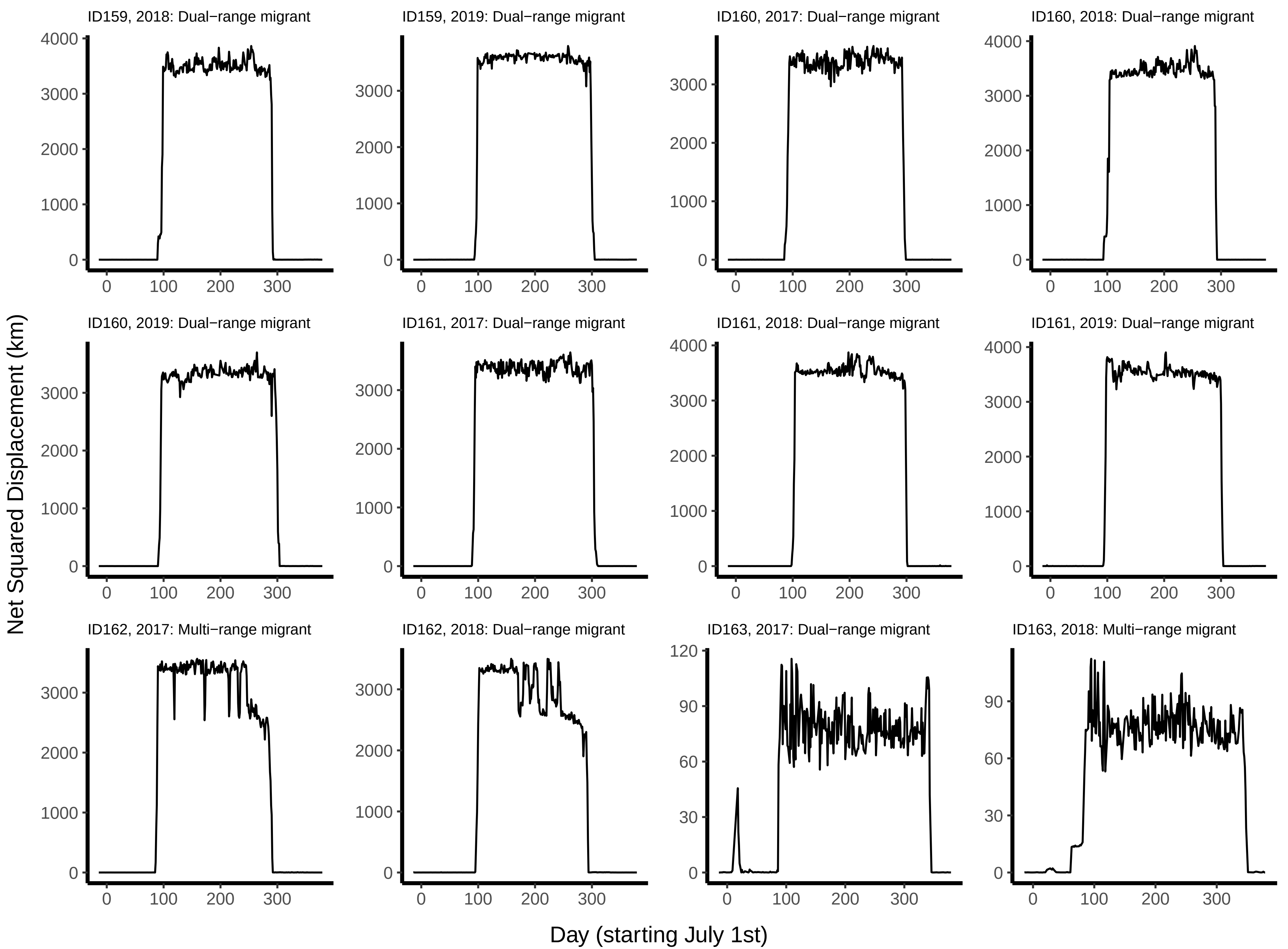

ID159, 2018: Dual−range migrant
ID159, 2019: Dual−range migrant
ID160, 2017: Dual−range migrant
ID160, 2018: Dual−range migrant
ID160, 2019: Dual−range migrant
ID161, 2017: Dual−range migrant
ID161, 2018: Dual−range migrant
ID161, 2019: Dual−range migrant
ID162, 2017: Multi−range migrant
ID162, 2018: Dual−range migrant
ID163, 2017: Dual−range migrant
ID163, 2018: Multi−range migrant
Net Squared Displacement (km)
Day (starting July 1st)

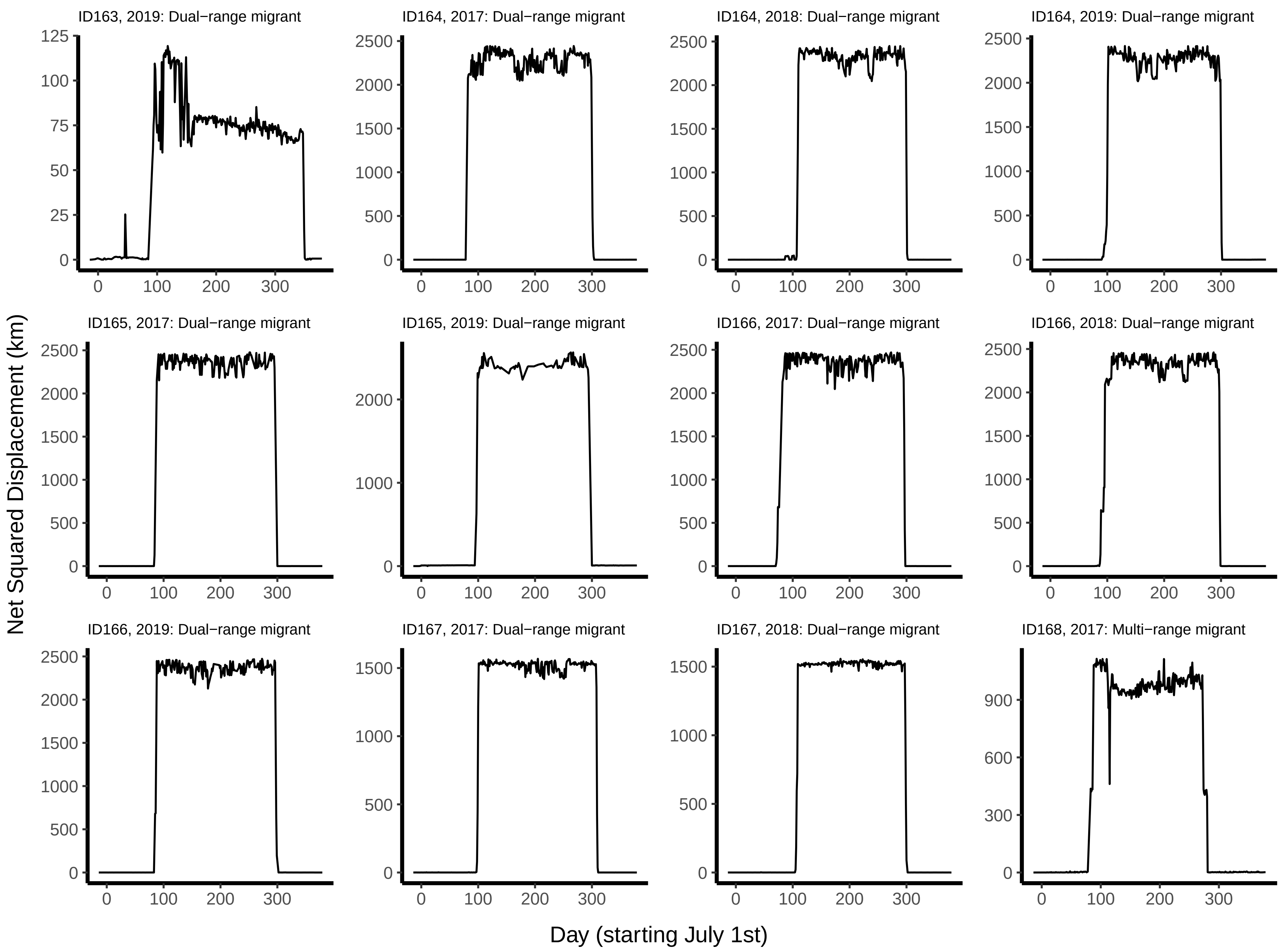
ID163, 2019: Dual−range migrant
ID164, 2017: Dual−range migrant
ID164, 2018: Dual−range migrant
ID164, 2019: Dual−range migrant
ID165, 2017: Dual−range migrant
ID165, 2019: Dual−range migrant
ID166, 2017: Dual−range migrant
ID166, 2018: Dual−range migrant
ID166, 2019: Dual−range migrant
ID167, 2017: Dual−range migrant
ID167, 2018: Dual−range migrant
ID168, 2017: Multi−range migrant
Net Squared Displacement (km)
Day (starting July 1st)

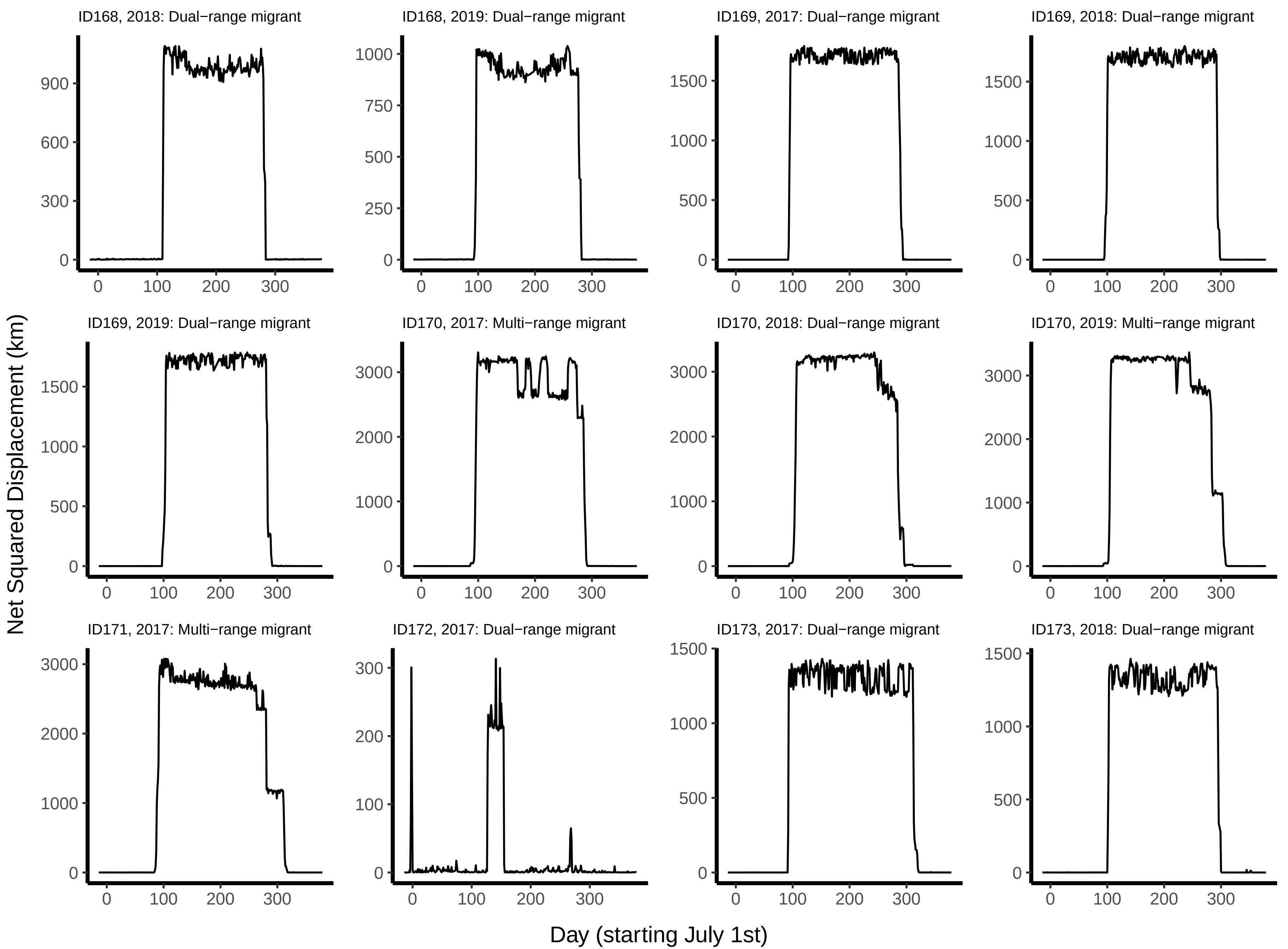
ID168, 2018: Dual−range migrant
ID168, 2019: Dual−range migrant
ID169, 2017: Dual−range migrant
ID169, 2018: Dual−range migrant
ID169, 2019: Dual−range migrant
ID170, 2017: Multi−range migrant
ID170, 2018: Dual−range migrant
ID170, 2019: Multi−range migrant
ID171, 2017: Multi−range migrant
ID172, 2017: Dual−range migrant
ID173, 2017: Dual−range migrant
ID173, 2018: Dual−range migrant
Net Squared Displacement (km)
Day (starting July 1st)

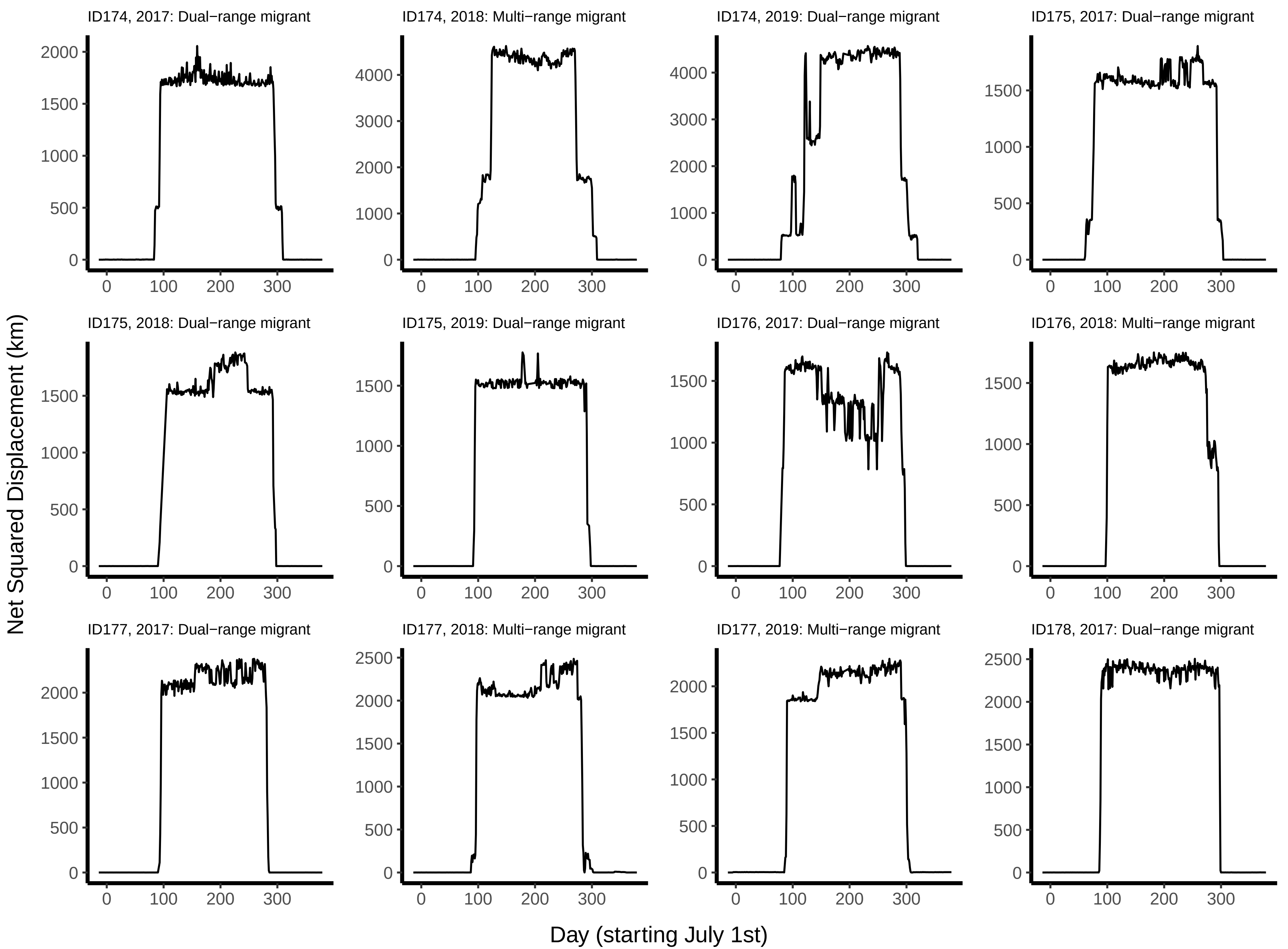

ID174, 2017: Dual−range migrant
ID174, 2018: Multi−range migrant
ID174, 2019: Dual−range migrant
ID175, 2017: Dual−range migrant
ID175, 2018: Dual−range migrant
ID175, 2019: Dual−range migrant
ID176, 2017: Dual−range migrant
ID176, 2018: Multi−range migrant
ID177, 2017: Dual−range migrant
ID177, 2018: Multi−range migrant
ID177, 2019: Multi−range migrant
ID178, 2017: Dual−range migrant
Net Squared Displacement (km)
Day (starting July 1st)

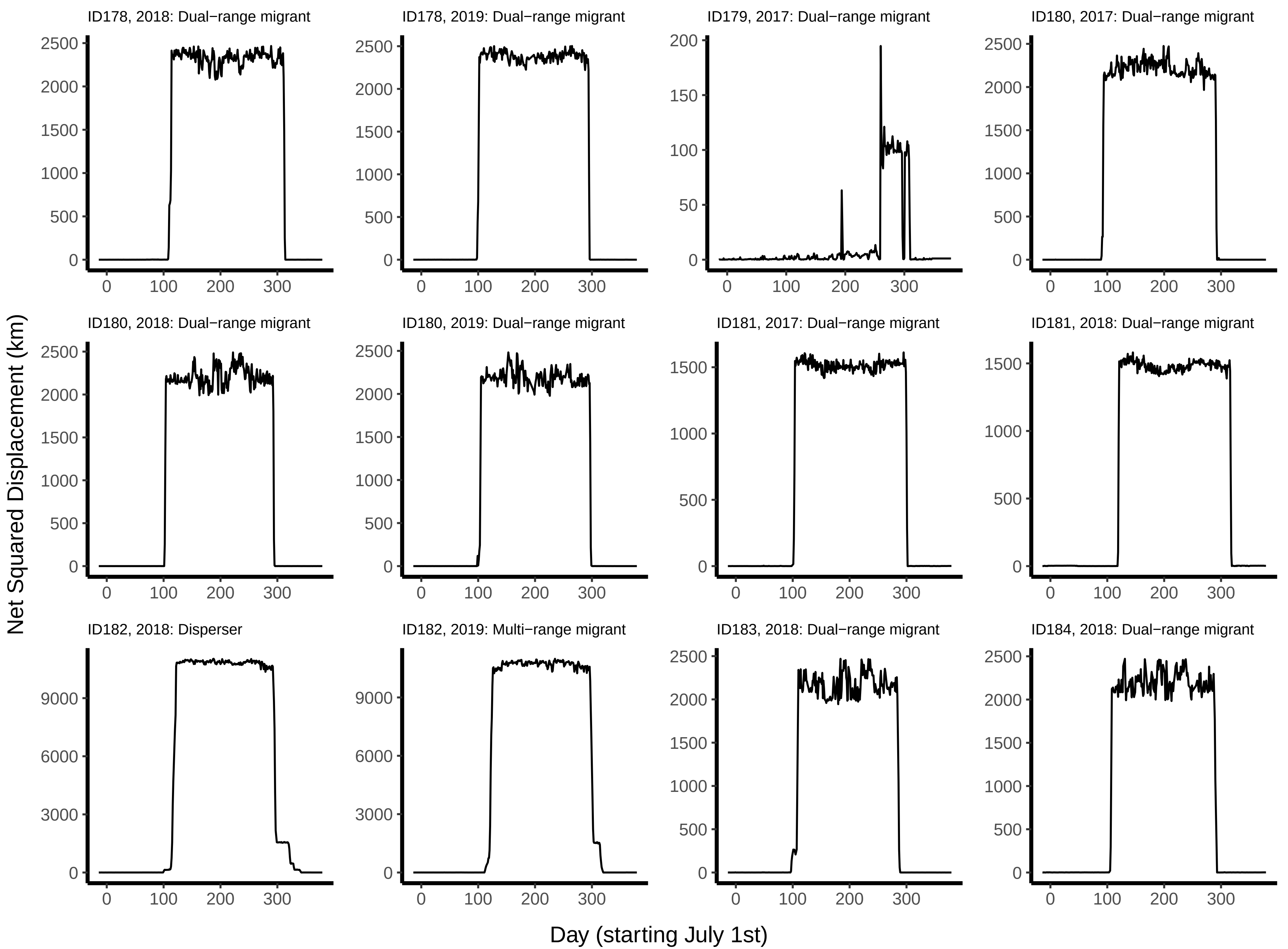

ID178, 2018: Dual−range migrant
ID178, 2019: Dual−range migrant
ID179, 2017: Dual−range migrant
ID180, 2017: Dual−range migrant
ID180, 2018: Dual−range migrant
ID180, 2019: Dual−range migrant
ID181, 2017: Dual−range migrant
ID181, 2018: Dual−range migrant
ID182, 2018: Disperser
ID182, 2019: Multi−range migrant
ID183, 2018: Dual−range migrant
ID184, 2018: Dual−range migrant
Net Squared Displacement (km)
Day (starting July 1st)

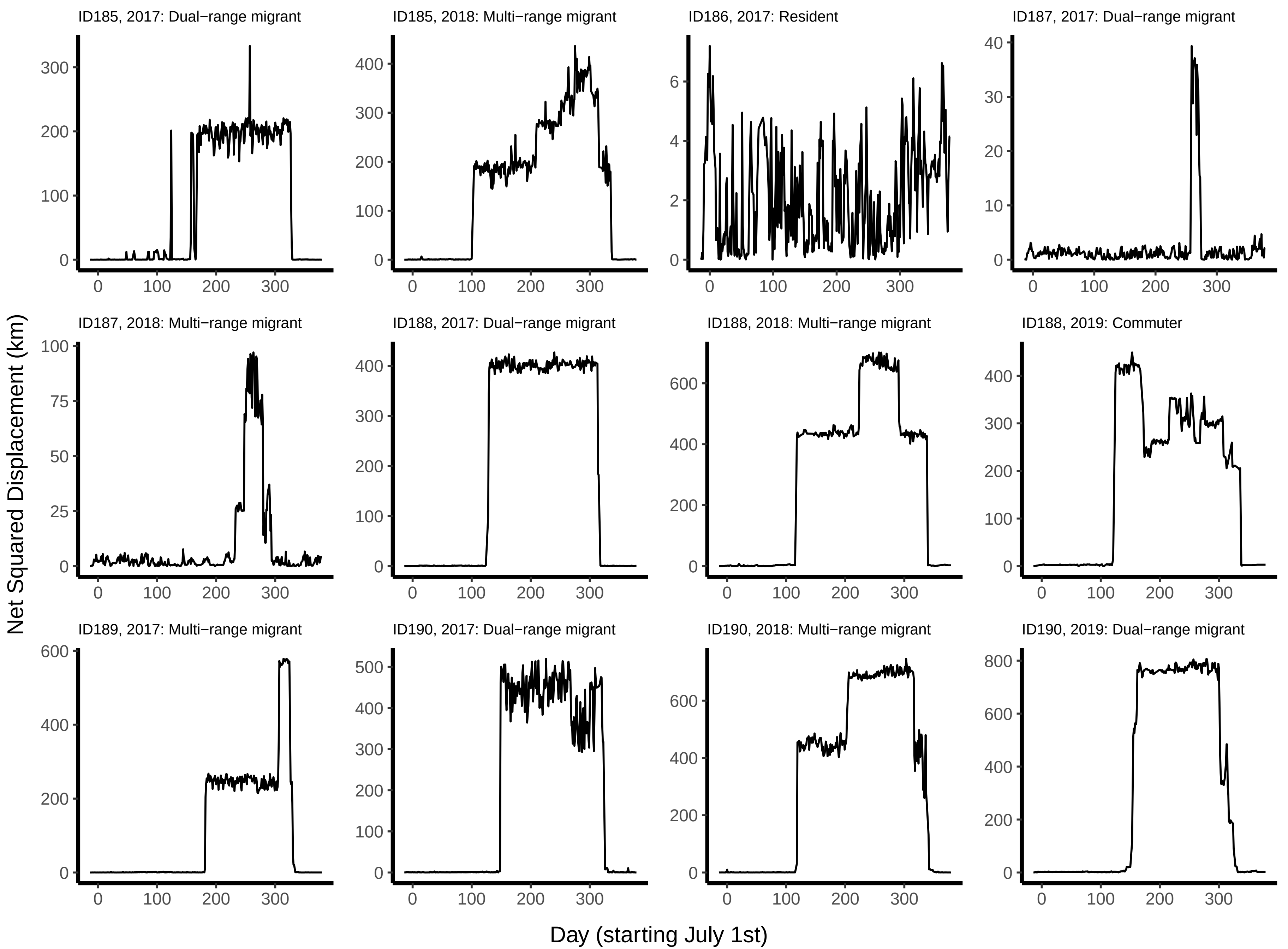
ID185, 2017: Dual−range migrant
ID185, 2018: Multi−range migrant
ID186, 2017: Resident
ID187, 2017: Dual−range migrant
ID187, 2018: Multi−range migrant
ID188, 2017: Dual−range migrant
ID188, 2018: Multi−range migrant
ID188, 2019: Commuter
ID189, 2017: Multi−range migrant
ID190, 2017: Dual−range migrant
ID190, 2018: Multi−range migrant
ID190, 2019: Dual−range migrant
Net Squared Displacement (km)
Day (starting July 1st)

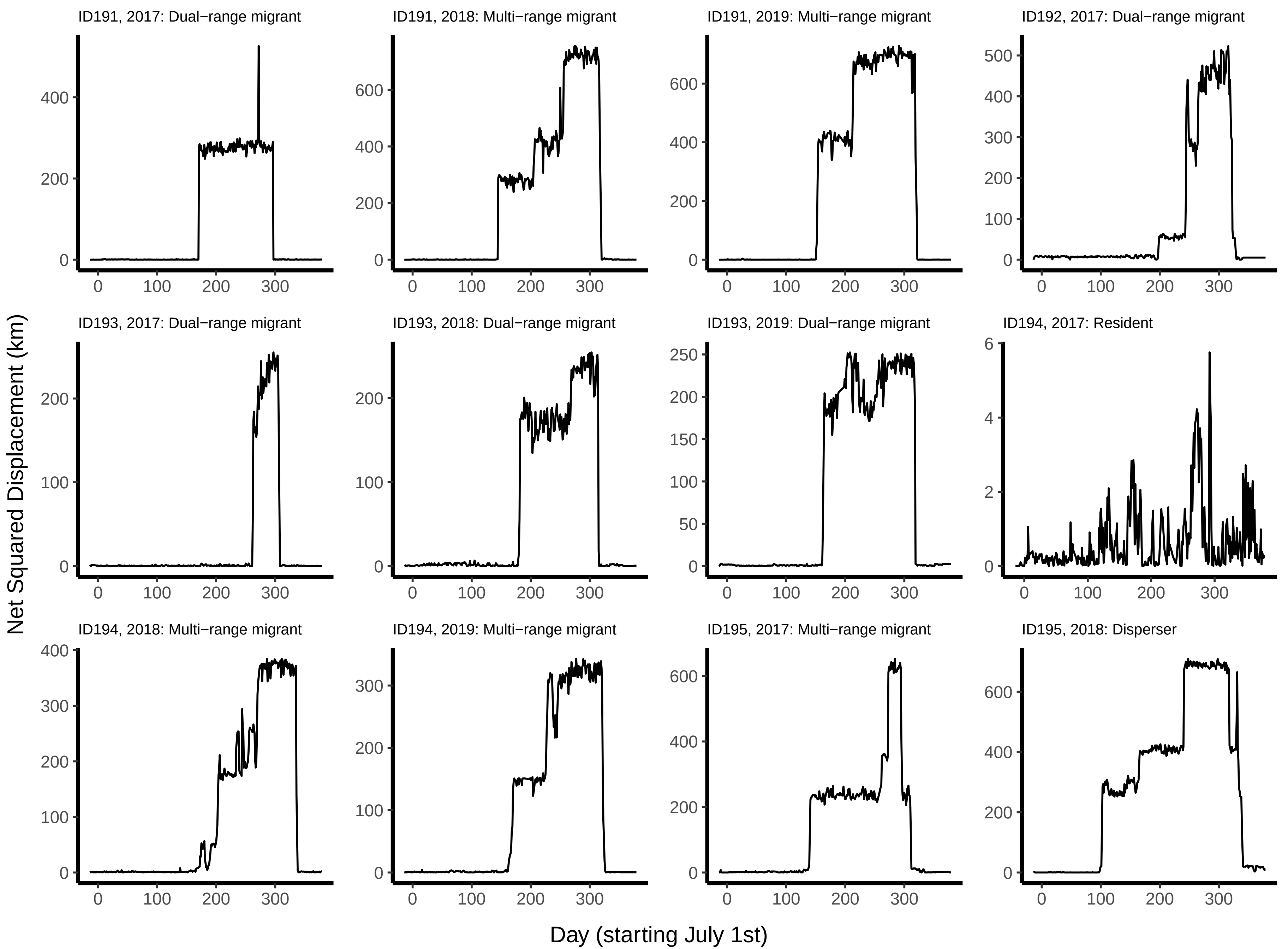

ID191, 2017: Dual−range migrant
ID191, 2018: Multi−range migrant
ID191, 2019: Multi−range migrant
ID192, 2017: Dual−range migrant
ID193, 2017: Dual−range migrant
ID193, 2018: Dual−range migrant
ID193, 2019: Dual−range migrant
ID194, 2017: Resident
ID194, 2018: Multi−range migrant
ID194, 2019: Multi−range migrant
ID195, 2017: Multi−range migrant
ID195, 2018: Disperser
Net Squared Displacement (km)
Day (starting July 1st)
0
100
200
300
400
500
600
50
150
250
2
4
6

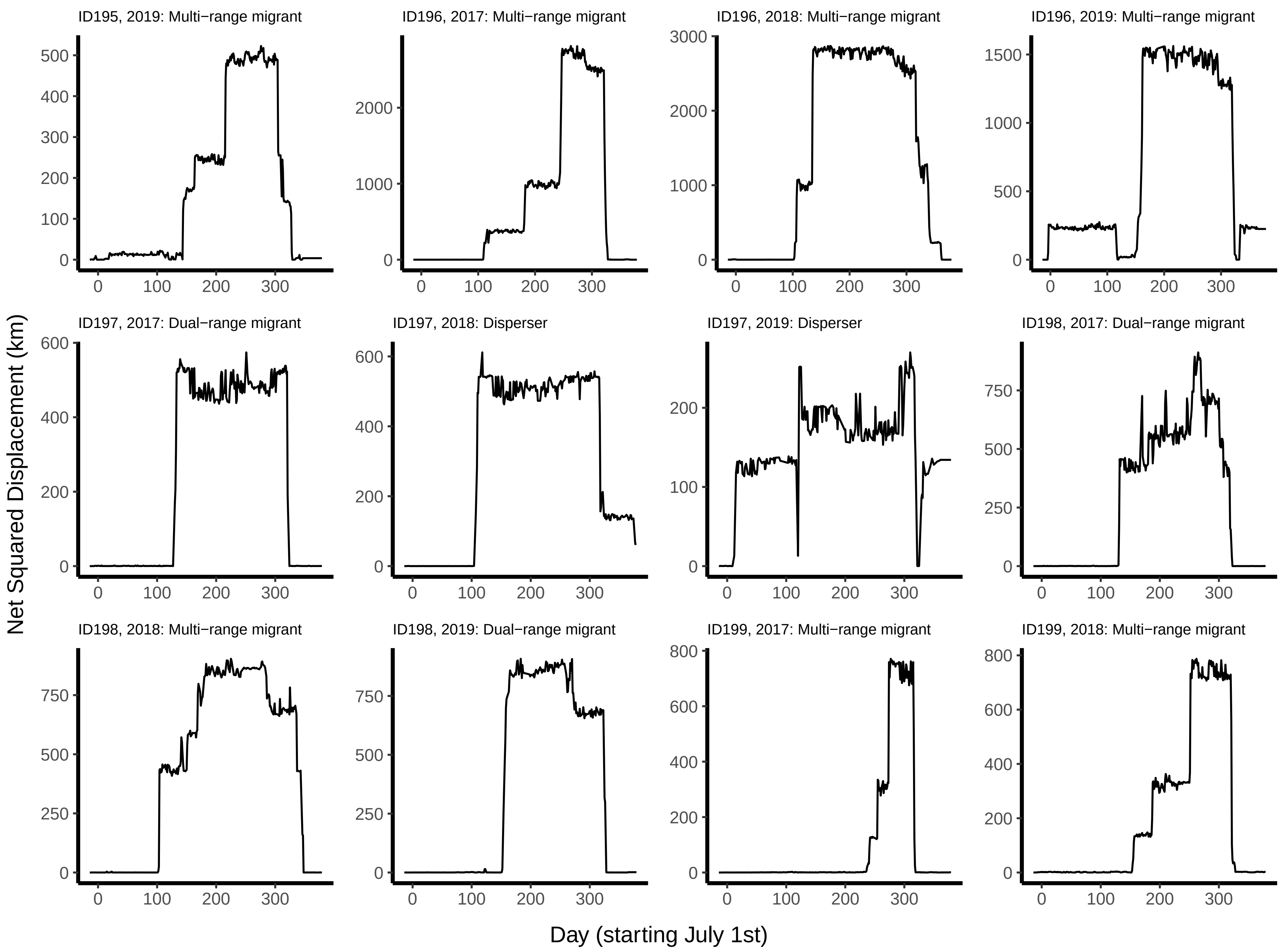

ID195, 2019: Multi−range migrant
ID196, 2017: Multi−range migrant
ID196, 2018: Multi−range migrant
ID196, 2019: Multi−range migrant
ID197, 2017: Dual−range migrant
ID197, 2018: Disperser
ID197, 2019: Disperser
ID198, 2017: Dual−range migrant
ID198, 2018: Multi−range migrant
ID198, 2019: Dual−range migrant
ID199, 2017: Multi−range migrant
ID199, 2018: Multi−range migrant
Net Squared Displacement (km)
Day (starting July 1st)

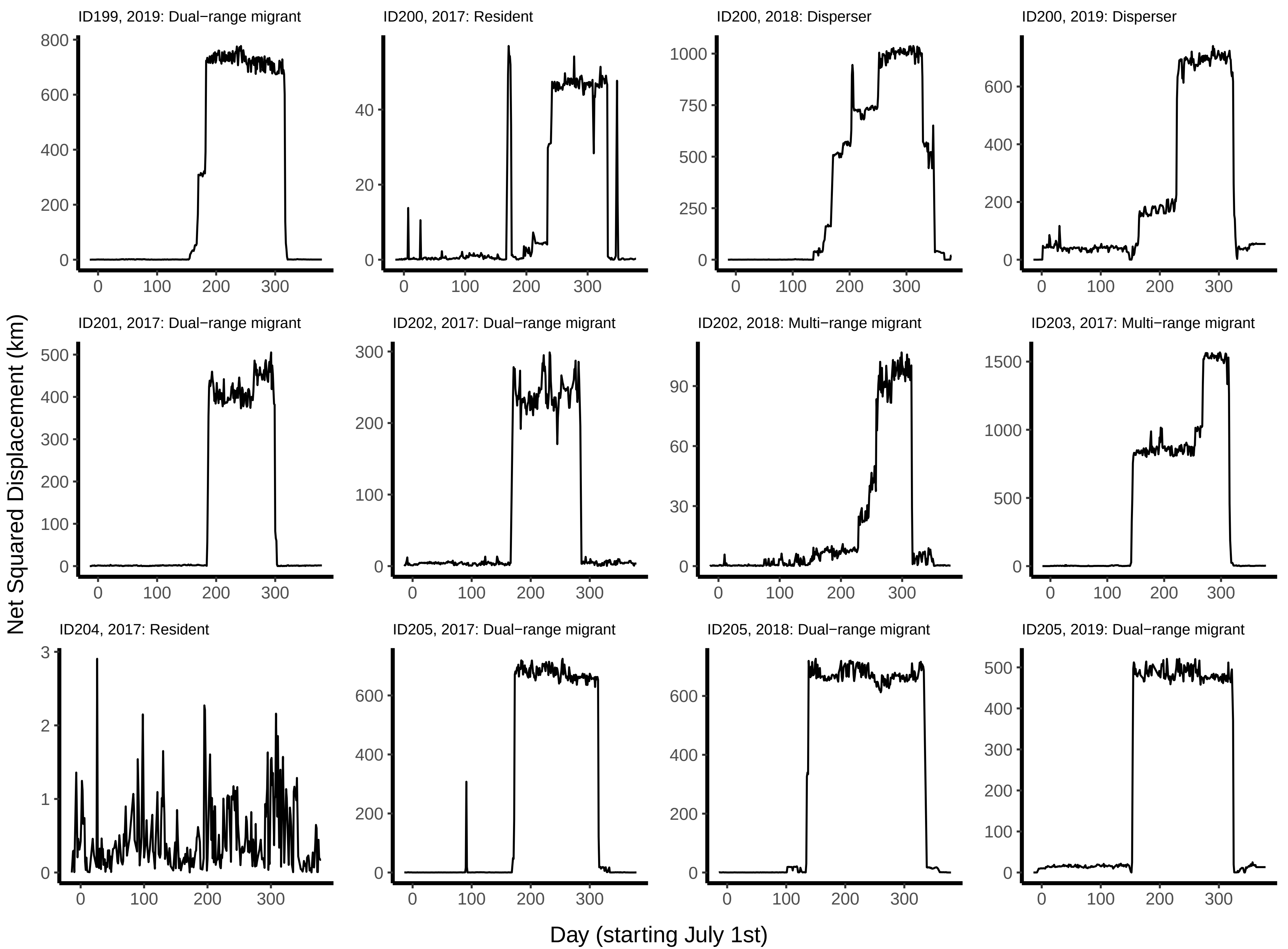

ID199, 2019: Dual−range migrant
ID200, 2017: Resident
ID200, 2018: Disperser
ID200, 2019: Disperser
ID201, 2017: Dual−range migrant
ID202, 2017: Dual−range migrant
ID202, 2018: Multi−range migrant
ID203, 2017: Multi−range migrant
ID204, 2017: Resident
ID205, 2017: Dual−range migrant
ID205, 2018: Dual−range migrant
ID205, 2019: Dual−range migrant
Net Squared Displacement (km)
Day (starting July 1st)

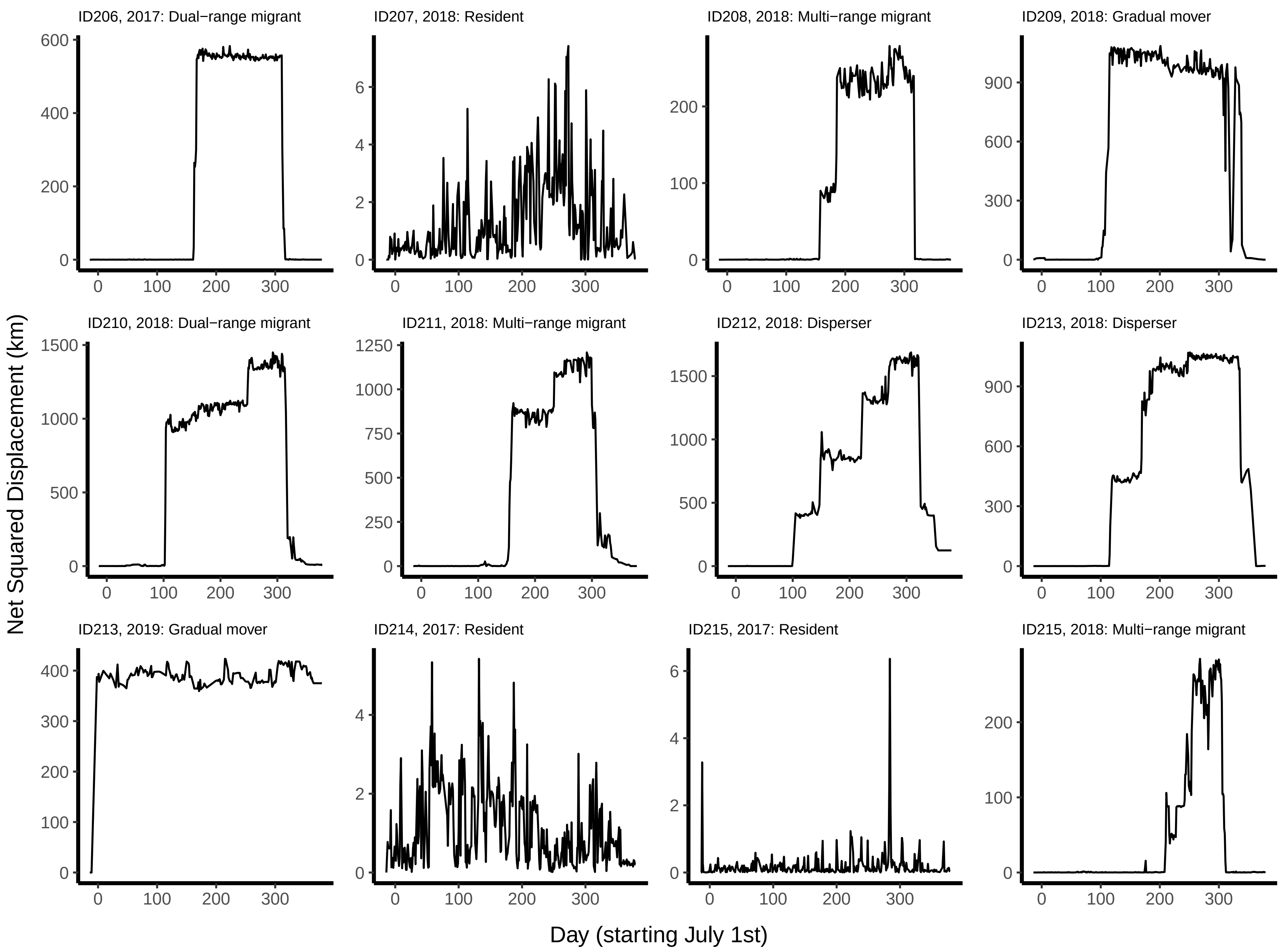
ID206, 2017: Dual−range migrant
ID207, 2018: Resident
ID208, 2018: Multi−range migrant
ID209, 2018: Gradual mover
ID210, 2018: Dual−range migrant
ID211, 2018: Multi−range migrant
ID212, 2018: Disperser
ID213, 2018: Disperser
ID213, 2019: Gradual mover
ID214, 2017: Resident
ID215, 2017: Resident
ID215, 2018: Multi−range migrant
Net Squared Displacement (km)
Day (starting July 1st)

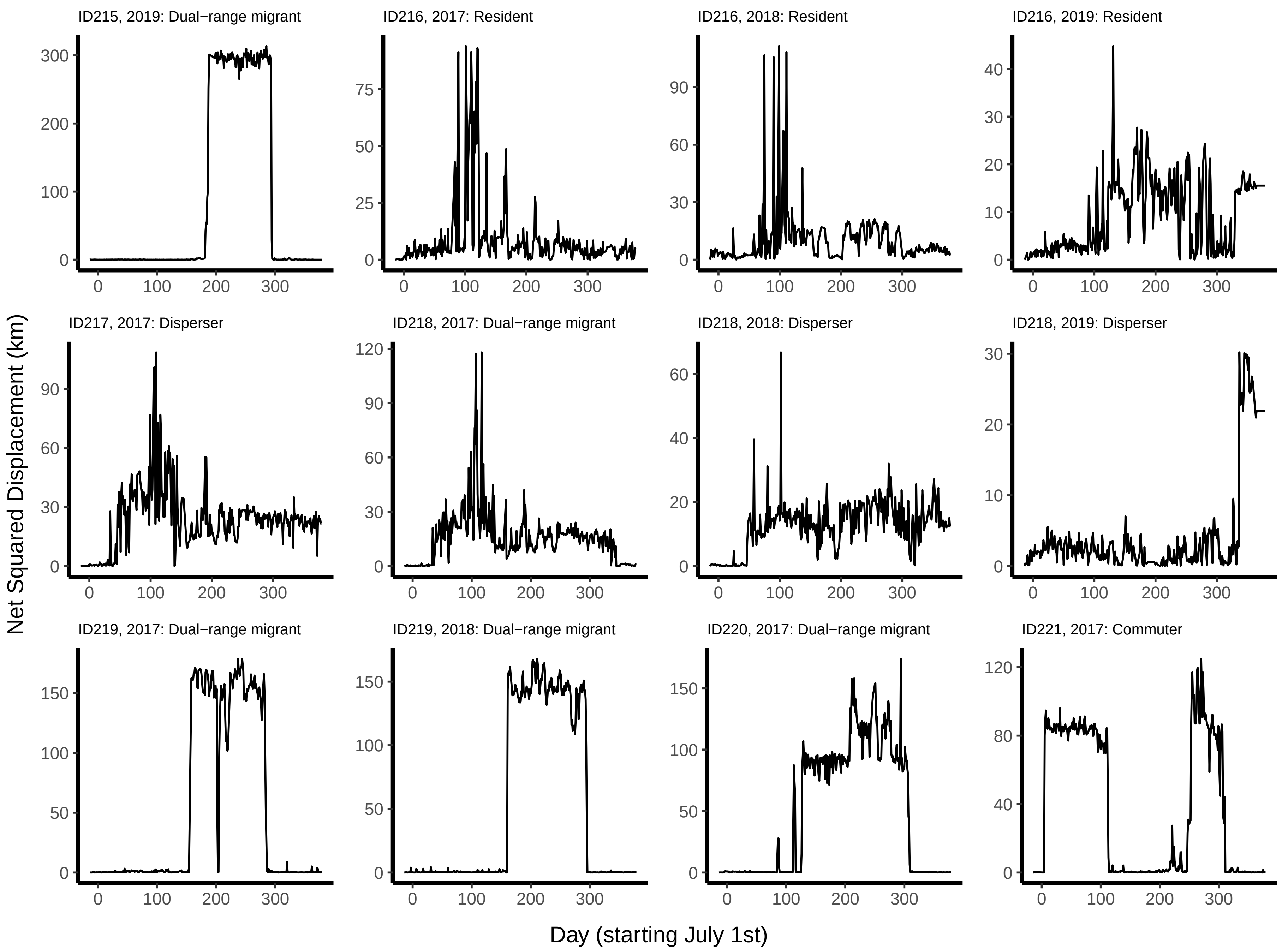
ID215, 2019: Dual−range migrant
ID216, 2017: Resident
ID216, 2018: Resident
ID216, 2019: Resident
ID217, 2017: Disperser
ID218, 2017: Dual−range migrant
ID218, 2018: Disperser
ID218, 2019: Disperser
ID219, 2017: Dual−range migrant
ID219, 2018: Dual−range migrant
ID220, 2017: Dual−range migrant
ID221, 2017: Commuter
Net Squared Displacement (km)
Day (starting July 1st)

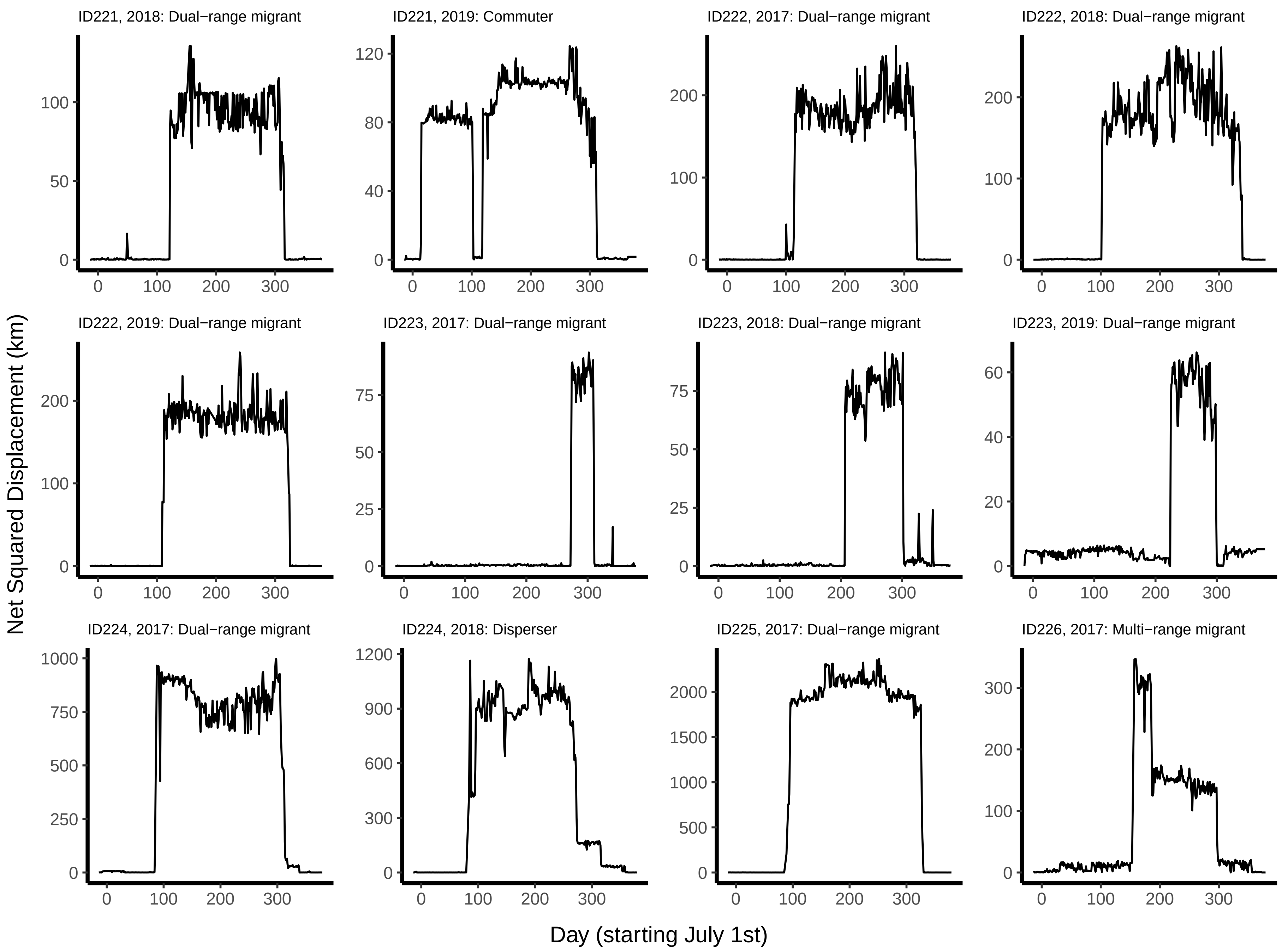

ID221, 2018: Dual−range migrant
ID221, 2019: Commuter
ID222, 2017: Dual−range migrant
ID222, 2018: Dual−range migrant
ID222, 2019: Dual−range migrant
ID223, 2017: Dual−range migrant
ID223, 2018: Dual−range migrant
ID223, 2019: Dual−range migrant
ID224, 2017: Dual−range migrant
ID224, 2018: Disperser
ID225, 2017: Dual−range migrant
ID226, 2017: Multi−range migrant
Net Squared Displacement (km)
Day (starting July 1st)

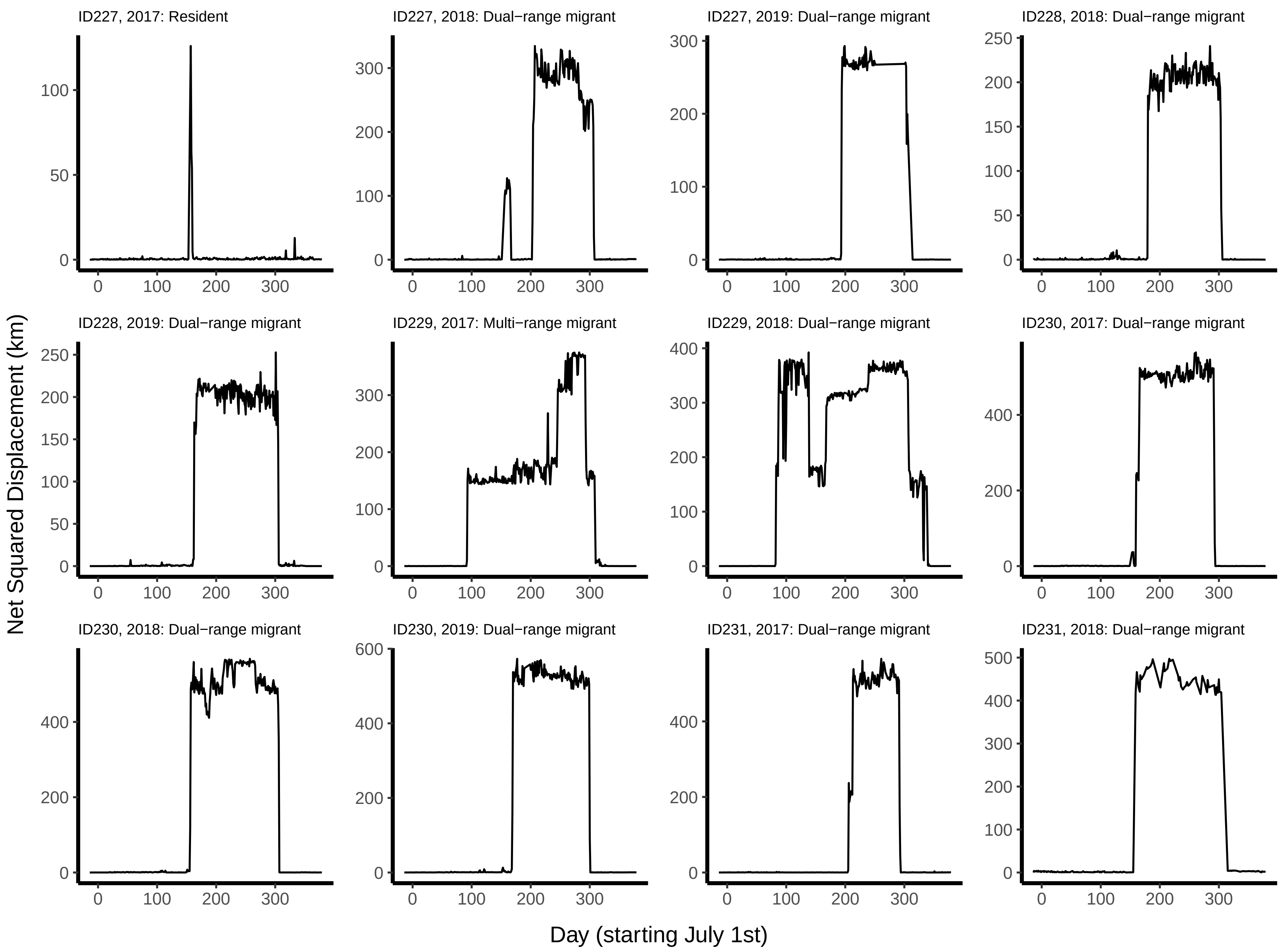

ID227, 2017: Resident
ID227, 2018: Dual−range migrant
ID227, 2019: Dual−range migrant
ID228, 2018: Dual−range migrant
ID228, 2019: Dual−range migrant
ID229, 2017: Multi−range migrant
ID229, 2018: Dual−range migrant
ID230, 2017: Dual−range migrant
ID230, 2018: Dual−range migrant
ID230, 2019: Dual−range migrant
ID231, 2017: Dual−range migrant
ID231, 2018: Dual−range migrant
Net Squared Displacement (km)
Day (starting July 1st)

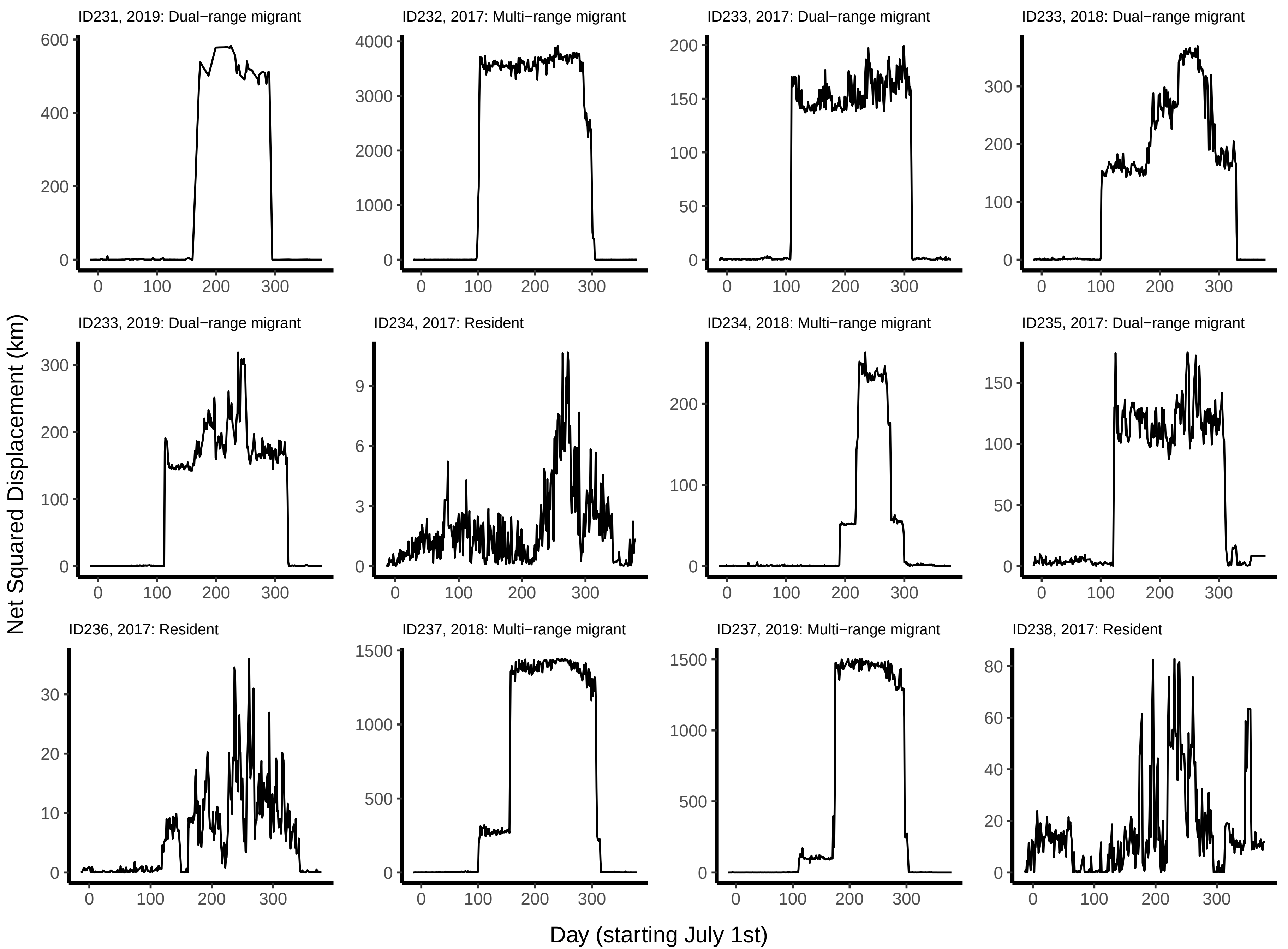

ID231, 2019: Dual−range migrant
ID232, 2017: Multi−range migrant
ID233, 2017: Dual−range migrant
ID233, 2018: Dual−range migrant
ID233, 2019: Dual−range migrant
ID234, 2017: Resident
ID234, 2018: Multi−range migrant
ID235, 2017: Dual−range migrant
ID236, 2017: Resident
ID237, 2018: Multi−range migrant
ID237, 2019: Multi−range migrant
ID238, 2017: Resident
Net Squared Displacement (km)
Day (starting July 1st)

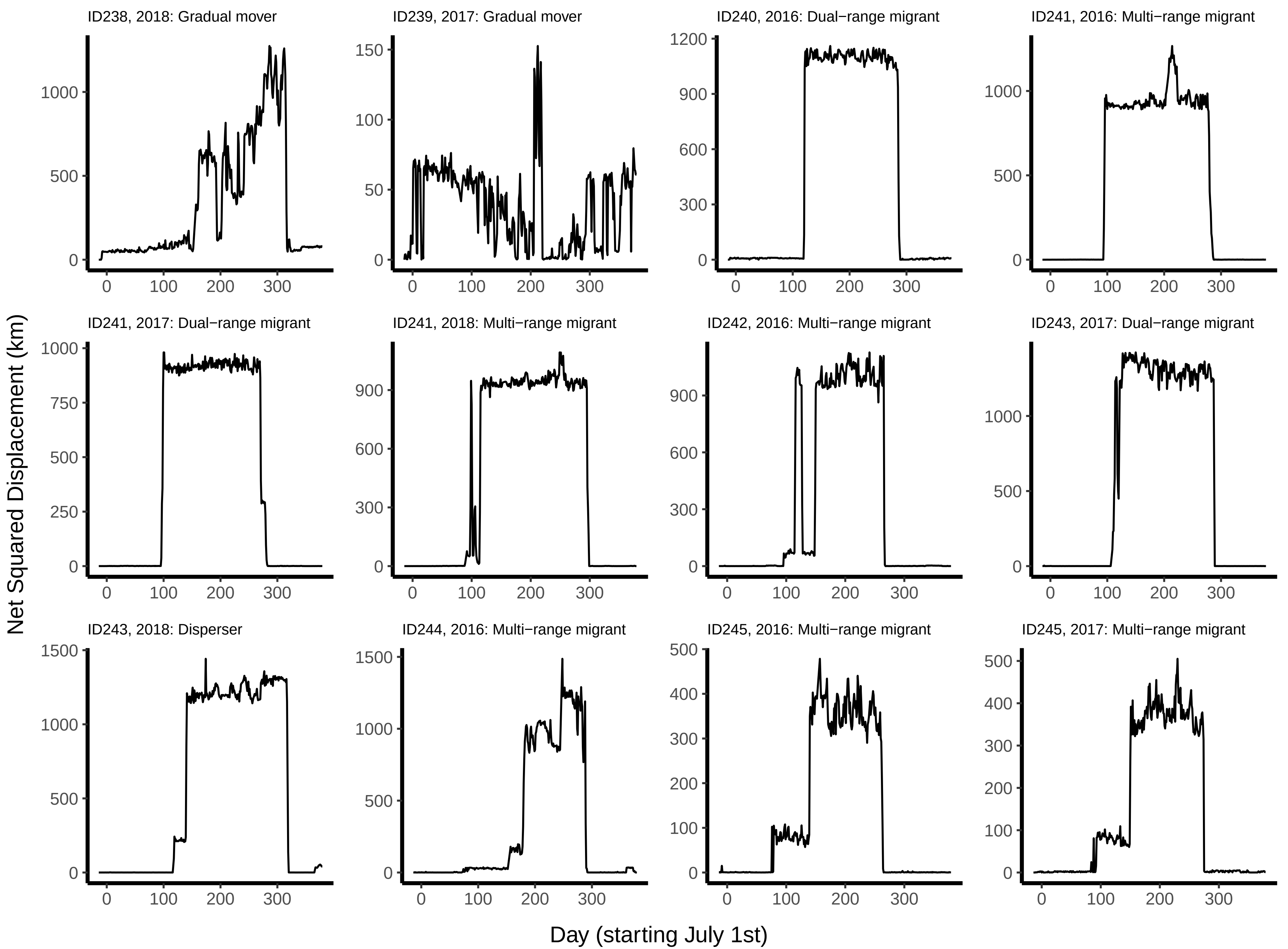

ID238, 2018: Gradual mover
ID239, 2017: Gradual mover
ID240, 2016: Dual−range migrant
ID241, 2016: Multi−range migrant
ID241, 2017: Dual−range migrant
ID241, 2018: Multi−range migrant
ID242, 2016: Multi−range migrant
ID243, 2017: Dual−range migrant
ID243, 2018: Disperser
ID244, 2016: Multi−range migrant
ID245, 2016: Multi−range migrant
ID245, 2017: Multi−range migrant
Net Squared Displacement (km)
Day (starting July 1st)

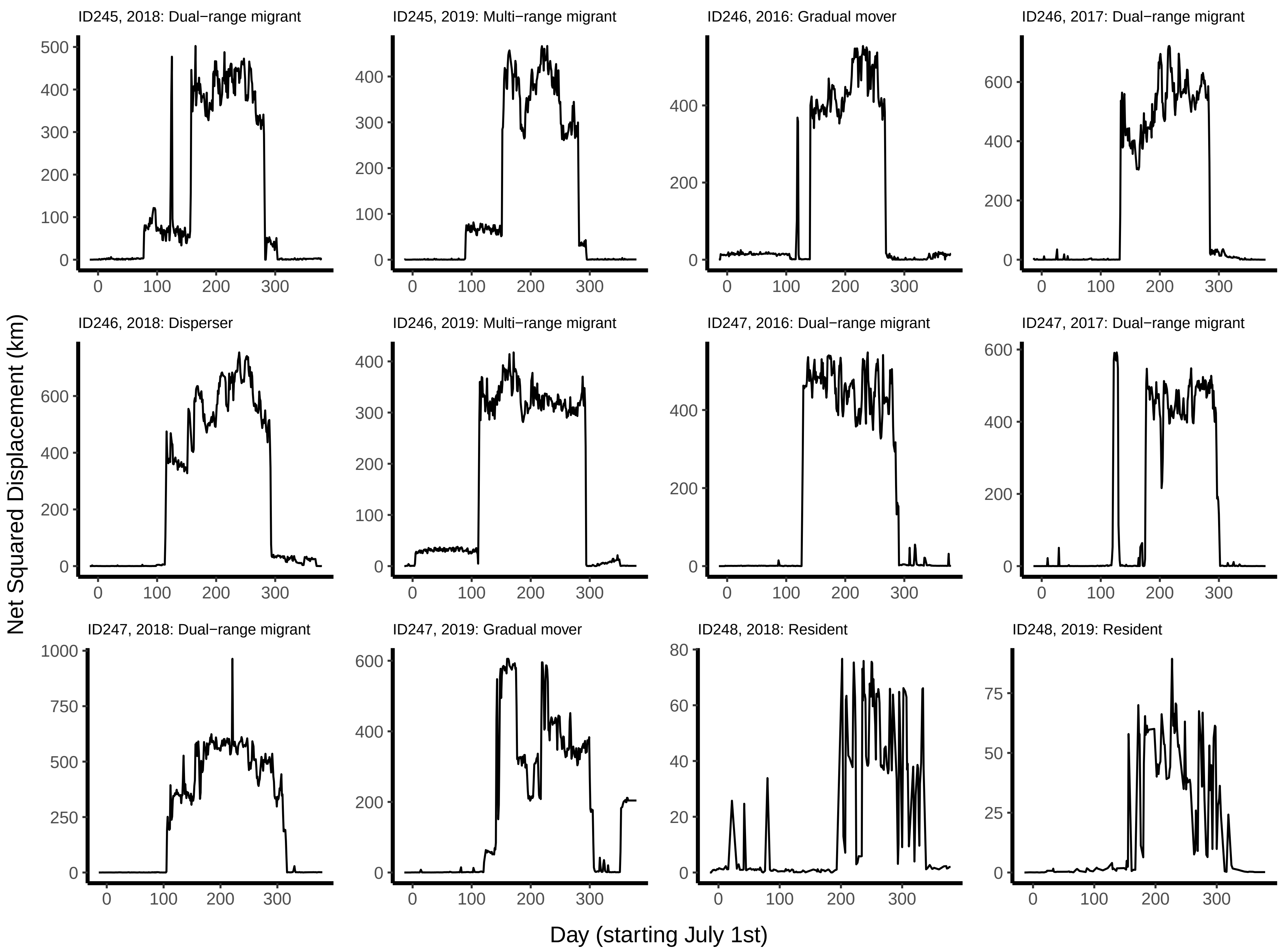

ID245, 2018: Dual−range migrant
ID245, 2019: Multi−range migrant
ID246, 2016: Gradual mover
ID246, 2017: Dual−range migrant
ID246, 2018: Disperser
ID246, 2019: Multi−range migrant
ID247, 2016: Dual−range migrant
ID247, 2017: Dual−range migrant
ID247, 2018: Dual−range migrant
ID247, 2019: Gradual mover
ID248, 2018: Resident
ID248, 2019: Resident
Net Squared Displacement (km)
Day (starting July 1st)

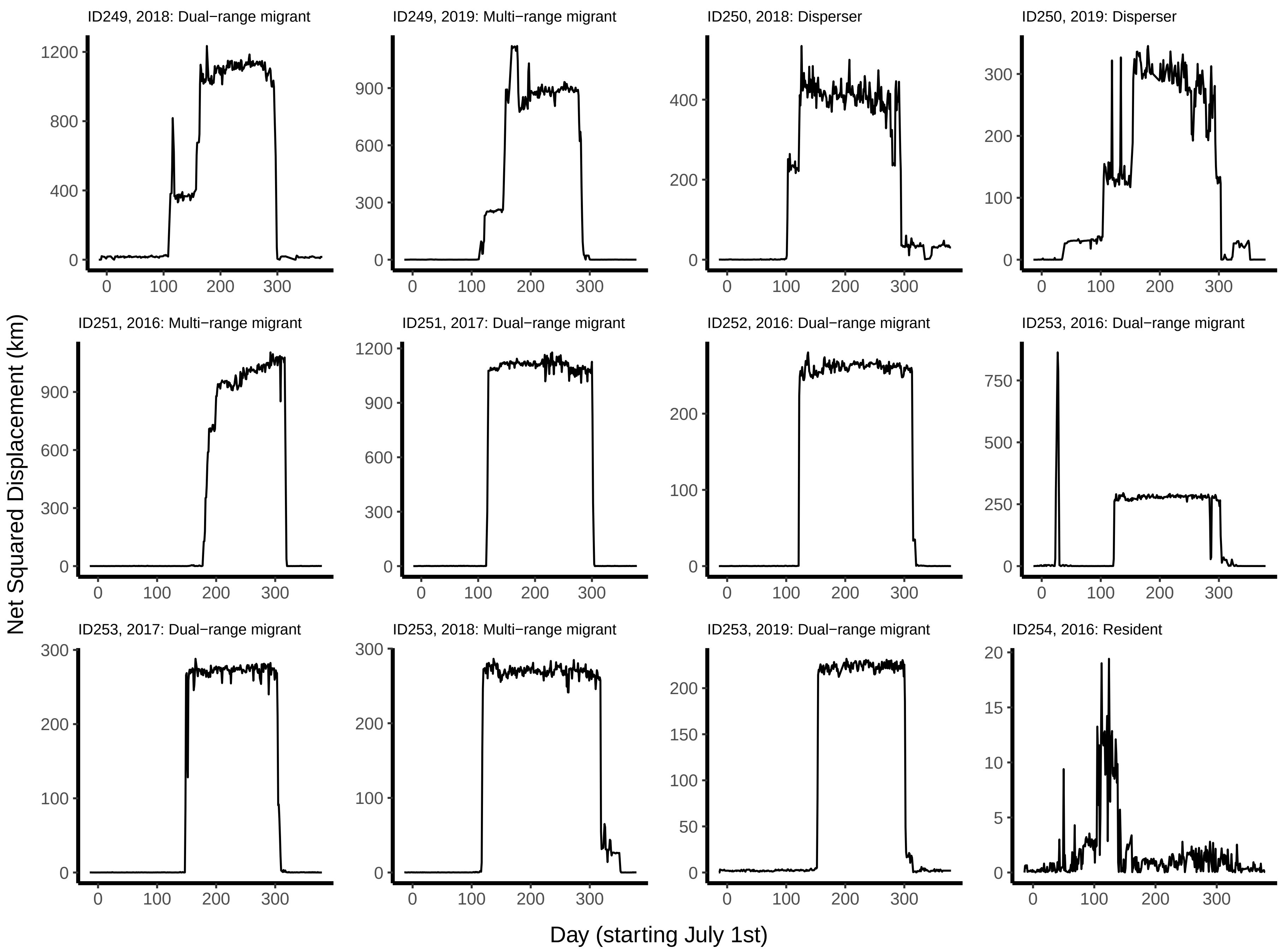

ID249, 2018: Dual−range migrant
ID249, 2019: Multi−range migrant
ID250, 2018: Disperser
ID250, 2019: Disperser
ID251, 2016: Multi−range migrant
ID251, 2017: Dual−range migrant
ID252, 2016: Dual−range migrant
ID253, 2016: Dual−range migrant
ID253, 2017: Dual−range migrant
ID253, 2018: Multi−range migrant
ID253, 2019: Dual−range migrant
ID254, 2016: Resident
Net Squared Displacement (km)
Day (starting July 1st)

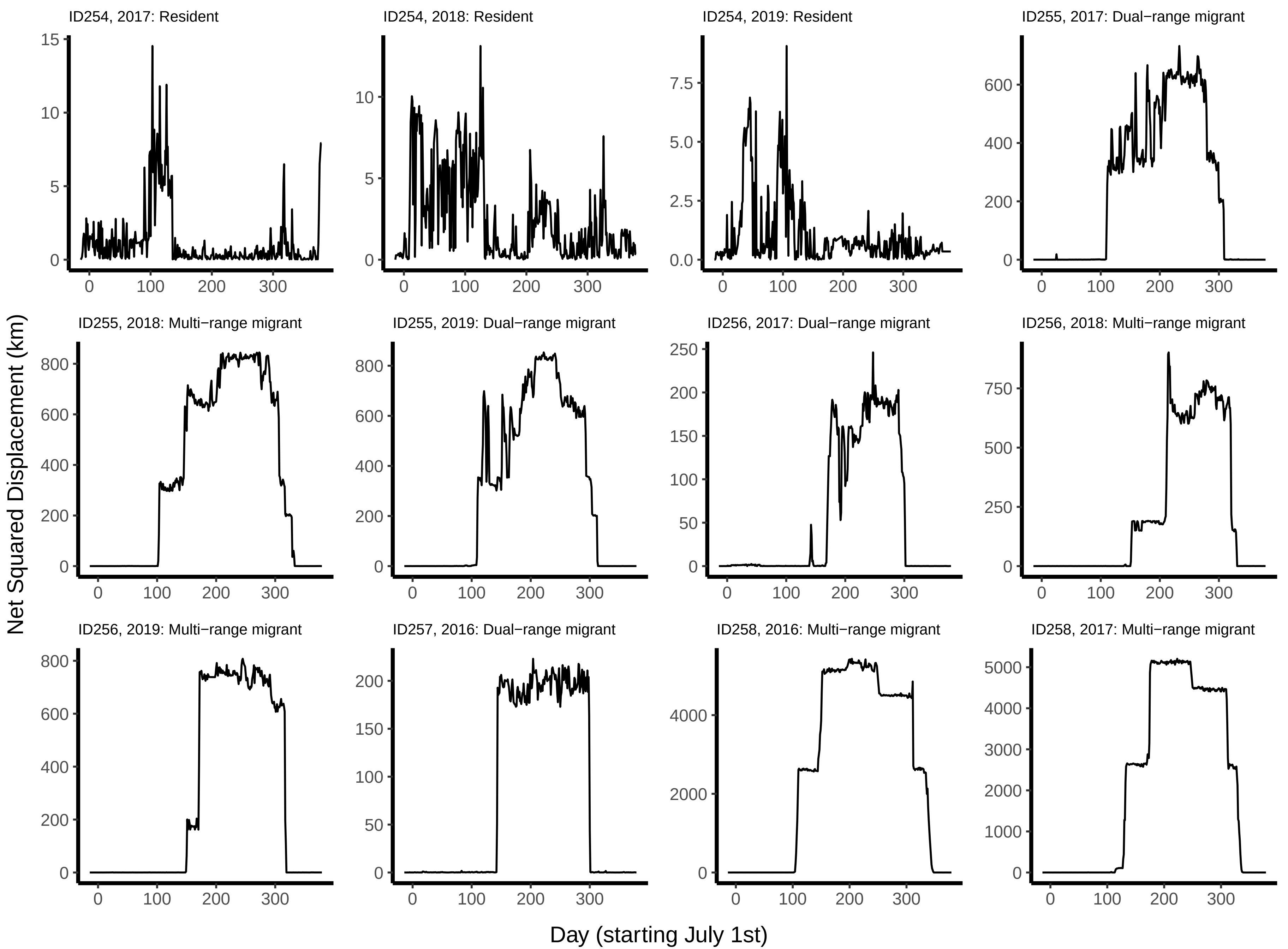
ID254, 2017: Resident
ID254, 2018: Resident
ID254, 2019: Resident
ID255, 2017: Dual−range migrant
ID255, 2018: Multi−range migrant
ID255, 2019: Dual−range migrant
ID256, 2017: Dual−range migrant
ID256, 2018: Multi−range migrant
ID256, 2019: Multi−range migrant
ID257, 2016: Dual−range migrant
ID258, 2016: Multi−range migrant
ID258, 2017: Multi−range migrant
Net Squared Displacement (km)
Day (starting July 1st)

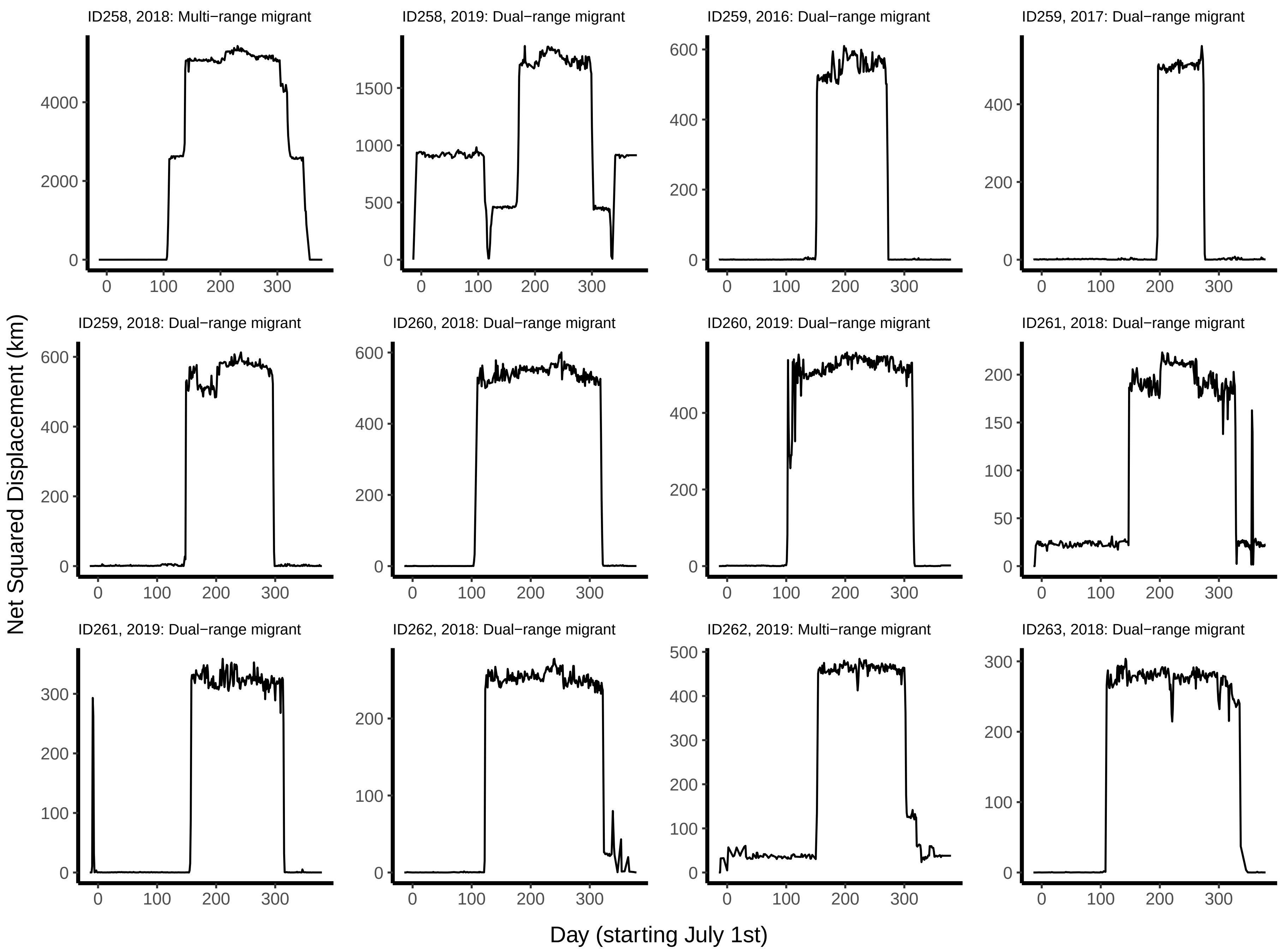

ID258, 2018: Multi−range migrant
ID258, 2019: Dual−range migrant
ID259, 2016: Dual−range migrant
ID259, 2017: Dual−range migrant
ID259, 2018: Dual−range migrant
ID260, 2018: Dual−range migrant
ID260, 2019: Dual−range migrant
ID261, 2018: Dual−range migrant
ID261, 2019: Dual−range migrant
ID262, 2018: Dual−range migrant
ID262, 2019: Multi−range migrant
ID263, 2018: Dual−range migrant
Net Squared Displacement (km)
Day (starting July 1st)

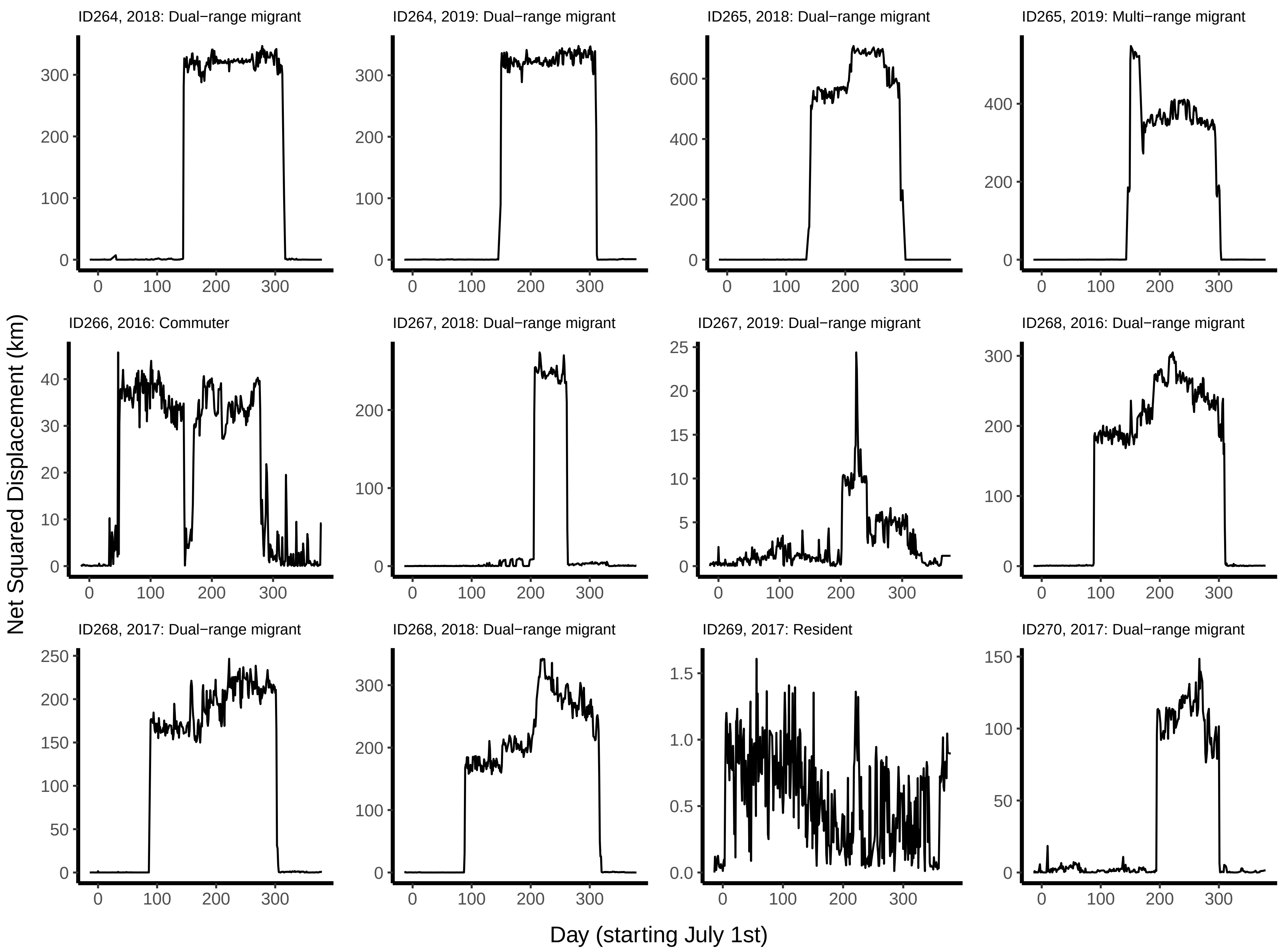

ID264, 2018: Dual−range migrant
ID264, 2019: Dual−range migrant
ID265, 2018: Dual−range migrant
ID265, 2019: Multi−range migrant
ID266, 2016: Commuter
ID267, 2018: Dual−range migrant
ID267, 2019: Dual−range migrant
ID268, 2016: Dual−range migrant
ID268, 2017: Dual−range migrant
ID268, 2018: Dual−range migrant
ID269, 2017: Resident
ID270, 2017: Dual−range migrant
Net Squared Displacement (km)
Day (starting July 1st)

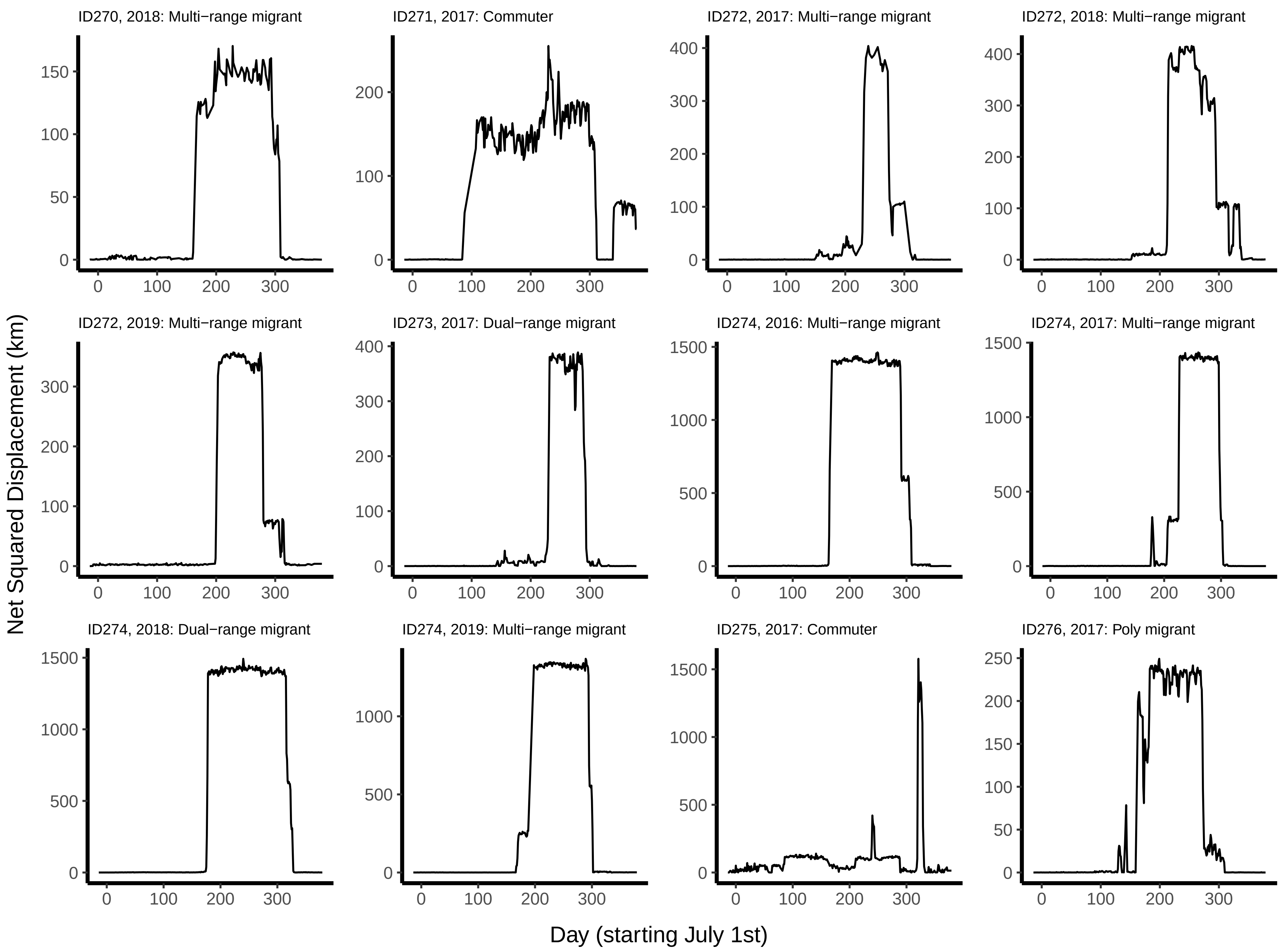
ID270, 2018: Multi−range migrant
ID271, 2017: Commuter
ID272, 2017: Multi−range migrant
ID272, 2018: Multi−range migrant
ID272, 2019: Multi−range migrant
ID273, 2017: Dual−range migrant
ID274, 2016: Multi−range migrant
ID274, 2017: Multi−range migrant
ID274, 2018: Dual−range migrant
ID274, 2019: Multi−range migrant
ID275, 2017: Commuter
ID276, 2017: Poly migrant
Net Squared Displacement (km)
Day (starting July 1st)

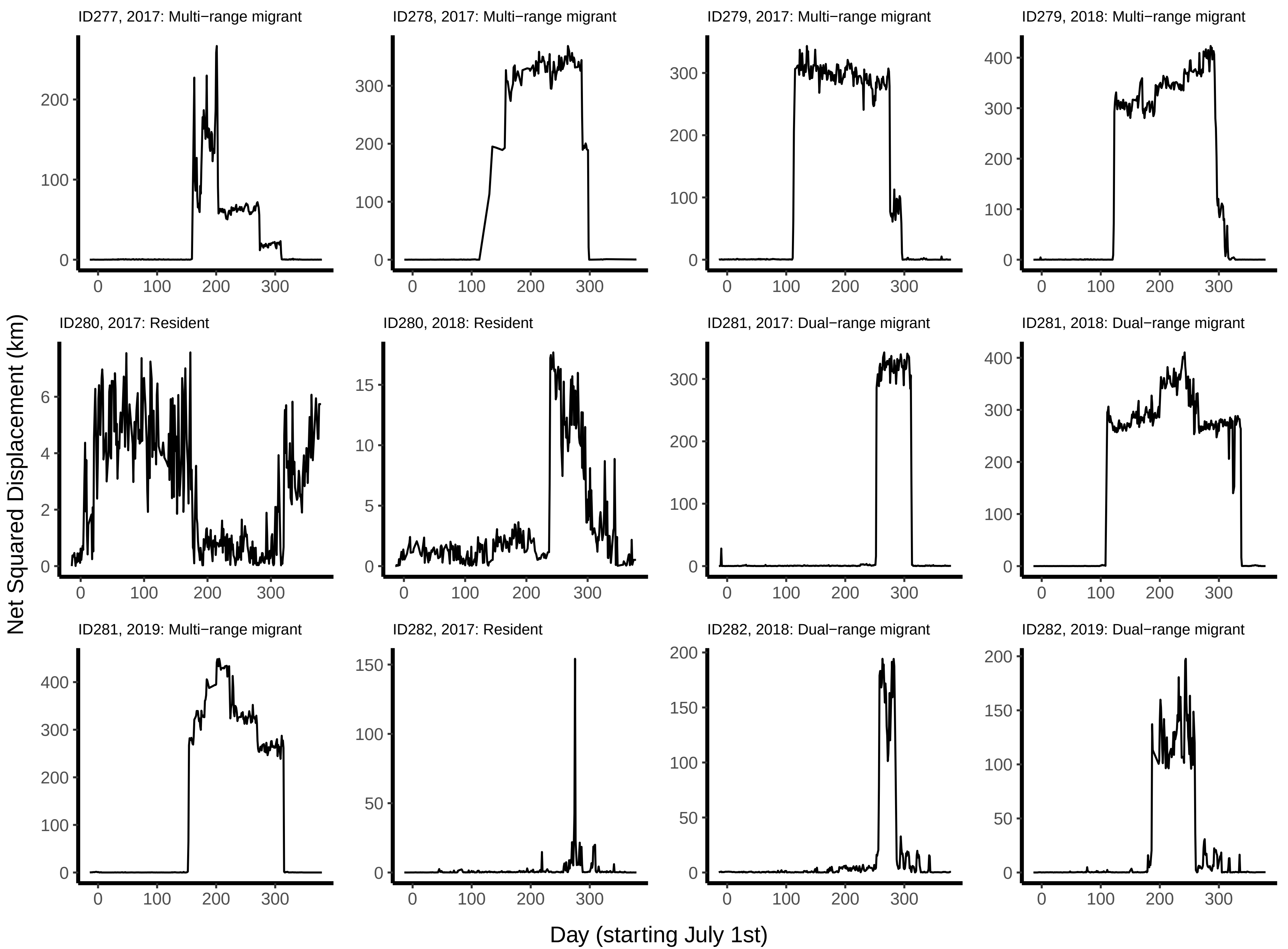
ID277, 2017: Multi−range migrant
ID278, 2017: Multi−range migrant
ID279, 2017: Multi−range migrant
ID279, 2018: Multi−range migrant
ID280, 2017: Resident
ID280, 2018: Resident
ID281, 2017: Dual−range migrant
ID281, 2018: Dual−range migrant
ID281, 2019: Multi−range migrant
ID282, 2017: Resident
ID282, 2018: Dual−range migrant
ID282, 2019: Dual−range migrant
Net Squared Displacement (km)
Day (starting July 1st)

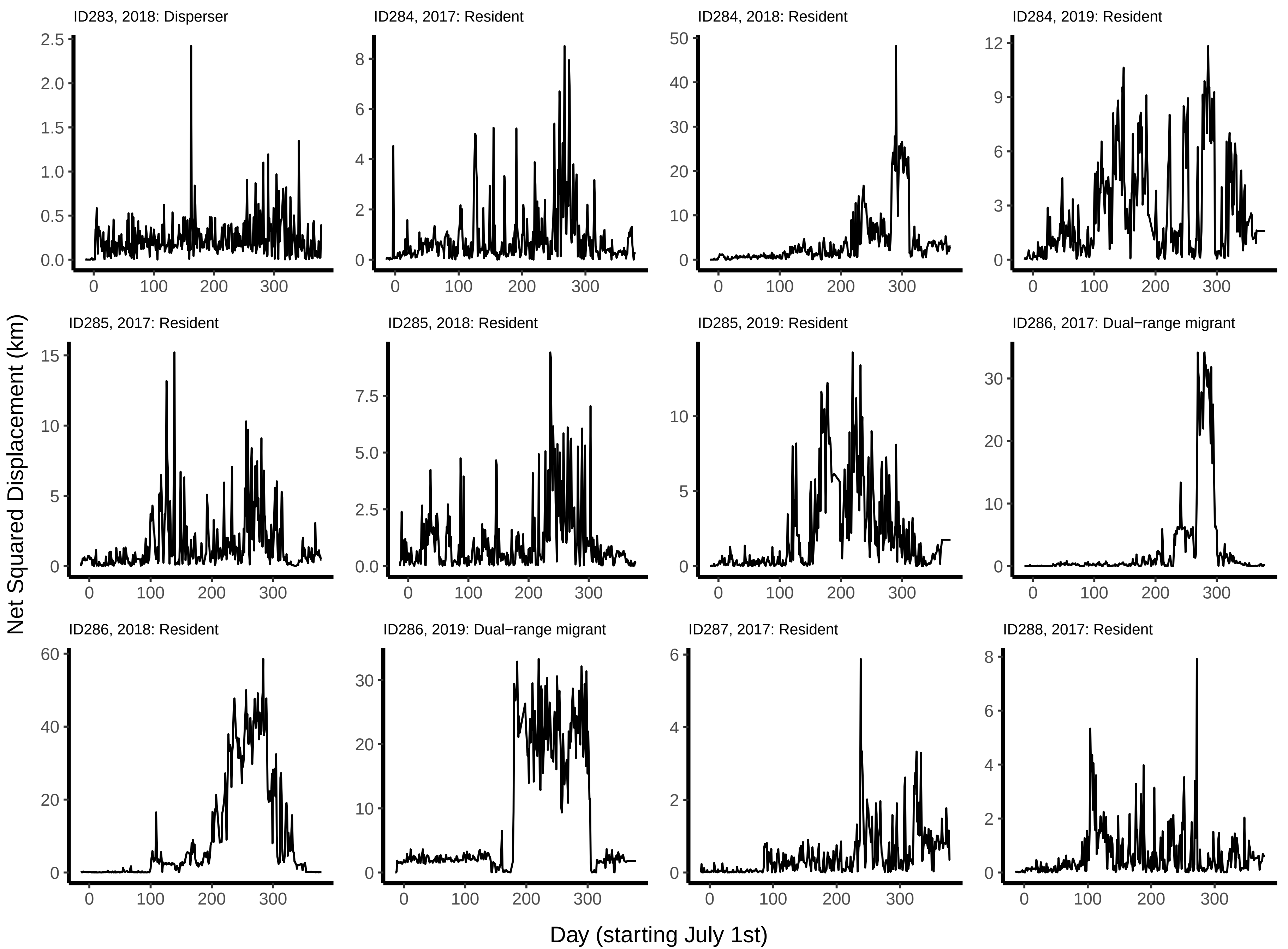

ID283, 2018: Disperser
ID284, 2017: Resident
ID284, 2018: Resident
ID284, 2019: Resident
ID285, 2017: Resident
ID285, 2018: Resident
ID285, 2019: Resident
ID286, 2017: Dual−range migrant
ID286, 2018: Resident
ID286, 2019: Dual−range migrant
ID287, 2017: Resident
ID288, 2017: Resident
Net Squared Displacement (km)
Day (starting July 1st)

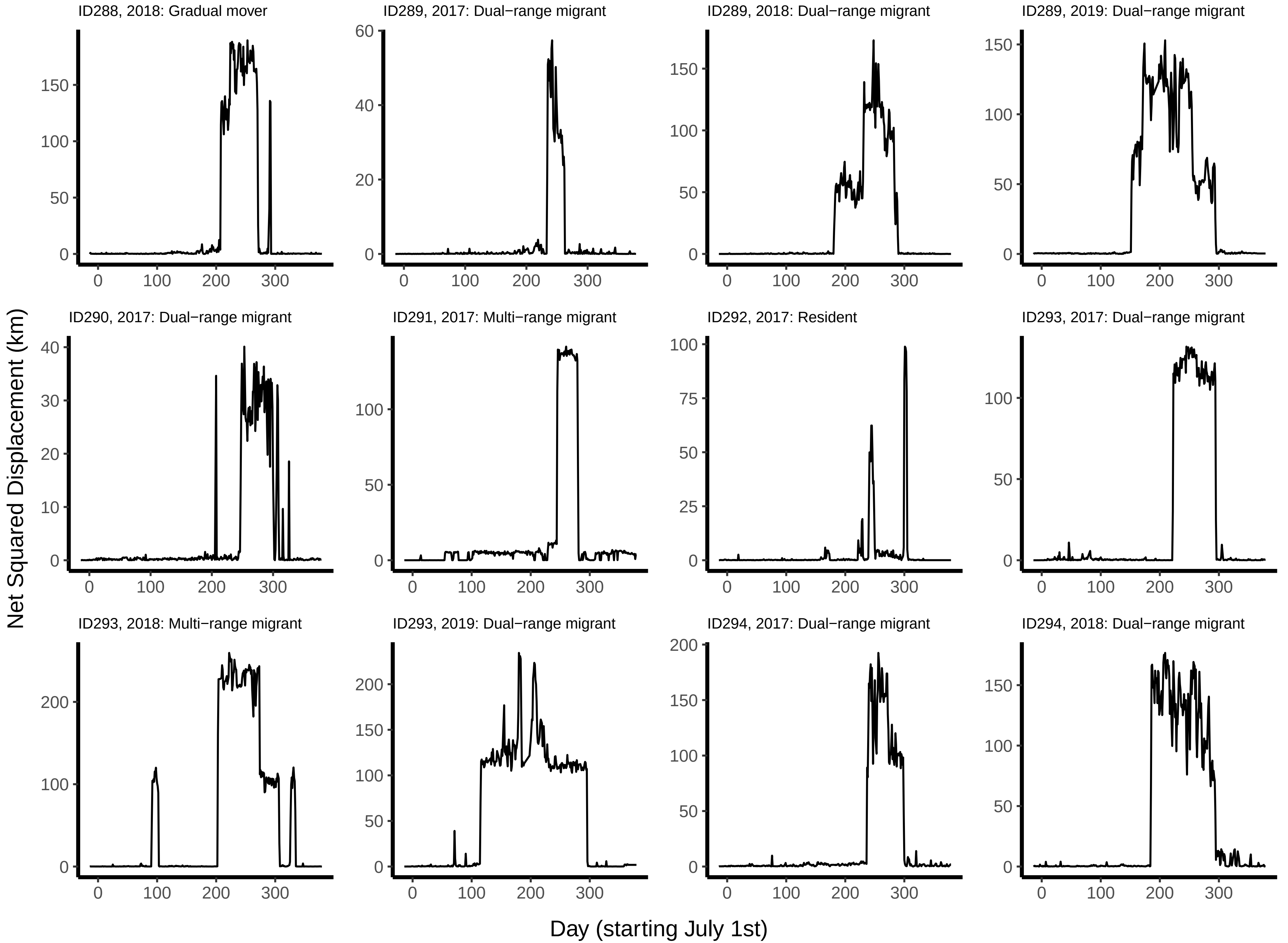

ID288, 2018: Gradual mover
ID289, 2017: Dual−range migrant
ID289, 2018: Dual−range migrant
ID289, 2019: Dual−range migrant
ID290, 2017: Dual−range migrant
ID291, 2017: Multi−range migrant
ID292, 2017: Resident
ID293, 2017: Dual−range migrant
ID293, 2018: Multi−range migrant
ID293, 2019: Dual−range migrant
ID294, 2017: Dual−range migrant
ID294, 2018: Dual−range migrant
Net Squared Displacement (km)
Day (starting July 1st)

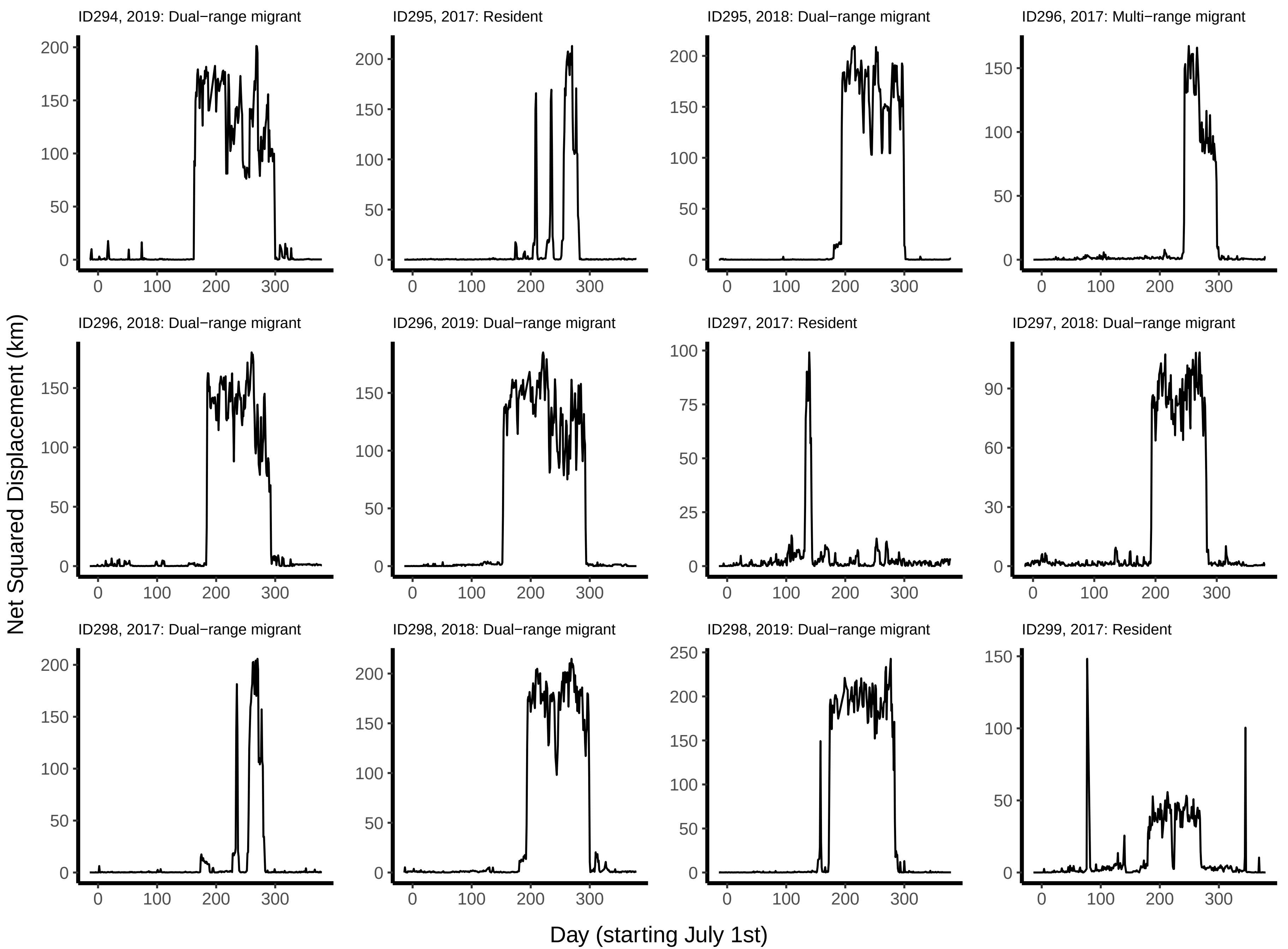

ID294, 2019: Dual−range migrant
ID295, 2017: Resident
ID295, 2018: Dual−range migrant
ID296, 2017: Multi−range migrant
ID296, 2018: Dual−range migrant
ID296, 2019: Dual−range migrant
ID297, 2017: Resident
ID297, 2018: Dual−range migrant
ID298, 2017: Dual−range migrant
ID298, 2018: Dual−range migrant
ID298, 2019: Dual−range migrant
ID299, 2017: Resident
Net Squared Displacement (km)
Day (starting July 1st)

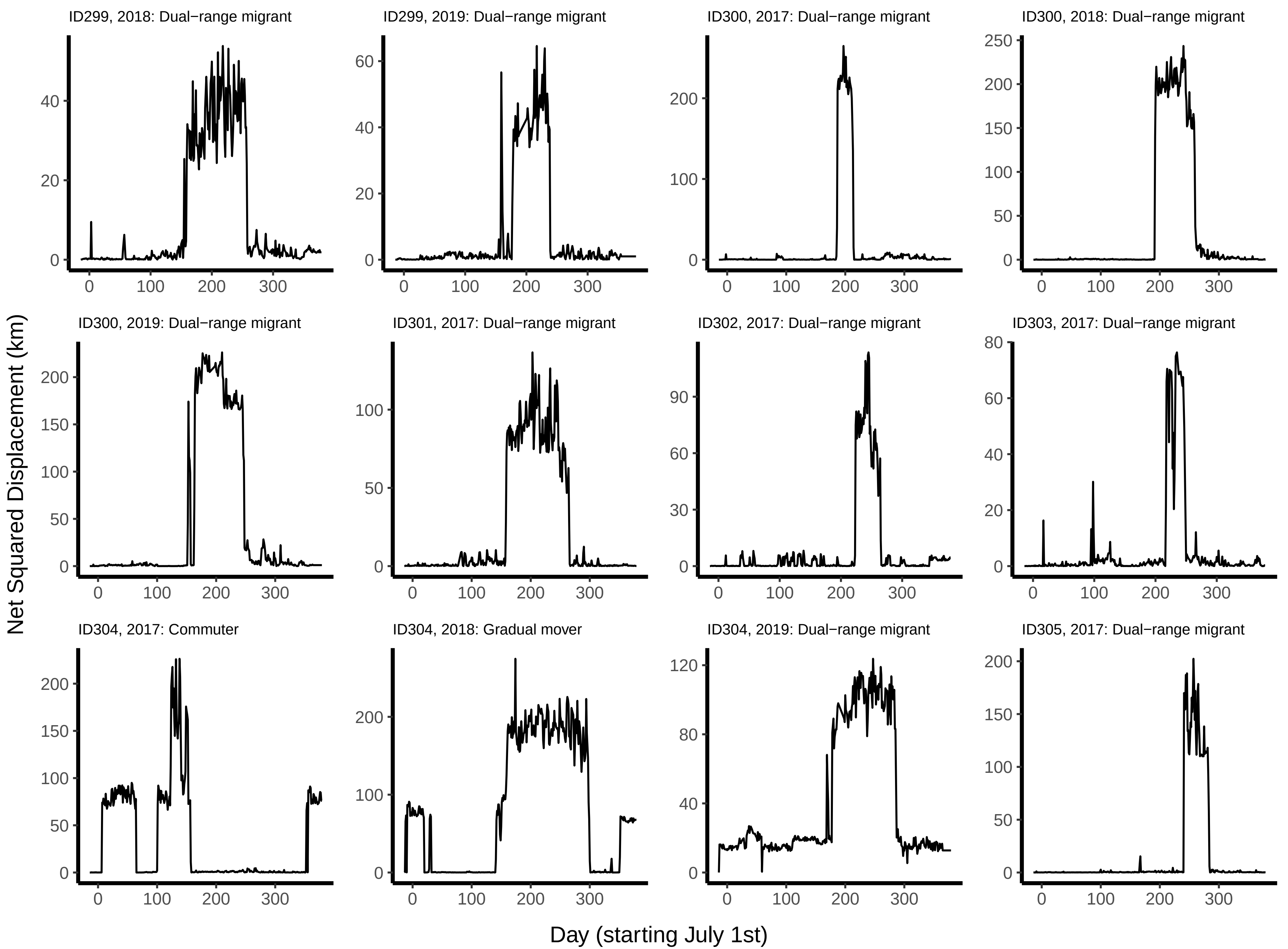
ID299, 2018: Dual−range migrant
ID299, 2019: Dual−range migrant
ID300, 2017: Dual−range migrant
ID300, 2018: Dual−range migrant
ID300, 2019: Dual−range migrant
ID301, 2017: Dual−range migrant
ID302, 2017: Dual−range migrant
ID303, 2017: Dual−range migrant
ID304, 2017: Commuter
ID304, 2018: Gradual mover
ID304, 2019: Dual−range migrant
ID305, 2017: Dual−range migrant
Net Squared Displacement (km)
Day (starting July 1st)

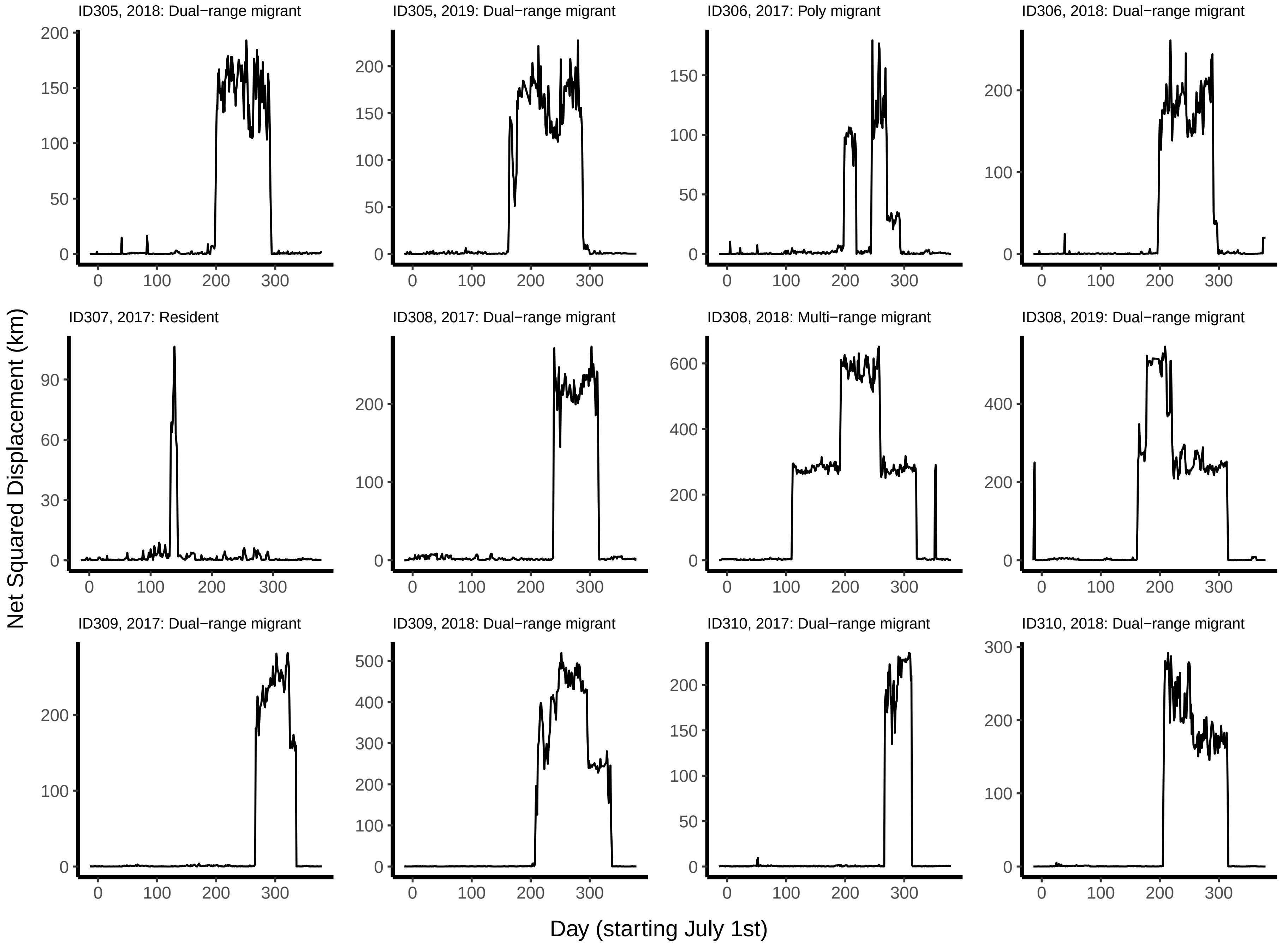
ID305, 2018: Dual−range migrant
ID305, 2019: Dual−range migrant
ID306, 2017: Poly migrant
ID306, 2018: Dual−range migrant
ID307, 2017: Resident
ID308, 2017: Dual−range migrant
ID308, 2018: Multi−range migrant
ID308, 2019: Dual−range migrant
ID309, 2017: Dual−range migrant
ID309, 2018: Dual−range migrant
ID310, 2017: Dual−range migrant
ID310, 2018: Dual−range migrant
Net Squared Displacement (km)
Day (starting July 1st)

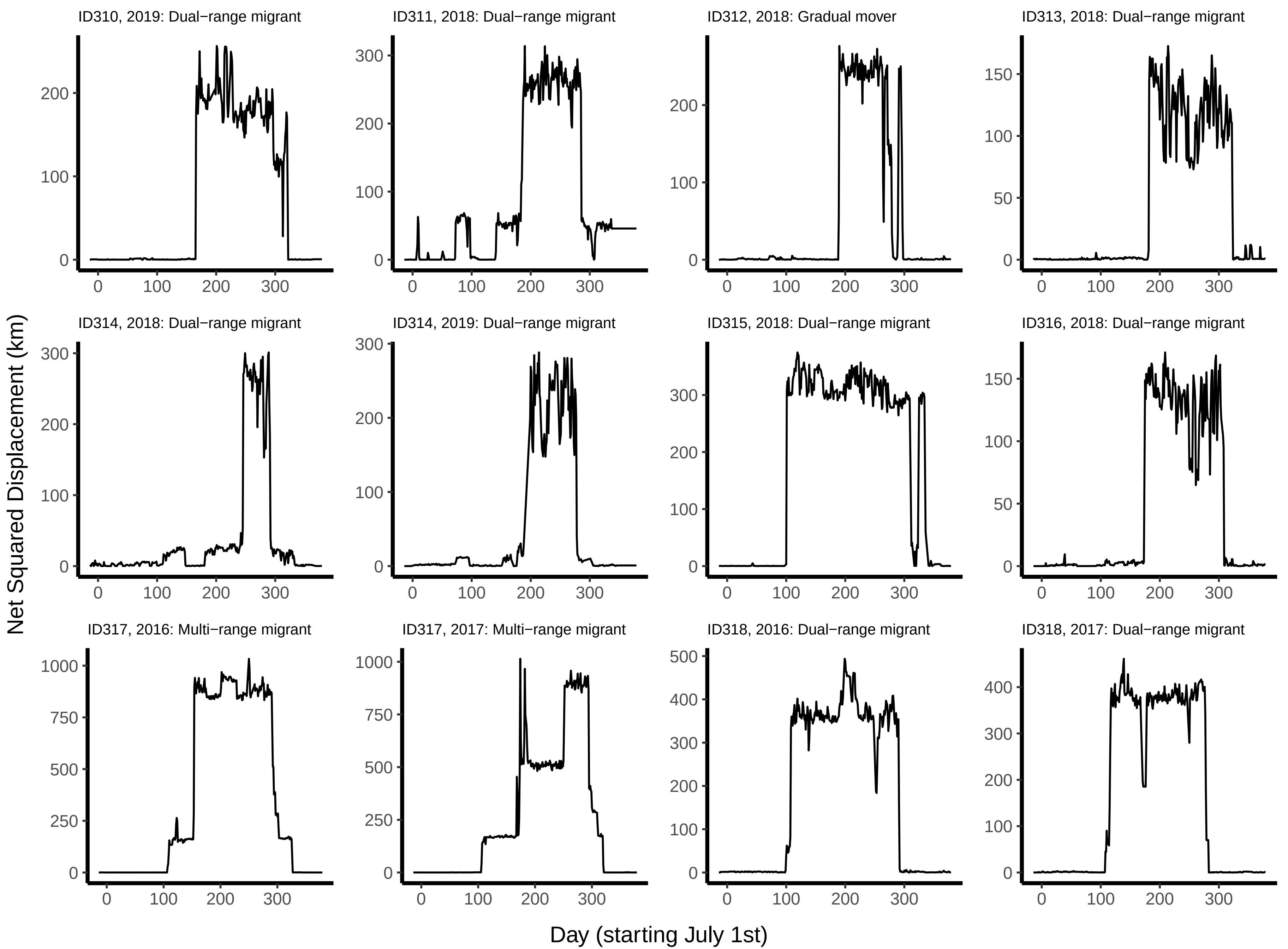
ID310, 2019: Dual−range migrant
ID311, 2018: Dual−range migrant
ID312, 2018: Gradual mover
ID313, 2018: Dual−range migrant
ID314, 2018: Dual−range migrant
ID314, 2019: Dual−range migrant
ID315, 2018: Dual−range migrant
ID316, 2018: Dual−range migrant
ID317, 2016: Multi−range migrant
ID317, 2017: Multi−range migrant
ID318, 2016: Dual−range migrant
ID318, 2017: Dual−range migrant
Net Squared Displacement (km)
Day (starting July 1st)

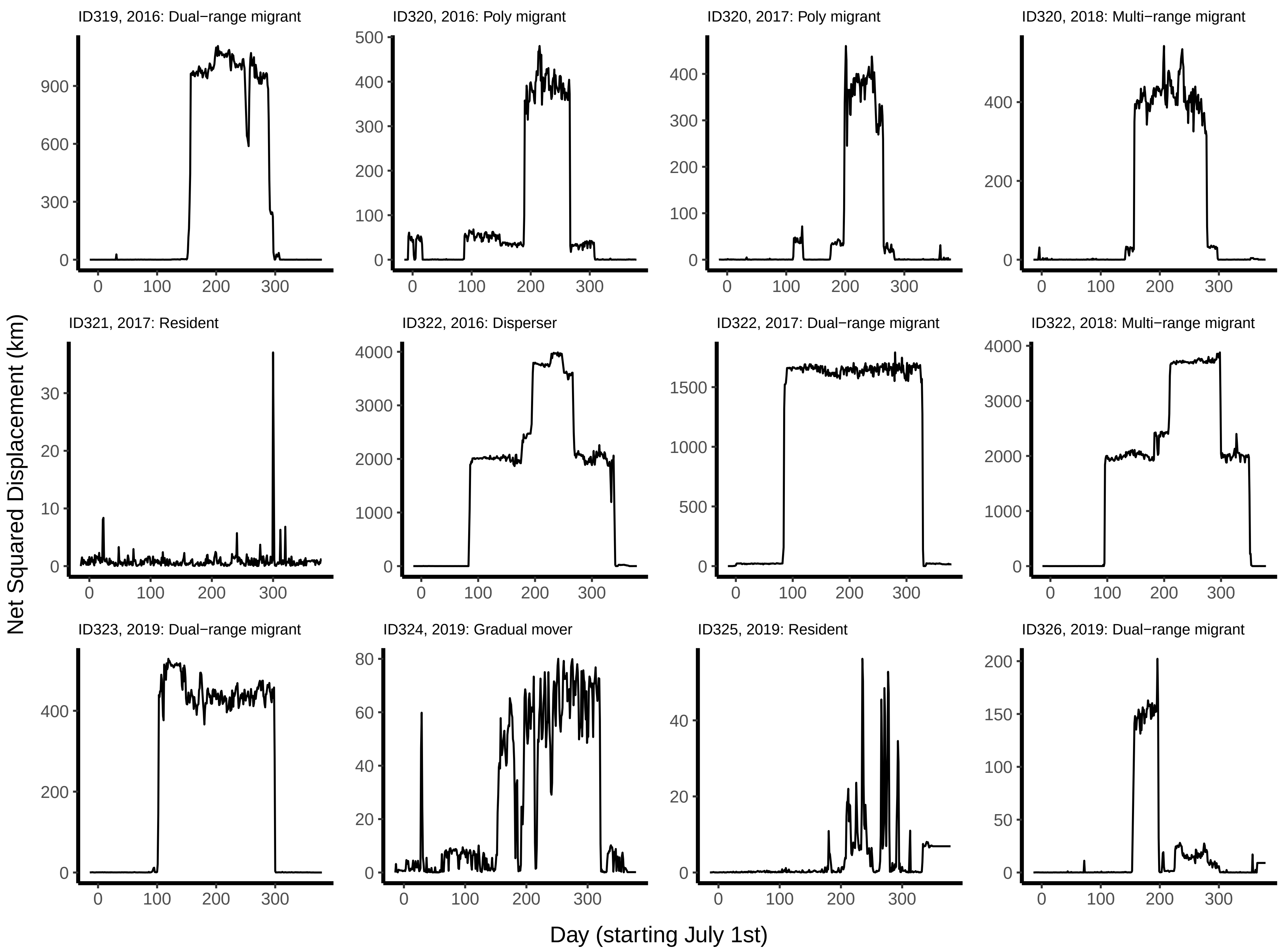
ID319, 2016: Dual−range migrant
ID320, 2016: Poly migrant
ID320, 2017: Poly migrant
ID320, 2018: Multi−range migrant
ID321, 2017: Resident
ID322, 2016: Disperser
ID322, 2017: Dual−range migrant
ID322, 2018: Multi−range migrant
ID323, 2019: Dual−range migrant
ID324, 2019: Gradual mover
ID325, 2019: Resident
ID326, 2019: Dual−range migrant
Net Squared Displacement (km)
Day (starting July 1st)

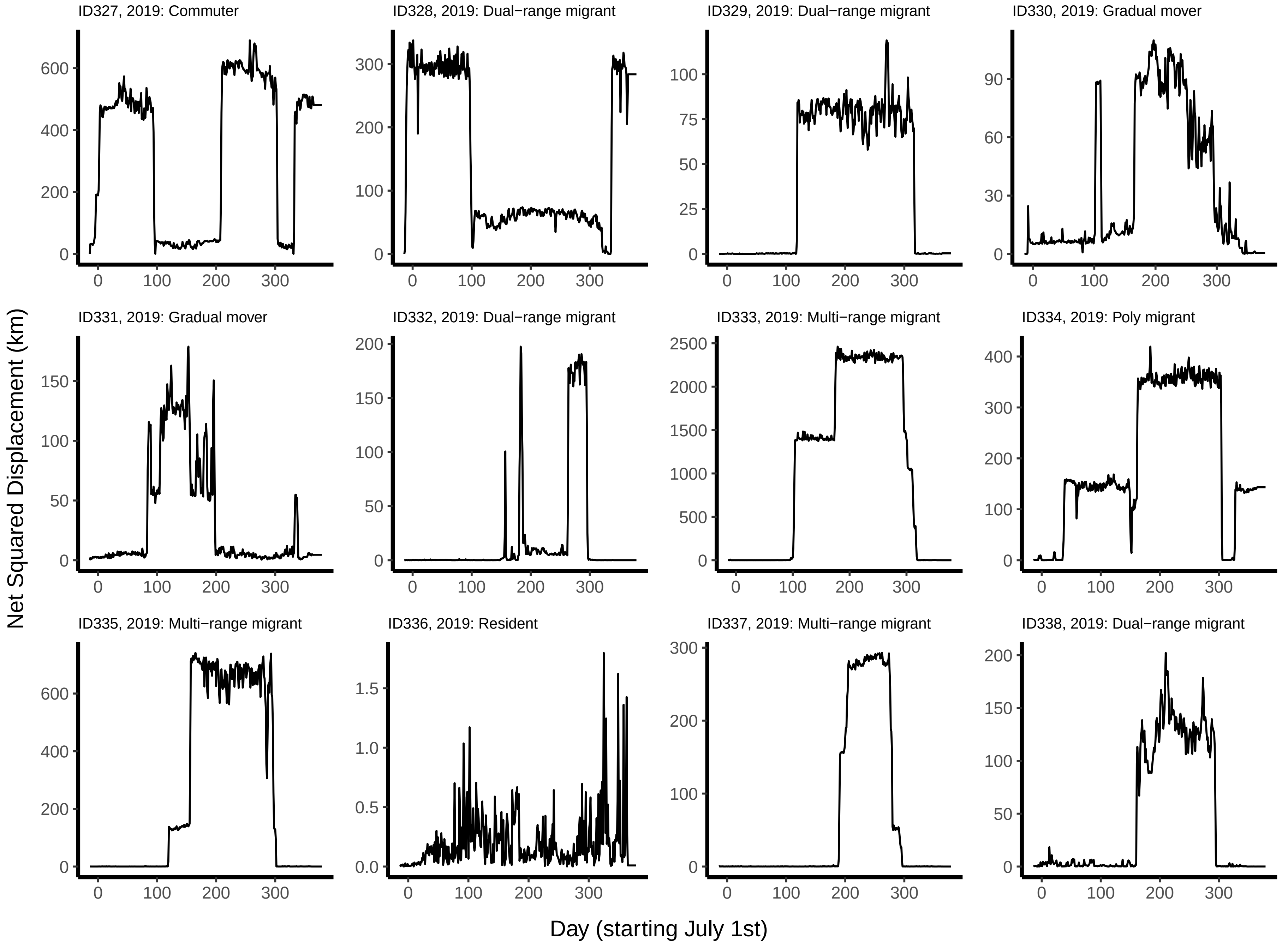

ID327, 2019: Commuter
ID328, 2019: Dual−range migrant
ID329, 2019: Dual−range migrant
ID330, 2019: Gradual mover
ID331, 2019: Gradual mover
ID332, 2019: Dual−range migrant
ID333, 2019: Multi−range migrant
ID334, 2019: Poly migrant
ID335, 2019: Multi−range migrant
ID336, 2019: Resident
ID337, 2019: Multi−range migrant
ID338, 2019: Dual−range migrant
Net Squared Displacement (km)
Day (starting July 1st)

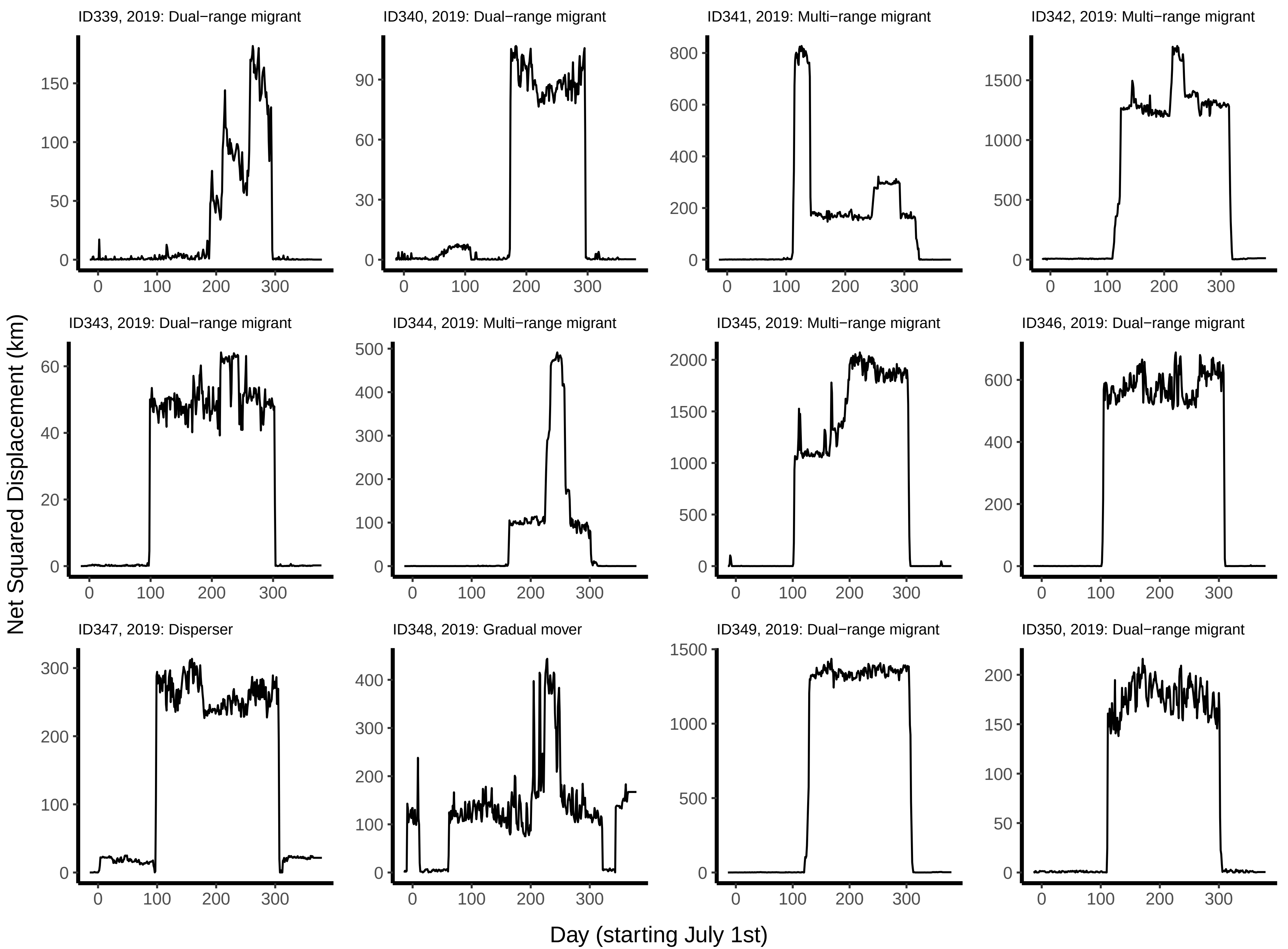

ID339, 2019: Dual−range migrant
ID340, 2019: Dual−range migrant
ID341, 2019: Multi−range migrant
ID342, 2019: Multi−range migrant
ID343, 2019: Dual−range migrant
ID344, 2019: Multi−range migrant
ID345, 2019: Multi−range migrant
ID346, 2019: Dual−range migrant
ID347, 2019: Disperser
ID348, 2019: Gradual mover
ID349, 2019: Dual−range migrant
ID350, 2019: Dual−range migrant
Net Squared Displacement (km)
Day (starting July 1st)

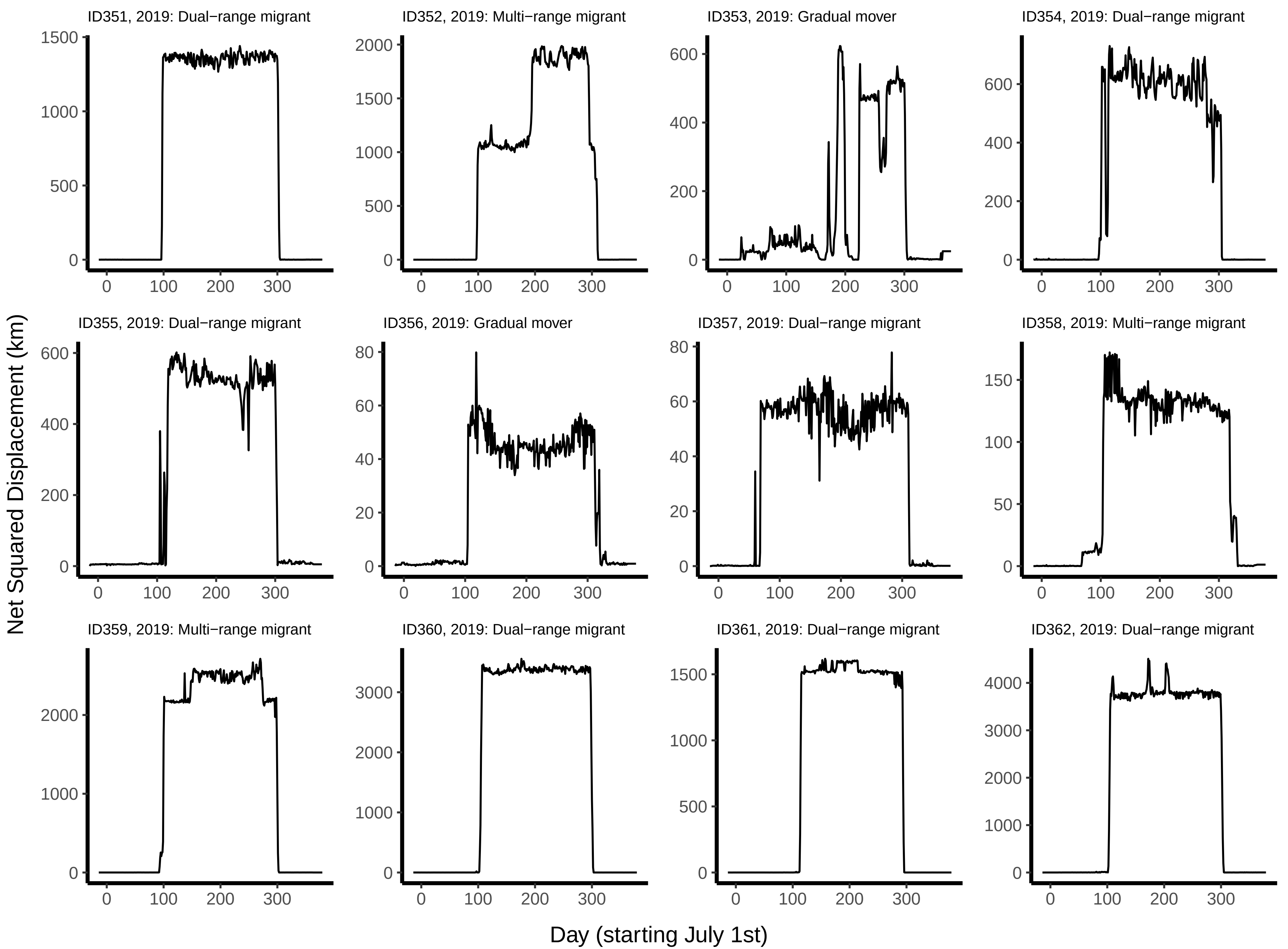
ID351, 2019: Dual−range migrant
ID352, 2019: Multi−range migrant
ID353, 2019: Gradual mover
ID354, 2019: Dual−range migrant
ID355, 2019: Dual−range migrant
ID356, 2019: Gradual mover
ID357, 2019: Dual−range migrant
ID358, 2019: Multi−range migrant
ID359, 2019: Multi−range migrant
ID360, 2019: Dual−range migrant
ID361, 2019: Dual−range migrant
ID362, 2019: Dual−range migrant
Net Squared Displacement (km)
Day (starting July 1st)

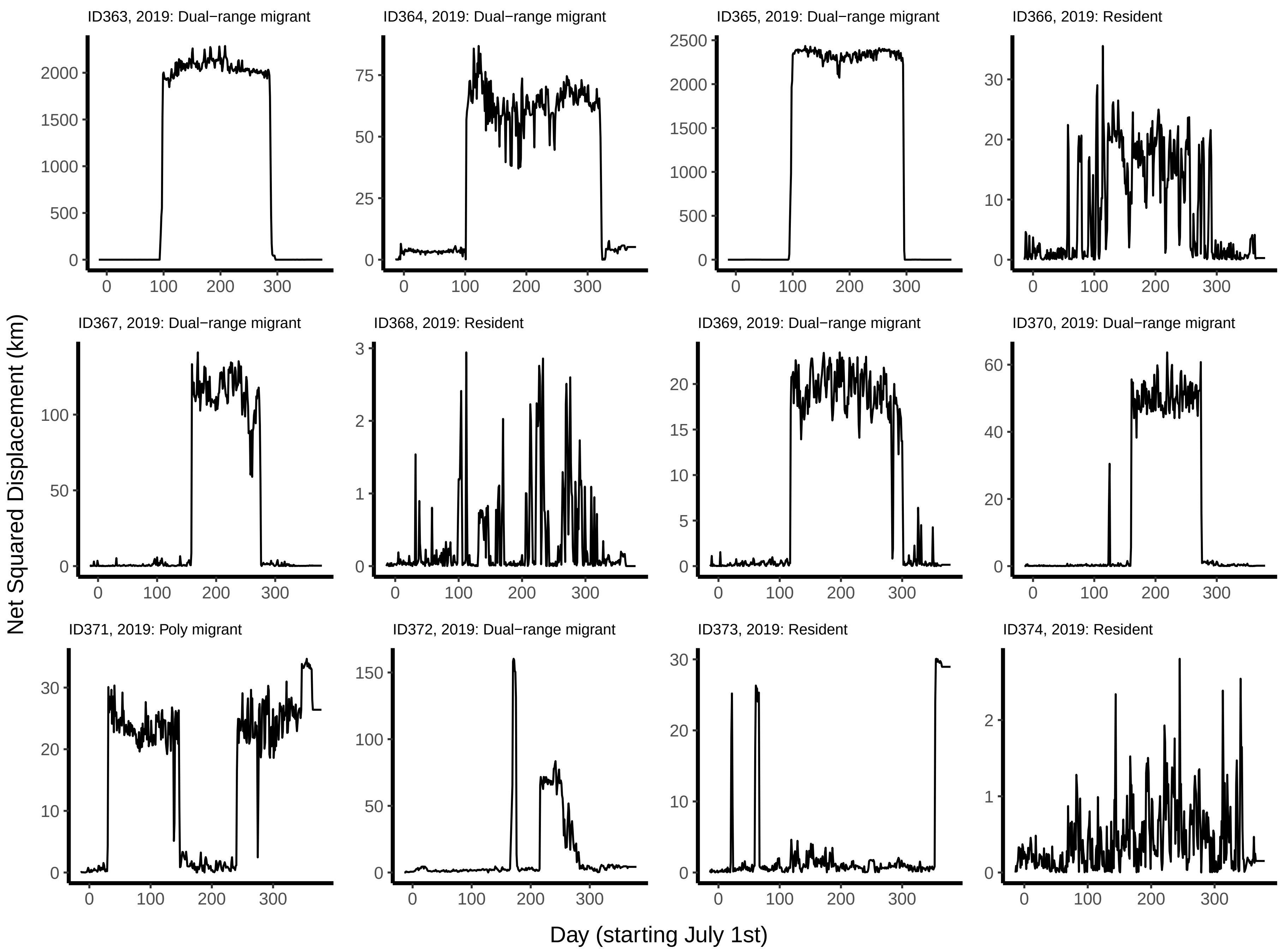

ID363, 2019: Dual−range migrant
ID364, 2019: Dual−range migrant
ID365, 2019: Dual−range migrant
ID366, 2019: Resident
ID367, 2019: Dual−range migrant
ID368, 2019: Resident
ID369, 2019: Dual−range migrant
ID370, 2019: Dual−range migrant
ID371, 2019: Poly migrant
ID372, 2019: Dual−range migrant
ID373, 2019: Resident
ID374, 2019: Resident
Net Squared Displacement (km)
Day (starting July 1st)

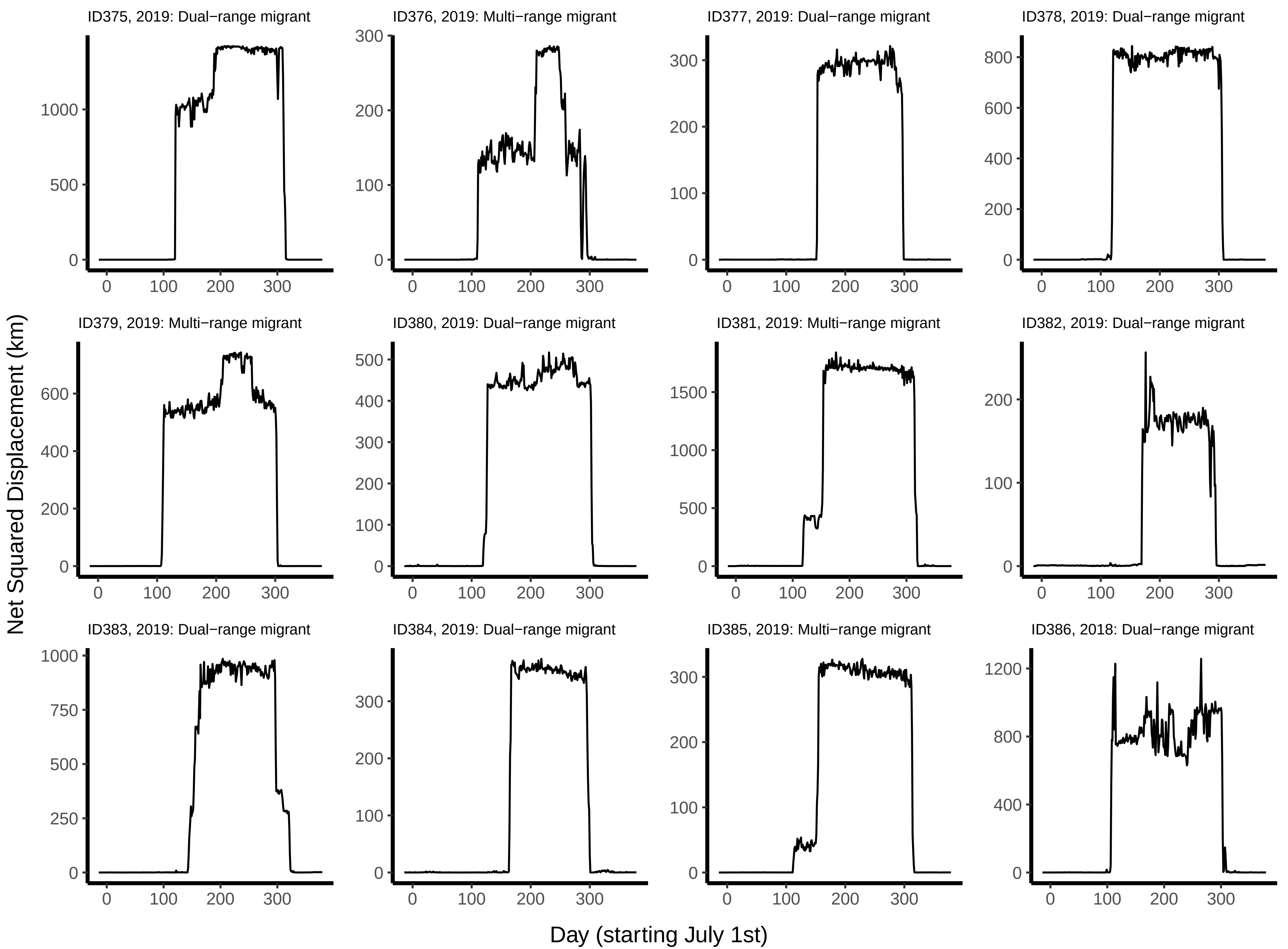

ID375, 2019: Dual−range migrant
ID376, 2019: Multi−range migrant
ID377, 2019: Dual−range migrant
ID378, 2019: Dual−range migrant
ID379, 2019: Multi−range migrant
ID380, 2019: Dual−range migrant
ID381, 2019: Multi−range migrant
ID382, 2019: Dual−range migrant
ID383, 2019: Dual−range migrant
ID384, 2019: Dual−range migrant
ID385, 2019: Multi−range migrant
ID386, 2018: Dual−range migrant
Net Squared Displacement (km)
Day (starting July 1st)

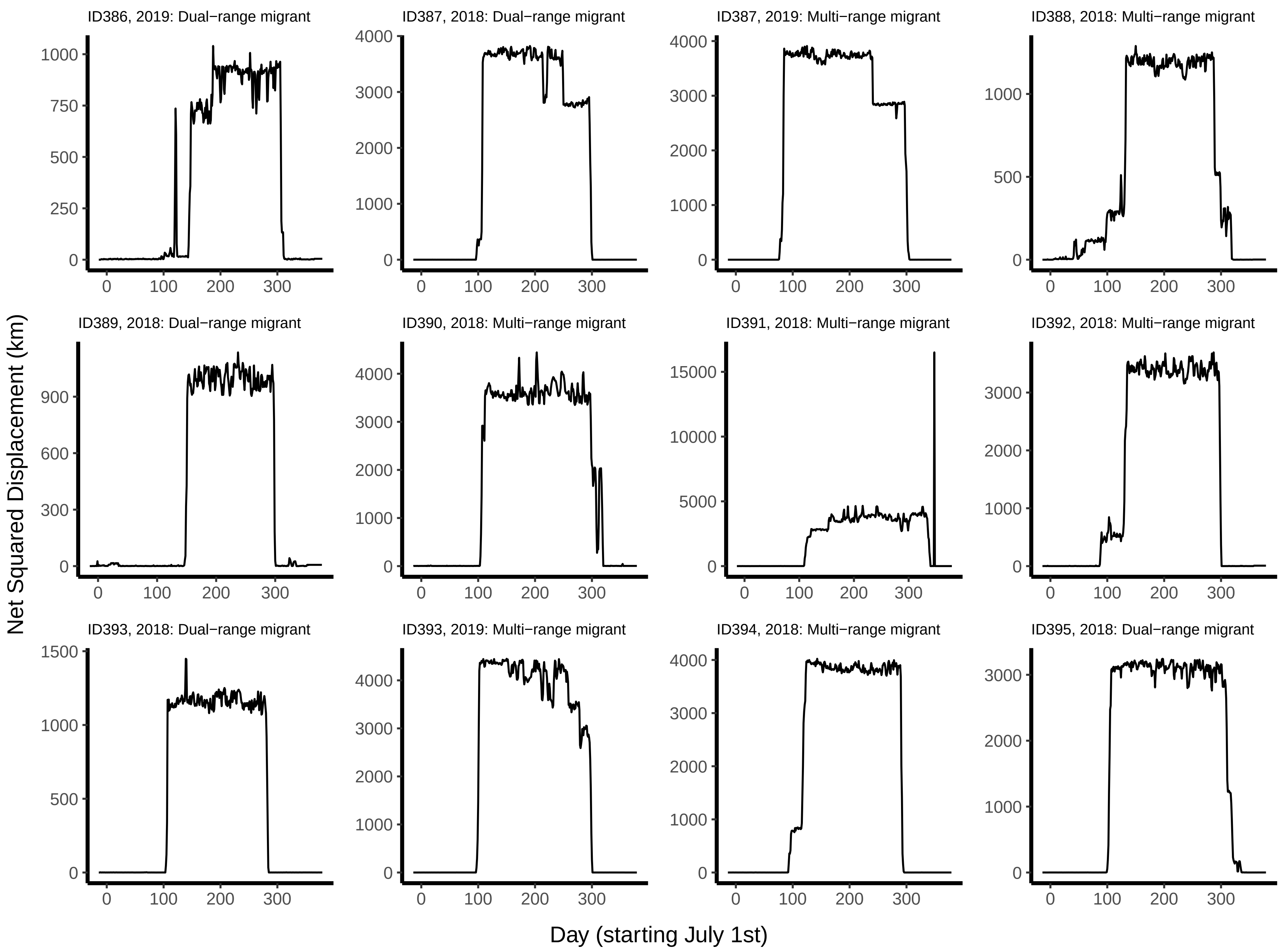

ID386, 2019: Dual−range migrant
ID387, 2018: Dual−range migrant
ID387, 2019: Multi−range migrant
ID388, 2018: Multi−range migrant
ID389, 2018: Dual−range migrant
ID390, 2018: Multi−range migrant
ID391, 2018: Multi−range migrant
ID392, 2018: Multi−range migrant
ID393, 2018: Dual−range migrant
ID393, 2019: Multi−range migrant
ID394, 2018: Multi−range migrant
ID395, 2018: Dual−range migrant
Net Squared Displacement (km)
Day (starting July 1st)

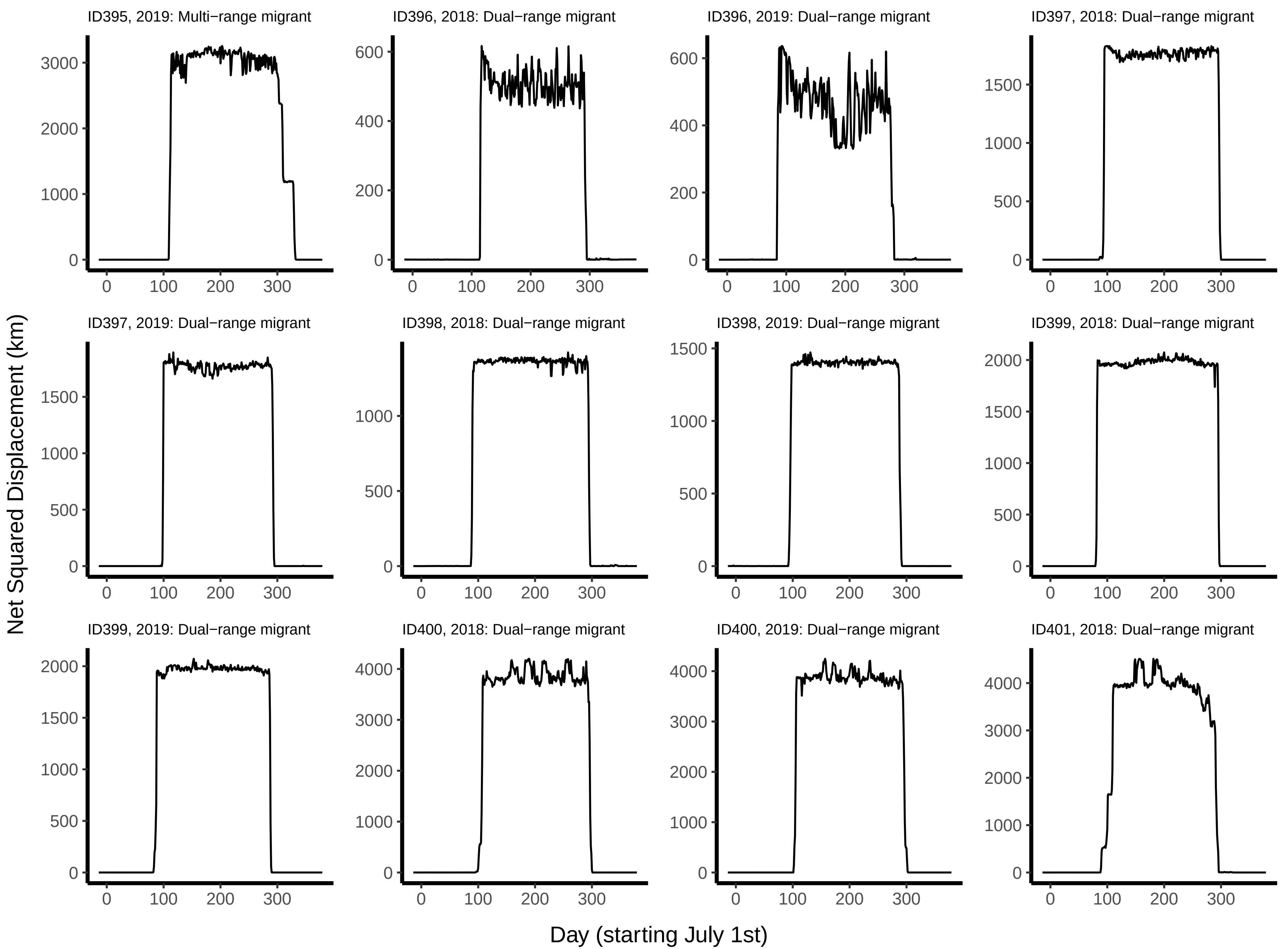
ID395, 2019: Multi−range migrant
ID396, 2018: Dual−range migrant
ID396, 2019: Dual−range migrant
ID397, 2018: Dual−range migrant
ID397, 2019: Dual−range migrant
ID398, 2018: Dual−range migrant
ID398, 2019: Dual−range migrant
ID399, 2018: Dual−range migrant
ID399, 2019: Dual−range migrant
ID400, 2018: Dual−range migrant
ID400, 2019: Dual−range migrant
ID401, 2018: Dual−range migrant
Net Squared Displacement (km)
Day (starting July 1st)

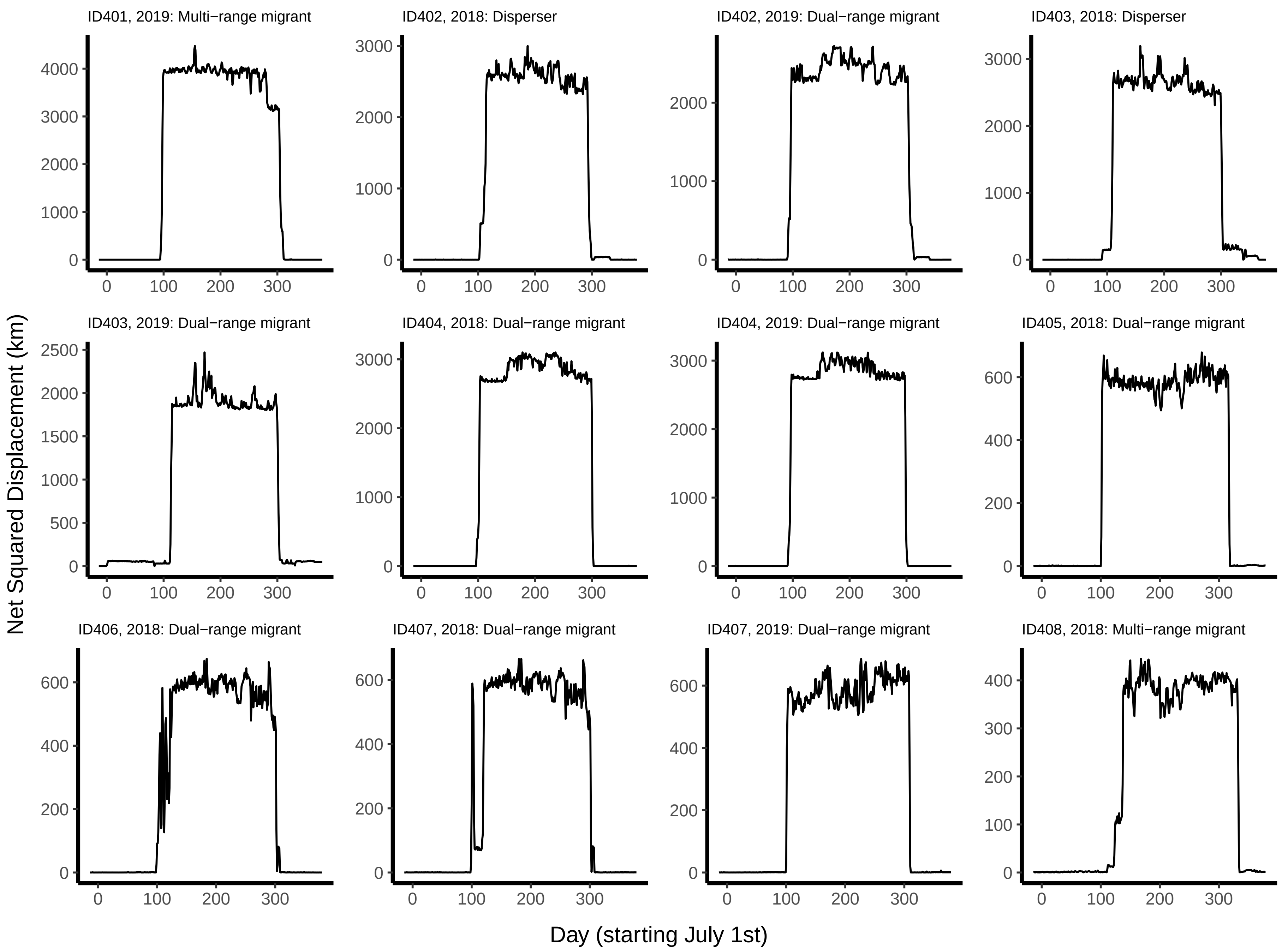

ID401, 2019: Multi−range migrant
ID402, 2018: Disperser
ID402, 2019: Dual−range migrant
ID403, 2018: Disperser
ID403, 2019: Dual−range migrant
ID404, 2018: Dual−range migrant
ID404, 2019: Dual−range migrant
ID405, 2018: Dual−range migrant
ID406, 2018: Dual−range migrant
ID407, 2018: Dual−range migrant
ID407, 2019: Dual−range migrant
ID408, 2018: Multi−range migrant
Net Squared Displacement (km)
Day (starting July 1st)

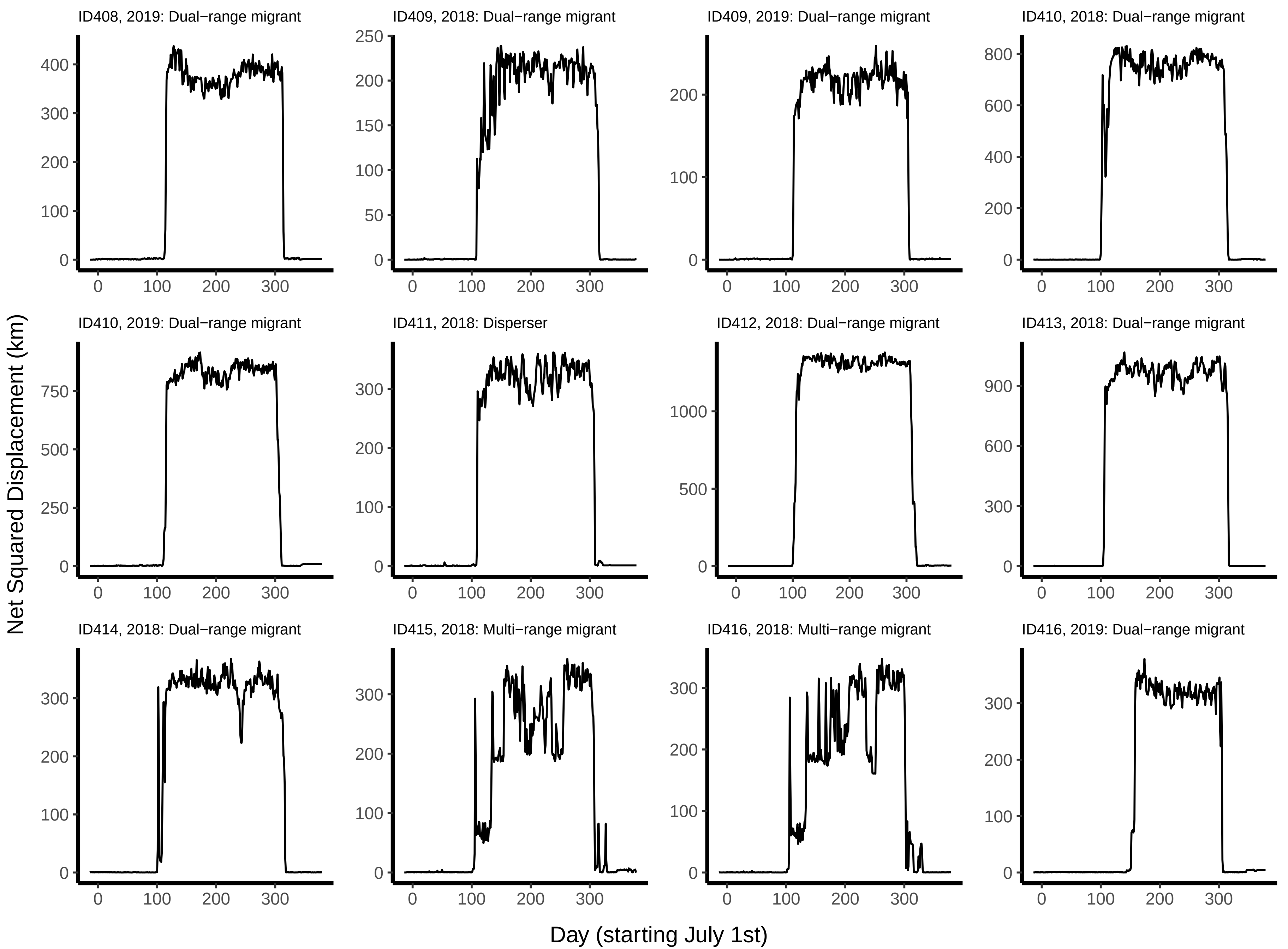

ID408, 2019: Dual−range migrant
ID409, 2018: Dual−range migrant
ID409, 2019: Dual−range migrant
ID410, 2018: Dual−range migrant
ID410, 2019: Dual−range migrant
ID411, 2018: Disperser
ID412, 2018: Dual−range migrant
ID413, 2018: Dual−range migrant
ID414, 2018: Dual−range migrant
ID415, 2018: Multi−range migrant
ID416, 2018: Multi−range migrant
ID416, 2019: Dual−range migrant
Net Squared Displacement (km)
Day (starting July 1st)

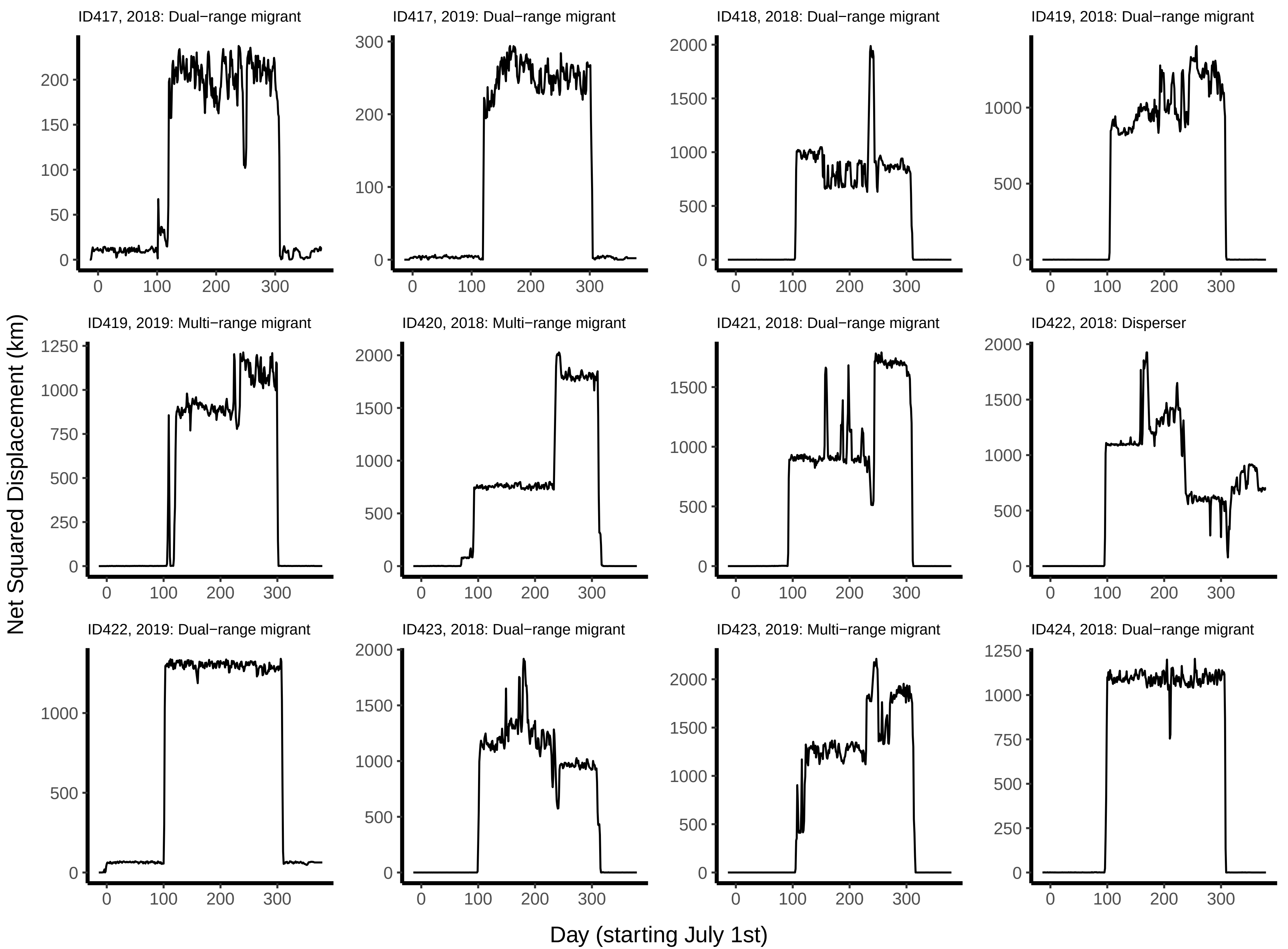

ID417, 2018: Dual−range migrant
ID417, 2019: Dual−range migrant
ID418, 2018: Dual−range migrant
ID419, 2018: Dual−range migrant
ID419, 2019: Multi−range migrant
ID420, 2018: Multi−range migrant
ID421, 2018: Dual−range migrant
ID422, 2018: Disperser
ID422, 2019: Dual−range migrant
ID423, 2018: Dual−range migrant
ID423, 2019: Multi−range migrant
ID424, 2018: Dual−range migrant
Net Squared Displacement (km)
Day (starting July 1st)

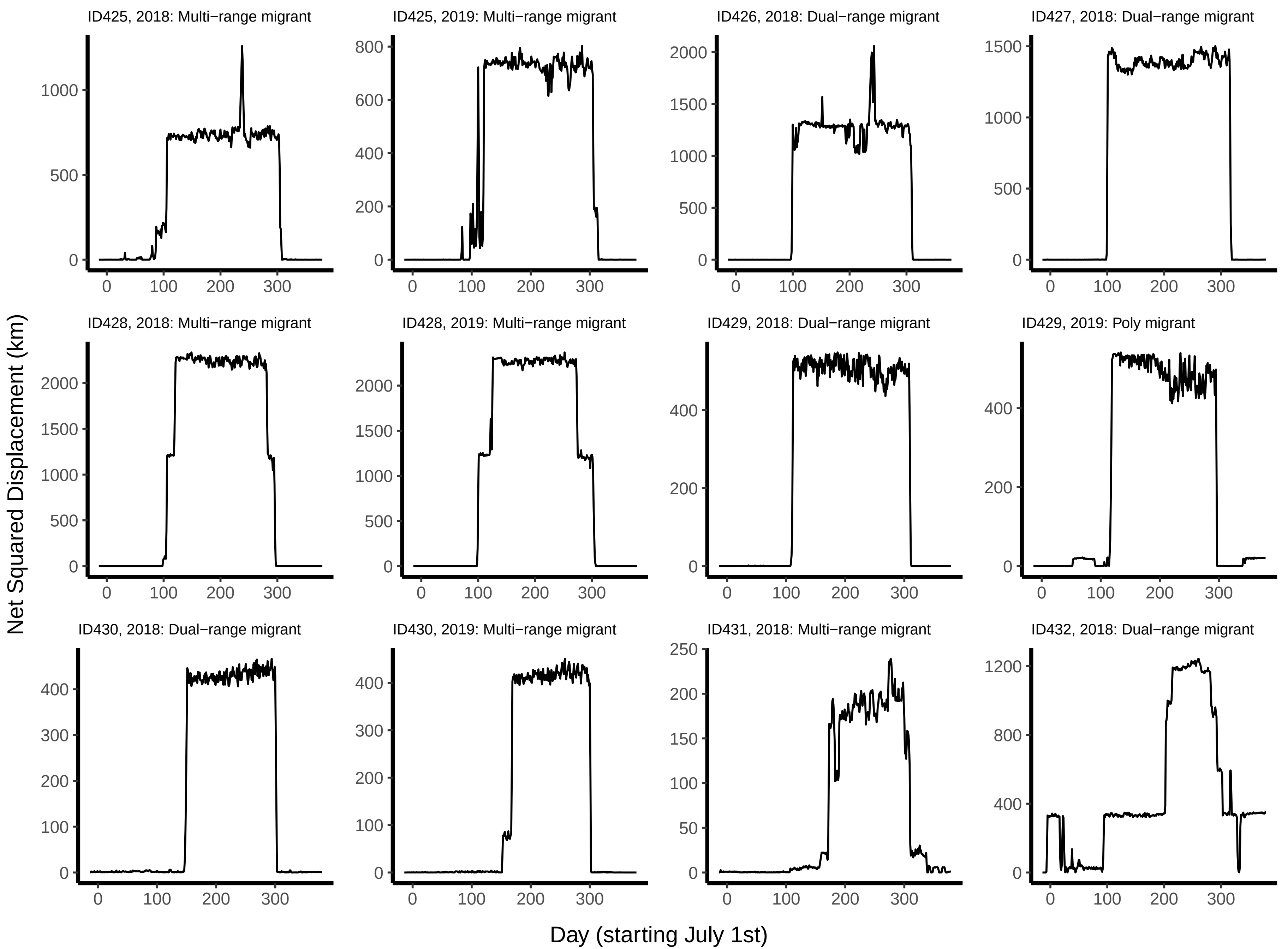

ID425, 2018: Multi−range migrant
ID425, 2019: Multi−range migrant
ID426, 2018: Dual−range migrant
ID427, 2018: Dual−range migrant
ID428, 2018: Multi−range migrant
ID428, 2019: Multi−range migrant
ID429, 2018: Dual−range migrant
ID429, 2019: Poly migrant
ID430, 2018: Dual−range migrant
ID430, 2019: Multi−range migrant
ID431, 2018: Multi−range migrant
ID432, 2018: Dual−range migrant
Net Squared Displacement (km)
Day (starting July 1st)

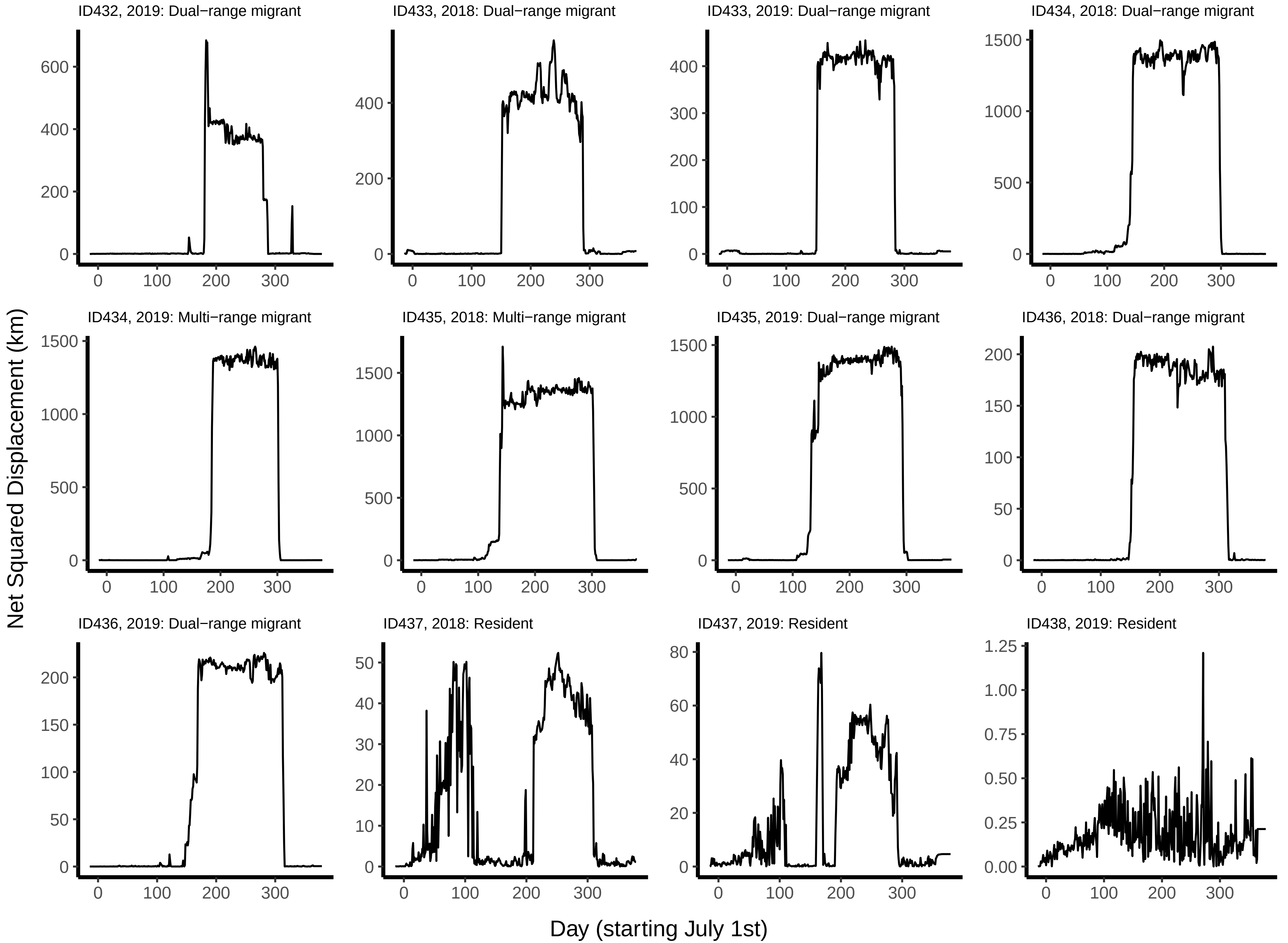

ID432, 2019: Dual−range migrant
ID433, 2018: Dual−range migrant
ID433, 2019: Dual−range migrant
ID434, 2018: Dual−range migrant
ID434, 2019: Multi−range migrant
ID435, 2018: Multi−range migrant
ID435, 2019: Dual−range migrant
ID436, 2018: Dual−range migrant
ID436, 2019: Dual−range migrant
ID437, 2018: Resident
ID437, 2019: Resident
ID438, 2019: Resident
Net Squared Displacement (km)
Day (starting July 1st)

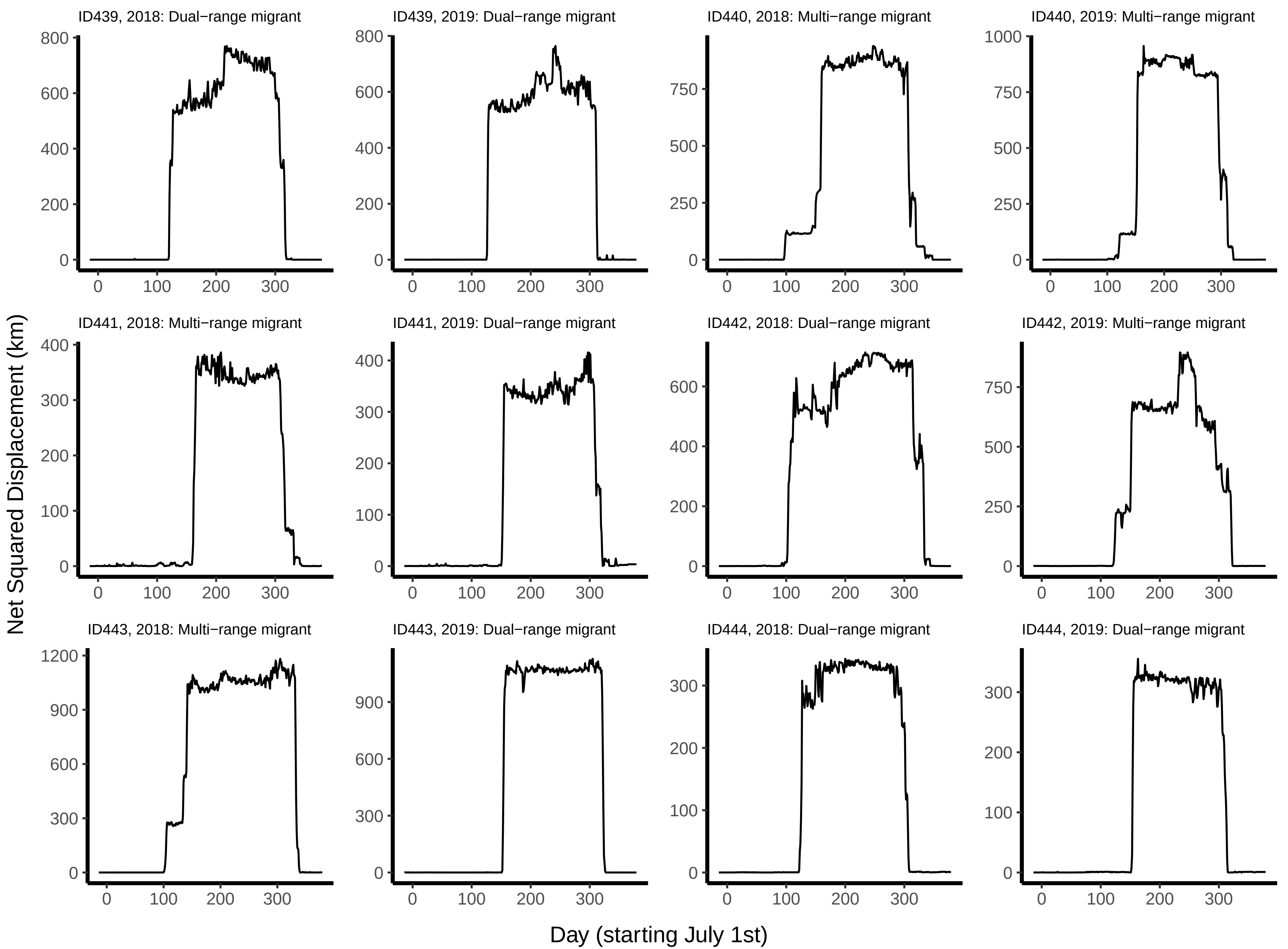

ID439, 2018: Dual−range migrant
ID439, 2019: Dual−range migrant
ID440, 2018: Multi−range migrant
ID440, 2019: Multi−range migrant
ID441, 2018: Multi−range migrant
ID441, 2019: Dual−range migrant
ID442, 2018: Dual−range migrant
ID442, 2019: Multi−range migrant
ID443, 2018: Multi−range migrant
ID443, 2019: Dual−range migrant
ID444, 2018: Dual−range migrant
ID444, 2019: Dual−range migrant
Net Squared Displacement (km)
Day (starting July 1st)

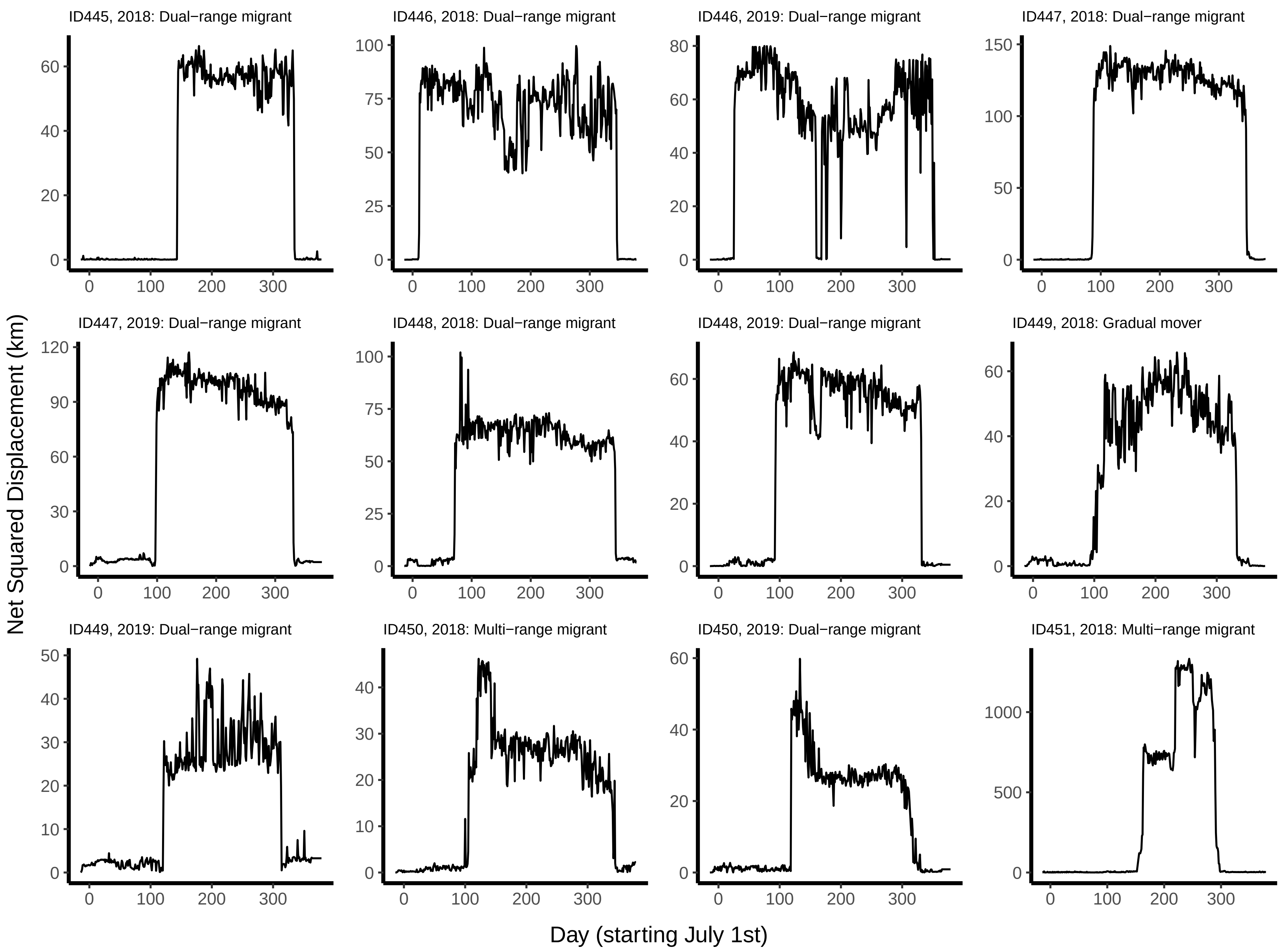

ID445, 2018: Dual−range migrant
ID446, 2018: Dual−range migrant
ID446, 2019: Dual−range migrant
ID447, 2018: Dual−range migrant
ID447, 2019: Dual−range migrant
ID448, 2018: Dual−range migrant
ID448, 2019: Dual−range migrant
ID449, 2018: Gradual mover
ID449, 2019: Dual−range migrant
ID450, 2018: Multi−range migrant
ID450, 2019: Dual−range migrant
ID451, 2018: Multi−range migrant
Net Squared Displacement (km)
Day (starting July 1st)

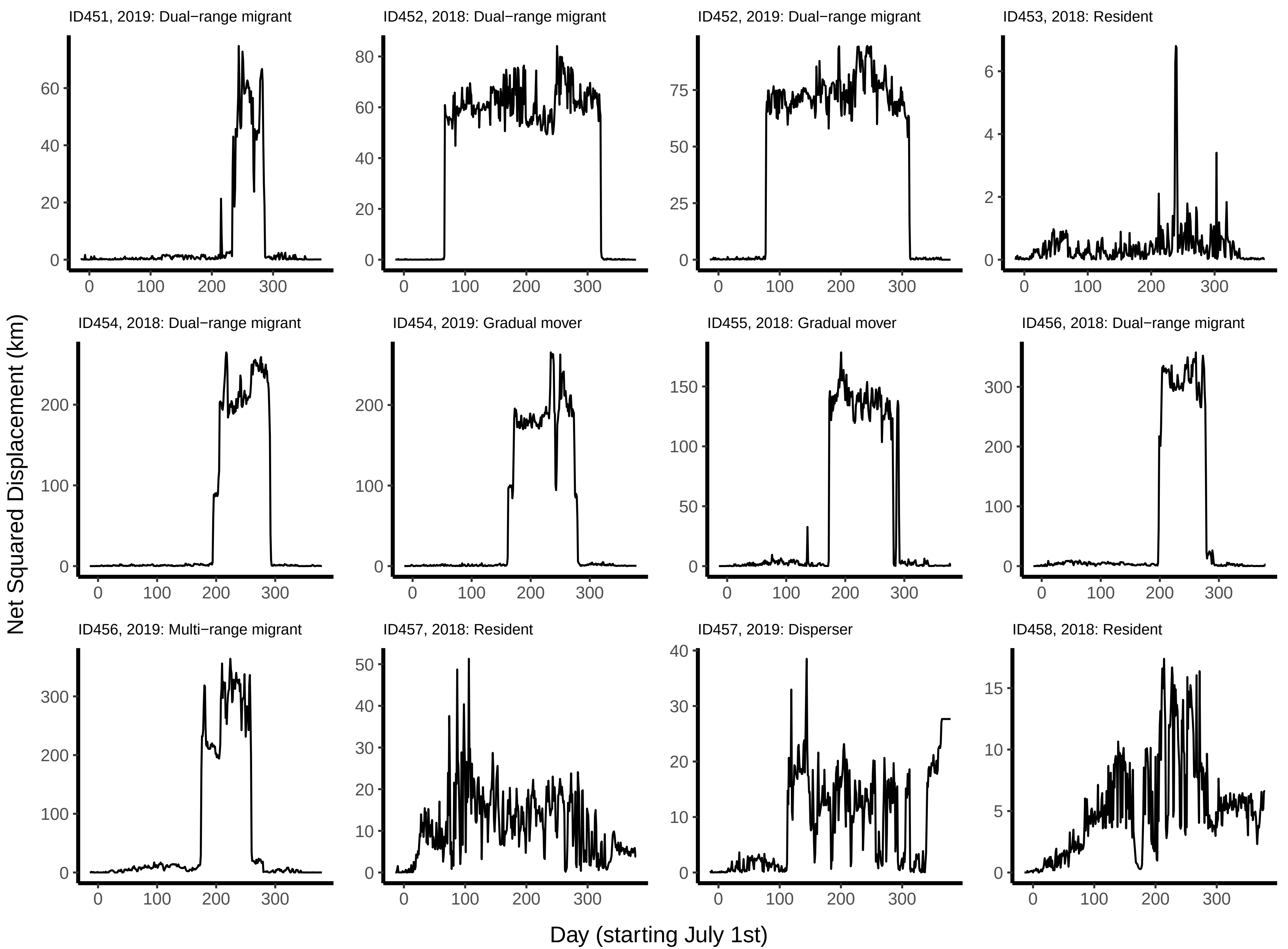

ID451, 2019: Dual−range migrant
ID452, 2018: Dual−range migrant
ID452, 2019: Dual−range migrant
ID453, 2018: Resident
ID454, 2018: Dual−range migrant
ID454, 2019: Gradual mover
ID455, 2018: Gradual mover
ID456, 2018: Dual−range migrant
ID456, 2019: Multi−range migrant
ID457, 2018: Resident
ID457, 2019: Disperser
ID458, 2018: Resident
Net Squared Displacement (km)
Day (starting July 1st)

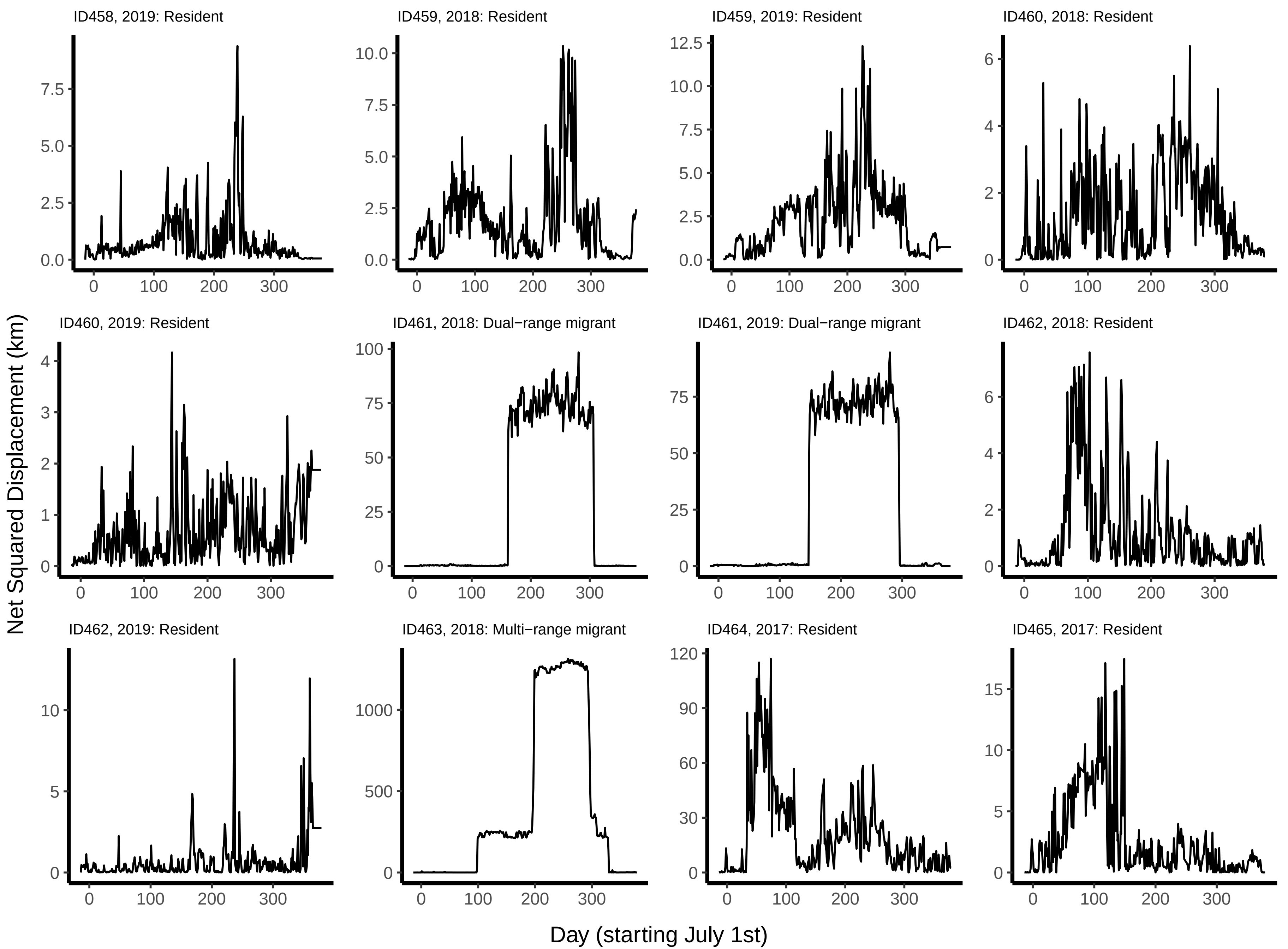
ID458, 2019: Resident
ID459, 2018: Resident
ID459, 2019: Resident
ID460, 2018: Resident
ID460, 2019: Resident
ID461, 2018: Dual−range migrant
ID461, 2019: Dual−range migrant
ID462, 2018: Resident
ID462, 2019: Resident
ID463, 2018: Multi−range migrant
ID464, 2017: Resident
ID465, 2017: Resident
Net Squared Displacement (km)
Day (starting July 1st)

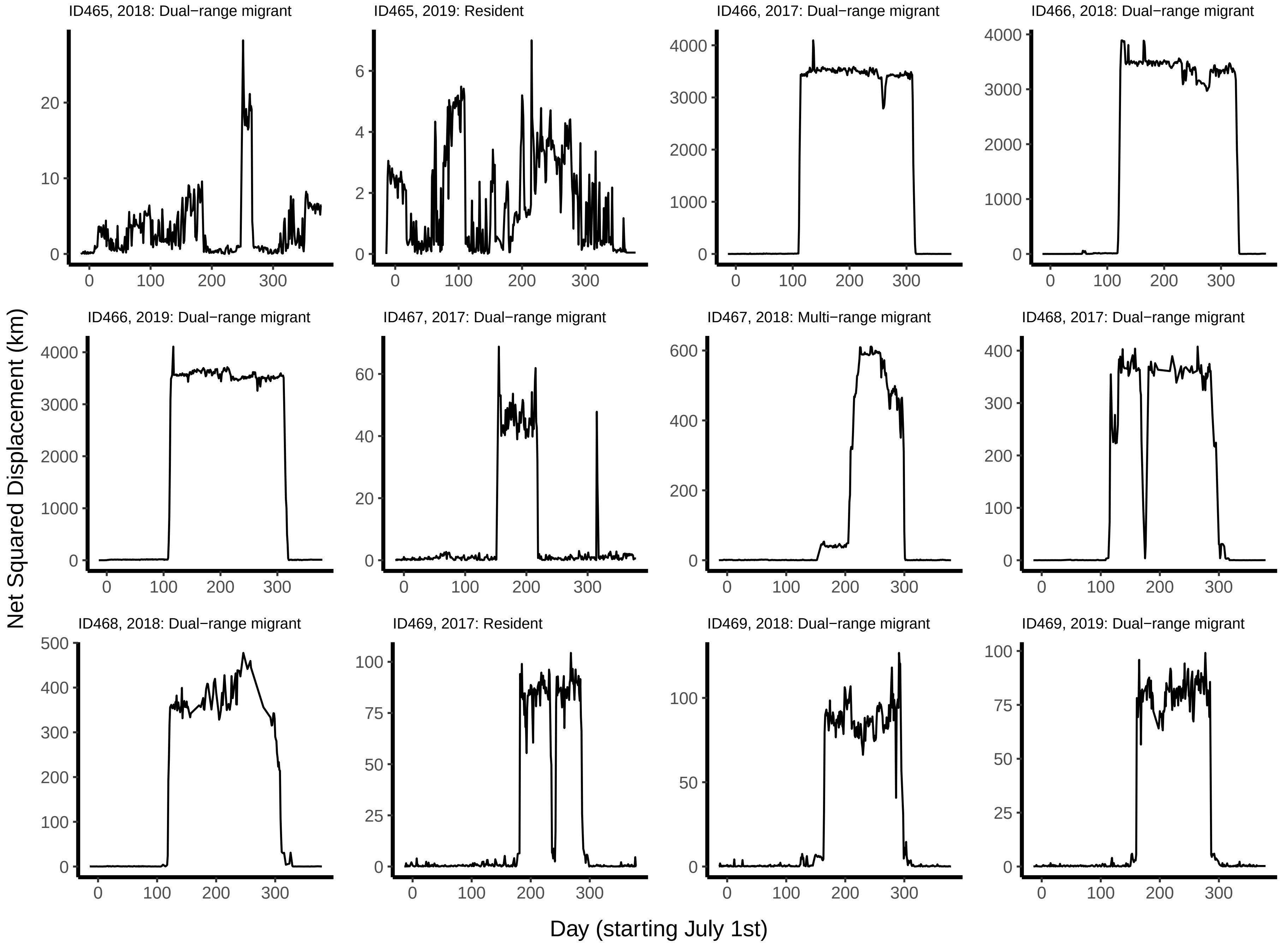
ID465, 2018: Dual−range migrant
ID465, 2019: Resident
ID466, 2017: Dual−range migrant
ID466, 2018: Dual−range migrant
ID466, 2019: Dual−range migrant
ID467, 2017: Dual−range migrant
ID467, 2018: Multi−range migrant
ID468, 2017: Dual−range migrant
ID468, 2018: Dual−range migrant
ID469, 2017: Resident
ID469, 2018: Dual−range migrant
ID469, 2019: Dual−range migrant
Net Squared Displacement (km)
Day (starting July 1st)

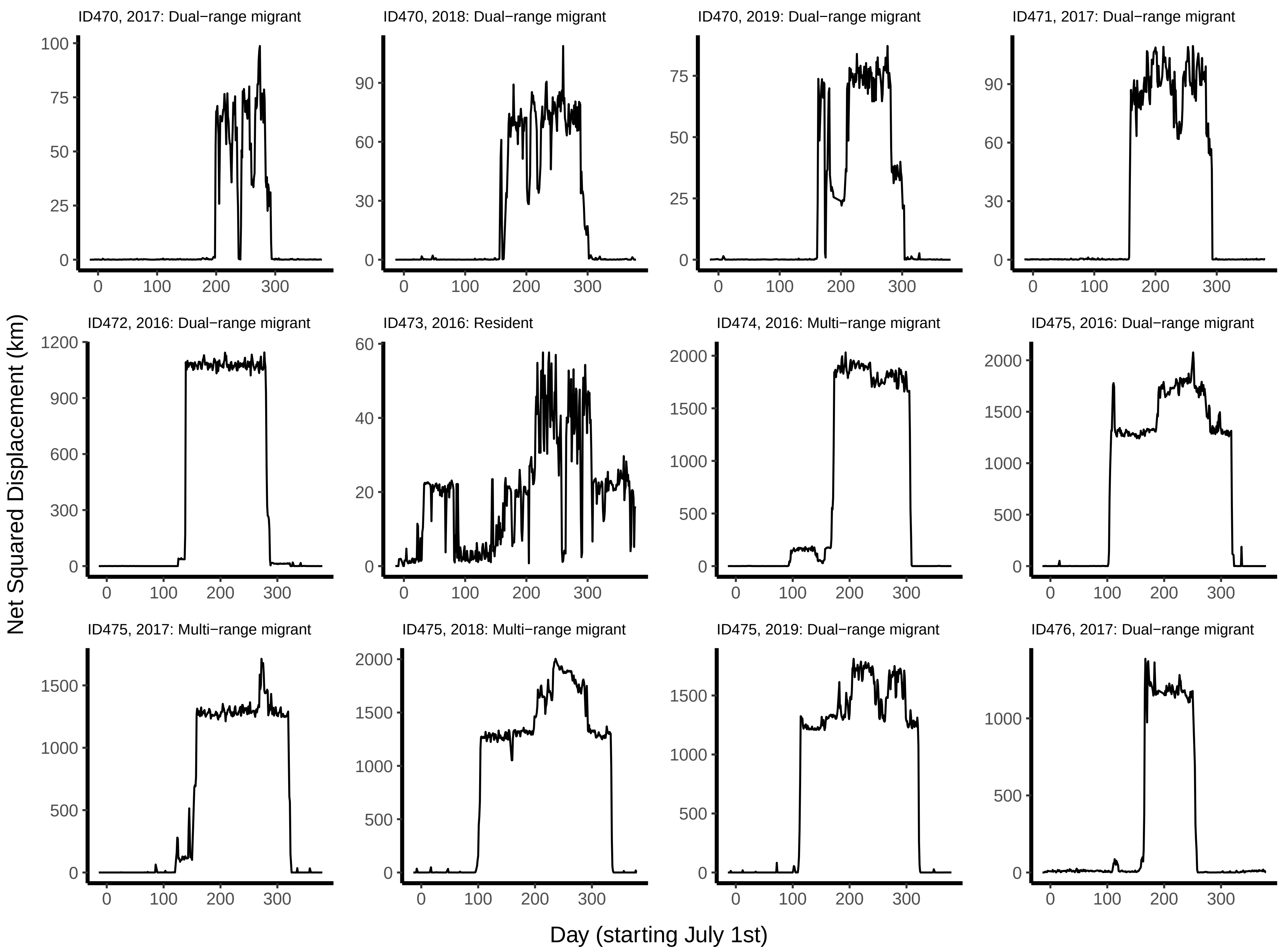
ID470, 2017: Dual−range migrant
ID470, 2018: Dual−range migrant
ID470, 2019: Dual−range migrant
ID471, 2017: Dual−range migrant
ID472, 2016: Dual−range migrant
ID473, 2016: Resident
ID474, 2016: Multi−range migrant
ID475, 2016: Dual−range migrant
ID475, 2017: Multi−range migrant
ID475, 2018: Multi−range migrant
ID475, 2019: Dual−range migrant
ID476, 2017: Dual−range migrant
Net Squared Displacement (km)
Day (starting July 1st)
0
100
200
300

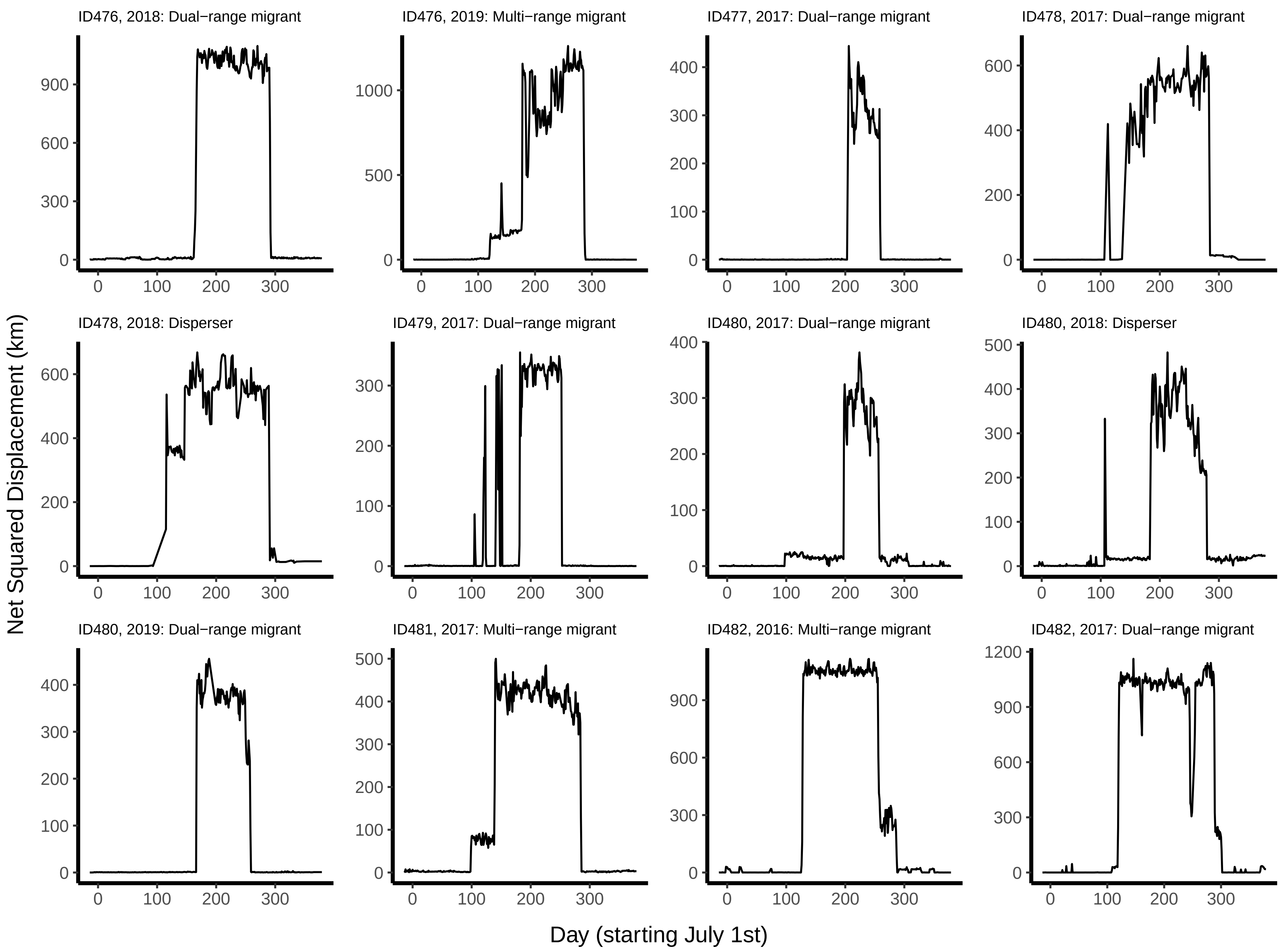
ID476, 2018: Dual−range migrant
ID476, 2019: Multi−range migrant
ID477, 2017: Dual−range migrant
ID478, 2017: Dual−range migrant
ID478, 2018: Disperser
ID479, 2017: Dual−range migrant
ID480, 2017: Dual−range migrant
ID480, 2018: Disperser
ID480, 2019: Dual−range migrant
ID481, 2017: Multi−range migrant
ID482, 2016: Multi−range migrant
ID482, 2017: Dual−range migrant
Net Squared Displacement (km)
Day (starting July 1st)

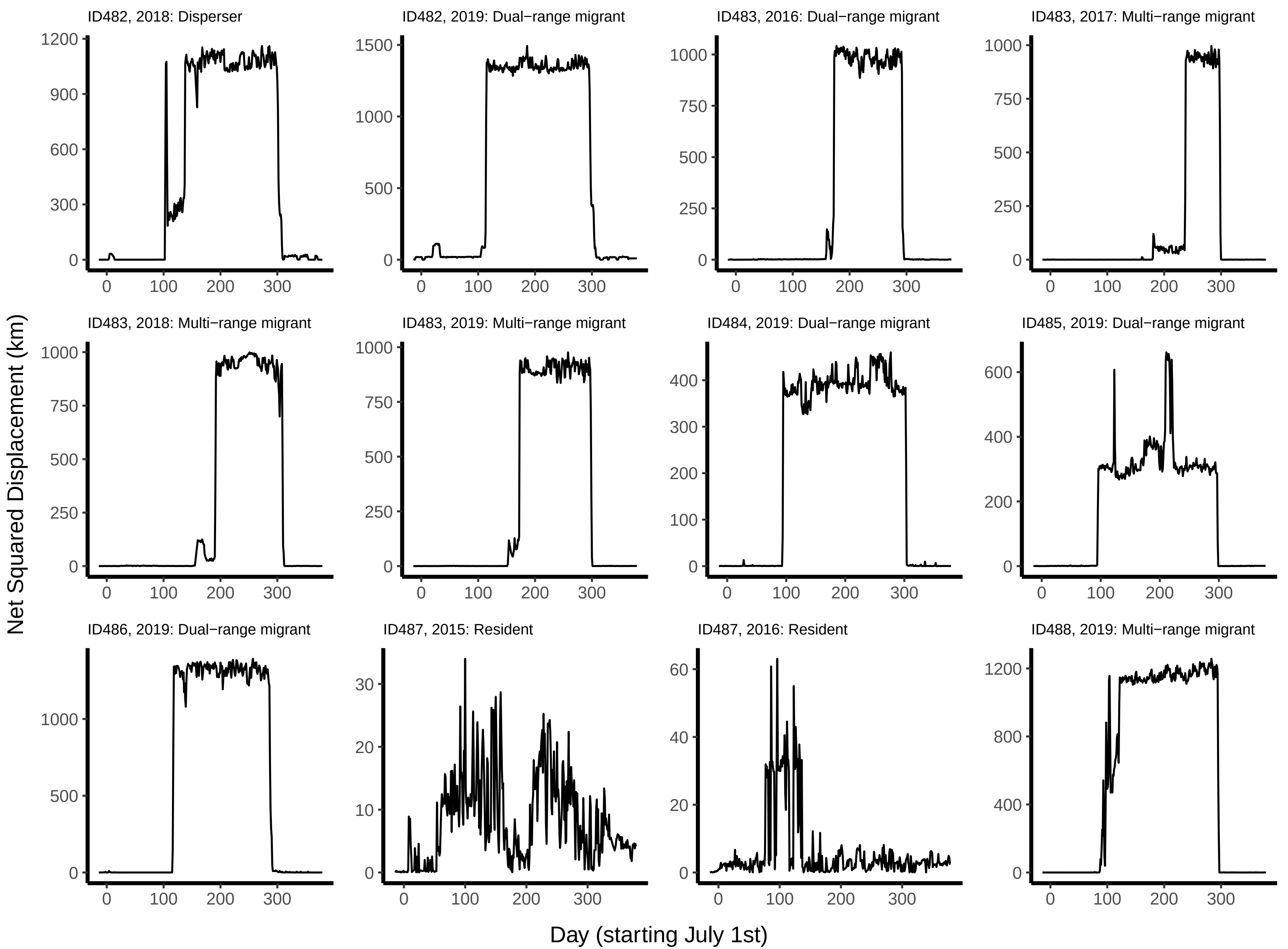

ID482, 2018: Disperser
ID482, 2019: Dual−range migrant
ID483, 2016: Dual−range migrant
ID483, 2017: Multi−range migrant
ID483, 2018: Multi−range migrant
ID483, 2019: Multi−range migrant
ID484, 2019: Dual−range migrant
ID485, 2019: Dual−range migrant
ID486, 2019: Dual−range migrant
ID487, 2015: Resident
ID487, 2016: Resident
ID488, 2019: Multi−range migrant
Net Squared Displacement (km)
Day (starting July 1st)

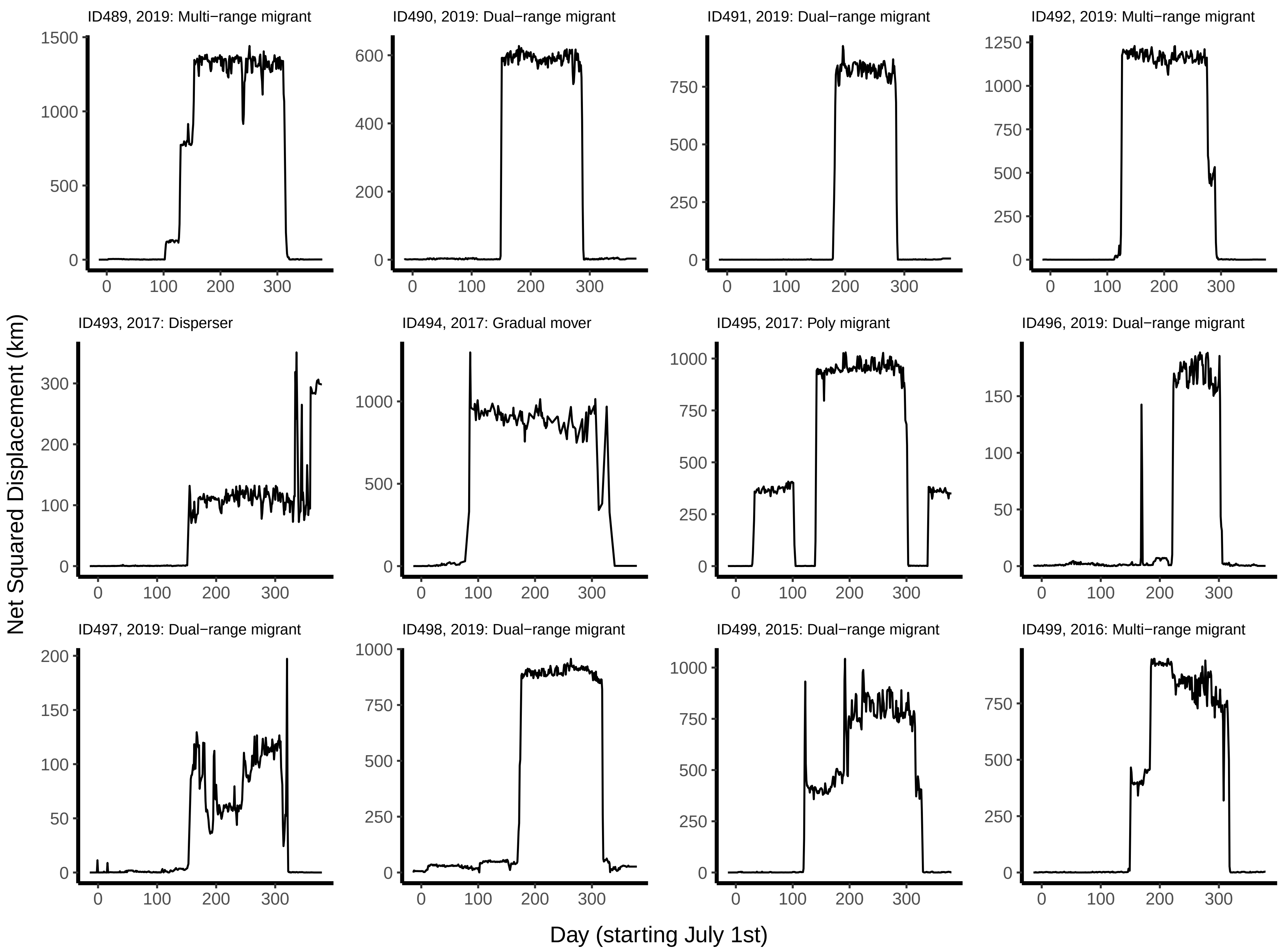

ID489, 2019: Multi−range migrant
ID490, 2019: Dual−range migrant
ID491, 2019: Dual−range migrant
ID492, 2019: Multi−range migrant
ID493, 2017: Disperser
ID494, 2017: Gradual mover
ID495, 2017: Poly migrant
ID496, 2019: Dual−range migrant
ID497, 2019: Dual−range migrant
ID498, 2019: Dual−range migrant
ID499, 2015: Dual−range migrant
ID499, 2016: Multi−range migrant
Net Squared Displacement (km)
Day (starting July 1st)

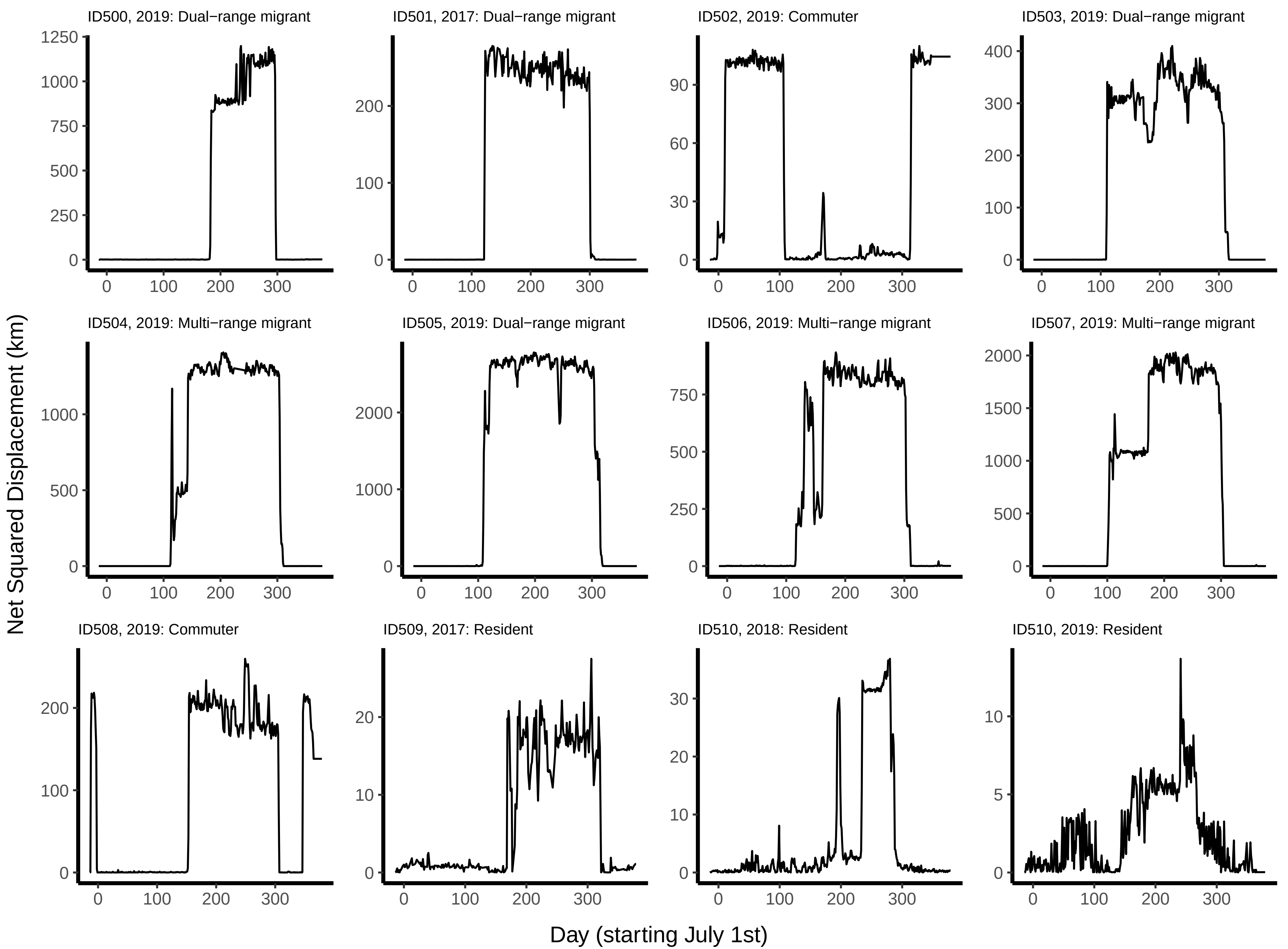

ID500, 2019: Dual−range migrant
ID501, 2017: Dual−range migrant
ID502, 2019: Commuter
ID503, 2019: Dual−range migrant
ID504, 2019: Multi−range migrant
ID505, 2019: Dual−range migrant
ID506, 2019: Multi−range migrant
ID507, 2019: Multi−range migrant
ID508, 2019: Commuter
ID509, 2017: Resident
ID510, 2018: Resident
ID510, 2019: Resident
Net Squared Displacement (km)
Day (starting July 1st)

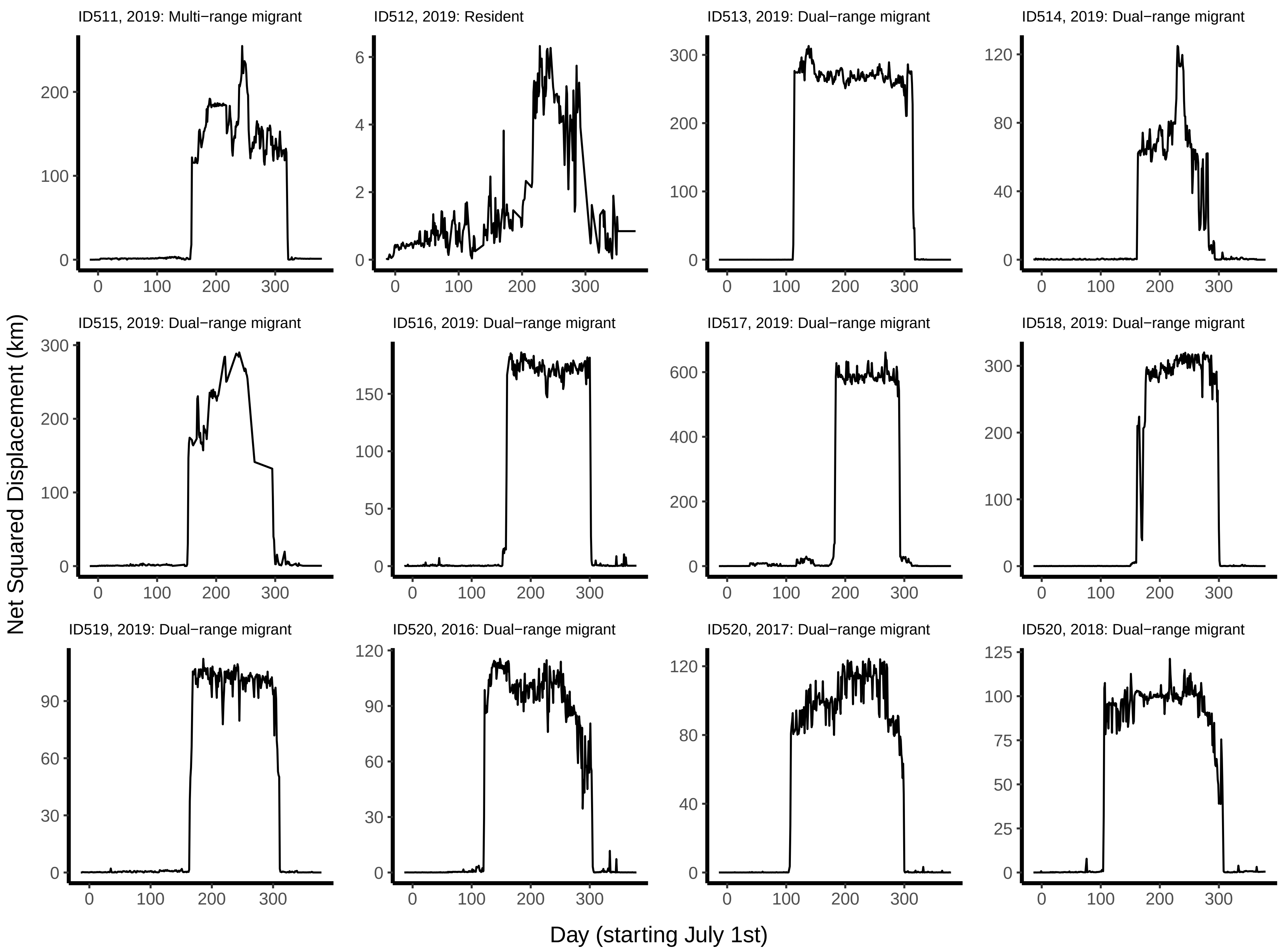
ID511, 2019: Multi−range migrant
ID512, 2019: Resident
ID513, 2019: Dual−range migrant
ID514, 2019: Dual−range migrant
ID515, 2019: Dual−range migrant
ID516, 2019: Dual−range migrant
ID517, 2019: Dual−range migrant
ID518, 2019: Dual−range migrant
ID519, 2019: Dual−range migrant
ID520, 2016: Dual−range migrant
ID520, 2017: Dual−range migrant
ID520, 2018: Dual−range migrant
Net Squared Displacement (km)
Day (starting July 1st)

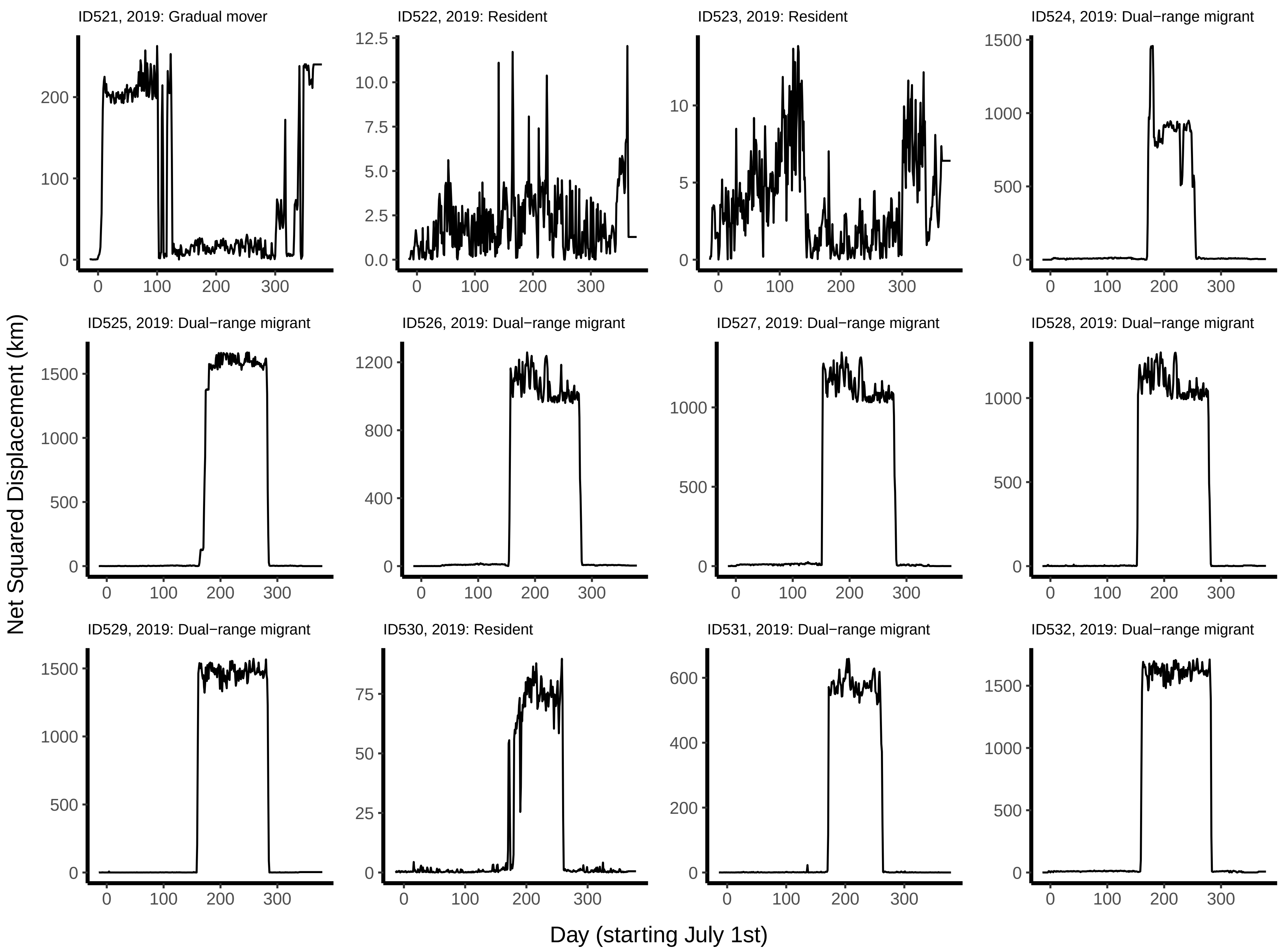
ID521, 2019: Gradual mover
ID522, 2019: Resident
ID523, 2019: Resident
ID524, 2019: Dual−range migrant
ID525, 2019: Dual−range migrant
ID526, 2019: Dual−range migrant
ID527, 2019: Dual−range migrant
ID528, 2019: Dual−range migrant
ID529, 2019: Dual−range migrant
ID530, 2019: Resident
ID531, 2019: Dual−range migrant
ID532, 2019: Dual−range migrant
Net Squared Displacement (km)
Day (starting July 1st)

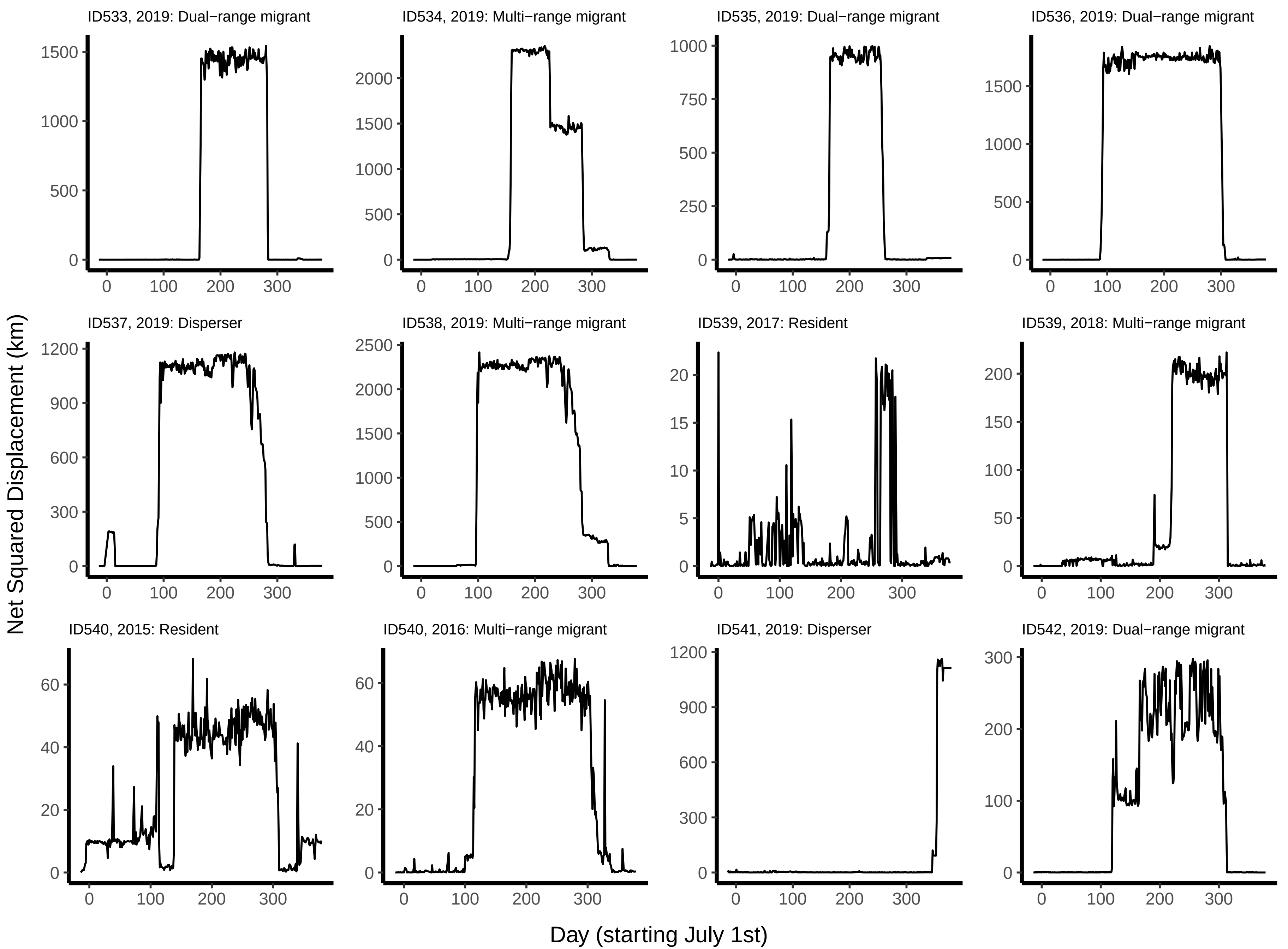
ID533, 2019: Dual−range migrant
ID534, 2019: Multi−range migrant
ID535, 2019: Dual−range migrant
ID536, 2019: Dual−range migrant
ID537, 2019: Disperser
ID538, 2019: Multi−range migrant
ID539, 2017: Resident
ID539, 2018: Multi−range migrant
ID540, 2015: Resident
ID540, 2016: Multi−range migrant
ID541, 2019: Disperser
ID542, 2019: Dual−range migrant
Net Squared Displacement (km)
Day (starting July 1st)

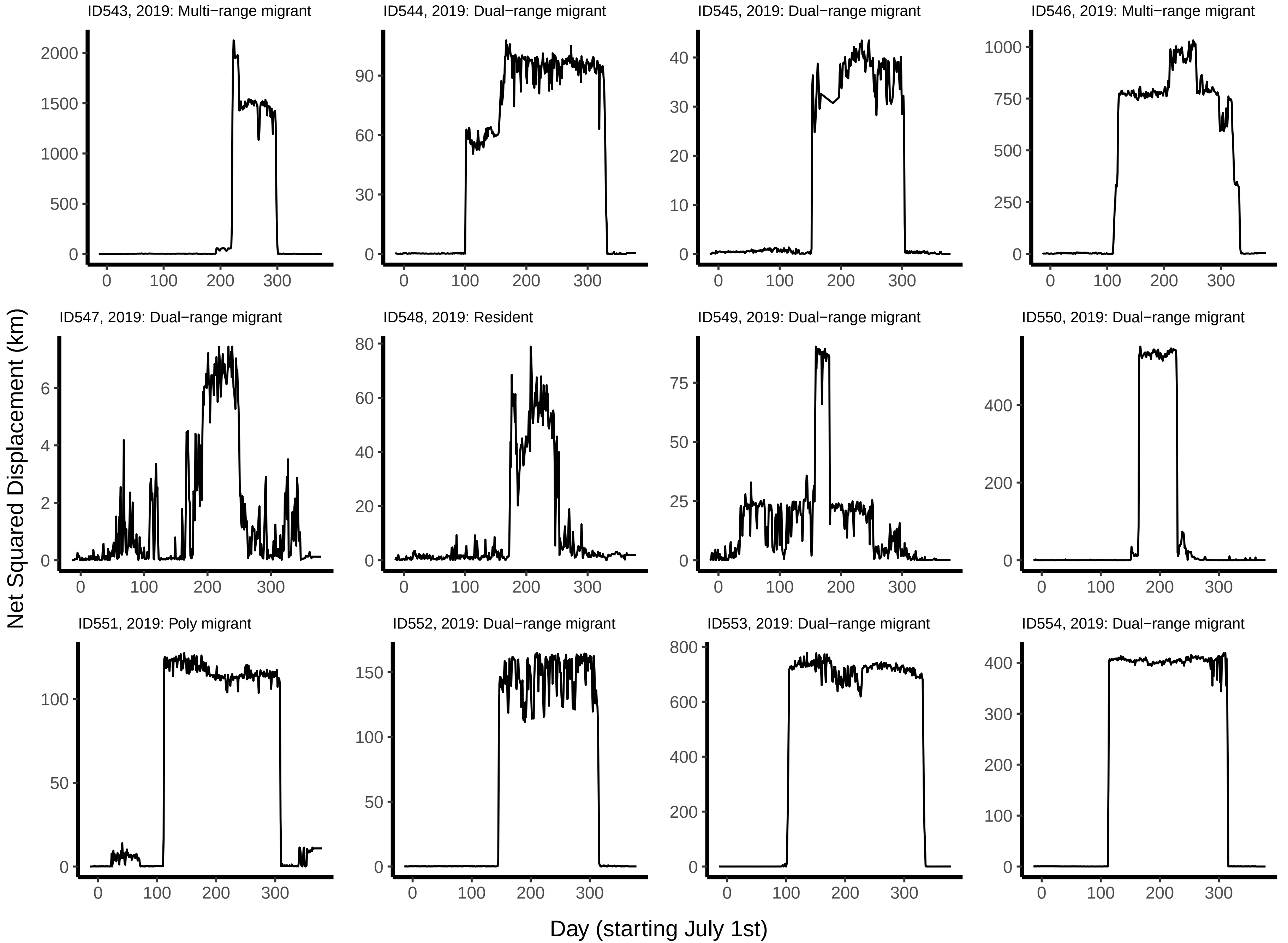
ID543, 2019: Multi−range migrant
ID544, 2019: Dual−range migrant
ID545, 2019: Dual−range migrant
ID546, 2019: Multi−range migrant
ID547, 2019: Dual−range migrant
ID548, 2019: Resident
ID549, 2019: Dual−range migrant
ID550, 2019: Dual−range migrant
ID551, 2019: Poly migrant
ID552, 2019: Dual−range migrant
ID553, 2019: Dual−range migrant
ID554, 2019: Dual−range migrant
Net Squared Displacement (km)
Day (starting July 1st)

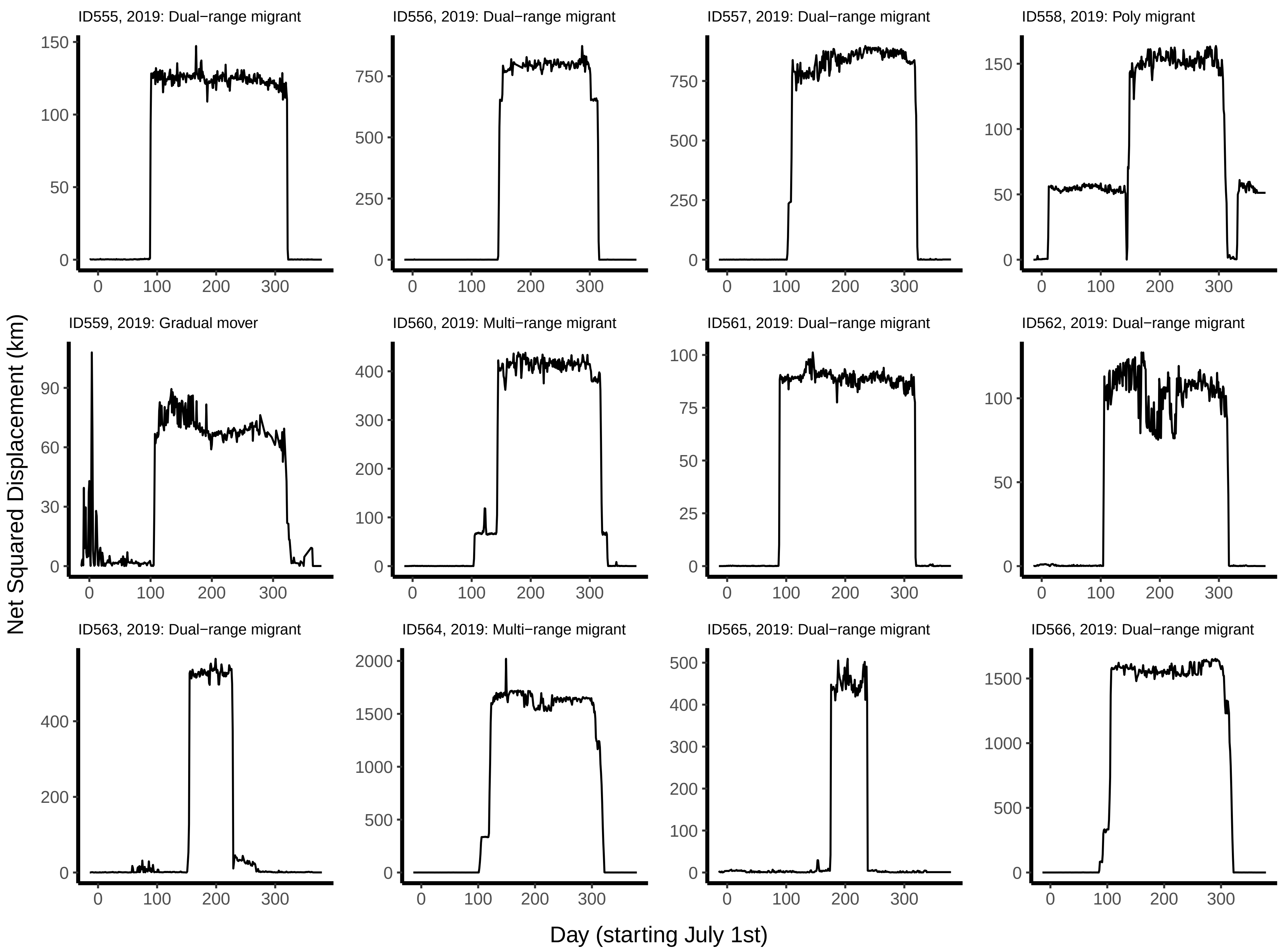

ID555, 2019: Dual−range migrant
ID556, 2019: Dual−range migrant
ID557, 2019: Dual−range migrant
ID558, 2019: Poly migrant
ID559, 2019: Gradual mover
ID560, 2019: Multi−range migrant
ID561, 2019: Dual−range migrant
ID562, 2019: Dual−range migrant
ID563, 2019: Dual−range migrant
ID564, 2019: Multi−range migrant
ID565, 2019: Dual−range migrant
ID566, 2019: Dual−range migrant
Net Squared Displacement (km)
Day (starting July 1st)

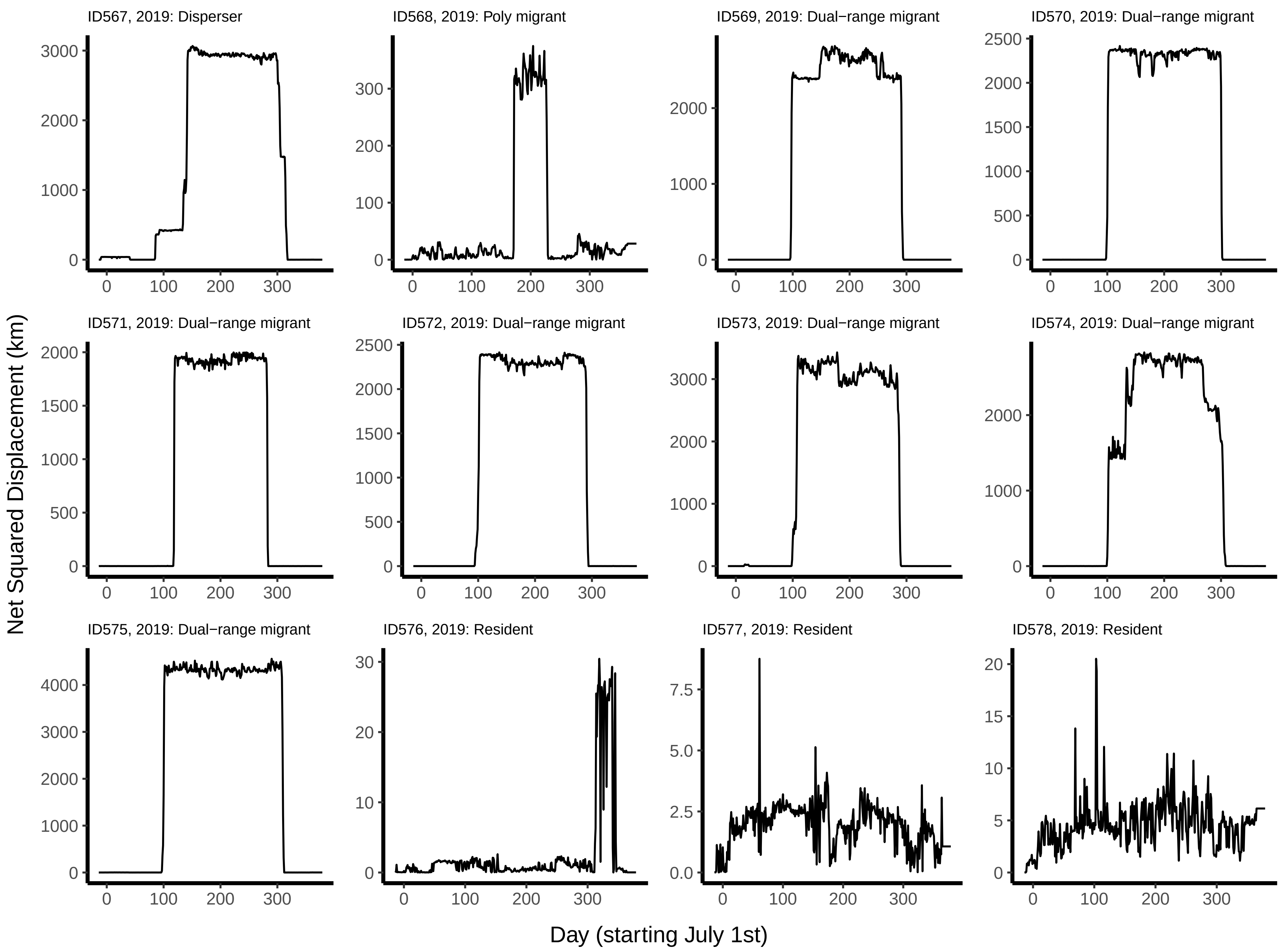
ID567, 2019: Disperser
ID568, 2019: Poly migrant
ID569, 2019: Dual−range migrant
ID570, 2019: Dual−range migrant
ID571, 2019: Dual−range migrant
ID572, 2019: Dual−range migrant
ID573, 2019: Dual−range migrant
ID574, 2019: Dual−range migrant
ID575, 2019: Dual−range migrant
ID576, 2019: Resident
ID577, 2019: Resident
ID578, 2019: Resident
Net Squared Displacement (km)
Day (starting July 1st)

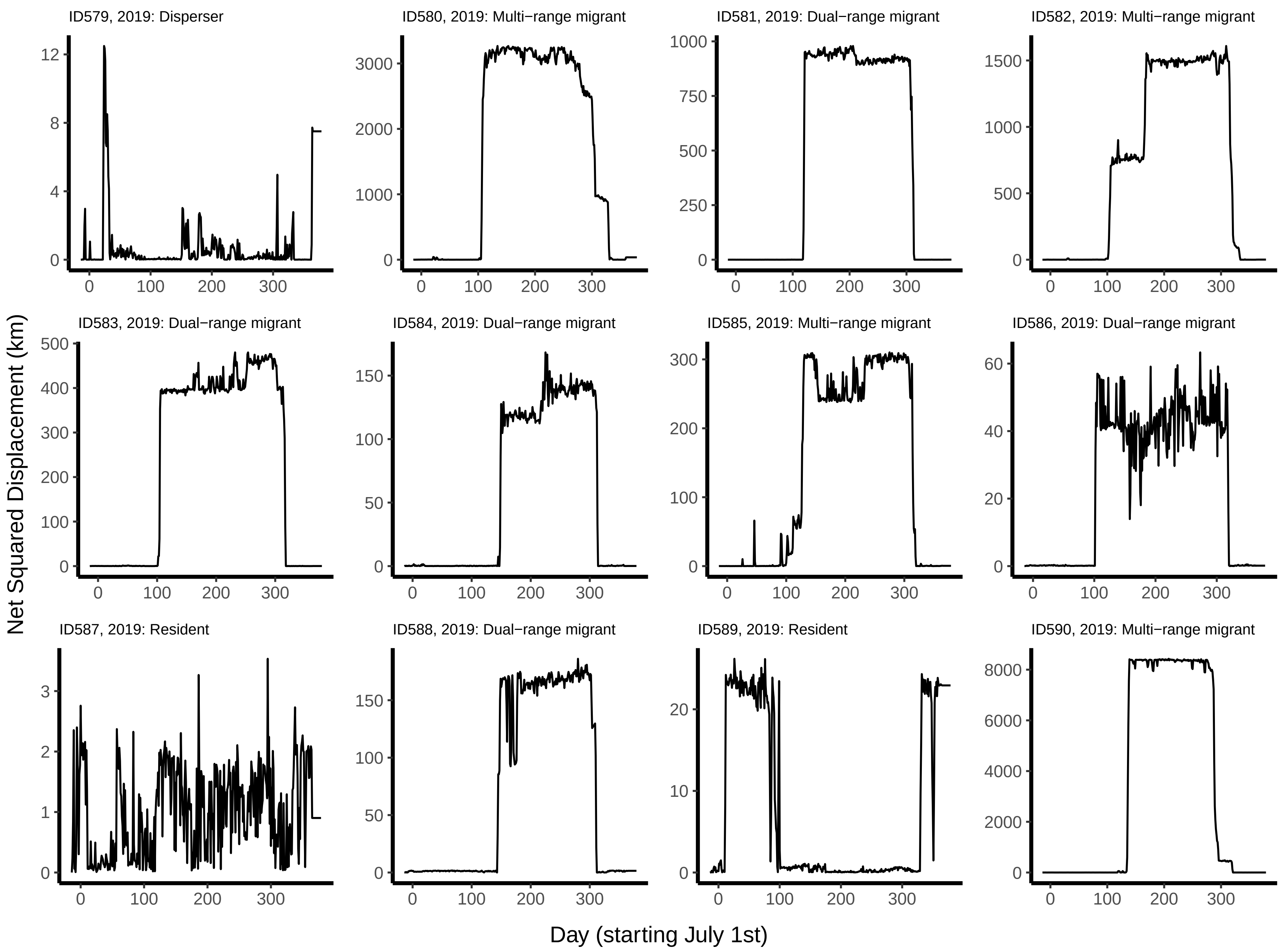

ID579, 2019: Disperser
ID580, 2019: Multi−range migrant
ID581, 2019: Dual−range migrant
ID582, 2019: Multi−range migrant
ID583, 2019: Dual−range migrant
ID584, 2019: Dual−range migrant
ID585, 2019: Multi−range migrant
ID586, 2019: Dual−range migrant
ID587, 2019: Resident
ID588, 2019: Dual−range migrant
ID589, 2019: Resident
ID590, 2019: Multi−range migrant
Net Squared Displacement (km)
Day (starting July 1st)

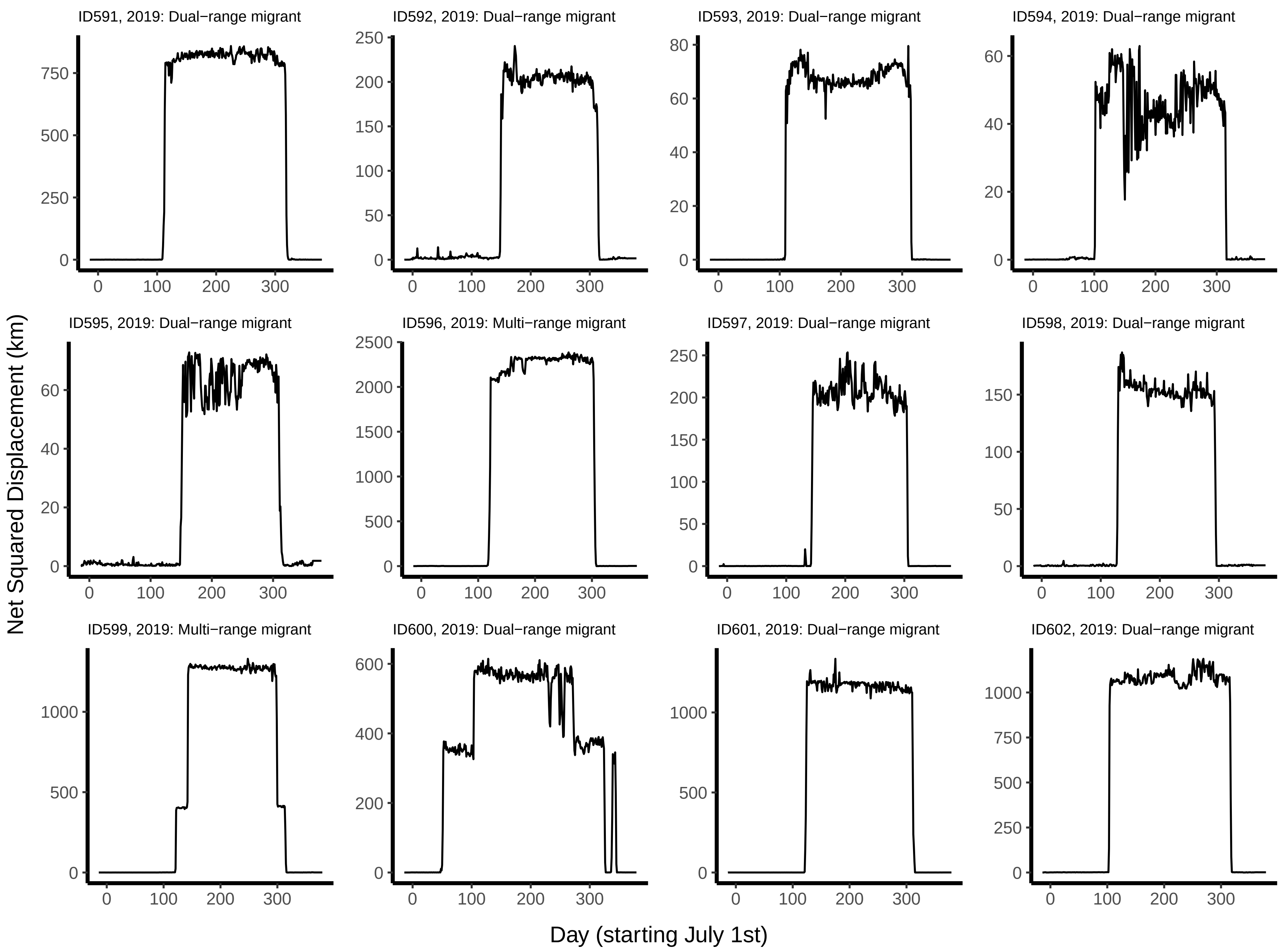
ID591, 2019: Dual−range migrant
ID592, 2019: Dual−range migrant
ID593, 2019: Dual−range migrant
ID594, 2019: Dual−range migrant
ID595, 2019: Dual−range migrant
ID596, 2019: Multi−range migrant
ID597, 2019: Dual−range migrant
ID598, 2019: Dual−range migrant
ID599, 2019: Multi−range migrant
ID600, 2019: Dual−range migrant
ID601, 2019: Dual−range migrant
ID602, 2019: Dual−range migrant
Net Squared Displacement (km)
Day (starting July 1st)

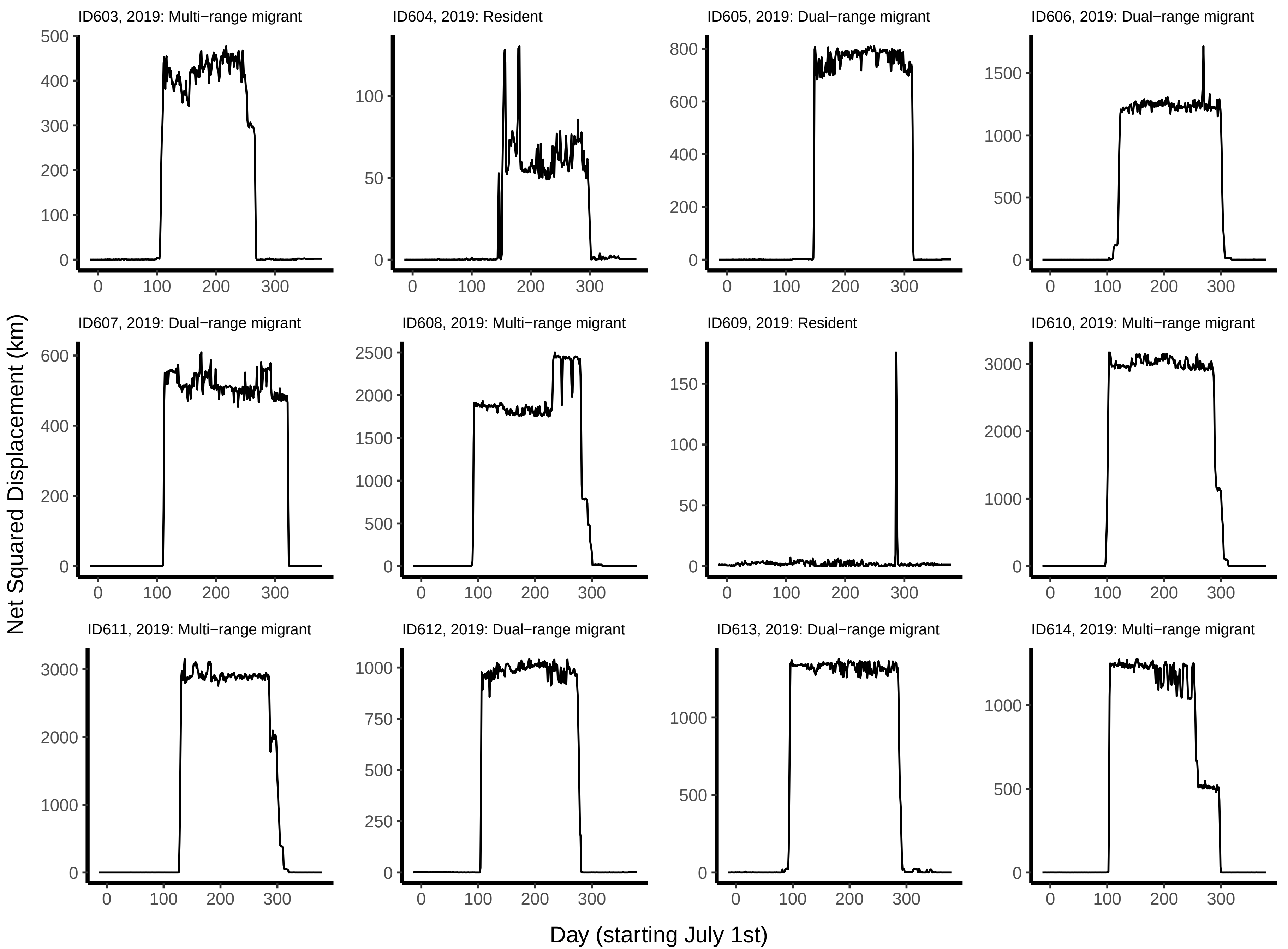
ID603, 2019: Multi−range migrant
ID604, 2019: Resident
ID605, 2019: Dual−range migrant
ID606, 2019: Dual−range migrant
ID607, 2019: Dual−range migrant
ID608, 2019: Multi−range migrant
ID609, 2019: Resident
ID610, 2019: Multi−range migrant
ID611, 2019: Multi−range migrant
ID612, 2019: Dual−range migrant
ID613, 2019: Dual−range migrant
ID614, 2019: Multi−range migrant
Net Squared Displacement (km)
Day (starting July 1st)

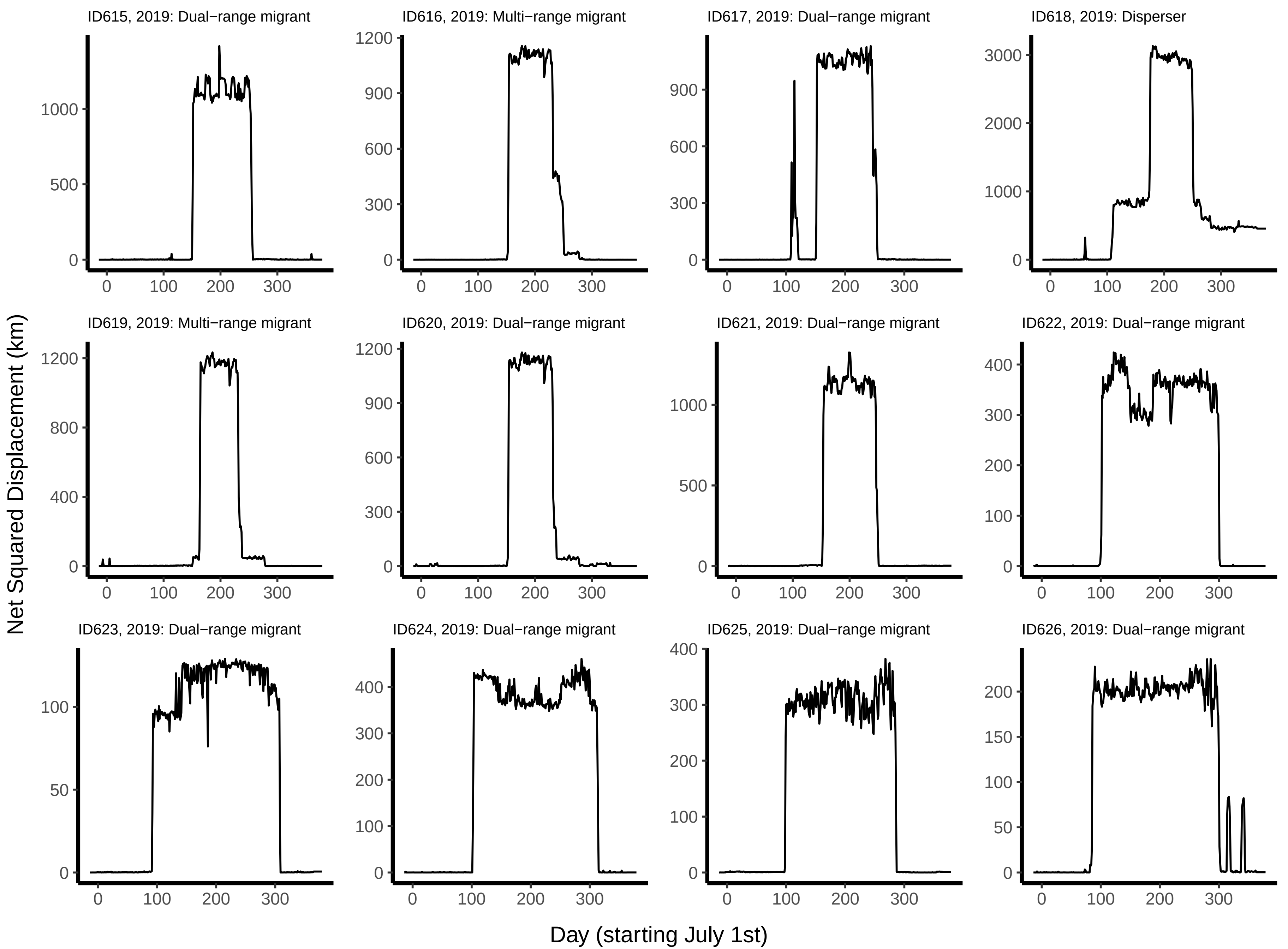

ID615, 2019: Dual−range migrant
ID616, 2019: Multi−range migrant
ID617, 2019: Dual−range migrant
ID618, 2019: Disperser
ID619, 2019: Multi−range migrant
ID620, 2019: Dual−range migrant
ID621, 2019: Dual−range migrant
ID622, 2019: Dual−range migrant
ID623, 2019: Dual−range migrant
ID624, 2019: Dual−range migrant
ID625, 2019: Dual−range migrant
ID626, 2019: Dual−range migrant
Net Squared Displacement (km)
Day (starting July 1st)

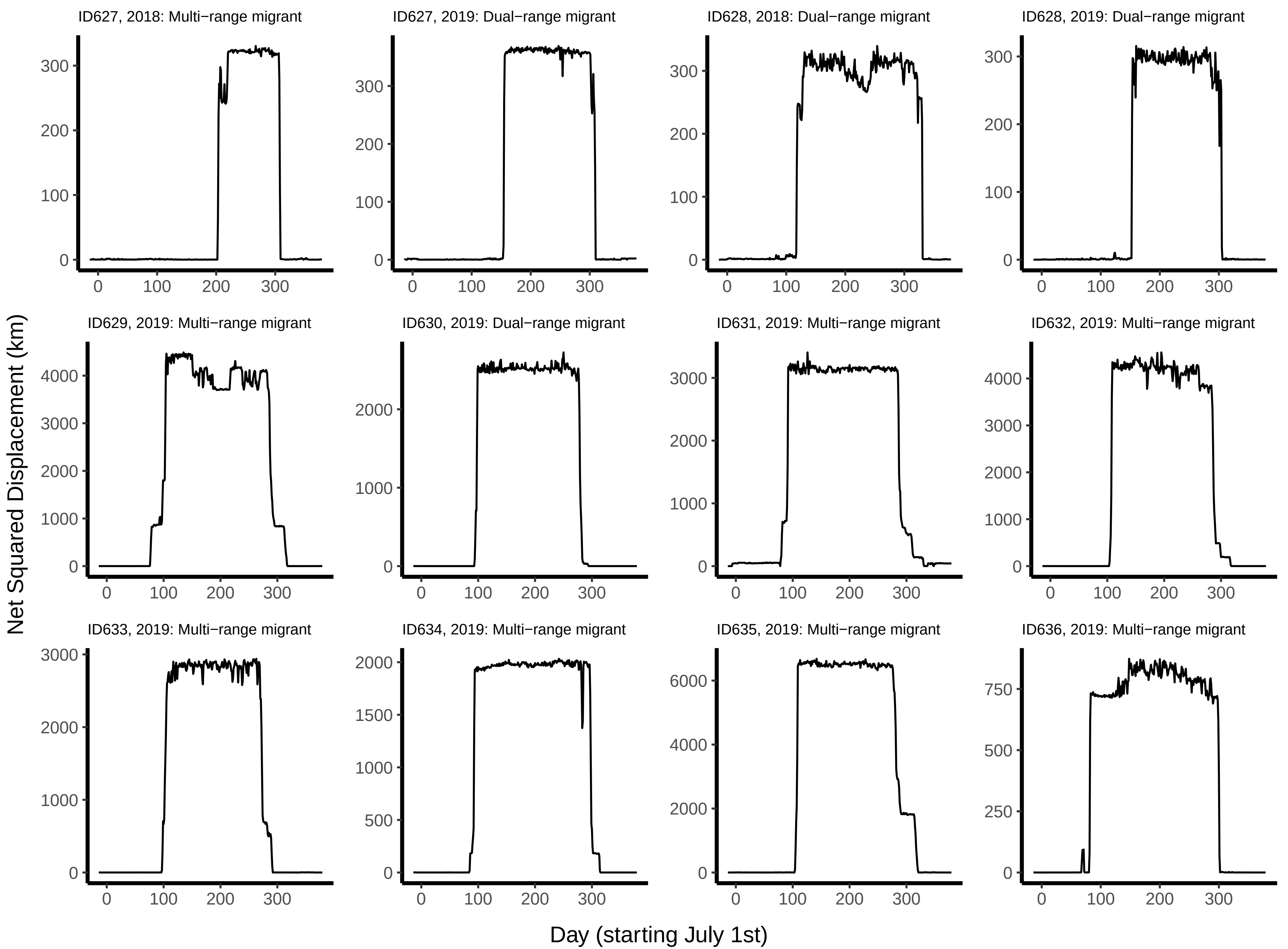

ID627, 2018: Multi−range migrant
ID627, 2019: Dual−range migrant
ID628, 2018: Dual−range migrant
ID628, 2019: Dual−range migrant
ID629, 2019: Multi−range migrant
ID630, 2019: Dual−range migrant
ID631, 2019: Multi−range migrant
ID632, 2019: Multi−range migrant
ID633, 2019: Multi−range migrant
ID634, 2019: Multi−range migrant
ID635, 2019: Multi−range migrant
ID636, 2019: Multi−range migrant
Net Squared Displacement (km)
Day (starting July 1st)

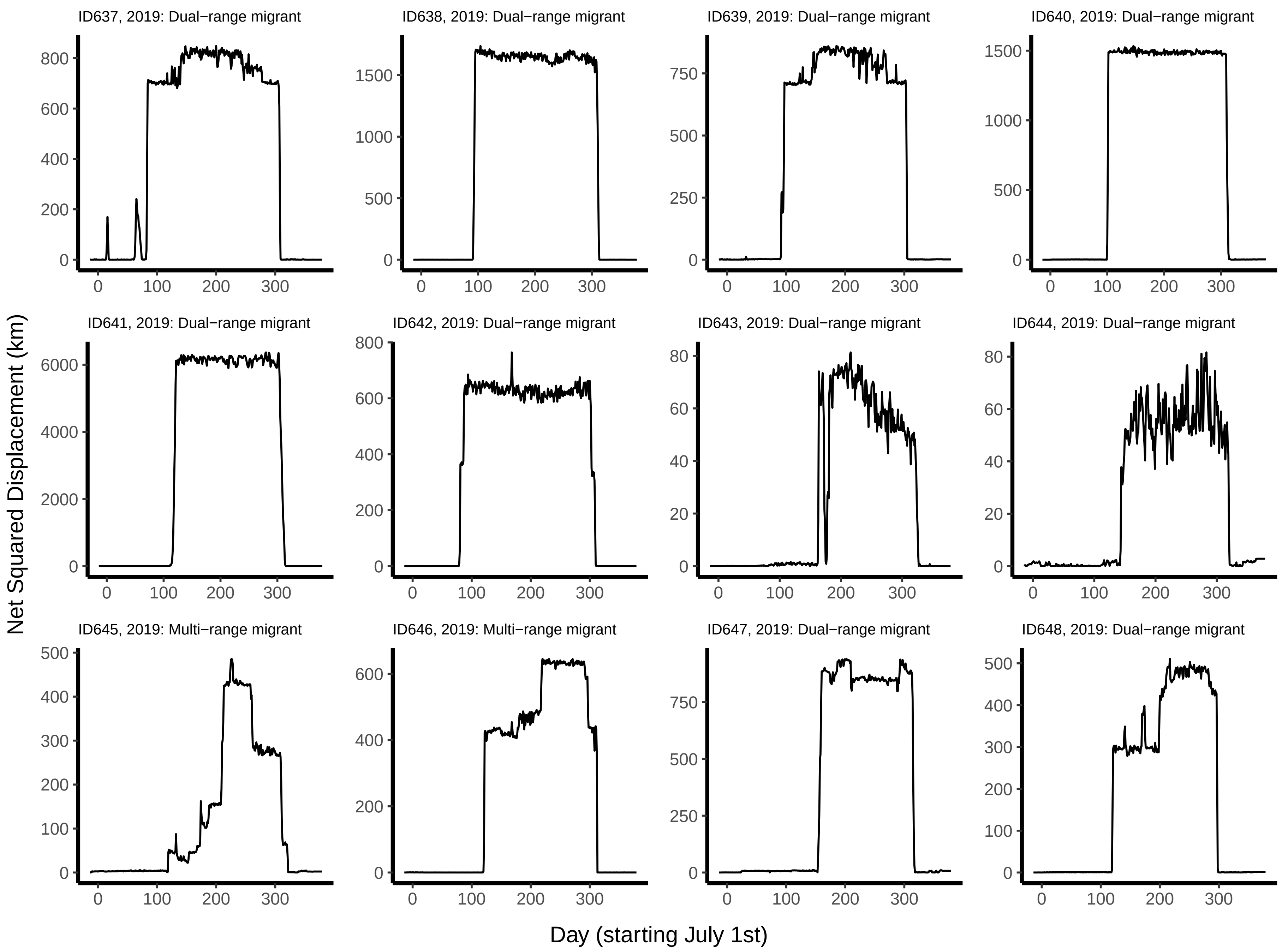

ID637, 2019: Dual−range migrant
ID638, 2019: Dual−range migrant
ID639, 2019: Dual−range migrant
ID640, 2019: Dual−range migrant
ID641, 2019: Dual−range migrant
ID642, 2019: Dual−range migrant
ID643, 2019: Dual−range migrant
ID644, 2019: Dual−range migrant
ID645, 2019: Multi−range migrant
ID646, 2019: Multi−range migrant
ID647, 2019: Dual−range migrant
ID648, 2019: Dual−range migrant
Net Squared Displacement (km)
Day (starting July 1st)

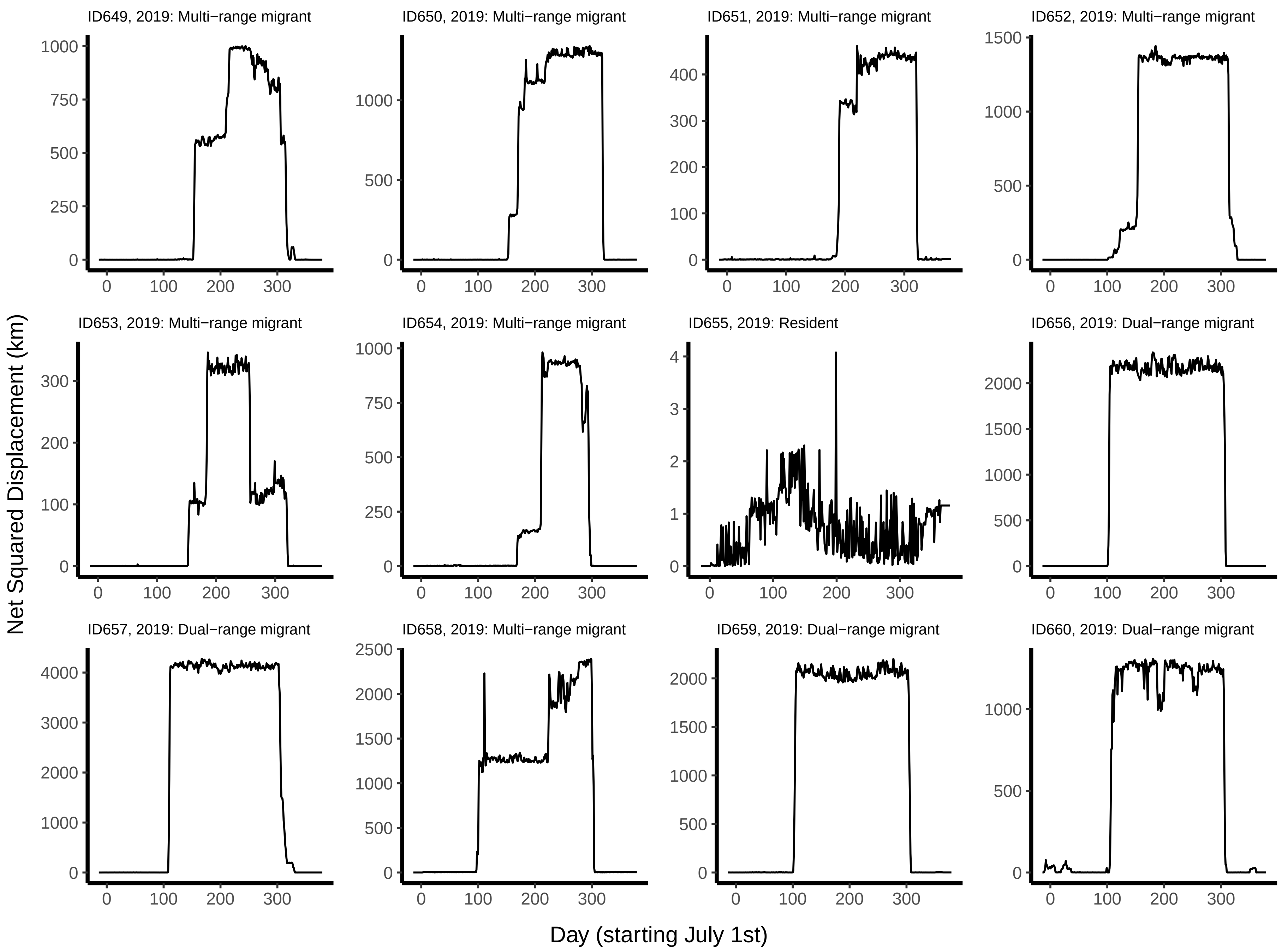

ID649, 2019: Multi−range migrant
ID650, 2019: Multi−range migrant
ID651, 2019: Multi−range migrant
ID652, 2019: Multi−range migrant
ID653, 2019: Multi−range migrant
ID654, 2019: Multi−range migrant
ID655, 2019: Resident
ID656, 2019: Dual−range migrant
ID657, 2019: Dual−range migrant
ID658, 2019: Multi−range migrant
ID659, 2019: Dual−range migrant
ID660, 2019: Dual−range migrant
Net Squared Displacement (km)
Day (starting July 1st)

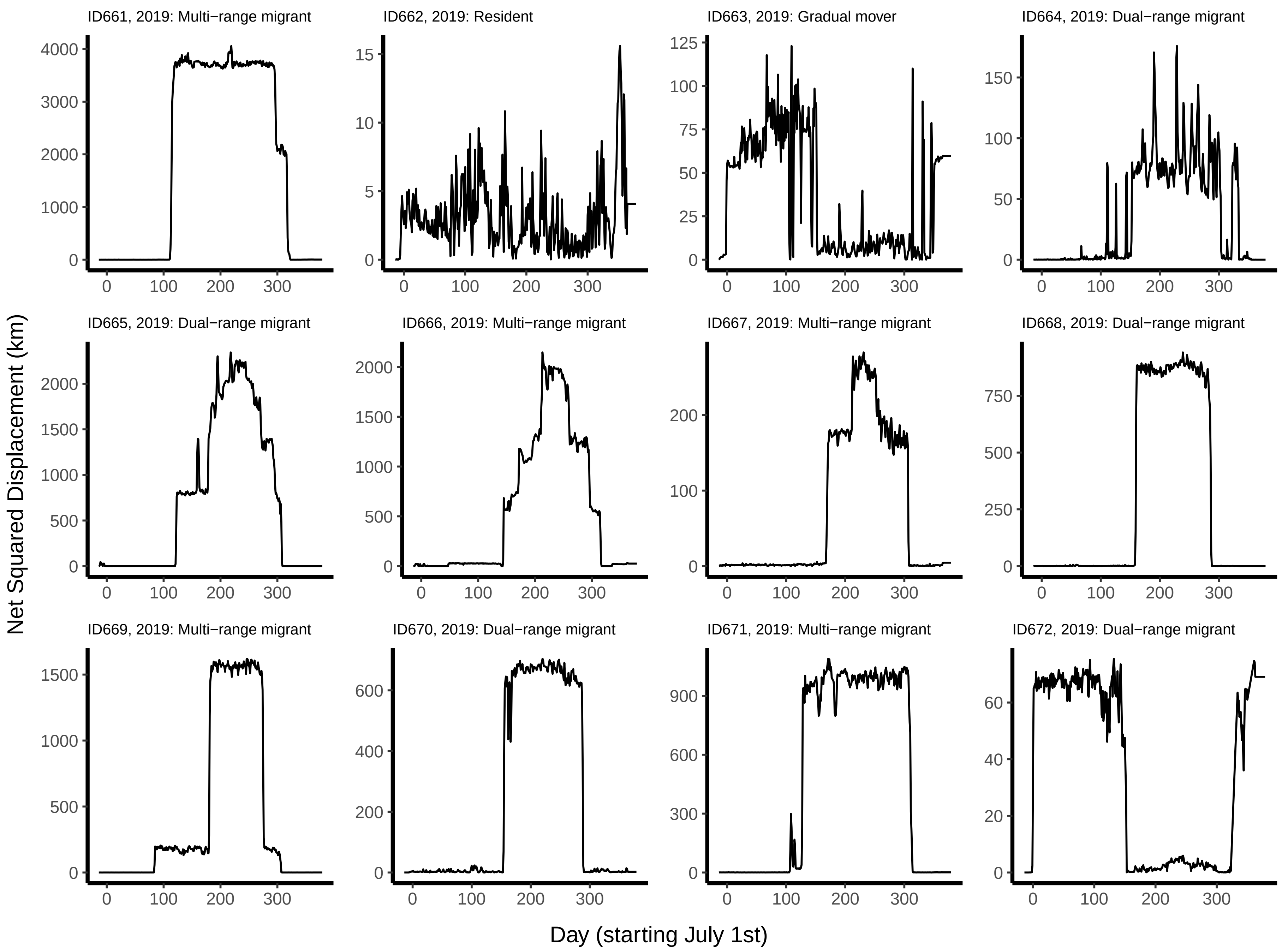

ID661, 2019: Multi−range migrant
ID662, 2019: Resident
ID663, 2019: Gradual mover
ID664, 2019: Dual−range migrant
ID665, 2019: Dual−range migrant
ID666, 2019: Multi−range migrant
ID667, 2019: Multi−range migrant
ID668, 2019: Dual−range migrant
ID669, 2019: Multi−range migrant
ID670, 2019: Dual−range migrant
ID671, 2019: Multi−range migrant
ID672, 2019: Dual−range migrant
Net Squared Displacement (km)
Day (starting July 1st)

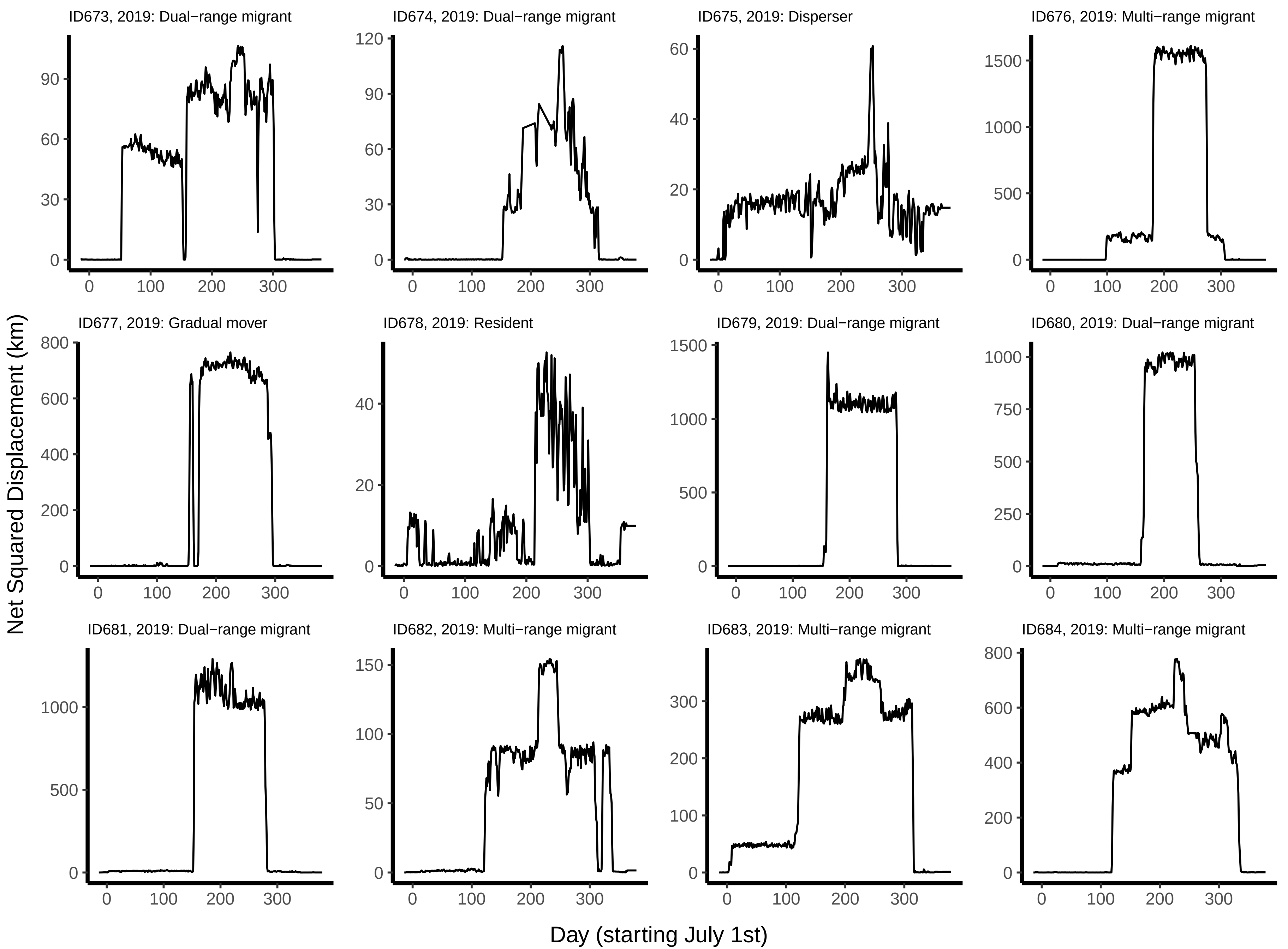

ID673, 2019: Dual−range migrant
ID674, 2019: Dual−range migrant
ID675, 2019: Disperser
ID676, 2019: Multi−range migrant
ID677, 2019: Gradual mover
ID678, 2019: Resident
ID679, 2019: Dual−range migrant
ID680, 2019: Dual−range migrant
ID681, 2019: Dual−range migrant
ID682, 2019: Multi−range migrant
ID683, 2019: Multi−range migrant
ID684, 2019: Multi−range migrant
Net Squared Displacement (km)
Day (starting July 1st)

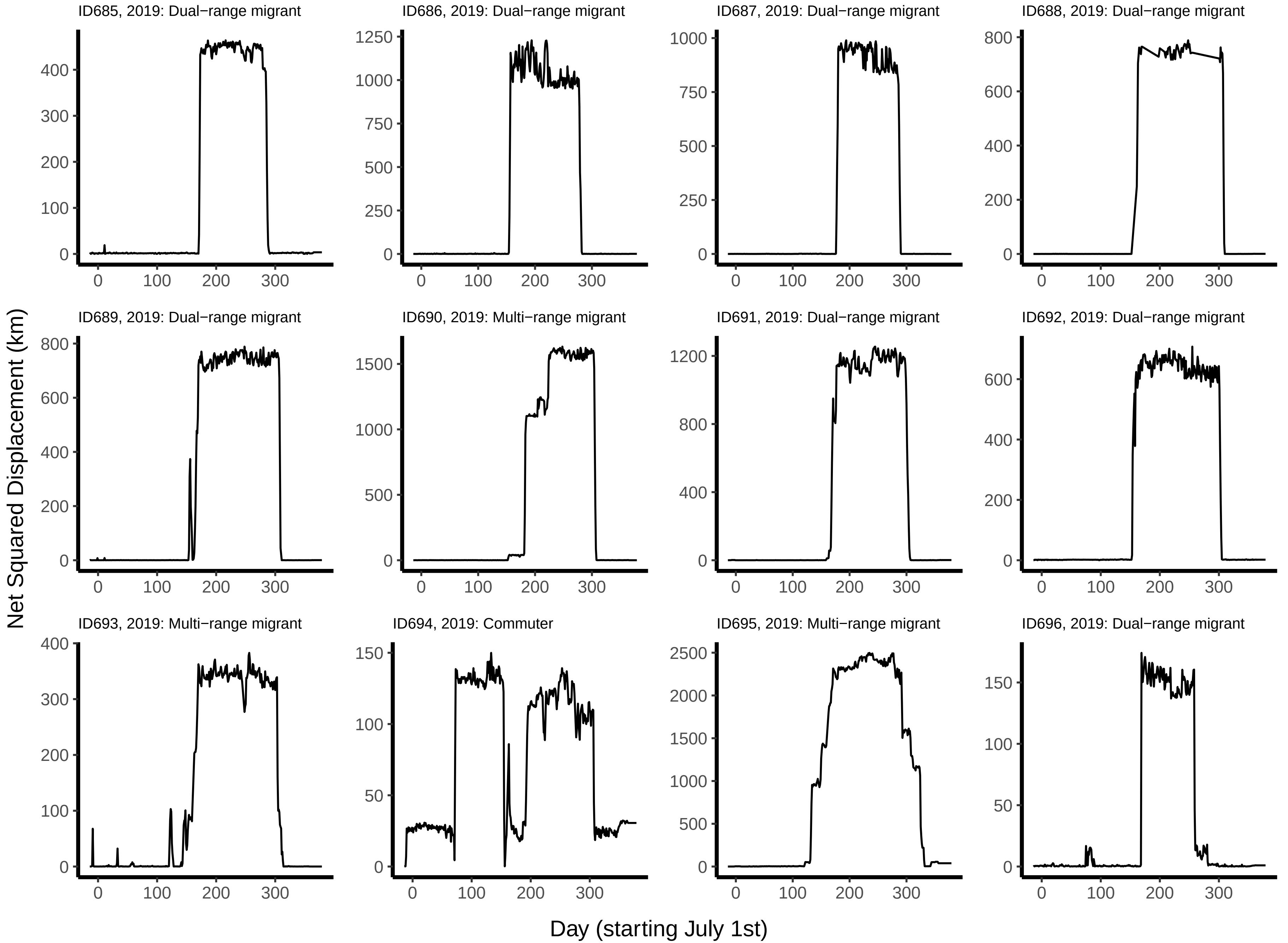

ID685, 2019: Dual−range migrant
ID686, 2019: Dual−range migrant
ID687, 2019: Dual−range migrant
ID688, 2019: Dual−range migrant
ID689, 2019: Dual−range migrant
ID690, 2019: Multi−range migrant
ID691, 2019: Dual−range migrant
ID692, 2019: Dual−range migrant
ID693, 2019: Multi−range migrant
ID694, 2019: Commuter
ID695, 2019: Multi−range migrant
ID696, 2019: Dual−range migrant
Net Squared Displacement (km)
Day (starting July 1st)

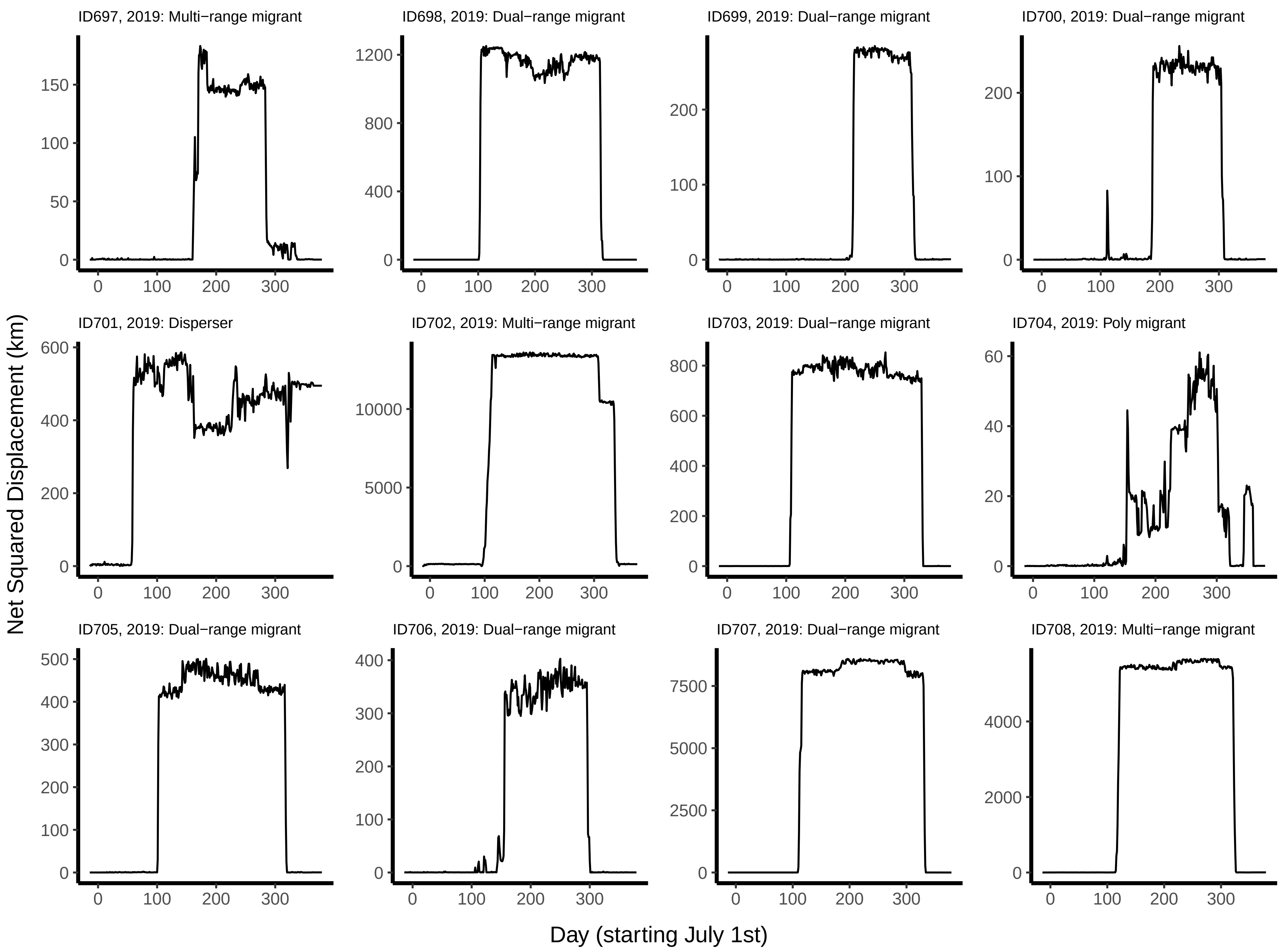

ID697, 2019: Multi−range migrant
ID698, 2019: Dual−range migrant
ID699, 2019: Dual−range migrant
ID700, 2019: Dual−range migrant
ID701, 2019: Disperser
ID702, 2019: Multi−range migrant
ID703, 2019: Dual−range migrant
ID704, 2019: Poly migrant
ID705, 2019: Dual−range migrant
ID706, 2019: Dual−range migrant
ID707, 2019: Dual−range migrant
ID708, 2019: Multi−range migrant
Net Squared Displacement (km)
Day (starting July 1st)

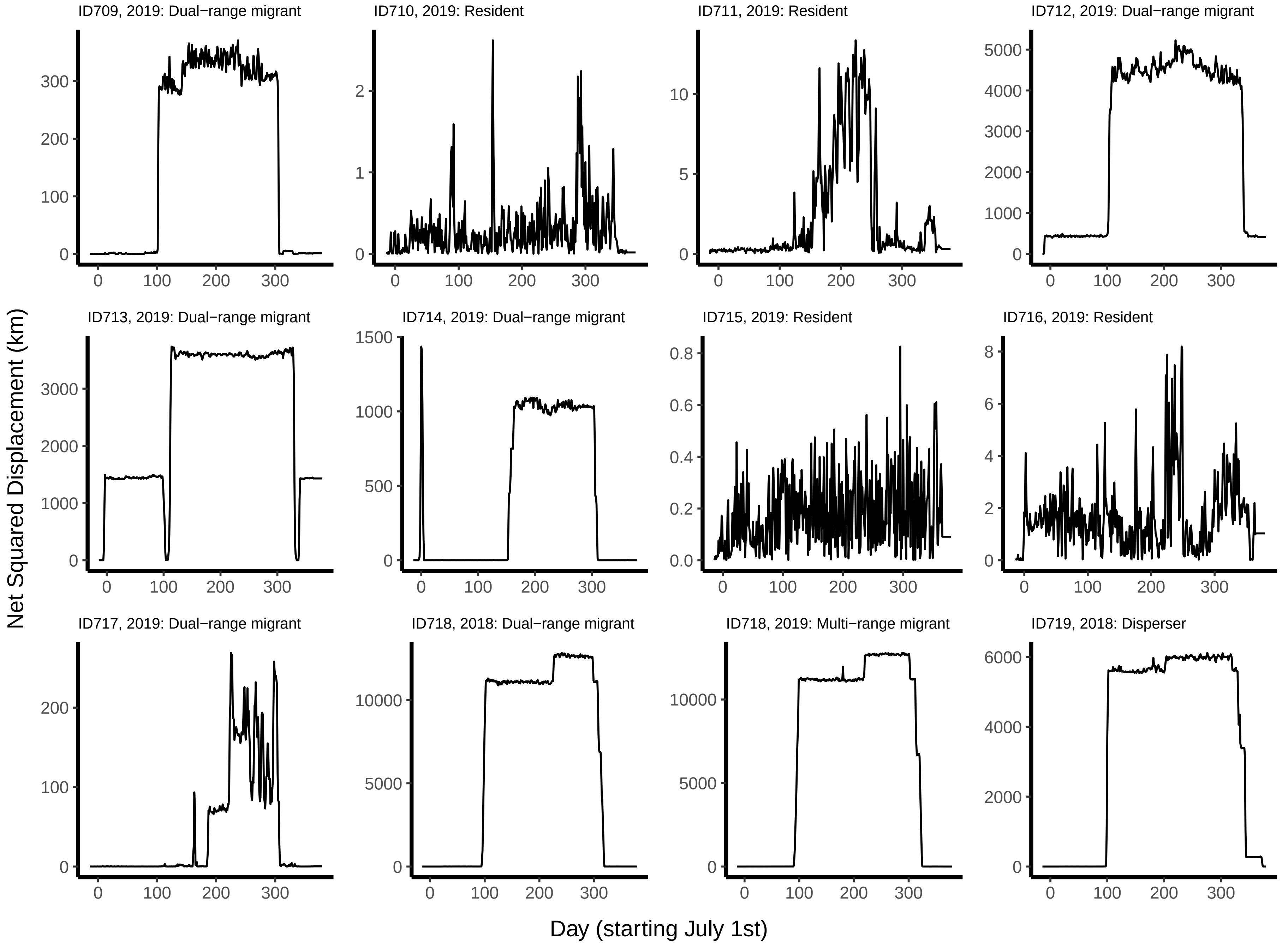

ID709, 2019: Dual−range migrant
ID710, 2019: Resident
ID711, 2019: Resident
ID712, 2019: Dual−range migrant
ID713, 2019: Dual−range migrant
ID714, 2019: Dual−range migrant
ID715, 2019: Resident
ID716, 2019: Resident
ID717, 2019: Dual−range migrant
ID718, 2018: Dual−range migrant
ID718, 2019: Multi−range migrant
ID719, 2018: Disperser
Net Squared Displacement (km)
Day (starting July 1st)

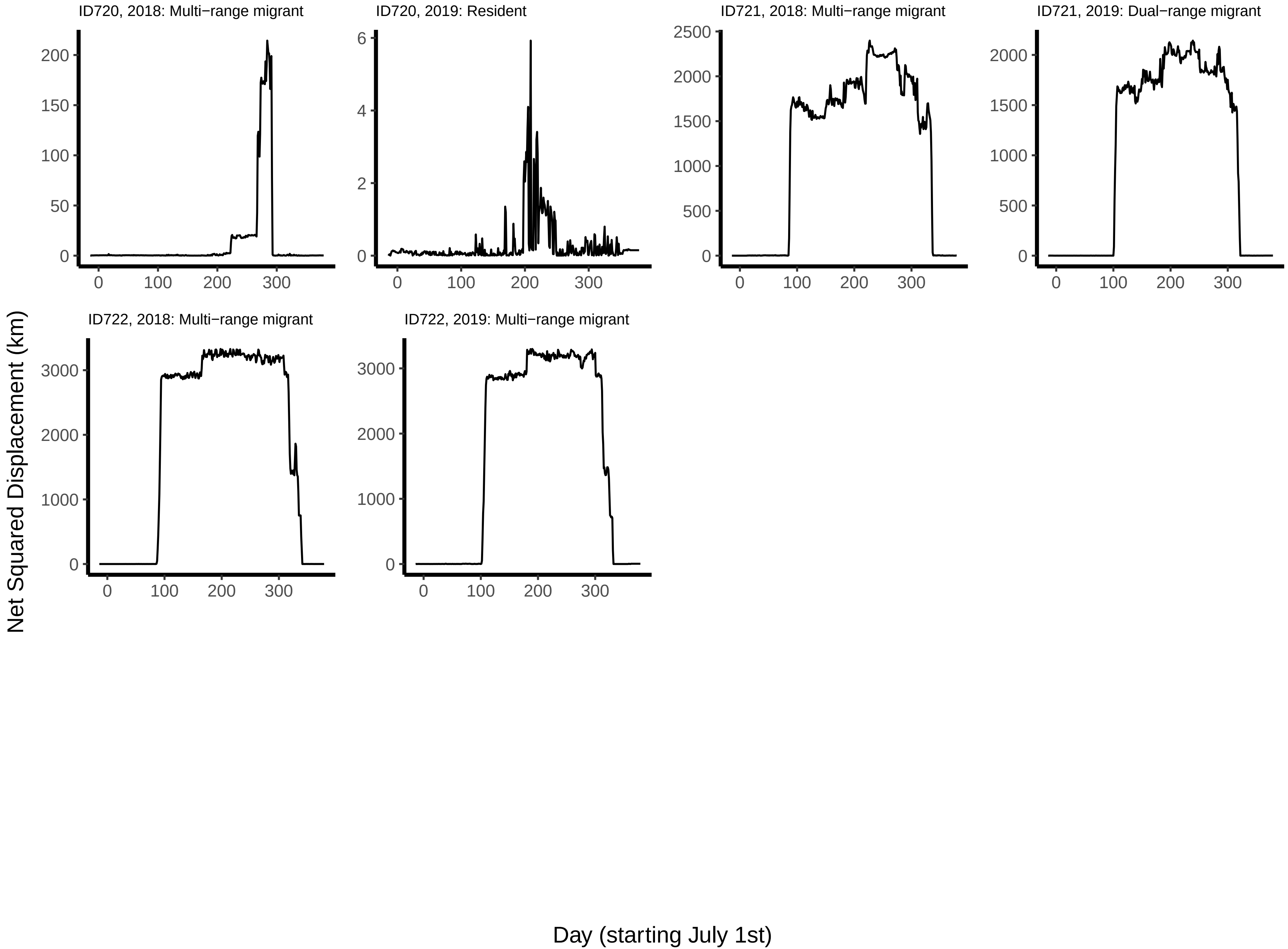

ID720, 2018: Multi−range migrant
ID720, 2019: Resident
ID721, 2018: Multi−range migrant
ID721, 2019: Dual−range migrant
ID722, 2018: Multi−range migrant
ID722, 2019: Multi−range migrant
Net Squared Displacement (km)
Day (starting July 1st)
